# Prioritizing smallholder animal health needs in East Africa, West Africa, and South Asia using three approaches: Literature review, expert workshops, and practitioner surveys

**DOI:** 10.1016/j.prevetmed.2021.105279

**Published:** 2021-04

**Authors:** Zoë Campbell, Paul Coleman, Andrea Guest, Peetambar Kushwaha, Thembinkosi Ramuthivheli, Tom Osebe, Brian Perry, Jeremy Salt

**Affiliations:** a International Livestock Research Institute (ILRI), P.O. Box 30709, Nairobi, 00100, Kenya; b H20 Venture Partners, 33-35 George Street, Oxford, OX1 2AY, United Kingdom; c GALVmed Asia Office, Unit 118 & 120 B, Splendor Forum, Plot No 3, Jasola District Centre, Jasola, New Delhi, 110025, India; d GALVmed Africa Office, International Livestock Research Institute (ILRI), Swing One, Naivasha Road, Nairobi, Kenya; e Nuffield College of Clinical Medicine, University of Oxford, United Kingdom; f College of Medicine and Veterinary Medicine, University of Edinburgh, Arthurstone House, Meigle, Blairgowrie, PH12 8QW, Scotland, United Kingdom; g GALVmed UK Office, Doherty Building, Pentlands Science Park, Bush Loan, Penicuik Edinburgh, EH26 0PZ, Scotland, United Kingdom

**Keywords:** Veterinary, Livestock disease, Global animal health, Poor, Poverty, Prioritisation

## Abstract

Managing the health needs of livestock contributes to reducing poverty and improving the livelihoods of smallholder and pastoralist livestock keepers globally. Animal health practitioners, producers, policymakers, and researchers all must prioritize how to mobilize limited resources. This study employed three approaches to prioritize animal health needs in East and West Africa and South Asia to identify diseases and syndromes that impact livestock keepers. The approaches were a) systematic literature review, b) a series of expert workshops, and c) a practitioner survey of veterinarians and para-veterinary professionals. The top constraints that emerged from all three approaches include endo/ ectoparasites, foot and mouth disease, brucellosis, peste des petits ruminants, Newcastle disease, and avian influenza. Expert workshops additionally identified contagious caprine pleuropneumonia, contagious bovine pleuropneumonia, mastitis, and reproductive disorders as constraints not emphasized in the literature review. Practitioner survey results additionally identified nutrition as a constraint for smallholder dairy and pastoralist small ruminant production. Experts attending the workshops agreed most constraints can be managed using existing veterinary technologies and best husbandry practices, which supports a shift away from focusing on individual diseases and new technologies towards addressing systemic challenges that limit access to veterinary services and inputs. Few research studies focused on incidence/ prevalence of disease *and* impact, suggesting better incorporation of socio-economic impact measures in future research would better represent the interests of livestock keepers.

## Highlights

• Literature review constraints included FMD, parasites, brucellosis, and PPR.
• Expert workshops added CCPP, CBPP, mastitis, and reproductive disorders.
• Most constraints manageable with existing technologies / best husbandry practices.
• Systemic challenges limit livestock keepers’ access to vet services and inputs.

## 1 Introduction

Healthy livestock support a growing human population, contribute to economic growth, and for the world’s poor, can offer a pathway out of poverty. Disease is a threat to these benefits, and humans have been concerned with the control of animal disease for thousands of years. A collection of papyri from the ancient Egyptian town of Kahun dating back to 2nd millennium BC containing detailed descriptions of cattle diseases and their treatment showcases our long history managing animal disease (Steverding, 2008). In modern times, motivations to control animal disease range from public health concerns in the case of zoonoses (Sherikar and Waskar, 2005; Kahn, 2006; Katz et al., 2009), economic gain (Singh et al., 2015; Webb et al., 2018; Barratt et al., 2019), and improvement of livelihoods (Peacock, 2005; Randolph et al., 2007; Kryger et al., 2010), especially for people around the world who depend on livestock. Controlling disease is a worthy but costly investment. Motivations for control often dictate how research and control efforts are prioritized.

### 1.1 Prioritization frameworks

This research contributes to an ongoing conversation about prioritization of animal health needs to maximize the impact of livestock on alleviating poverty. A seminal study described by Perry et al. (2002) was the first large-scale attempt to assess disease impacts with this goal in mind (Perry et al., 2002). The study considered all livestock species, all types of diseases (endemic, epidemic, zoonotic, and food-borne), and three major geographies where major concentrations of poverty exist: South-east Asia, South Asia, and Sub-Saharan Africa. The result was a ranking of priority diseases based on literature review and semi-quantitative input from groups of experts working in veterinary services, NGOs, and international organizations. Disease categorizations were overlaid on databases of livestock production systems and poverty.

Heffernan (2009) contributed a data-driven livestock disease prioritization framework that broadened the data inputs beyond a group of experts. The framework incorporates standard poverty measures, rankings of the importance of respective livestock species, income data, and mortality and morbidity rates of disease to yield a ‘disease gap’ or change in the poverty status of a household due to the impact of influence of a livestock disease. The study found large variations in the impact of the same disease across the wealth groups and within the same production system. East Coast fever had the greatest impact on poverty amongst pastoralists and smallholder farmers, while foot and mouth disease was more problematic for the better-off pastoralists (Heffernan, 2009). The framework was informed by data from livestock keeping households in Kenya. Data availability is a constraint to expanding this framework to new geographies. The Global Burden of Animal Diseases (GBAD) program, launched in 2018, is addressing this gap through the creation of a dataset used to estimate direct and indirect losses caused by animal diseases worldwide (Rushton et al., 2018). While the GBAD program will involve some data collection, it relies on exploiting existing public and private datasets.

This study contributes to the animal health needs prioritization conversation by identifying the most important animal health constraints affecting the livelihoods of commercially oriented smallholder farmers and pastoralists using three methods. By assessing the incidence/ prevalence and impact of smallholder animal health constraints using multiple methods across three geographic regions, we identify priorities and compare methods of prioritization.

## 2 Methods

The study contains three activities: a) a systematic literature review, b) a series of expert workshops, and c) a practitioner survey of veterinarians and para-veterinary professionals (Table 1). The geographic focus of the study was South Asia, West Africa, and East Africa. This study was designed as an initiative within the Global Alliance for Livestock Veterinary Medicines (GALVmed) 2030 strategy development process. GALVmed is a not-for-profit company operating as a livestock health product development and access partnership and a UK registered charity (“GALVmed”, 2020).

**Table 1:** Summary of study methods with associated geographies, livestock categories, and production systems.

Method	Geography	Livestock categories	Production systems
1. Systematic literature review	**South Asia:** India, Nepal, *Bangladesh (only if paired with India/ Nepal)* **West Africa:** Senegal, Mali, Ghana, Burkina Faso, Ivory Coast, Togo, Benin, Nigeria **East Africa:** Tanzania, Kenya, Uganda, Ethiopia, South Sudan, Malawi, Mozambique, Zambia	**Bovids:** Cattle, dairy cows, buffalo, yaks **Small ruminants:** Sheep, goats **Poultry:** Chickens, ducks, guinea fowl	Smallholders, agro-pastoralists, pastoralists, small-scale commercial

2. Expert workshops	**South Asia:** Held in India, participants from India and Nepal **West Africa:** Held in Ghana, participants from Chad, Mali, Côte d’Ivoire, Nigeria, Ghana, Sierra Leone **East Africa:** Held in Ethiopia, participants from Kenya, Tanzania, Ethiopia, Uganda	Poultry, small ruminants, cattle, dairy (including buffalo)	Smallholder and pastoralists

3. Veterinary practitioner survey	**East Africa:** Practitioners from East Africa, Uganda best represented	Emergent commercial poultry farming Low input small ruminant farming Smallholder large ruminant (non-dairy) Small scale dairy farming Pastoralist large ruminant farming Pastoralist small ruminant farming	

### 2.1 Systematic literature review

**Fig. 1:**
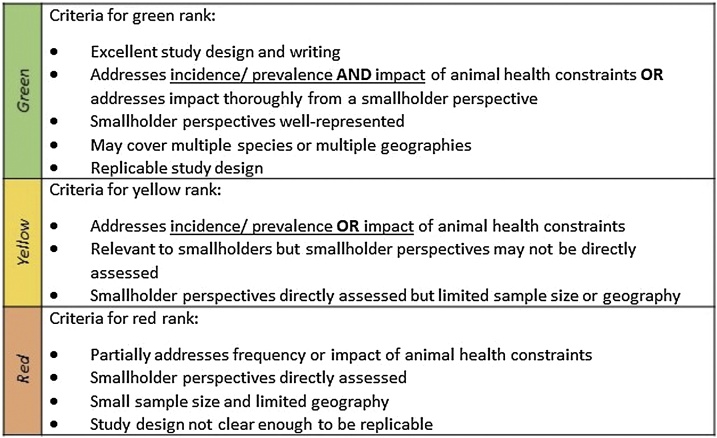
**Ranking criteria** used for the subset of articles focusing on impact of animal health constraints on smallholders and pastoralists.

A systematic literature review was conducted to understand the current state of published knowledge regarding smallholder animal health needs and the impact of diseases and syndromes (Fig. 1). We conducted searches in PubMed, CAB Abstracts, and Web of Science databases (Supplementary material 1). Document eligibility criteria included relevant location and livestock species, primary or secondary data, written in English, and published between 2002 and 2019. Each article was tagged with animal health constraint, location, and livestock category tags (Supplementary material 2 and 3). We were unable to consistently determine whether studies represented smallholder farmers, pastoralists, and/ or both so we did not disaggregate. The animal health tags were standardized based on health constraints that came up in the literature. All articles were given preliminary tags using keyword searches of the title and abstract. After the eligibility screening, a subset of articles with a focus on impact were selected and systematically tagged after reading the full text. These articles were purposively chosen for their consideration of incidence/ prevalence, impact, and smallholder perspective.

Summarized articles were given a ranking (green, yellow, red) to indicate the extent of the article’s focus on incidence/ prevalence of animal health constraints and their impact (Fig. 1). Green-ranked articles are best, focusing on incidence/ prevalence and impact. Four researchers trained on a shared set of about 20 articles, discussed conflicts, then subsequent articles were ranked by a single researcher. A team of two researchers worked together on each geographic area with one member of each team professionally based in or responsible for the relevant geographic area. Incidence/ prevalence included disease seroprevalence, outbreak incidence, number of cases, or percentage of self- reported disease. Impact included mortality, morbidity, production losses, economic losses, monetary quantification of disease burden, or qualitative descriptions of impact by smallholder livestock keepers. The principal summary measure used in the results was whether a given animal health constraint was mentioned in the article regardless of the type of study conducted. We did not attempt to assess bias of individual studies that may have affected their study outcome although the quality of the impact articles is reflected in the ranking criteria.

### 2.2 Expert workshops

Three workshops were held with sector experts in Ethiopia, Ghana, and India in 2019.

Experts were selected based on having extensive, first-hand knowledge of smallholder livestock production systems in relevant settings within the specific regions of interest (East Africa, West Africa, India & Nepal) and operational knowledge of veterinary provision to smallholder livestock keepers. The objective was to identify big picture priorities from the perspective of a commercially oriented smallholder livestock keeper across a large geographic area. By default, such an approach fails to capture finer scale variation and doesn’t include first-hand testimonials from farmers, farmer groups, or value chain actors who interact with farmers. The workshop relied on experts to synthesize their experiences working with livestock keepers into succinct identification of constraints that are important on a large scale.

Workshop sessions were structured to achieve a consensus of animal health constraint ranking while attempting to address inherent biases brought by the experts (e.g. resulting from their research focus or those diseases prioritized by government veterinary services) and in the literature (e.g. focus on specific diseases without examining the broader impact to the smallholder). To assist the experts in developing independent opinions without being biased by the group during the workshop, each participant was asked to rank the top three animal health priorities before the meeting, using a standard form. Attention was drawn to the “animal health constraint” so the experts could prioritize syndromes and not be restricted to individual diseases. The literature review was shared with the attendees, after they had completed their survey, but before travelling to the workshop. The workshop itinerary is presented in Supplementary material 4.

### 2.3 Veterinary practitioner survey

An animal health priority questionnaire (Supplementary material 5) was administered to veterinarians and para-veterinary professionals working in East Africa. They were asked to rank animal health constraints in a series of categories of species/ production systems (Table 1). The survey administration was managed by two GALVmed regional veterinary consultants on contract for a larger survey project. The consultants reached out to the veterinary practitioners and requested they complete the 30- minute survey using an online link or by printing and scanning a paper form. A payment of $10 USD was set as an incentive to return the survey within one week. The target was to request a completed survey from 20 practitioners from each of the 3 countries (Kenya, Tanzania, and Uganda) for a total of 60 people contacted.

## 3 Results

### 3.1 Systematic literature review

**Fig. 2:**
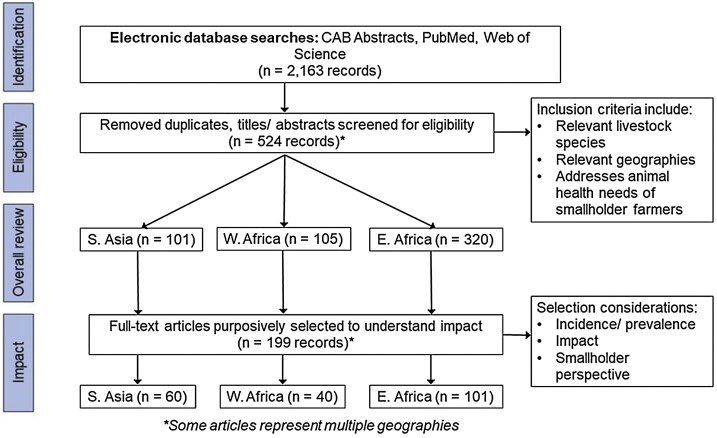
**PRISMA flow diagram** showing the selection of articles for the overall literature review and more targeted impact study.

The literature search resulted in 524 eligible documents for the overall literature review and a subset of 199 documents that focused on impact (see Fig. 2 and Supplementary materials 3, 6–8). Of the 199 impact articles, only 17 articles across all geographies met the criteria for the “best” green rank, “addressing incidence/ prevalence AND impact of animal health constraints OR addressed impact thoroughly from a smallholder perspective”.

**Fig. 3:**
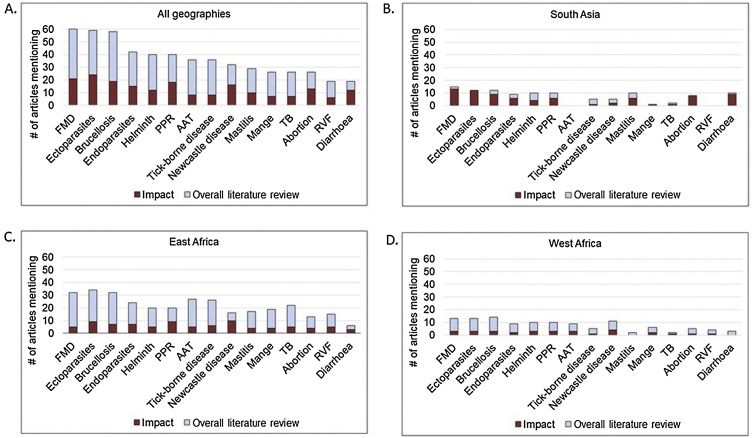
**Top 15 animal health constraints** by geographic area ordered by number of articles mentioning the concern. (n = 524 articles) The height of the bar represents the total number of articles, the upper, light blue bar represents the number of articles in the overall literature review, and the lower, dark red bar represents the subset of articles focusing on impact. **Abbreviations:** Foot and mouth disease (FMD), peste des petits ruminants (PPR), animal African trypanosomiasis (AAT), tuberculosis (TB), Rift Valley Fever (RVF), lumpy skin disease (LSD). **Note:** Some constraints do not apply to all geographic areas, such as Rift Valley Fever in South Asia. Some articles mention multiple health constraints.

The top 15 animal health constraints for all geographies in order of number of overall literature review articles that mentioned them were foot and mouth disease (FMD), ectoparasites, brucellosis, endoparasites, helminths, peste des petits ruminants (PPR), animal African trypanosomiasis (AAT), tick-borne diseases, Newcastle disease (ND), mastitis, mange, tuberculosis, abortion, Rift Valley Fever (RVF), and diarrhea. The number of documents mentioning each health concern ranged from 60 for FMD to 19 for diarrhea (Fig. 3). A full list of all health constraints and annotated bibliographies by region and livestock type are presented in Supplementary material 6. Five green-ranked articles mentioned FMD and trypanosomiasis respectively (three articles discussing animal African trypanosomiasis plus two discussing trypanosomiasis in South Asia) and two mentioned lumpy skin disease (LSD) (Supplementary material 8). Health constraints mentioned by a single, green-ranked article included ectoparasites, abortion, East Coast fever, Newcastle disease, PPR, and black quarter.

When all impact articles are broken down by geography and production system, as shown in Table 2, important health concerns include FMD and brucellosis for bovids (which includes cattle and dairy), PPR for small ruminants, and Newcastle disease for poultry with the addition of avian influenza in West Africa and South Asia. Broad categories including endoparasites, exoparasites, and helminths are top concerns in all geographies, especially for bovids and small ruminants.

**Table 2:** **Animal health constraints:** Findings from overall literature review documents including subset of impact articles. Health constraints mentioned by only one article not listed.

Overall review	Region	n	1.	2.	3.	4.	5.
Poultry	East Africa	29	ND (16)	IBD (10)	*Fowl pox (4)	*Coccidiosis (4)	*Endo/exoparasites (4)
	West Africa	24	Avian influenza (9)	ND (8)	IBD (4)		
	South Asia	16	ND (5)	Avian influenza (3)	Coccidiosis (2)	Salmonellosis (2)	
Small ruminants	East Africa	100	PPR (20)	Ectoparasites (19)	Mange (16)	Helminths/ endoparasites (12)	RVF (12)
	West Africa	34	Ectoparasites (8)	Helminths (7)	Endoparasites (6)	Brucellosis (6)	
	South Asia	35	PPR (9)	Ectoparasites (8)	Helminths (7)	Endoparasites (6*)	Brucellosis (6*)
Cattle/dairy	East Africa	240	FMD (31)	Brucellosis (29)	AAT (27)	Ectoparasites (25)	TBDs (22)
	West Africa	67	FMD (13)	Brucellosis (12)	AAT (9*)	Ectoparasites (9*)	Helminths (9*)
*(w. buffalo)*	South Asia	61	Ectoparasites (12*)	FMD (12*)	Brucellosis (11)	Mastitis (10)	Theileriosis (8)
Impact	Region		1.	2.	3.	4.	5.
Poultry	East Africa	12	ND (10)	IBD (3)	Fowl pox (4)	Endo/exoparasites (3*)	Coccidiosis (3*)
	West Africa	10	Avian influenza (5)	ND (4)	CRD (2)		
	South Asia	9	Avian influenza (3)	ND (2)	Coccidiosis (2)		
Small ruminants	East Africa	35	PPR (10)	Ectoparasites (9)	Mange (9)	Endoparasites (6)	
	West Africa	24	Helminths (4)	Ectoparasites (3)	Endoparasites (3)	Haemonchosis (3)	
	South Asia	24	Helminths (4)	PPR (3*)	Endoparasites (3*)	Ectoparasites (3*)	Haemonchosis (3*)
Cattle/dairy	East Africa	67	Brucellosis (9)	FMD (8*)	AAT (8*)	Ectoparasites (8*)	TBDs/ LSD (8*)
	West Africa	25	Brucellosis (5*)	Helminths (5*)	FMD (4*)	Endoparasites (4*)	
*(w. buffalo)*	South Asia	37	FMD (10)	Ectoparasites (8*)	Brucellosis (8*)	Diarrhea (7)	

**Abbreviations:** Newcastle disease (ND), Infectious bursal disease (IBD), peste des petits ruminants (PPR), Rift Valley Fever (RVF), African trypanosomiasis (AAT), tick-borne diseases (TBDs), lumpy skin disease (LSD). **Notes:** TBDs include heartwater, ECF, anaplasmosis, babesiosis. Helminths is a subset of the endoparasite category.

* Indicates a tie.

### 3.2 Expert workshops

A total of 23 participants attended the three expert workshops (12 in South Asia, 13 in West Africa, and 8 in East Africa). The experts included academic researchers, government veterinarians, and representatives from private sector companies and NGOs delivering veterinary products and services to smallholder livestock keepers. Only three participants were women and there were no women participants in the South Asia workshop. The workshops, unlike the systematic literature review, differentiated between animal health constraints for smallholder farmers and pastoralists and divided dairy and cattle into two separate categories.

Top concerns included FMD, PPR, Newcastle disease, avian influenza, consistent with the top results of the systematic literature review, with the addition of Contagious Caprine Pleuropneumonia (CCPP), Contagious Bovine Pleuropneumonia (CBPP), mastitis, and reproductive disorders (Table 3). The workshop results contained additional health constraints not mentioned in the top 15 animal health constraints of the systematic literature review, including dermatophilosis, Gumboro disease (infectious bursal disease), sheep and goat pox, respiratory problems, abortion, coccidiosis, lameness, orf, and foot rot.

**Table 3:** **Animal health constraints:** Consensus from East Africa, West Africa, and South Asia expert workshops.

Smallholders	Region	1.	2.	3.	4.	5.	6.
Poultry	East Africa	ND	Nutrition	Diarrhea	Gumboro	Fowl typhoid	Fowl pox
	West Africa	ND	Gumboro	Diarrhea	Avian influenza	Fowl pox	
	South Asia	Avian Influenza	ND	Fowl pox	Coccidiosis	Ecto/Endoparasites	Gumboro
Small ruminants	East Africa	CCPP	PPR	SGP	Helminths	Abortion	Foot rot
	West Africa	PPR	Helminths	Mange	Abortion	Heartwater	Lameness
	South Asia	PPR	Endoparasites/ diarrhea	Respiratory problems	Vesicular disease	Zoonoses	Abortion
Cattle	East Africa	FMD	TBDs	LSD	Helminths	AAT	
	West Africa	CBPP	Dermatophilosis	Abortion	Ecto/Endo parasites	Lameness	
Dairy	East Africa	Mastitis	Abortion	Brucellosis	FMD	LSD	TBDs
	West Africa	Masititis	Abortion	FMD	Helminths	Dermatophilosis	
*(w/ buffalo)*	South Asia	Reproductive disorders	FMD	Mastitis	Ecto/Endo/ Haemo parasites	Metabolic diseases	Zoonoses
Pastoralists							
Small ruminants	East Africa	CCPP	Helminths	PPR	SGP	Orf	Abortion
	West Africa	PPR	Helminths	Abortion	Mange	Heartwater	
Cattle	East Africa	FMD	TBDs	AAT	LSD	Helminths	Anthrax
	West Africa	CBPP	Dermatophilosis	FMD	Bovine TB	Abortion	Ecto/ Endoparasites

**Abbreviations:** Newcastle disease (ND), contagious caprine pleuropneumonia (CCPP), peste des petits ruminants (PPR), sheep and goat pox (SGP), foot and mouth disease (FMD), tick-borne diseases (TBDs), lumpy skin disease (LSD), animal African trypanosomiasis (AAT), contagious bovine pleuropneumonia (CBPP), bovine tuberculosis (bovine TB). **Note:**Haemo parasites include theileria, trypanosomes, and babesia; metabolic diseases include milk fever and ketosis; vesicular diseases include Orf, contagious ecthyma, FMD, SGP; zoonoses include Johne’s disease and brucellosis; reproductive disorders include symptoms resulting from brucellosis, Infectious bovine rhinotracheitis (IBR), bovine viral diarrhea (BVD), poor diet and infertility from unknown causes.

The increasing endemicity of avian influenza in West Africa and India was highlighted as a threat to smallholder poultry production, with broader veterinary public health implications. FMD was recognized as a constraint to large ruminant production in all three workshops. The availability of FMD vaccines, either through public immunization campaigns or private sector delivery, was identified as a major factor in controlling disease for smallholder livestock keepers.

The constraints in Table 3 can be grouped into three classes: vaccine preventable (registered vaccines available in the region/country), treatable with pharmaceuticals (registered pharmaceuticals available within the region/country), and largely avoidable through best practice in husbandry (recognized methods in production which if followed will reduce the risk of certain conditions). The experts were challenged to reflect whether a smallholder livestock keeper with the knowledge and financial resources could address these constraints within their country, given the existing private and public sector set-ups as they currently stand. For most of the constraints, the answer was “yes”.

One exception to the constraints acknowledged to be avoidable through vaccination, treatment, or good husbandry, was reproductive disorders. In all workshops, it was recognized there was a poor understanding of the etiology underlying these disorders, particularly for abortions and neo-natal mortality. While *Brucella* species were commonly implicated, the evidence base was often poor, with those few studies conducting more rigorous diagnostic investigations suggesting a much more complicated and unclear picture as to the main reasons for abortions and neo-natal mortality.

### 3.3 Workshop discussion

In addition to the prioritization of animal health constraints, workshop participants brought up additional, cross-cutting concerns including improved vaccines, regulatory reform, product quality and correct application, and emerging trends.

#### 3.3.1 Improved vaccines

A common theme from across all three workshops was the desire for combination vaccines. The idea was discussed particularly with reference to the global programme for the control and eradication of PPR, which will provide an important platform for controlling other diseases including CCPP and SGP. The profile of a combination eye-drop vaccine for poultry disease was also supported. Reduced vial size and simplification of administration (e.g. pre- diluted) were also common themes, widely recognized as helping to improve uptake by smallholder livestock keepers.

#### 3.3.2 Regulatory reform

Reforms in specific legislation were identified as important in addressing several of the animal health constraints. In Kenya, the mismatch between the desire for smallholder livestock keepers to vaccinate against FMD privately, and the regulated role of the state in providing all FMD vaccinations (without enough resources to meet demand) was one such example. Across both Africa meetings, the role of community animal health workers was discussed. The need for improved standards for artificial insemination operatives, and the ability for them to increase income from the provision of other services, such as vaccination, was highlighted. Similar concerns were expressed for the standardization of para-veterinarians.

#### 3.3.3 Product quality and correct application

Quality issues with vaccines, pharmaceuticals and supplements/feed were highlighted as a contributory factor for many of the constraints listed in Table 3. It was recognized that private animal health product and service providers should be able to address these issues through well managed supply chains. Developing a recognizable and enforceable mark for veterinary inputs, such as the Pan African Veterinary Centre of the African Union (PANVAC) stamp on vaccines, was proposed as a way for smallholder livestock keepers to assess quality. With intensification of smallholder livestock production, issues of feed quality and the need for supplementation of incomplete diets is increasingly necessary to capitalize on market opportunities.

Outside of the quality of product, correct application was identified as another important contributory factor to the persistence of avoidable constraints. Well-recognized examples included under-application of acaricides and underdosing with antimicrobials leading to increased problems of resistance. Linked to this, on the off-take side, poor practices drive antimicrobial and acaricide/insecticide contamination of eggs, milk, meat, and hides.

The growth of both antimicrobial drug resistance and acaricide resistance were addressed in all three workshops. It was recognized that lack of knowledge on correct administration, as well as poor quality or even counterfeit products, were major contributors to the trend.

#### 3.3.4 Emerging trends

Participants in all three workshops brought up two additional emerging trends: the growing importance of goats and climate change.

Although the drivers were different (e.g., export trade to the Middle East in East Africa, land pressures and migration pattern change in West Africa, rise of popularity of goat milk in India), goats were seen as an increasingly important source of income for smallholder livestock keepers. Traditionally, goats have been perceived as a livestock option that requires minimal investment, with smallholders not investing much in animal health inputs. The PPR vaccination programs, coupled with increased market demand for goat meat and milk, provide an opportunity to alter these perceptions.

Increased climatic variability and increased frequency of extreme weather events were considered important trends in all three settings. This is contributing to changes in land use which greater pressure on water and grazing land; this in turns drives driving intensification and changes in production systems, such as the switch from cattle to goats.

### 3.4 Veterinary practitioner survey

The results of the veterinary practitioner survey are shown in Table 4. Constraints match those mentioned in the workshop, with the addition of chronic respiratory disease for poultry, anaplasmosis in smallholder dairy operations, and the inclusion of nutrition as a constraint, mentioned in relation to smallholder dairy and pastoralist small ruminants. Out of 60 practitioners contacted, only 20 returned surveys (response rate of 33 %). The survey was sent soon after another extensive survey done by GALVmed which likely led to contact and practitioner fatigue and contributed to the low response rate. Most practitioners were from Uganda (13), followed by Tanzania and Kenya (3 each), and one from Ethiopia. The practitioners had an average of 15 years of field experience. The gender of the practitioners was not recorded.

**Table 4:** **Animal health constraints:** Consensus from veterinary practitioner survey conducted in East Africa.

Smallholders	1.	2.	3.	4.	5.
Poultry	ND	Coccidiosis	Ecto/Endo parasites	Fowl pox	Chronic respiratory disease
Small ruminants	Helminths	TBDs	CCPP	PPR	Orf
Cattle	FMD	TBDs	LSD	Helminths	Trypanosomiasis
Dairy	Mastitis	Infertility & abortion	Nutrition	ECF	Anaplasmosis
Pastoralists					
Small ruminants	CCPP	PPR	Endoparasites	Nutrition	Orf & pasteurellosis
Cattle	FMD	CBPP	ECF	TBDs	Endoparasites

**Abbreviations:** Newcastle disease (ND), tick-borne diseases (TBDs), contagious caprine pleuropneumonia (CCPP), peste des petits ruminants (PPR), foot and mouth disease (FMD), lumpy skin disease (LSD), East Coast fever (ECF), contagious bovine pleuropneumonia (CBPP).

## 4 Discussion

The objective of this study was to assess the incidence/ prevalence and impact of smallholder animal health needs using multiple methods across three geographic regions, identify priorities, and compare methods of prioritization.

### 4.1 Incidence, prevalence, and impact

Less than 10 % (17/199) of the impact articles met the criteria for green ranking, evidence that few studies were able to address impact thoroughly from a smallholder perspective or incorporate measures of incidence/ prevalence and impact of animal health constraints. In this section, we briefly discuss the types of articles found in the literature review, highlight two excellent, green-ranked studies, and identify opportunities for better incorporating impact measures in future research.

Many articles were characterizations of production systems with limited sample sizes in a single geographic area (Kamal et al., 2012; Seyoum et al., 2013; Olobatoke et al., 2015; Satisha et al., 2018). Others addressed incidence/ prevalence with a larger sample size in a broader geographic area (for example serosurveys for a specific disease) but failed to connect incidence or prevalence estimates to the impact on smallholder livestock keepers (Jagun and Onoja, 2011; Kabi et al., 2015; Abdela, 2017; Chengat Prakashbabu et al., 2017; Okumu et al., 2019). A few articles estimated economic impact of specific diseases or outbreak events at household level (Sitawa et al., 2016), farm level (Govindaraj et al., 2018), or herd level (Alhaji and Isola, 2018).

Examples of green-ranking articles include Jemberu et al. (2014) with an outbreak investigation of FMD in Ethiopia which reported on morbidity/ mortality rates and economic losses due to milk loss, draft power loss, and mortality (Jemberu et al., 2014). The study also disaggregated data based on production system, differentiating between pastoralists and crop-livestock mixed system. A second example of a green ranking article is a study by Kumar et al. (2017), who conducted retrospective risk analyses to estimate economic losses in India due to trypanosomiasis. In addition to reporting an estimated total annual loss of US $ 671.1 million (US $ 344–US $ 1209 million at 95 % confidence interval), they included loss parameters relevant to smallholder livestock keepers including reduction in milk yield (36 % of total loss), reproductive losses (26 %), reduction in growth (10 %), reduction in draught power (8 %), and additional opportunity cost (3 %) (Kumar et al., 2017).

Opportunities for contributing to the current body of literature for smallholder animal health needs include studies from interdisciplinary teams that connect results from epidemiology studies with socio-economic impact assessments identify priorities and compare methods of prioritization and consideration and inclusion of impact indicators that are relevant for a smallholder context. All three prioritization methods identified constraints such as endo and ectoparasites, mange, mastitis, reproductive disorders, and even nutrition in the veterinary practitioner survey, that contribute to morbidity rather than mortality. Impact on smallholder livestock keepers may come in the form of production losses, less profitable sales, increased veterinary costs, or increased labor or time required to care for animals. Thoughtful identification of relevant indicator measurements of impact and measurement of these indicators will allow for more useful impact estimates. Interdisciplinary teams that include economists and social scientists are more likely to have a broader vocabulary and include a wider range of impact indicators that studies undertaken by veterinarians or epidemiologists only. The conspicuous absence of gender as a consideration in this and other studies is evidence of the limited capacity of many animal health research teams. The role of women in agriculture and livestock production is well-documented (FAO, 2010) and the different roles played by men and women in livestock keeping means they may be differentially impacted by disease (Kristjanson et al., 2010), prioritize livestock species differently (Wieland et al., 2016), and face different challenges in accessing information and veterinary services such as vaccines (Mutua et al., 2019). While this study does not provide a metric for the number of articles that incorporated a gender perspective, it is clearly an area for improvement in future research projects. Lastly, stratifying disease priorities by production system is useful in interpreting animal health priorities, however not all studies in the systematic literature review were specific enough to distinguish between pastoralists and smallholder farmers or even identify the level of intensification. Clearly indicating this type of information makes studies more useful for prioritizing smallholder animal health constraints.

### 4.2 Identifying priorities

Globally, ten constraints emerge from all three methods as having high impact on smallholder livestock keepers: endo/ ectoparasites, FMD, brucellosis, PPR, Newcastle disease, avian influenza, CCPP, CBPP, mastitis, and reproductive disorders. This list is in no way exhaustive and it may be more useful to look at the full list of disease constraints identified by region or production system.

As noted by the workshop participants, many of these constraints, except for reproductive disorders, can be prevented or treated using best practices with existing technologies available in the respective geographies. This supports the prioritization of addressing systemic limitations that make it challenging for smallholder livestock keepers to access veterinary services and inputs. The conclusions about regulatory reform and product quality/ correct application raised by workshop participants are examples of such challenges. Improved vaccines, especially combination vaccines that prevent multiple diseases, may be a valuable contribution for populations with limited access to veterinary services. Supporting infrastructural improvements to veterinary systems through support of paraprofessionals or strengthening public private partnerships may also be a valuable entry-point for addressing animal health constraints. Prioritizing the systems and infrastructure that delivery existing veterinary technologies is another direct invitation to incorporate gender analyses into future studies. While women livestock keepers may face more barriers to access, they can be powerful allies in disease control when given access to the appropriate tools, as evidenced by a study in Kenya linking the formation of women’s groups with improved control of transboundary livestock diseases (Kristjanson et al., 2010).

### 4.3 Comparing methods

Lastly, we compare methods of prioritization within the study and across similar studies. While there is some consensus across the three methods of prioritizing animal health constraints within this study, the following constraints were not in the top 15 identified in the literature review overall or for any geographic region but were identified as important in the workshops and/ or practitioner survey: CBPP, CCPP, heartwater, and lumpy skin disease.

Published literature focused more on individual diseases rather than syndromes, such as reproductive disorders (although abortion was a top constraint), or production constraints, such as nutrition. Funding and research priorities are not necessarily driven by improving the welfare of smallholder livestock keepers and using published studies to inform priorities may lead to bias towards zoonoses such as zoonotic tuberculosis, biologically interesting diseases such as trypanosomiasis, or well-recognized diseases such as FMD at the expensive of chronic and low-mortality constraints such as mange or syndromes such as reproductive disorders. Workshop participants may also introduce biases stemming from their personal research interests. Veterinary practitioners may underestimate the danger of outbreaks of rare diseases or constraints that have economic and public health consequences beyond the impact on the clients they work with.

Conclusions of this study shared by Perry et al. (2002) and Heffernan (2009) , two other studies prioritizing animal health concerns as a function of their impact on the poor, are the need for improved impact measures and the promise of using existing technologies and best practices in conjunction with improving the delivery of animal health services. A key finding from Heffernan’s study was that for diseases with a high morbidity but comparatively low mortality, poor households spent much less on treatment and prevention than the better off, and are therefore disproportionately affected (Heffernan, 2009). The delivery of animal health services and the need to make existing technologies “more effective and appropriate for the poor” was addressed by Perry et al. (2002). Debates about how best to serve farmers in smaller scale production systems continue, as evidenced by the theme of the 2018 Tanzania Veterinary Association conference “Veterinary Profession as a Catalyst for Transformative Change of the Animal Industry”, where veterinary practitioners discussed the changing landscape of veterinary governance and extension service delivery system (Tanzania Veterinary Association, 2018).

While some overarching challenges remain constant, the systematic literature review revealed some notable progress managing animal health constraints since publication of the first systematic prioritization study by Perry et al in 2002. Examples include the global eradication of rinderpest (Roeder and Rich, 2009), a disease that ranked within the top 20 conditions with impact on the poor, and commitment from the international community to begin working towards the eradication of peste des petits ruminants (OIE/FAO, 2015). There have also been beneficial technological advances, such as the development of a thermotolerant Newcastle disease vaccine (I-2), which is now produced and available in many developing countries (Fisher, 2014).

### 4.4 Limitations

Limitations to this study include lack of representation of grey literature published outside of peer-reviewed journals, potential exclusion of relevant articles from Francophone countries in West Africa by limiting the search to articles published in English, having only one reviewer assigning impact score for each article in the systematic literature review, the selection of a small number of experts for the workshops, lack of representation of livestock keepers themselves in the workshop, and the limited geography of the veterinary practitioner survey, which ideally would have also been administered in West Africa and South Asia. Despite literature documenting the gender gap in agriculture and the role of women in livestock production, the under-representation of women in the stakeholder workshops (only 3 of 23 experts were women) perpetuates the status quo where the priorities and interests of men dominate discussions about how resources should be prioritized. Pigs were not included in this study due to the organizational prioritizations of GALVmed and its funders, however they play an important role in smallholder livelihoods globally. Nutrition and feeding strategies and antimicrobial resistance were excluded in the systematic literature review, but nutrition was identified as an important constraint in the veterinary practitioner survey and antimicrobial resistance was brought up in the expert workshops. There were few representatives of broader animal management constraints such as nutritionists or welfare specialists in the expert workshops, which likely contributed to their underrepresentation in the findings. Lastly, there were some small differences in the disease constraint definitions across the three methods because a categorization system was independently created for each method. For example, the systematic literature review included ectoparasites and endoparasites separately while the stakeholder workshop combined them. This allowed the disease constraints to more closely match the priorities that emerged from each method, however, introduces minor challenges when comparing results across methods.

### 4.5 Conclusion

Managing animal health constraints is a pathway to improving the livelihoods of smallholder and pastoralist livestock keepers globally. By employing three methods of prioritization, we identify diseases and syndromes that have the greatest impact on livestock keepers. Most of the top priorities can be managed using existing technologies and best practices which supports a shift away from focusing on individual diseases and new technologies and towards addressing systemic challenges that limit access to veterinary services and inputs. Few research studies focused on incidence/ prevalence of disease and impact on smallholders suggesting opportunities for interdisciplinary studies that include gender analyses and define impact using socioeconomic indicators in addition to epidemiology indicators.

## Declaration of Competing Interest

Paul Coleman, Andrea Guest, Brian Perry, and Zoë Campbell contributed as consultants for GALVmed to complete these research activities.
