# Supplementary material for: Prioritizing smallholder animal health needs in East Africa, West Africa, and South Asia using three approaches: Literature review, expert workshops, and practitioner surveys
Source: Prev Vet Med. 2021 Apr;189:105279. doi: 10.1016/j.prevetmed.2021.105279 (PMC8024747; doi:10.1016/j.prevetmed.2021.105279)
Supplement: Supplementary file 1 [file mmc1.docx]

# Supplementary material 1: Search criteria for systematic literature review

**LOCATION**

**South Asia:** India, Nepal, *Bangladesh (only if paired with India/ Nepal)*

**West Africa:** Senegal, Mali, Ghana, Burkina Faso, Ivory Coast, Togo, Benin, Nigeria

**East Africa:** Tanzania, Kenya, Uganda, Ethiopia, South Sudan, Malawi, Mozambique, Zambia, *(Zimbabwe if relevant)*

**LIVESTOCK SPECIES**

**Dairy/ beef:** Cattle, buffalo, yaks

**Poultry:** Chickens, sucks, guinea fowl

**Small ruminants:** Goats, sheep

**TIME FRAME:** Based on publication date of citation.

2002 - 2019

**DOCUMENT INCLUSION CRITERIA**

- Relevant location and livestock species
- Primary or secondary data, reviews only if very relevant and well-written.
- Written in English
- Addresses animal health needs of smallholder farmers

*Exclude following topics:*

- Breeding strategies
- Nutrition/ feeding strategies
- Antimicrobial resistance
- Production constraints (diseases not specified)
- Characterizations of production systems (diseases not specified)
- Ethno-veterinary practices (diseases not specified)
- Reports of zoonotic diseases focusing only on human health impact
- Genotyping or other basic research on animal health diseases / vectors if smallholder impact not specified
- Vaccine trials
- Human serosurveys of zoonoses (unless accompanied by animal serosurveys)

*Include following topics:*

- Participatory epidemiology
- Ethno-veterinary practices (diseases specified)
- Aflatoxins

**SOURCES**

1. CAB Abstract
2. PubMed
3. Web of Science

Example of Pubmed broad search for South Asia: ((((((India) OR Nepal)) AND ((((animal husbandry) AND disease) NOT Drug Resistance, Microbial) AND ("2002/01/01"[PDat] : "3000/12/31"[PDat]) AND veterinary[sb])) AND ((((((cattle) OR chickens) OR sheep) OR goats) OR buffaloes) AND ("2002/01/01"[PDat] : "3000/12/31"[PDat]) AND veterinary[sb])) NOT (pigs OR china)) AND ("2002/01/01"[PDat] : "3000/12/31"[PDat]) AND veterinary[sb]

# Supplementary material 2: Systematic literature review tags

Excel document.

# Supplementary material 3: Article database

Separate file

# Supplementary material 4: Stakeholder workshop itinerary

Day 1

| **Start** | **End** | **Day 1** |
| --- | --- | --- |
| 9:00 AM | 9:30 AM | Welcome & Introductions |
| 9:30 AM | 10:00 AM | Purpose, Structure, Objectives |
| 10:00 AM | 11:00 AM | Session 1 (Poultry) Outcome: priority list defined |
| 11:00 AM | 11:15 AM | Break/Refreshments |
| 11:15 A | 12:15 PM | Session 2 (Small Ruminant – Small Holder & Pastoralist [where applicable] |
| 12:15 PM | 12:30 PM | Break/Refreshments |
| 12:30 PM | 1:30 PM | Continued Session 2 (Small Ruminant – Small Holder & Pastoralist [where applicable]) Outcome: priority list defined |
| 1:30 PM | 2:45 PM | Lunch |
| 2:45 PM | 3:45 PM | Session 4 (Large Ruminant – Dairy, Cattle non-dairy, Pastoralist [where applicable]) |
| 3:45 PM | 4:00 PM | Break/Refreshments |
| 4:00 PM | 5:00 PM | Continued Session 4 (Large Ruminant – Dairy, Cattle non-dairy, Pastoralist [where applicable]) |
| 5:00 PM | 6:00 PM | Wrap Up |

**Day 2**

| **Start** | **End** | **Day 2** |
| --- | --- | --- |
| 9:00 AM | 9:15 AM | Welcome back, structure of day |
| 9:15 AM | 10:15 AM | Poultry: Review yesterday’s list, adjust.  Analysis of constraints and future trends |
| 10:15 AM | 10:30 AM | Break/Refreshments |
| 10:30 AM | 11:30 AM | Small Ruminants: Review yesterday’s list, adjust.  Analysis of constraints and future trends |
| 11:30 AM | 11:45 PM | Break/Refreshments |
| 11:45 AM | 12:45 PM | Cattle: Review yesterday’s list, adjust.  Analysis of constraints and future trends |
| 12:45 PM | 1:30 PM | Wrap Up |

**Session Structure & Details**

| **Items** | **Notes** |
| --- | --- |
| **Objective:** | To agree a priority list of animal health constraints impacting small holder livestock farmers |
| **Background:** | 1. Ahead of the meeting, the group of attending practitioners will be asked to rank the top (five) priorities as they see them in each stratum (questionnaire). This information will be analyzed for discussion at the workshop 2. Geographically focused literature review will be shared as a resource in advance and used during to inform discussions as well as compare and contrast against priority lists defined over the duration or the workshop. |
| **Day 1 Session Structure:** | 1. Introduction to each other 2. Introduction to the workshop goal and structure 3. Break-out into small groups and create an animal priority constraint list, present list, analyze each group’s list and create one consensus 4. The attendee’s initial rankings (pre workshop questionnaire) will be shared with the group to challenge consensus formed in step 3. New consensus agreed. 5. Compare list against the findings of the literature review. New consensus agreed. 6. Final consensus and major considerations agreed / discussion points summarized including:    1. How did we come to this conclusive list? What were the factors and how does it compare or contrast to the survey result and literature review?    2. How does the condition result in economic losses to the smallholder?   Note: In East Africa, a group of ‘on the ground’ practitioners were also asked (in advance of the workshop via an online survey) to list the priority animal health constraints. This was then used in the Addis workshop alongside the literature review and the attendee’s pre workshop lists to challenge the workshop consensus list |
| **Day 2 Session Structure:** | 1. Introduction 2. Analysis of results: 3. Why is the constraint not currently controlled? (technical, policy, market failure?) 4. What is required to address the constraint? 5. Other species for consideration? What trends should we be aware of? 6. Wrap Up |

# Supplementary material 5: Animal health priority questionnaire

Separate file

# Supplementary material 6: Annotated bibliography and systematic literature review results by geography and livestock type

Zip file containing 14 documents used for stakeholder meetings.

# Supplementary material 7: List of references broken down by geography and classification of article

Separate file

# Supplementary material 8: Table of animal health constraints broken down by different analyses in the literature review

Separate file
