# Supplementary material for: Prioritizing smallholder animal health needs in East Africa, West Africa, and South Asia using three approaches: Literature review, expert workshops, and practitioner surveys
Source: Prev Vet Med. 2021 Apr;189:105279. doi: 10.1016/j.prevetmed.2021.105279 (PMC8024747; doi:10.1016/j.prevetmed.2021.105279)
Supplement: Supplementary file 3 [file mmc3.pdf]

Smallholder Animal Health Needs Assessment

East Africa Cattle

GALVmed

August 5, 2019

Assembled by Zoë Campbell, Thembinkosi Ramuthivheli, Tom Osebe, and Paul Coleman

Meta-analysis summary

The literature review yielded 240 articles, 67 of which focused on impact, incidence, and prevalence, and are summarized below. The table below shows the number of articles in which each animal health concern appears in the literature about cattle in East Africa (many articles mention multiple concerns). The summarized articles are in the bibliography within this document. The full list of articles considered in the East Africa meta-analysis is available as a separate document.

The most mentioned concerns are foot and mouth disease, brucellosis, African Animal Trypanosomiasis (AAT), ectoparasites, tick-borne disease, tuberculosis, lumpy skin disease, and mastitis (mentioned in 15+ articles). Main topics of the green ranking articles that address impact and incidence/ prevalence include foot and mouth disease, AAT, and lumpy skin disease.

Animal health concern	Frequency of mention (meta-analysis)	Frequency of mention (impact articles)	Main topic of green rank article
Foot and mouth disease	31	8	3
Brucellosis	29	9	1
AAT	27	8	2
Ectoparasites	25	8	1
Tick-borne disease	22	8	0
Tuberculosis	21	4	0
Lumpy skin disease	19	7	2
Mastitis	16	6	0
Anaplasmosis	15	6	1
Mange	15	5	0
East Coast fever	15	5	1

Endoparasites	14	4	1
Helminth	13	3	0
Abortion	12	6	1
RVF	10	1	1
Blackquarter	10	2	1
CBPP	8	3	0
Anthrax	8	2	0
Theileriosis	7	4	0
Babesiosis	6	3	1
Pasteurellosis	6	1	1
Fascioliasis	5	1	0
Malignant catarrhal fever	5	1	0
Leptospirosis	5	2	0
Bovine viral diarrhoea virus	5	1	1
Heartwater	4	2	0
Dermatophilosis	4	1	0
Q fever	4	1	0
Cryptosporidiosis	4	0	0
Schistosomiasis	3	0	0
Diarrhoea	3	2	0
Acaricide resistance	3	1	0
Aflatoxin	3	0	0
Hydatidosis	2	1	0
Dystocia	2	0	0
Neospora caninum infection	2	2	1

Retention foetal membrane	2	0	0
Bluetongue	2	0	0
Reproductive disorder	2	0	0
Trypanocide resistance	2	1	0
Toxoplasmosis	1	0	0
Colibacillosis	1	0	0
Haemonchosis	1	0	0
Bloat	1	1	0
Colisepticemia	1	0	0
Cysticercosis	1	1	0
Tetanus	1	0	0
Enzootic bovine leucosis	1	1	0

Table of articles

Abbreviations:<br><br>ECF – East Coast fever<br><br>FMD – Foot and mouth disease<br><br>AAT – African Animal Trypanosomosis<br><br>TBD – Tick-borne diseases<br><br>LSD – Lumpy skin disease<br><br>TB – Tuberculosis

Note: Clicking on the article number links to an expanded summary of the article below. The “web” link connects to the abstract of the article online or to full text for open access articles.

#	Citation	Country	Study design	Pathogen/ disease	Findings
1<br>1<br>web	(W. Jemberu, Mourits, & Hogeveen, 2015)	Ethiopia	Cross-sectional, Questionnaire, 293 farmers (84 crop-livestock mixed farmers, 100 pastoralists,	FMD	**Incidence/ Prevalence:** The majority of the crop-livestock-mixed farmers (67%) and market oriented farmers (55%) reported a frequency of every five and two years, respectively.<br>**Impact:** When FMD impact was compared with the consequences of other livestock diseases, it

			109 market oriented farmers)		was perceived as medium by the majority of farmers.<br>**Perceptions:** All pastoralists, 86% of the crop-livestock-mixed farmers, and 74% of the market-oriented farmers knew of FMD, and 97%, 93% and 63% of the farmers who knew the disease in the respective systems experienced the disease in their own herd.
2<br>1<br>web	(Baluka, 2016)	Uganda	Mixed-methods, Socio-economic survey,<br>873 farmers in 4 districts,<br>Dairy,<br>Smallholder & small commercial	FMD	**Impact:** All actors along the cattle marketing chain incur losses during FMD outbreaks, but smallholder farmers are most affected. Farmers with small and medium herds incurred higher control costs, whereas large herds experienced the highest milk losses. FMD control cost per head of cattle during an outbreak was highest in the small herds ($19 USD), more than three and eight times for medium and large herds, respectively. Mortality losses in small and medium case study herds were $429 and $214, respectively, and there was no mortality in large herds. The salvage losses in small and medium case study herds were $143 and $113, respectively, and there were no salvage losses in large herds, i.e. large herds did not sell any cattle at salvage prices during outbreaks and quarantine. Milk losses were estimated at $267 for small herds, $629 for medium herds, and $2,670 for large herds.

3<br>1<br>web	(W. T. Jemberu, Mourits, Woldehanna, & Hogeveen, 2014)	Ethiopia	Outbreak investigation, Questionnaire, 512 respondents, Smallholders and pastoralists	FMD	**Impact:** Foot and mouth disease morbidity rates of 85.2% and 94.9% at herd level; and 74.3% and 60.8% at animal level in the affected herds were determined for crop–livestock mixed system and pastoral system, respectively. The overall and calf specific mortality rates were 2.4% and 9.7% for the crop–livestock mixed system, and 0.7% and 2.6% for the pastoral system, respectively. The economic losses of foot and mouth disease outbreak due to milk loss, draft power loss and mortality were on average USD 76 per affected herd and USD 9.8 per head of cattle in the affected herds in crop–livestock mixed system; and USD 174 per affected herd and USD 5.3 per head of cattle in the affected herds in the pastoral system.
4<br>2<br>web	(W. T. Jemberu et al., 2016)	Ethiopia	5 year retrospective study of outbreak data, 115 randomly selected districts	FMD	**Incidence/ Prevalence:** The national incidence of FMD outbreaks during the study period was 1.45 outbreaks per five district years. Outbreaks were geographically widespread affecting all major regional states in the country.
5<br>2<br>web	(Negusssie, Kyule, Yami, Ayelet, & Jenberie T, 2011)	Ethiopia	Outbreak investigation, Multiple production systems	FMD	**Impact:** Of a total of 7,781 cattle observed and recorded in six districts, 1,409 (19.6%) were infected, and 15 (0.12%) died during outbreaks of FMD.
6	(Abdela, 2017)	Ethiopia	Review of sero-prevalence	FMD	**Incidence/ Prevalence:** Seropositivity ranges from 6- 43 % in cattle and from 4- 11% in small

2<br>web			reports 2004-2016		ruminants. Five of seven serotypes have endemic distribution: serotypes O, A, C, SAT1 and SAT2.
7<br>3<br>web	(Kerfua et al., 2019)	Uganda Tanzania	Phylogenetic analysis of FMD virus circulating along Tanzania and Uganda border	FMD	8 out of the 11 sequences obtained belonged to serotype O and three belonged to serotype A. The vaccine strain K35/1980 belongs to Africa G VII with an average sequence divergence of 20.5% from the study sequences. The genetic distances between current vaccine strains and circulating field strains underscores the crucial need for regular vaccine matching.
8<br>3<br>web	(Sulayeman, Dawo, Mammo, Gizaw, & Shegu, 2018)	Ethiopia	Cross-sectional, Serosurvey, Outbreak investigation, Molecular characterization, 378 cattle	FMD	**Incidence/ Prevalence:** 28.8% (n = 378) cattle showed signs and lesions suggestive of FMD and 34 samples were subjected to virus isolation. Three different serotypes (A, O and SAT 2) were responsible for the outbreaks of the disease.
9<br>3<br>web	(Lyons et al., 2015)	Kenya	Longitudinal cohort study, Outbreak investigation, Focus group, Commercial dairy, 644 cows	Foot and Mouth Disease (FMD), Mastitis	**Incidence/ Prevalence:** 400/644 (62.1%) of cattle were reported to be affected with FMD due to serotype SAT2. 63/644 (9.78%) developed clinical mastitis. A univariable analysis showed no effect of FMD on rate of mastitis. 76/644 (11.8%) animals were culled.
10	(Coffin, Monje,	Uganda	Participatory epidemiology,	Anthrax TBD	**Perceptions:** Tick-borne diseases were ranked number one by farmers in terms of importance

2 web	Asiimwe-Karimu, Amuguni, & Odoch, 2015)		Questionnaire, Focus groups, Agro-pastoralists, 11 focus groups and 76 structured questionnaires in 7 villages	Brucellosis	and brucellosis was ranked number two. Although anthrax is perceived as costly, it is rare, which led participants to rank it as less important in livelihood success.<br>**Impact:** Participants did mention livestock and human illness and deaths from anthrax, yet they ranked other impacts as more important. Depreciation of the value of the animal or products, cost of treatment, and particularly market closure were all emphasized.
11 2 web	(Sijapenda, Komba, & Nonga, 2017)	Tanzania	Cross-sectional Seroprevalence with 300 cattle Questionnaire with 60 farmers Smallholders	Brucellosis	**Perceptions:** 85% of farmers in the study were not aware of brucellosis.<br>**Incidence/ Prevalence:** Proportions of positive reactors to brucellosis were 6.0% and 5.2% based on RBPT and c-ELISA respectively. Risk factors included higher age of cattle, history of abortion, and improper disposal of aborted material.
12 2 web	(Kabi, Muwanika, & Masembe, 2015)	Uganda	Cross-sectional, Seroprevalence, 925 indigenous breed cattle randomly selected from 209 herds Agro-pastoralists	Brucellosis	**Incidence/ Prevalence:** *Brucella* antibodies occurred in approximately 8.64% (80/925) and 28.70% (95% CI: 22.52, 34.89) of the sampled individual cattle and herds, respectively. Similar seroprevalence reported for indigenous cattle in Tanzania and Ethiopia. Recent incidences of abortions within the previous 12 months were significantly ($p < 0.001$) associated with seropositive herds. Culling of older cattle (>72 months) and those which have had an abortion

					especially in the third trimester as may be more associated with brucellosis.
13<br>3<br>web	(Madut et al., 2018)	South Sudan	Serosurvey of humans (87) and cattle (893), Cross-sectional, Agro-pastoralists	Brucellosis	**Incidence/ Prevalence:** Bovine brucellosis prevalence was 31% (95%CI = 28.0-34.2), with the highest 63% (95%CI = 53-70) and lowest 10% (95%CI = 4.5-20.1) prevalence estimates in Wau and Gogrial states respectively.
14<br>2<br>web	(Makita et al., 2011)	Uganda	Cross-sectional, Serosurvey, Small commercial/ Dairy,<br>423 cows in 177 herds	Brucellosis	**Incidence/ prevalence:** The adjusted herd prevalence of brucellosis was 6.5% (11/177) and the adjusted individual animal prevalence was 5.0% (21/423).
15<br>2<br>web	(Njeru et al., 2016)	Kenya	Literature review, Pastoralists, Smallholders	Brucellosis	**Incidence/ Prevalence:** Seroprevalences for *Brucella (B.) abortus* and *B. melitensis* were reported as 9.9–16.9 % for cattle, 11.9 % for sheep, and 13.0–16.1 % for goats in pastoralist managed herds. Seroprevalences of 9.9–16.9 % for cattle, 11.9 % for sheep, and 13.0–16.1 % for goats were found in pastoralist managed herds.
16<br>2<br>web	(Vhoko et al., 2018)	Zimbabwe	Longitudinal, Smallholder and commercial dairy,<br>12,359 milk samples from	Brucellosis	**Incidence/ Prevalence:** 31% (95% C.I.: 24% - 38%) of the farms tested were found positive (47/156).

			156 farms over 5 years		
17<br>3<br>web	(Edao et al., 2018)	Ethiopia	Cross-sectional, Serosurvey, Knowledge, attitudes, and practices (KAP) questionnaire, Dairy, 1,550 cattle in 127 farms	Brucellosis, Abortion, Retention foetal membrane	**Incidence/ Prevalence:** More than 70% of medium and large farms and 43% of small sized farms had reproductive problems (abortion, stillbirth, retained fetal membrane and repeat breeding) in their farms. 43 of the 1,550 tested cattle tested positive for brucellosis infection using serological tests.
18<br>2<br>web	(Mugizi et al., 2015)	Uganda	Cross-sectional, Serosurvey, Smallholders, 500 sera (from 116 cattle herds in Gulueg), 507 sera (from 50 herds in Soroti town)	Brucellosis Abortion	**Incidence/ prevalence:** Individual animal-level and herd-level seroprevalences were 7.5% (76/1007) and 27.1% (45/166) respectively. Seroprevalence of brucellosis in cattle with a history of abortion and retained placenta in Soroti were 8% and 11%, respectively.
19<br>1<br>web	(Okumu, John, Wabacha, Tsuma, & VanLeeuwen, 2019)	Kenya	Cross-sectional serosurvey of 398 dairy cows, 17 month longitudinal sero-epidemiological survey of 260	Bovine viral diarrhoea virus, Brucellosis, *Neospora caninum*, Abortion	**Incidence/ Prevalence:** In the 398 randomly selected cattle on 64 dairy herds, the seroprevalences of antibodies to Bovine viral diarrhoea virus (BVDV), *Neospora caninum* (NC), and Brucella abortus (BA) were 79.1, 25.6 and 16.8%, respectively.<br>**Impact:** Among 260 monitored pregnant dairy cattle on the same 64 dairy farms, an incidence

			dairy cows on 64 farms,<br>Small and large scale dairy		risk for abortion of 10.8% (28/260) was identified, while the incidence of other foetal losses was 1.1% (3/260). *Neospora caninum* was associated with most cases (29.0%) of fetal losses, followed by mixed infections of NC and BVDV (12.9%), BVDV (9.9%) and co-infections of BA and NC (6.5%).
20<br>1<br>web	(Seyoum, Terefe, & Ashenafi, 2013)	Ethiopia	Cross-sectional, Questionnaire, 84 agro-pastoralists from 2 districts	African Animal Trypanosomosis (AAT), Ectoparasites, Pasteurellosis, Blackleg, Endoparasites	**Incidence/ prevalence:** By frequency of report by respondents: Trypanosomosis (95.2%), fly strike/nuisance (66.7%), ectoparasite (tick) infestation (52.4%), pasteurellosis (42.9%), blackleg (35.7%) and internal parasites (33.3%).<br>**Impact:** According to the respondents, among the 65 cattle deaths reported, trypanosomiasis accounted for 65% of the total annual deaths in the last 12 months of the year (2011/2012) and the mortality rate of cattle was 6.4%.<br>**Perceptions:** 95% reported that they are familiar with bovine trypanosomosis. 94% of the respondents listed bovine trypanosomosis as the prime economically important cattle disease in the river basins/districts.
21<br>2<br>web	(Moti et al., 2015)	Ethiopia	Longitudinal Trypanosome testing of 106 positive and 119 negative bovines over 6 months Agro-pastoralists	AAT	**Incidence/ Prevalence:** Diminazene aceturate (DA) was used by 95.5% of the farmers the year preceding this survey. Species detection of trypanosomes showed 38 (59.4%) *Trypanosoma congolense* savannah, of which 31 (81.6%) showed a DA resistant profile.

					**Perceptions:** Livestock keepers are focusing on sick animals and do not think in terms of prophylaxis. There was a trend to overdose young small animals and to under dose large ones. Oxen were treated very frequently (nearly 20 times/year) and calves almost never.
22<br>3<br>web	(Simwango et al., 2017)	Tanzania	Cross-sectional, Molecular prevalence, 1002 cattle and 886 tsetse flies	AAT	**Incidence/ Prevalence:** The overall prevalence of trypanosome infections was 17.2% in cattle and 3.4% in tsetse flies. *Trypanosoma vivax, T. brucei, T. simiae, T. theileri* and *T. congolense*, were present. No human-infective trypanosomes were detected in either cattle or tsetse fly DNA.
23<br>2<br>web	(Magona, Walubengo, & Odimin, 2008)	Uganda	Outbreak investigation, 401 cattle	Trypanosomosis/ acute haemorrhagic syndrome	**Incidence/ Prevalence:** The prevalence of bovine trypanosomosis in herds experiencing mortality (21.5%) was significantly higher than in those without mortality (2.6%) suggesting *T. vivax*-induced acute haemorrhagic syndrome.<br>**Impact:** Out of an original population of 844 cattle, 295 (35%) died.
24<br>3<br>web	(Kimaro, Toribio, & Mor, 2017)	Tanzania	Participatory epidemiology, Qualitative methods, Pastoralists	East Coast fever (ECF), AAT	**Perceptions:** Comparing 2014–1984, participant groups consistently reported declines in rainfall, vegetation cover and quality pasture, as well as increases in severe droughts. Experiences with ECF/AAT and vector abundance between these time periods was more variable across villages, and likely relates to changes in climate and animal management practices over the last 30 years.

25<br>2<br>web	(Weny et al., 2017)	Uganda	Cross-sectional, Serosurvey, 186 cattle and 317 goats from 40 households, Pastoralists	Animal African trypanosomiasis, Anaplasmosis, Theileriosis, Babesiosis	**Incidence/ Prevalence:** Hemoparasite infection prevalence of 30.0% in cattle. Prevalence of specific genera of hemoparasites in cattle was *Theileria*, 15.1%; *Trypanosoma*, 4.30%; and *Anaplasma*, 1.61%.
26<br>2<br>web	(Musinguzi et al., 2016)	Zambia	Cross-sectional, PCR-based survey, Pastoralists, 472 cattle and 53 goats	Animal African trypanosomiasis, Anaplasmosis, Theileriosis, Babesiosis	**Incidence/ Prevalence:** Hemoparasites detected in cattle and goats were of the genera Theileria, Anaplasma, and Trypanosoma with a prevalence of 15.1%, 1.6%, and 4.3% respectively in cattle, and 10%, 6.0%, and 0.0%, respectively in goats.
27<br>1<br>web	(Muraguri, McLeod, McDermott, & Taylor, 2005)	Kenya	Observational Longitudinal Smallholder 130 calves in 67 farms	ECF Trypanosomosis Anaplasmosis Babesiosis	**Incidence/ Prevalence:** The incidences of East Coast fever (ECF) (23.1%) and trypanosomosis (29.1%) were the highest among the vector-borne diseases. The annual incidence rates of anaplasmosis and babesiosis were 10.9 and 1.2%, respectively.<br>**Impact:** The mortality incidence rates of ECF and trypanosomosis were 10.9 and 3.6%, respectively. There was no mortality arising specifically from anaplasmosis or babesiosis.
28<br>2<br>web	(Gachohi, Ngumi, Kitala, & Skilton, 2010)	Kenya	Cross-sectional, Seroprevalence, Smallholder, 440 cattle	Tick-borne diseases	**Incidence/ Prevalence:** Overall estimation of seroprevalences and their 95% confidence limits were: *T. parva* (19% [14%, 25%]), *T. mutans* (25% [20%, 29%]), *A. marginale* (58% [52%, 64%]) and *B. bigemina* (19% [15%, 23%]).

29<br>2<br>web	(Vudriko et al., 2016)	Uganda	Cross-sectional, Larval population test, Small commercial, 54 purposively selected farms (from 17 districts) that mostly had a history of acaricide failure	Acaricide resistance, Ectoparasites	**Incidence/ Prevalence:** 93.5 % (29/31) of the larval population tested had resistance to at least one class of acaricide molecule; all of them belonging to the genus *Rhipicephalus*. Resistance against synthetic pyrethroid (SP) was detected in 90.0 % (27/30) of the tick populations tested. Resistance was also detected against co-formulations of organophosphate and synthetic pyrethrois (43.3 %), organophosphate chlorfenvinphos (13.3 %), and amitraz (12.9 %).
30<br>2<br>web	(Katale et al., 2013)	Tanzania	Cross-sectional, Intradermal tuberculin skin test, Questionnaire, 1,103 indigenous cattle from 32 herds and 108 pastoralist respondents	Tuberculosis	**Incidence/ prevalence:** Apparent individual animal prevalence of tuberculin reactors was 2.4%, whereas the true prevalence was 0.6%. Herd prevalence was 50% (16/32).<br>**Perceptions:** 65% individuals had not heard about bovine tuberculosis at all.
31<br>2<br>web	(Romha, Gebru, Asefa, & Mamo, 2018)	Ethiopia	Systematic literature review of epidemiology of *M. bovis* and	Tuberculosis	**Incidence/ Prevalence:** Prevalence of tuberculous lesion, ranging from 3.5 to 10.2%, have been reported in cattle slaughtered in different abattoirs. Both the prevalence and severity of pathology have been found higher in HF than in

			M. tuberculosis in livestock		zebu cattle, and cattle managed intensively/kept in-door were more at risk than cattle kept in pasture. PPD reactors (positive TB skin test) in small ruminants occur in low prevalence ranging from 0.6% to 7.6%. The main strain was found to be SB1176.
32<br>2<br>web	(Kemal et al., 2019)	Ethiopia	Cross-sectional, Prevalence study, Questionnaire, 315 cattle, Mixed production systems	Tuberculosis	**Incidence/ Prevalence:** The overall prevalence of bovine tuberculosis was 20.3% (n = 64) in dairy cattle (positive intradermal tuberculin skin test). From a total of 43 farms/households tested, 22 were positive; each farm exhibited at least one tuberculin positive reactor animal with a total herd level prevalence of 51.2%.<br>**Perceptions:** Only 33% had the knowledge of or had heard about bovine tuberculosis and 23% respondents were aware of the zoonotic importance of the disease. More than 50% of the interviewees prefered consuming raw milk to boiled milk.
33<br>3<br>web	(Moiane et al., 2014)	Mozambique	Cross-sectional, Intradermal tuberculin skin test, 1,136 animals (125 commercial, 1,011 smallholders)	Tuberculosis	**Incidence/ prevalence:** Overall prevalence of positive reactors was 39.6%.

34<br>1<br>web	(Molla, de Jong, Gari, & Frankena, 2017)	Ethiopia	Cross-sectional, questionnaire, 243 herd owners, Commercial dairy and smallholders	Lumpy skin disease	**Incidence/ Prevalence:** 21% of cattle were reported to be infected (941 of 4430) with infected cattle in 82% of herds (200 of 243).<br>**Impact:** The median total economic loss of an LSD outbreak at herd level was USD 1,176 (USD 489 in subsistence farm and USD 2,735 in commercial farm). At herd level, the largest component of the economic loss was due to mortality (USD 1,000) followed by milk loss (USD 120). LSD control costs were the least contributor to herd level losses. The financial analysis showed a positive net profit of USD 136 (USD 56 for subsistence farm herds and USD 283 for commercial herds) per herd due to LSD vaccine investment.
35<br>1<br>web	(Ochwo et al., 2018)	Uganda	Retrospective, longitudinal study of government outbreak records 2002-2016	Lumpy skin disease	**Incidence/ Prevalence:** Morbidity, mortality, and case fatality rates were 4.77, 0.03 and 0.72%, respectively. 1,161 outbreaks and 319,355 cases of LSD were reported from 55 out of 56 districts of Uganda.<br>**Impact:** Morbidity, mortality, and case fatality rates were 4.77, 0.03 and 0.72%, respectively.
36<br>2<br>web	(Gari, Bonnet, Roger, & Waret-Szkuta, 2011)	Ethiopia	Cross-sectional, Questionnaire, 747 smallholder farmers purposively selected across 5 districts	Lumpy skin disease	**Impact:** The financial cost in infected herds was estimated to be USD 6.43 (5.12–8) per head for local zebu and USD 58 (42–73) per head for Holstein Fresian/crossbred cattle.

37<br>2<br>web	(Swiswa, Masocha, Pfukenyi, Dhliwayo, & Chikerema, 2017)	Zimbabwe	Retrospective spatial analysis of 20 (1995-2014) years of cases, Multiple production systems	Lumpy skin disease	**Incidence/ Prevalence:** Number of outbreaks is increasing and geographic distribution is increasing. From 1995–1999, there were only ten hotspots located mostly in the communal land in the northern, southern and south-eastern parts of the country. During the period 2000–2014, the number of significant hotspots increased from 115 in the second 5-year period to 187 in the last 5-year period. At the same time, the geographic distribution of hotspots expanded to other regions including the western areas of the country.
38<br>2<br>web	(Gari, Warer-Szkuta, Grosbois, Jacquiet, & Roger, 2010)	Ethiopia	Cross-sectional Questionnaire 330 households Agro-pastoral	Lumpy skin disease (LSD)	**Incidence/ Prevalence:** Overall observed LSD prevalence at animal-level was 8.1% (95% CI 7.3–8.9).<br>**Impact:** Observed mortality was 2.12% (95% CI 1.73–2.6).
39<br>2<br>web	(Mafirakureva, Saidi, & Mbanga, 2017)	Zimbabwe	2 year retrospective study of veterinary records, Molecular characterization	Lumpy skin disease	**Incidence/ Prevalence/ Impact:** Over the 2-year period, a total of 10,038 cases and 880 deaths (8.77 %) were recorded. LSD was reported from all regions of the country. Phylogenetic analyses showed that the viruses formed two major clusters implying that at least two strains of LSDV are in circulation in Zimbabwe.
40<br>2<br>web	(Ayelet et al., 2014)	Ethiopia	Longitudinal, Questionnaire, 2,174 cattle (1,992 from 40 commercial	Lumpy skin disease (LSD)	**Incidence/ prevalence:** Retrospective analysis of data showed that 1,675 outbreaks of LSD were reported to the Ethiopian Ministry of Agriculture from 2001-2011.

			feedlots, 182 cattle from 40 smallholder farms)		**Impact:** Morbidity and mortality rates of 13.61% (296) and 4.97% (108) were recorded in the active outbreak. Morbidity (15.1%) and mortality rates (5.37%) were higher in vaccinated animals than in unvaccinated animals, indicating vaccine failure.
41<br>2<br>web	(Kivaria, Noordhuizen, & Msami, 2007)	Tanzania	Cross-sectional<br>Smallholder Dairy<br>182 cows	Mastitis	**Incidence/ Prevalence:** Clinical inspection indicated that 3.8% of the lactating cows had clinical mastitis. Subclinical mastitis was detected in 90.3% of lactating cows screened.<br>**Perceptions:** 5% of dairy workers were aware of the presence of subclinical mastitis.
42<br>2<br>web	(Mekonnen et al., 2017)	Ethiopia	Cross-sectional<br>Prevalence study<br>167 farms<br>Small commercial Dairy	Mastitis	**Incidence/ Prevalence:** On average, 33% of the quarters and 62% of the cows were California Mastitis Test (CMT) positive, but the within herd quarter level prevalence ranged between 0 and 100%. A total of 1,543 milk samples, being 27 quarters that showed signs of CM, 606 CMT positive quarters and 910 CMT negative quarters were cultured, respectively 40%, 67% and 47% was positive on bacteriological culture.
43<br>2<br>web	(Abebe, Hatiya, Abera, Megersa, & Asmare, 2016)	Ethiopia	Cross-sectional, Prevalence study, 529 lactating cows from 95 herds,<br>Small commercial dairy	Mastitis	**Incidence/ Prevalence:** The prevalence of mastitis at herd-level was 74.7% (95% CI: 64.5, 82.8). The corresponding cow-level prevalence was 62.6% (95% CI: 58.3, 66.7), of which 59.2 and 3.4% were sub-clinical and clinical mastitis cases, respectively. *S. aureus* was isolated from 51.2% of the milk samples cultured and 73.2% of the herds affected with mastitis.

44<br><br>2<br><br>web	(Sitawa, Mbogoh, Gathuma, & Kairu, 2016)	Kenya	Cross-sectional<br>330 households<br>Dairy Smallholder/ small commercial	East Coast fever	**Impact:** ECF-vaccinating households realized an overall net economic return of Kshs 44,575 (about US$ 450) per cow per year while the ECF non-vaccinating households realized a net loss of Kshs 9,975 (about US$ 100) per cow per year. Study estimates ECF non-vaccinated dairy animals are twice more likely to die from ECF.
45<br><br>2<br><br>web	(Kerario et al., 2017)	Tanzania	Cross-sectional, Serosurvey, 648 cattle across 3 regions, Agro-pastoralists, Pastoralists	ECF	**Incidence/ Prevalence:** The prevalence of *T. parva* across the three regions was 14.2%. There was high variation in tick burden and *T. parva* prevalence across the regions.
46<br><br>2<br><br>web	(Malak et al., 2012)	South Sudan	Cross-sectional, Participatory epidemiology, Seroprevalence for cattle, Agro-pastoralists	Diarrhoea<br>ECF<br>TBD<br>Heartwater<br>Blackquarter<br>CBPP<br>CCPP	**Perceptions:** Diseases ranked as important by agro-pastoralists included diarrhoea in small ruminants, ECF, ticks (destructive in their own right rather than as vectors for TBDs), and a syndrome at various times consistent with heartwater, blackquarter, and contagious bovine pleuropneumonia or contagious caprine pleuropneumonia.<br>**Incidence/ Prevalence:** Prevalence of *B. bovis* was found to be low (4.0% and 7.4% in Kajo Keji and Yei, respectively). 35% of the samples screened with the *T. parva* gene nested PCR assay were positive.<br>**Impact:** For Kajo Keji country only: East Coast fever was estimated to have an annual relative

					incidence of 19.2% and a case fatality rate of 70%. Mortality due to ECF was estimated at 13.5%. Therefore, ECF was responsible for 26.7% and 34.6% of all morbidity and mortality, respectively.
47<br>1<br>web	(Wanyoike & Rich, 2010)	Kenya	Value chain analysis, semi-structured interviews with 45 respondents, social accounting matrix, Multiple production systems	Rift Valley fever	**Impact:** The 2007 RVF outbreak in Kenya had wide-ranging impacts on the livestock sector and other segments of the economy that are often overlooked in the analysis of animal disease. The main negative effects on producers were caused by the loss of animals that died of RVF, which in turn had impacts on food security and future income (e.g., from the loss of future stock caused by animal abortions). Total economic losses from livestock mortality in these two regions were calculated at over US$9.3 million. As a result of the RVF outbreak, the value of total domestic supply in a variety of areas from petroleum to food staples to tourism fell by US$32 million.
48<br>3<br>web	(Shabani, Ezekiel, Mohamed, & Moshiro, 2015)	Tanzania	Cross-sectional, Questionnaire, 463 community members, Pastoralists	Rift Valley fever	**Impact:** Ninety percent (90%) agreed that RVF posed a threat to public health as well as the local livestock economy (5 point Likert scale).<br>**Perceptions:** Most of the respondents (97.6 %) had heard about RVF. Overall knowledge regarding RVF vector, modes of transmission, symptoms and prevention among respondents was low, with only 11.3% recorded as knowledgeable.

49<br>2<br>web	(Nyakarahuka et al., 2018)	Uganda	Abattoir study, Serosurvey of humans (655) and cattle, goats, sheep (1,051)	RVF	**Incidence/ Prevalence:** The overall animal seropositivity was 13%, with 27% of cattle, 7% of goats, and 4% of sheep seropositive.
50<br>3<br>web	(Nanyingi et al., 2017)	Kenya	Cross-sectional, Serosurvey, 370 cattle, sheep, and goats, Purposive sampling in RVF-prone area, Agro-pastoralists	Rift Valley fever	**Incidence/ Prevalence:** Sheep, cattle, and goats had seroprevalences of 32.2% (95% CI [20.6–31]), 33.3% (95% CI [6.7–60]), and 25.8% (95% CI [22.4–42]), respectively.
51<br>3<br>web	(Hassan, Ahlm, Sang, & Evander, 2011)	Sudan	Outbreak report	Rift Valley fever	The 2007 outbreak was the first reported RVF outbreak in Sudan for 36 years, although studies indicate that the virus has been circulating in Sudan.
52<br>2<br>web	(Onono, Wieland, & Rushton, 2014)	Kenya	Modelling	CBPP	**Impact:** The impact of CBPP on pastoralists in Kenya is estimated at US$ 7.6 million per year. If all pastoralists whose cattle are at risk of infection adopt vaccination as a control strategy, the impact will only be US$ 3.3 million per year with savings amounting to US$ 4.3 million.
53<br>3<br>web	(Kairu-Wanyoike et al., 2014)	Kenya	Cross-sectional, Willingness to pay for vaccine, Questionnaire, 190 smallholders	Contagious bovine pleuropneumonia (CBPP)	**Perceptions:** 87% of respondents had some knowledge of CBPP, 61.6% gave CBPP priority with regard to control. 70.0% saw a high likelihood of their cattle suffering CBPP. 54% of households had experienced adverse CBPP post-vaccination

					reactions in their herds. Only 34.4% of farmers were willing to pay an amount greater than zero for the current vaccine and vaccination. The remaining 65.6% were not willing to pay (zero and negative amount) and would need compensation to vaccinate their cattle against CBPP using the current vaccine.
54<br>2<br>web	(Carlson et al., 2019)	Sub-Saharan Africa	Global incidence database of human, wildlife, and livestock anthrax cases from multiple sources spanning 71 countries from 1914-2018	Anthrax	**Incidence/ Prevalence:** Using past occurrence as a proxy for risk, in Sub-Saharan Africa 30.5 million cattle, 22.4 million goats, and 14.5 million sheep are at risk.<br>**Impact:** Vaccination rates in Sub-Saharan Africa are 0-6%.
55<br>3<br>web	(Mohamed et al., 2018)	Sudan	Cross-sectional, Serosurvey, 180 cattle	Theileriosis	**Incidence/ Prevalence:** Prevalence rate of hemoparasite infection was 63%. Nineteen of 44 animals were co-infected with more than one species of Theileria. Phylogenetic analysis revealed three Theileria spp. that were predominant in cattle including pathogenic *T. annulata* and apathogenic *T. velifera* and *T. mutans.*
56<br>2	(Nyirenda et al., 2019)	Zambia	Abattoir study from 2013-2017, 69,152 carcasses	Fascioliasis	**Incidence/ prevalence:** 64% of carcasses (44,511) were positive for adult *Fasciola* worms. According to the intensity of pathological lesions, 55.3% constituted severely affected livers, 30.3% were moderately affected livers and 14.4% were lightly

web					affected livers. Most prevalent was *Fasciola gigantica* (56%) and it mostly affected animals with poor body condition (71%).<br>**Impact:** The economic loss was estimated to be $3.60 USD/kg of liver multiplied by 164,600 kg condemned and destroyed between 2013 and 2017, for a total of $592,560 USD.
57<br>2<br>web	(Assenga, Matemba, Muller, Mhamphi, & Kazwala, 2015)	Tanzania	Cross-sectional, Serosurvey, Agro-pastoralists, 1,103 cattle, 248 goats, 267 humans	Leptospirosis	**Incidence/ prevalence:** The prevalence of leptospiral antibodies was 30% in cattle, 8% in goats, and 30% in humans.
58<br>2<br>web	(Schoonman & Swai, 2010)	Tanzania	Cross-sectional Seroprevalence Smallholder, dairy represented, 655 serum samples	Leptospirosis	**Incidence/ Prevalence:** Positive titers were detected in 30.3% [95% confidence intervals (CI) =26.7– 33.9] of cattle and 58.5% (95% CI=49.5–67.1) of herds, respectively.
59<br>2<br>web	(Ndhlovu & Masika, 2015)	Zimbabwe	Cross-sectional, Questionnaire, 185 smallholder cattle owners	Dermatophilosis	**Incidence/ Prevalence:** 45.4% of the herds were clinically positive for dermatophilosis.<br>**Perceptions:** Concerns were raised by farmers about the effectiveness of these drugs against bovine dermatophilosis. 74.1% of the farmers treated their cattle using antibiotics. Tetracyclines were the antibiotics used by most farmers (79.3%), with 19.1% using penicillin.

60<br>2<br>web	(Thumbi et al., 2015)	Kenya	Longitudinal, Syndromic surveillance, 1500 households, Smallholder	Syndromes	**Incidence/ Prevalence:** Of the illness cases reported among each livestock species, gastro-intestinal tract syndrome cases were the most common, comprising of 52%, 53%, and 34% of total cases in cattle, goats, and sheep respectively. For these three species, respiratory syndromes were the second most commonly observed.
61<br>2<br>web	(Nonga & Karimuribo, 2009)	Tanzania	Longitudinal, Abattoir study, 115,186 cattle, Multiple production systems	Hydatidosis	**Incidence/ Prevalence:** 4% infection rate in cattle.
62<br>2<br>web	(Dorny, Phiri, Gabriel, Speybroeck, & Vercruysse, 2002)	Zambia	Seroprevalence Cross-sectional 628 cattle Multiple production systems	Cysticercosis	**Incidence/ Prevalence:** 6.1% seroprevalence of cysticercosis. Cysticercosis was significantly more prevalent in feedlots and traditional farming systems than in dairy farms.<br>**Impact:** Economic significance of this parasite may be considerable due to downgrading and condemnation of carcasses.
63<br>2<br>web	(Wardrop et al., 2016)	Kenya	Cross-sectional, Serosurvey of 2,049 humans and 955 cattle in 416 homesteads, Pastoralists	Q fever	**Incidence/ Prevalence:** Seroprevalence of 10.5% in cattle.
64	(Getaw, Beyene,	Ethiopia	Cross-sectional, Abattoir study,	Hydatidosis	**Incidence/ Prevalence:** Hydatidosis was prevalent in 47% of cattle, 29% of sheep, and 7% of goats. In

2 web	Ayana, Megersa, & Abunna, 2010)		Retrospective data analysis, 1,152 ruminants (cattle, sheep, goats)		cattle, 326 (55%) of the lung, 219 (37%) of the liver, 21 (4%) of the spleen, 15 (3%) of the heart and 10 (2%) of the kidney were found to be infected with hydatid cysts. According to the retrospective data, a total of 107,333 cattle were slaughtered and during this period 13,519 of the liver, 18,304 of the lung, 1,142 of the kidneys, 537 of the hearts and 150 of the spleens were found to be infected with hydatidosis. **Impact:** The total annual economic loss incurred due to organ meat condemnation at meat inspection from hydatidosis in ruminants slaughtered at Adama municipal abattoir was estimated to be to $5,870 USD.
65 2 web	(Ibrahim, Elfahal, & El Hussein, 2012)	South Sudan	Cross-sectional Serosurvey Dairy 262 cattle from 25 herds	*Neospora caninum* infection	**Incidence/ Prevalence:** The prevalence rates of *N. caninum* antibodies in cattle were high both at herd level (44%) and at individual animal level (10.7%). This study represents the first serological evidence for presence of *N. caninum* infection in Sudan.
66 3 web	(Gelaye, Mekonnen, Jenberie, & Ayelet, 2013)	Ethiopia	Cross-sectional, Outbreak intectigation, Pastoralist	Sheep-associated malignant catarrhal fever in cattle	**Incidence/ prevalence:** First report of a diagnostic investigation resulting in the detection of ovine OvHV-2 in cattle and confirming the existence of sheep-associated malignant catarrhal fever in Ethiopia. **Impact:** Disease outbreak caused morbidity and mortality in 18 cattle out of a total of more than

					2,000 kept by farmers in the village and surrounding area.
67 3 web	(Pandey et al., 2017)	Zambia	Case report, Dairy	Enzootic bovine leucosis	**Incidence/ Prevalence:** Two clinical cases of EBL were confirmed in a dairy cattle herd in Zambia. This is the first report of confirmed EBL in Zambia. **Impact:** Economic impact is perceived to be minimal however BLV is likely underdiagnosed.

Article summaries

Jemberu, Wudu T., Mourits, M. C. M., & Hogeveen, H. (2015). Farmers' Intentions to Implement Foot and Mouth Disease Control Measures in Ethiopia. *PloS One*, *10*(9), e0138363. https://doi.org/10.1371/journal.pone.0138363

Cross-sectional study to explore farmers' intentions to implement foot and mouth disease (FMD) control in Ethiopia, and to identify perceptions about the disease and its control measures that influence these intentions using the Health Belief Model framework. A questionnaire was administered to 293 farmers from multiple production systems (84 crop-livestock mixed farmers, 100 pastoralists, and 109 market oriented farmers).

- The majority of the crop-livestock-mixed farmers (67%) and market oriented farmers (55%) reported a frequency of every five and two years, respectively.
- When FMD impact was compared with the consequences of other livestock diseases, it was perceived as medium by the majority of farmers.
- All pastoralists, 86% of the crop-livestock-mixed farmers, and 74% of the market-oriented farmers knew of FMD, and 97%, 93% and 63% of the farmers who knew the disease in the respective systems experienced the disease in their own herd.
- The majority of farmers in the pastoral (94%) and market oriented (92%) systems had the intention to implement vaccination with charge but only 42% of the crop-livestock mixed farmers had the intention to do so. Most farmers in all production systems perceived vaccination as a highly effective disease control measure.

Baluka, S. A. (2016). Economic effects of foot and mouth disease outbreaks along the cattle marketing chain in Uganda. *Veterinary World*, *9*(6), 544–553.

Mixed-methods socio-economic survey of 873 farmers across four districts in Uganda to assess the economic impact of foot and mouth disease outbreaks and quarantine policies on farmers with small, medium, and large herds. This incorporates smallholders, dairy, and small commercial.

- All actors along the cattle marketing chain incur losses during FMD outbreaks, but smallholder farmers are most affected.
- Farmers with small and medium herds incurred higher control costs, whereas large herds experienced the highest milk losses. FMD control cost per head of cattle during an outbreak was highest in the small herds ($19 USD), more than thrice and eight times for medium and large herds, respectively. Milk losses were estimated at $267 for small herds, $629 for medium herds, and $2,670 for large herds.

- Mortality losses in small and medium case study herds were $429 and $214, respectively, and there was no mortality in large herds. The salvage losses in small and medium case study herds were $143 and $113, respectively, and there were no salvage losses in large herds, i.e. large herds did not sell any cattle at salvage prices during outbreaks and quarantine.
- Total income earned by the workers at the processing level was reduced by 23% per month.

Jemberu, W. T., Mourits, M. C. M., Woldehanna, T., & Hogeveen, H. (2014). Economic impact of foot and mouth disease outbreaks on smallholder farmers in Ethiopia. *Preventive Veterinary Medicine*, *116*(1–2), 26–36. https://doi.org/10.1016/j.prevetmed.2014.06.004

Impacts estimated from case outbreaks of FMD in six districts in Ethiopia. Impact was estimated using a questionnaire administered to 512 cattle owners in a crop-livestock mixed system and a pastoral farming system.

- Foot and mouth disease morbidity rates of 85.2% and 94.9% at herd level; and 74.3% and 60.8% at animal level in the affected herds were determined for crop–livestock mixed system and pastoral system, respectively.
- The overall and calf specific mortality rates were 2.4% and 9.7% for the crop–livestock mixed system, and 0.7% and 2.6% for the pastoral system, respectively.
- The economic losses of foot and mouth disease outbreak due to milk loss, draft power loss and mortality were on average USD 76 per affected herd and USD 9.8 per head of cattle in the affected herds in crop–livestock mixed system; and USD 174 per affected herd and USD 5.3 per head of cattle in the affected herds in the pastoral system.
- The major loss due to the disease occurred as a result of milk losses and draft power losses whereas mortality losses were relatively low.

Jemberu, W. T., Mourits, M. C. M., Sahle, M., Siraw, B., Vernooij, J. C. M., & Hogeveen, H. (2016). Epidemiology of Foot and Mouth Disease in Ethiopia: a Retrospective Analysis of District Level Outbreaks, 2007-2012. Transboundary and Emerging Diseases, 63(6), e246–e259. https://doi.org/10.1111/tbed.12338

District level FMD outbreak data were collected from 115 randomly selected districts in Ethiopia using a questionnaire administered to district animal health officers.

- The national incidence of FMD outbreaks during the study period was 1.45 outbreaks per five district years. Outbreaks were geographically widespread affecting all major regional states in the country.

Negusssie, H., Kyule, M. N., Yami, M., Ayelet, G., & Jenberie T, S. (2011). Outbreak

investigations and genetic characterization of foot-and-mouth disease virus in Ethiopia in 2008/2009. *Tropical Animal Health and Production*, *43*(1), 235–243. https://doi.org/10.1007/s11250-010-9683-2

Outbreak investigation of eight FMD outbreaks in Ethiopia in 2008/ 2009.

- Of a total of 7,781 cattle observed and recorded in six districts, 1,409 (19.6%) were infected, and 15 (0.12%) died during outbreaks of FMD.
- Epidemiological investigations revealed that the morbidity rate of the disease was 21.1% in Akaki-kality sub-city, but the mortality rate was <2% in all districts.
- The mortality and case fatality rates were relatively higher, 1.6% and 8.9% in calves than the other age groups, respectively.

Abdela, N. (2017). Sero-prevalence, risk factors and distribution of foot and mouth disease in Ethiopia. *Acta Tropica*, *169*, 125–132. https://doi.org/10.1016/j.actatropica.2017.02.017

Review of seroprevalence reports of Foot and mouth disease in Ethiopia from 2004-2016.

- Seropositivity ranges from 6% to 43% in cattle and from 4% to 11% in small ruminants.
- In Ethiopia endemic distributions of five of seven serotypes, namely serotypes O, A, C, SAT1 and SAT2 have been documented. The dominant serotype being reported recently is serotype O and serotype C has not been reported in the country since 1983. However, serotype C specific antibody was detected in cattle indicating that circulation of serotype C viruses in the country may have gone unnoticed.

Kerfua, S. D., Shirima, G., Kusiluka, L., Ayebazibwe, C., Martin, E., Arinaitwe, E., Haydon, D. T. (2019). Low topotype diversity of recent foot-and-mouth disease virus serotypes O and A from districts located along the Uganda and Tanzania border. *Journal of Veterinary Science*, *20*(2), e4. https://doi.org/10.4142/jvs.2019.20.e4

Viral sequencing to determine the relationship between foot-and-mouth disease viruses circulating in districts along the Uganda and Tanzanian border between 2016 and 2017 and currently used vaccines.

- 8 out of the 11 sequences obtained belonged to serotype O and three belonged to serotype A. The serotype O sequences obtained showed limited nucleotide divergence (average of 4.9%) and belonged to topotype East Africa-2, whereas the most common O-type vaccine strain used in the region (O/KEN/77/78) belonged to East Africa-1.
- The vaccine strain K35/1980 belongs to Africa G VII with an average sequence divergence of 20.5% from the study sequences.
- The genetic distances between current vaccine strains and circulating field strains underscores the crucial need for regular vaccine matching.

Sulayeman, M., Dawo, F., Mammo, B., Gizaw, D., & Shegu, D. (2018). Isolation, molecular characterization and sero-prevalence study of foot-and-mouth disease virus circulating in central Ethiopia. *BMC Veterinary Research*, *14*(1), 110. https://doi.org/10.1186/s12917-018-1429-9

A cross-sectional study design was conducted from September 2015 to May 2016 to isolate and characterize FMD virus from outbreak cases of foot-and-mouth disease in central Ethiopia.

- 28.8% (n = 378) cattle showed signs and lesions suggestive of FMD and 34 samples were subjected to virus isolation.
- Three different serotypes (A, O and SAT 2) were responsible for the outbreaks of the disease.

Lyons, N. A., Alexander, N., Stärk, K. D. C., Dulu, T. D., Rushton, J., & Fine, P. E. M. (2015). Impact of foot-and-mouth disease on mastitis and culling on a large-scale dairy farm in Kenya. *Veterinary Research*, *46*(1), 41. https://doi.org/10.1186/s13567-015-0173-4

A cohort study on a FMD (virus serotype SAT2) outbreak on a large-scale dairy farm in Nakuru County, Kenya, to evaluate the impact of FMD on clinical mastitis and culling rate. A cohort of 644 cattle were followed for twelve months after the outbreak.

- 400/644 (62.1%) of cattle were reported to be affected with FMD due to serotype SAT2. 63/644 (9.78%) developed clinical mastitis.
- A univariable analysis showed no effect of FMD on rate of mastitis.
- There was stronger evidence of an increased rate of clinical mastitis in the first month after the onset of the outbreak.
- 76/644 (11.8%) animals were culled.

Coffin, J. L., Monje, F., Asiimwe-Karimu, G., Amuguni, H. J., & Odoch, T. (2015). A One Health, participatory epidemiology assessment of anthrax (Bacillus anthracis) management in Western Uganda. *Social Science & Medicine*, *129*, 44–50. https://doi.org/10.1016/j.socscimed.2014.07.037

Mixed-methods study to investigate impact of anthrax on farmer livelihood near a Queen Elizabeth National Park in Uganda where reported anthrax outbreaks (2004-2005 and 2010) killed over 500 wild animals and over 400 domestic animals. Data collection included 11 focus groups and 76 structured questionnaires in 7 villages

- Tick-borne diseases were ranked number one by farmers in terms of importance and Brucellosis was ranked number two. Although anthrax is perceived as costly, it is rare, which led participants to rank it as less important in livelihood success.

- Participants did mention livestock and human illness and deaths from anthrax, yet they ranked other impacts as more important. Depreciation of the value of the animal or products, cost of treatment, and particularly market closure were all emphasized.

Sijapenda, J. E., Komba, E. V. G., & Nonga, H. E. (2017). Studies on seroprevalence and risk factors for occurrence of bovine brucellosis in cattle in Lindi District, Tanzania. *Tanzania Veterinary Journal*, *35*(Special Issue), 82–89.

Cross-sectional survey measuring seroprevalence of brucellosis in 300 cattle and a questionnaire with 60 farmers.

- Eighty-five percent of farmers in the study were not aware of brucellosis.
- Proportions of positive reactors to brucellosis were 6.0% and 5.2% based on RBPT and c-ELISA respectively. Risk factors included higher age of cattle, history of abortion, and improper disposal of aborted material.

Kabi, F., Muwanika, V., & Masembe, C. (2015). Spatial distribution of *Brucella* antibodies with reference to indigenous cattle populations among contrasting agro-ecological zones of Uganda. *Preventive Veterinary Medicine*, *121*(1–2), 56–63. https://doi.org/10.1016/j.prevetmed.2015.06.007

Cross-sectional study designed to look at presence of *Brucella* antibodies in indigenous cattle across agro-ecological zones in Uganda. Sample of 925 indigenous breed cattle randomly selected from 209 herds.

- *Brucella* antibodies occurred in approximately 8.64% (80/925) and 28.70% (95% CI: 22.52, 34.89) of the sampled individual cattle and herds, respectively. Similar seroprevalence reported for indigenous cattle in Tanzania and Ethiopia.
- Recent incidences of abortions within the previous 12 months were significantly ($p< 0.001$) associated with seropositive herds.
- Identified potential brucellosis control solution of culling of older cattle (>72 months) and those which have had an abortion especially in the third trimester as may be more associated with brucellosis.

Madut, N. A., Muwonge, A., Nasinyama, G. W., Muma, J. B., Godfroid, J., Jubara, A. S., Kankya, C. (2018). The sero-prevalence of brucellosis in cattle and their herders in Bahr el Ghazal region, South Sudan. *PLOS Neglected Tropical Diseases*, *12*(6), e0006456. https://doi.org/10.1371/journal.pntd.0006456

Cross-sectional serosurvey of brucellosis in cattle and agro-pastoral herders using random sampling of cattle across four purposively chosen states in South Sudan. A total of 893 and 87 animal and human sera were examined.

- Bovine brucellosis prevalence was 31% (95%CI = 28.0-34.2), with the highest 63% (95%CI = 53-70) and lowest 10% (95%CI = 4.5-20.1) prevalence estimates in Wau and Gogrial states respectively
- The overall brucellosis sero-prevalence in herders was estimated at 33.3% (23.9-44.3).

Makita, K., Fèvre, E. M., Waiswa, C., Eisler, M. C., Thrusfield, M., & Welburn, S. C. (2011). Herd prevalence of bovine brucellosis and analysis of risk factors in cattle in urban and peri-urban areas of the Kampala economic zone, Uganda. *BMC Veterinary Research*, *7*(1), 60. https://doi.org/10.1186/1746-6148-7-60

A cross-sectional study to measure the prevalence of brucellosis in cattle and risk factors for the disease in urban and peri-urban areas of the Kampala economic zone within Uganda dairy farming systems. Sample size was 423 cows in 177 herds.

- The adjusted herd prevalence of brucellosis was 6.5% (11/177) and the adjusted individual animal prevalence was 5.0% (21/423) based on diagnosis using commercial kits of the competitive enzyme-linked immunosorbent assay (CELISA) for *Brucella abortus* antibodies.
- The study identified two risk factors for brucellosis at the herd level: large herd size and history of abortion.

Njeru, J., Wareth, G., Melzer, F., Henning, K., Pletz, M. W., Heller, R., & Neubauer, H. (2016). Systematic review of brucellosis in Kenya: disease frequency in humans and animals and risk factors for human infection. *BMC Public Health*, *16*(1), 853. https://doi.org/10.1186/s12889-016-3532-9

A systematic literature review of brucellosis in Kenya, disease frequency in humans and animals, and risk factors for human infection.

- Bacteriological evidence revealed the presence of *Brucella (B.) abortus* and *B. melitensis* in cattle and human patients, whilst *B. suis* was isolated from wild rodents only.
- High seroprevalence i.e., 9.9–16.9 % for cattle, 11.9 % for sheep, and 13.0–16.1 % for goats were found in pastoralist managed herds.
- Seroprevalences of 0.8–2.4 % in cattle, 2.4 % in sheep, and 0–1.3 % in goats were reported in herds managed from smallholder farms.
- Seroprevalence tended to be lower in agro-pastoralist herds than in pastoralist-managed herds.

Vhoko, K., Iannetti, S., Burumu, J., Ippoliti, C., Bhebhe, B., & De Massis, F. (2018). Estimating the prevalence of brucellosis in cattle in Zimbabwe from samples submitted to the

central veterinary laboratory between 2010 and 2014. *Veterinaria Italiana*, *54*(1), 21–27. https://doi.org/10.12834/VetIt.1111.6191.2

Longitudinal study of brucellosis prevalence using milk samples submitted to the central veterinary laboratory in Zimbabwe between 2010 and 2014 by 156 smallholder and commercial dairy farms (12,359 milk samples). Samples were tested with Rose Bengal Test (RBT), Complement Fixation Test (CFT) and Milk Ring Test (MRT).

- 31% (95% C.I.: 24% - 38%) of the farms tested were found positive (47/156).
- Underestimation of prevalence is possible because of the relatively low specificity of Milk Ring Test.

Edao, B. M., Hailegebreal, G., Berg, S., Zewude, A., Zeleke, Y., Sori, T., Wood, J. L. N. (2018). Brucellosis in the Addis Ababa dairy cattle: the myths and the realities. *BMC Veterinary Research*, *14*(1), 396. https://doi.org/10.1186/s12917-018-1709-4

- A cross-sectional study was conducted from November 2016 to May 2017 to estimate the prevalence and associated risk factors of bovine brucellosis and to assess knowledge-attitude and practices (KAP) of farm workers in Addis Ababa dairy farms. The serosurvey sample size was 1,550 cattle across 127 farms (103 small, 17 medium, and 7 large farms).
- More than 70% of medium and large farms and 43% of small sized farms had reproductive problems (abortion, stillbirth, retained fetal membrane and repeat breeding) in their farms.
- 43 of the 1550 tested cattle tested positive for brucellosis infection using serological tests.

Mugizi, D. R., Boqvist, S., Nasinyama, G. W., Waiswa, C., Ikwap, K., ROCK, K., Erume, J. (2015). Prevalence of and factors associated with Brucella sero-positivity in cattle in urban and peri-urban Gulu and Soroti towns of Uganda. *Journal of Veterinary Medical Science*, *77*(5), 557–564. https://doi.org/10.1292/jvms.14-0452

Cross-sectional epidemiological study to investigate the seroprevalence of Brucella and factors associated with *Brucella* seropositivity in smallholder-owned cattle in urban and peri-urban areas of Gulu and Soroti towns (Uganda). Sample size was 500 sera (from 116 cattle herds in Gulueg) and 507 sera (from 50 herds in Soroti town).

- Individual animal-level and herd-level seroprevalences were 7.5% (76/1007) and 27.1% (45/166) respectively.

- The study found an insignificant association between history of abortion and seropositivity, or retained placenta and seropositivity. This points to possible occurrence of other abortifacients and causes of retained placenta in cattle in the study areas.
- Large herd size was significantly associated with seropositivity, herds with >20 cattle were 7.8 times more likely to test sero-positive compared to those of 1–5 cattle.
- Ankole cattle kept in herds with more than 20 cattle were 3.3 times more likely to test seropositive compared to other breeds or their counterparts in smaller herds.

Okumu, T. A., John, N. M., Wabacha, J. K., Tsuma, V., & VanLeeuwen, J. (2019). Seroprevalence of antibodies for bovine viral diarrhoea virus, Brucella abortus and Neospora caninum, and their roles in the incidence of abortion/foetal loss in dairy cattle herds in Nakuru District, Kenya. *BMC Veterinary Research*, *15*(1), 95. https://doi.org/10.1186/s12917-019-1842-8

Cross-sectional serosurvey of 398 dairy cows in Nakuru district, Kenya and a 17 month longitudinal sero-epidemiological survey of 260 dairy cows on 64 farms to assess the seroprevalence of antibodies for bovine viral diarrhoea virus, Brucella abortus, and Neospora caninum, and their roles in the incidence of abortion/fetal loss.

- In the 398 randomly selected cattle on 64 dairy herds, the seroprevalences of antibodies to Bovine viral diarrhoea virus (BVDV), *Neospora caninum* (NC), and Brucella abortus (BA) were 79.1, 25.6 and 16.8%, respectively.
- Among 260 monitored pregnant dairy cattle on the same 64 dairy farms, an incidence risk for abortion of 10.8% (28/260) was identified, while the incidence of other foetal losses was 1.1% (3/260). *Neospora caninum* was associated with most cases (29.0%) of fetal losses, followed by mixed infections of NC and BVDV (12.9%), BVDV (9.9%) and co-infections of BA and NC (6.5%).
- The incidence rates of sero-conversion for NC, BVD and BA were 1.1, 0.06 and 0.5 new infections/100 cow-months at risk, respectively.
- Evicence of mixed/ co-infections: Of the cattle seropositive to NC, 83.3% were also seropositive to BVDV and 13.7% to BA. Of the cattle seropositive to BVDV, 17.1% were also seropositive to BA.

Seyoum, Z., Terefe, G., & Ashenafi, H. (2013). Farmers' perception of impacts of bovine trypanosomosis and tsetse fly in selected districts in Baro-Akobo and Gojeb river basins, Southwestern Ethiopia. *BMC Veterinary Research*, *9*(1), 214. https://doi.org/10.1186/1746-6148-9-214

In this study a standardized questionnaire survey was used to generate information on the knowledge and attitude of eighty-four agro-pastoralists from two districts on the presence,

impact and management of bovine trypanosomosis in Baro-Akobo and Gojeb river basins in Southern Nations National Peoples Regional State in Southwestern Ethiopia.

- Trypanosomosis (95.2%), fly strike/nuisance (66.7%), ectoparasite (tick) infestation (52.4%), pasteurellosis (42.9%), blackleg (35.7%) and internal parasites (33.3%) were most frequently reported diseases in that order.
- According to the respondents, among the 65 cattle deaths reported, trypanosomiasis accounted for 65% of the total annual deaths in the last 12 months of the year (2011/2012) and the mortality rate of cattle was 6.4%.
- Diseases, shortage of feed, improper grazing land management, lack of institutional support, inadequate veterinary services and market problem were most frequently reported constraints of cattle production in the study districts.
- Most livestock keepers 95.2% (92.9% in Gimbo and 97.6% in Guraferda) reported that they are familiar with bovine trypanosomosis (locally called "Gendi or Golebo"). Among these, 94.1% of the respondents listed bovine trypanosomosis as the prime economically important cattle disease in the river basins/districts.
- Overall, trypanocides 96.3% (79/82) followed by anthelmintics 67.1% (55/82), antibiotics 47% (39/82) and acaricide 35.4% (29/82) (to control ectoparasites) have been most commonly used drugs against cattle illness and ectoparasite infestation in the study districts.

Moti, Y., Deken, R. de, Thys, E., Abbeele, J. van den, Duchateau, L., & Delespaux, V. (2015). PCR and microsatellite analysis of diminazene aceturate resistance of bovine trypanosomes correlated to knowledge, attitude and practice of livestock keepers in South-Western Ethiopia. *Acta Tropica*, *146*, 45–52.

Longitudinal study over 6 months of 106 trypanosome positive bovines and 119 negative bovines in Ethiopia with goals of understanding resistance to diminazene aceturate (DA). Trypanosome positive bovines were treated with a single dose of DA, a double dose if relapsed, and with isometamidium chloride (ISM) if relapsed a third time.

- The main events were new infections (40.0%) and relapses (37.5%) with cures lagging at 22.5%, suggesting resistance to DA.
- Livestock keepers are focusing on sick animals and do not think in terms of prophylaxis. There was a trend to overdose young small animals and to under dose large ones. Oxen were treated very frequently (nearly 20 times/year) and calves almost never.
- Diminazene aceturate (DA) was used by 95.5% of the farmers the year preceding this survey. Species detection of trypanosomes showed 38 (59.4%) *Trypanosoma congolense* savannah, of which 31 (81.6%) showed a DA resistant profile.

- Diminazene aceturate (DA) resistance is a challenge to treating against AAT.
- Need for application of strategic vector control methods that consider drug resistance trends.

Simwango, M., Ngonyoka, A., Nnko, H. J., Salekwa, L. P., Ole-Neselle, M., Kimera, S. I., & Gwakisa, P. S. (2017). Molecular prevalence of trypanosome infections in cattle and tsetse flies in the Maasai Steppe, northern Tanzania. *Parasites & Vectors*, *10*(1), 507. https://doi.org/10.1186/s13071-017-2411-2

Cross-sectional, molecular prevalence study of trypanosome infections in cattle and tsetse flies in northern Tanzania. A total of 1,002 cattle and 886 tsetse flies were sampled.

- The overall prevalence of trypanosome infections was 17.2% in cattle and 3.4% in tsetse flies. *Trypanosoma vivax, T. brucei, T. simiae, T. theileri* and *T. congolense*, were present.
- No human-infective trypanosomes were detected in either cattle or tsetse fly DNA.

Magona, J. W., Walubengo, J., & Odimin, J. T. (2008). Acute haemorrhagic syndrome of bovine trypanosomosis in Uganda. *Acta Tropica*, *107*(2), 186–191. https://doi.org/10.1016/j.actatropica.2008.05.019

An observational and serosurvey was conducted to investigate an acute haemorrhagic syndrome causing mortality in cattle. Four hundred and one cattle belonging to 158 farmers were randomly sampled from a population of 549 and screened using a combination for trypanosomosis.

- Forty-nine cattle (12.2%) had trypanosome infection.
- The prevalence of bovine trypanosomosis in herds experiencing mortality (21.5%) was significantly higher than in those without mortality (2.6%) suggesting *T. vivax*-induced acute haemorrhagic syndrome.
- Out of an original population of 844 cattle, 295 (35%) died.

Kimaro, E. G., Toribio, J.-A. L. M. L. M. L., & Mor, S. M. (2017). Climate change and cattle vector-borne diseases: Use of participatory epidemiology to investigate experiences in pastoral communities in Northern Tanzania. *Preventive Veterinary Medicine*, *147*, 79–89. https://doi.org/10.1016/j.prevetmed.2017.08.010

Participatory epidemiology and focus groups of pastoralists in Northern Tanzania focusing on the epidemiology of ECF and AAT and climatic variables such as rainfall, drought, pasture availability, and vectors.

- Comparing 2014–1984, participant groups consistently reported declines in rainfall, vegetation cover and quality pasture, as well as increases in severe droughts. Experiences with ECF/AAT and vector abundance between these time periods was more

variable across villages, and likely relates to changes in climate and animal management practices over the last 30 years.

Weny, G., Okwee-Acai, J., Okech, S. G., Tumwine, G., Ndyanabo, S., Abigaba, S., & Goldberg, T. L. (2017). Prevalence and Risk Factors Associated with Hemoparasites in Cattle and Goats at the Edge of Kibale National Park, Western Uganda. *Journal of Parasitology*, *103*(1), 69–74. https://doi.org/10.1645/16-33

Cross-sectional study of 186 cattle and 317 goats from 40 pastoralist households in villages bordering Kibale National Park in Western Uganda area to assess the prevalence and risk factors associated with hemoparasites causing African Animal Trypanosomiasis, anaplasmosis, and theileriosis.

- Hemoparasite infection prevalence of 30.0% in cattle (12.9% in communities at the park edge and 3.7% in control communities).
- Prevalence of specific genera of hemoparasites in cattle was *Theileria*, 15.1%; *Trypanosoma*, 4.30%; and *Anaplasma*, 1.61%.
- Cattle were more likely than goats to be infected with *Theileria*, whereas goats were more likely than cattle to be infected with *Anaplasma.*
- Cattle in households bordering the park had higher prevalence of *Trypanosoma* than cattle from control villages ≥3 km from the park boundary, where no *Trypanosoma* infections were observed at all.
- *Theileria* infection in cattle were significantly higher in cross-bred cattle than in indigenous breeds.
- Wildlife–livestock interface is a high-risk area for hemoparasite infection.

Musinguzi, S., Suganuma, K., Asada, M., Laohasinnarong, D., Sivakumar, T., Yokoyama, N., Inoue, N. (2016). A PCR-based survey of animal African trypanosomosis and selected piroplasm parasites of cattle and goats in Zambia. *Journal of Veterinary Medical Science*, *78*(12), 1819–1824. https://doi.org/10.1292/jvms.16-0240

In this study, the prevalence of AAT and babesiosis in the three districts of Chama, Monze and Mumbwa in Zambia was determined using PCR-based methods. A total of 472 cattle from Chama (n=292), Mumbwa (n=96) and Monze (n=84) and 53 goats (all from Chama) were examined for the presence of animal African trypanosomes (*Trypanozoon, T. congolense and T. vivax), B. bigemina* and *T. parva*.

- Hemoparasites detected in cattle and goats were of the genera Theileria, Anaplasma, and Trypanosoma with a prevalence of 15.1%, 1.6%, and 4.3% respectively in cattle, and 10%, 6.0%, and 0.0%, respectively in goats.

- Although babesiosis is recognized as an economically important disease, unlike theileriosis and trypanosomiosis, its control and eradication have not been a priority.
- Results showed that goats can be an important reservoir for trypanosomes and that the treatment of goats could lead to a reduction in the incidence of trypanosomiosis in Zambia and other African countries.

Muraguri, G. R., McLeod, A., McDermott, J. J., & Taylor, N. (2005). The incidence of calf morbidity and mortality due to vector-borne infections in smallholder dairy farms in Kwale District, Kenya. *Veterinary Parasitology*, *130*(3–4), 305–315. https://doi.org/10.1016/j.vetpar.2004.11.026

The longitudinal study looked at morbidity and mortality caused by vector-borne infections of 137 pre-weaned calves in 67 farms (30,062 day risk period).

- The incidences of East Coast fever (ECF) (23.1%) and trypanosomosis (29.1%) were the highest among the vector-borne diseases. The annual incidence rates of anaplasmosis and babesiosis were 10.9 and 1.2%, respectively.
- The mortality incidence rates of ECF and trypanosomosis were 10.9 and 3.6%, respectively. There was no mortality arising specifically from anaplasmosis or babesiosis.

Gachohi, J. M., Ngumi, P. N., Kitala, P. M., & Skilton, R. A. (2010). Estimating seroprevalence and variation to four tick-borne infections and determination of associated risk factors in cattle under traditional mixed farming system in Mbeere District, Kenya. *Preventive Veterinary Medicine*, *95*(3–4), 208–223. https://doi.org/10.1016/j.prevetmed.2010.03.015

Cross-sectional, seroprevalence study of four tick-borne infections of cattle in smallholder households in Mbeere District, Kenya. A total of 440 cattle in 80 farms, selected by stratified random sampling from the four divisions in the district, were surveyed.

- Overall estimation of seroprevalences and their 95% confidence limits were: *T. parva* (19% [14%, 25%]), *T. mutans* (25% [20%, 29%]), *A. marginale* (58% [52%, 64%]) and *B. bigemina* (19% [15%, 23%]).

Vudriko, P., Okwee-Acai, J., Tayebwa, D. S., Byaruhanga, J., Kakooza, S., Wampande, E., Suzuki, H. (2016). Emergence of multi-acaricide resistant Rhipicephalus ticks and its implication on chemical tick control in Uganda. *Parasites & Vectors*, *9*(1), 4. https://doi.org/10.1186/s13071-015-1278-3

Cross-sectional study of emergence of multi-acaricide resistant *Rhipicephalus* ticks and its implication on chemical tick control in Uganda. Larval population tests conducted at 54 purposively selected small commercial farms from 17 districts, most with a history of acaricide failure.

- 93.5 % (29/31) of the larval population tested had resistance to at least one class of acaricide molecule; all of them belonging to the genus *Rhipicephalus*.
- *Rhipicephalus (Rhipicephalus) appendiculatus* and *Rhipicephalus (Boophilus) decoloratus* were the major (95.6 %) tick species in farms sampled.
- Resistance against synthetic pyrethroid (SP) was detected in 90.0 % (27/30) of the tick populations tested.
- Resistance was also detected against co-formulations of organophosphate and synthetic pyrethrois (43.3 %), organophosphate chlorfenvinphos (13.3 %), and amitraz (12.9 %).
- Multi-acaricide resistance (at least 2 molecules) was detected in 55.2 % (16/29) of the resistant *Rhipicephalus* ticks and significantly associated with *R. decoloratus.*
- This research is the first in Uganda to report emergence of super SP resistant and multi-acaricide resistant *R. appendiculatus* and *R. decoloratus* ticks.

Katale, B. Z., Mbugi, E. V, Karimuribo, E. D., Keyyu, J. D., Kendall, S., Kibiki, G. S., Matee, M. I. (2013). Prevalence and risk factors for infection of bovine tuberculosis in indigenous cattle in the Serengeti ecosystem, Tanzania. *BMC Veterinary Research*, *9*(1), 267. https://doi.org/10.1186/1746-6148-9-267

A cross sectional study to estimate the prevalence and risk factors associated with bovine tuberculosis transmission in indigenous cattle at the human-animal interface in the Serengeti ecosystem of Tanzania. Tuberculin skin test with 1,103 indigenous cattle from 32 herds and questionnaire administered to 108 pastoralist respondents.

- The apparent individual animal prevalence of tuberculin reactors was 2.4% (95% confidence interval (CI), 1.7 – 3.5%), whereas the true prevalence was 0.6% CI, 0.6 – 0.7% as indicated by a reaction to avian tuberculin purified protein derivatives (PPD) which is more than 4 mm greater than the reaction to avian tuberculin PPD.
- Tuberculin skin test results were found to be significantly associated with age, location, size of the household and animal tested.
- Of 108 respondents, 70 (65%) individuals had not heard about bovine tuberculosis at all. Thirty five percent (38/108) of respondents at least were aware of bovine tuberculosis. About 60% (23/38) of respondents who were aware of tuberculosis had some knowledge on how it is spread.

Romha, G., Gebru, G., Asefa, A., & Mamo, G. (2018). Epidemiology of Mycobacterium bovis and Mycobacterium tuberculosis in animals: Transmission dynamics and control challenges of zoonotic TB in Ethiopia. *Preventive Veterinary Medicine*, *158*, 1–17. https://doi.org/10.1016/j.prevetmed.2018.06.012

A systematic literature review of articles published from 1998-2017 focusing on tuberculosis in Ethiopian livestock.

- Prevalence of tuberculous lesion, ranging from 3.5 to 10.2%, have been reported in cattle slaughtered in different abattoirs.
- Both the prevalence and severity of pathology have been found higher in HF than in zebu cattle, and cattle managed intensively/kept in-door were more at risk than cattle kept in pasture.
- The main strain was found to be SB1176.

Kemal, J., Sibhat, B., Abraham, A., Terefe, Y., Tulu, K. T., Welay, K., & Getahun, N. (2019). Bovine tuberculosis in eastern Ethiopia: prevalence, risk factors and its public health importance. *BMC Infectious Diseases*, *19*(1), 39. https://doi.org/10.1186/s12879-018-3628-1

A cross-sectional study of bovine tuberculosis prevalence using an intradermal tuberculin skin test with 315 cows on 43 farms in eastern Ethiopia. A questionnaire was also administered to herd-owners. Multiple production systems were represented including smallholder and small commercial dairy.

- The overall prevalence of bovine tuberculosis was 20.3% (*n* = 64) in dairy cattle (positive intradermal tuberculin skin test). From a total of 43 farms/households tested, 22 were positive; each farm exhibited at least one tuberculin positive reactor animal with a total herd level prevalence of 51.2%.
- Only 33% had the knowledge of or had heard about bovine tuberculosis and 23% respondents were aware of the zoonotic importance of the disease.
- More than 50% of the interviewees prefered consuming raw milk to boiled milk.

Moiane, I., Machado, A., Santos, N., Nhambir, A., Inlamea, O., Hattendorf, J., Correia-Neves, M. (2014). Prevalence of Bovine Tuberculosis and Risk Factor Assessment in Cattle in Rural Livestock Areas of Govuro District in the Southeast of Mozambique. *PLoS ONE*, *9*(3), e91527. https://doi.org/10.1371/journal.pone.0091527

A cross-sectional study was conducted in Govuro district in southeast Mozambique to determine bovine tuberculosis prevalence in cattle and identify associated risk factors. The sample size was 1,136 cattle (125 from commercial production, 1,011 from smallholders).

- The overall apparent prevalence was estimated at 39.6%.

Molla, W., de Jong, M. C. M. M., Gari, G., & Frankena, K. (2017). Economic impact of lumpy skin disease and cost effectiveness of vaccination for the control of outbreaks in Ethiopia. *Preventive Veterinary Medicine*, *147*, 100–107.

https://doi.org/10.1016/j.prevetmed.2017.09.003

Questionnaire administered to 243 herd owners representing commercial dairy and smallholders from 15 districts in Ethiopia. Sampling strategy for selecting participants is not well-specified.

- 21% of cattle were reported to be infected (941 of 4430) with infected cattle in 82% of herds (200 of 243).
- *Assumptions:* Median loss of USD 375 (USD 325 in local Zebu and USD 1250 in Holstein-Friesian local Zebu cross cattle) was estimated per dead animal. Median losses per affected lactating cow were USD 141 (USD 63 in local Zebu cows and USD 216 in Holstein-Friesian local Zebu cross cows) and, USD 36 per affected ox. Diagnosis and medication cost per affected animal were estimated at USD 5.
- The median total economic loss of an LSD outbreak at herd level was USD 1176 (USD 489 in subsistence farm and USD 2735 in commercial farm). At herd level, the largest component of the economic loss was due to mortality (USD 1000) followed by milk loss (USD 120). LSD control costs were the least contributor to herd level losses. The financial analysis showed a positive net profit of USD 136 (USD 56 for subsistence farm herds and USD 283 for commercial herds) per herd due to LSD vaccine investment.

Ochwo, S., VanderWaal, K., Munsey, A., Ndekezi, C., Mwebe, R., Okurut, A. R. A., Mwiine, F. N. (2018). Spatial and temporal distribution of lumpy skin disease outbreaks in Uganda (2002–2016). *BMC Veterinary Research*, *14*(1), 174. https://doi.org/10.1186/s12917-018-1503-3

A retrospective study on the epidemiology of lumpy skin disease (LSD) in Uganda between the years 2002 and 2016, using data on reported outbreaks collected monthly by the central government veterinary administration.

- Morbidity, mortality, and case fatality rates were 4.77, 0.03 and 0.72%, respectively.
- 1,161 outbreaks and 319,355 cases of LSD were reported from 55 out of 56 districts of Uganda.
- Average of 77 (± 51.4 SD) outbreaks per year and a median of 70 outbreaks per year. During this 15-year period, 319,552 cases were recorded, with an average of 21,303 ± 4121 SD cases per year, and 2169 recorded deaths (average of 146 ± 17 SD deaths per year) attributed to LSD.
- The Central region reported the highest number of outbreaks (n = 418, 36%) followed by Eastern (n = 372, 32%), Southwestern (n = 140, 12%), Northern (n = 131, 11%), Northeastern (n = 37, 3%), Western (n = 41, 4%) and Northwestern (n = 22, 2%) regions.

- Outbreaks in endemic hotspots were less seasonal and had strikingly lower mortality and case-fatality rates than the other regions, suggesting an underlying difference in the epidemiology and impact of LSD in these different zones.

Gari, G., Bonnet, P., Roger, F., & Waret-Szkuta, A. (2011). Epidemiological aspects and financial impact of lumpy skin disease in Ethiopia. *Preventive Veterinary Medicine*, *102*(4), 274–283. https://doi.org/10.1016/j.prevetmed.2011.07.003

Cross-sectional questionnaire administered to cattle owners purposively selected "based on occurrence of Lumpy skin disease in their herd". Due to sampling strategy, incidence rates are likely skewed high. Sample size of local zebu owners versus Holstein Fresian / crossbreed cattle not given.

- The financial cost in infected herds was estimated to be USD 6.43 (5.12–8) per head for local zebu and USD 58 (42–73) per head for Holstein Fresian/crossbred cattle.

Swiswa, S., Masocha, M., Pfukenyi, D. M., Dhliwayo, S., & Chikerema, S. M. (2017). Long-term changes in the spatial distribution of lumpy skin disease hotspots in Zimbabwe. *Tropical Animal Health and Production*, *49*(1), 195–199. https://doi.org/10.1007/s11250-016-1180-9

A retrospective spatial analysis of twenty years of lumpy skin disease cases (1995-2014) in multiple production systems in Zimbabwe. The analyses identify hotspots, excess of reported cases in both space and time than would be expected to occur by chance and relate them to changing land use.

- Number of outbreaks is increasing and geographic distribution is increasing.
- From 1995–1999, there were only ten hotspots located mostly in the communal land in the northern, southern and south-eastern parts of the country. During the period 2000–2014, the number of significant hotspots increased from 115 in the second 5-year period to 187 in the last 5-year period. At the same time, the geographic distribution of hotspots expanded to other regions including the western areas of the country.

Gari, G., Waret-Szkuta, A., Grosbois, V., Jacquiet, P., & Roger, F. (2010). Risk factors associated with observed clinical lumpy skin disease in Ethiopia. *Epidemiology and Infection*, *138*(11), 1657–1666. https://doi.org/10.1017/S0950268810000506

Cross-sectional survey using a questionnaire administered to 330 agro-pastoral households across 15 districts in Ethiopia. Multi-stage sampling strategy, not clear whether households were randomly selected.

- Overall observed lumpy skin disease prevalence at animal-level was 8.1% (95% CI 7.3–8.9).
- Observed mortality was 2.12% (95% CI 1.73–2.6).

Mafirakureva, P., Saidi, B., & Mbanga, J. (2017). Incidence and molecular characterisation of lumpy skin disease virus in Zimbabwe using the P32 gene. *Tropical Animal Health and Production*, *49*(1), 47–54. https://doi.org/10.1007/s11250-016-1156-9

A two-year retrospective study of veterinary records of an LSD outbreak and molecular characterization of lumpy skin disease virus in Zimbabwe.

- Over the 2-year period, a total of 10,038 cases and 880 deaths (8.77 %) were recorded. LSD was reported from all regions of the country. Phylogenetic analyses showed that the viruses formed two major clusters implying that at least two strains of LSDV are in circulation in Zimbabwe.

Ayelet, G., Haftu, R., Jemberie, S., Belay, A., Gelaye, E., Sibhat, B., Asmare, K. (2014). Lumpy skin disease in cattle in central Ethiopia: outbreak investigation and isolation and molecular detection of the virus. *Revue Scientifique et Technique (International Office of Epizootics)*, *33*(3), 877–887.

A longitudinal study, combining two investigations into active outbreaks of lumpy skin disease (LSD) in cattle in central Ethiopia and a retrospective analysis of outbreak reports between January 2007 and December 2011 covering the entire country. Sample size was 2,174 cattle (1,992 from 40 commercial feedlots and 182 cattle from 40 smallholder farms).

- Retrospective analysis of data from January 2007 to December 2011 showed that 1,675 outbreaks of LSD were reported to the Ethiopian Ministry of Agriculture.
- Outbreaks were more frequently observed between September and December.
- In the active outbreak, morbidity and mortality rates of 13.61% (296) and 4.97% (108) were recorded.
- Morbidity rates were higher in young animals (18%) than in the adults (11.9%).
- Morbidity rate was higher in female animals (23.07%) than in the males (13.5%).
- Morbidity (15.1%) and mortality rates (5.37%) were higher in vaccinated animals than in unvaccinated animals, indicating vaccine failure.
- Morbidity (14.1%) and mortality (5.26%) rates were higher in feedlot cattle than in those extensively managed.

Kivaria, F. M., Noordhuizen, J. P. T. M., & Msami, H. M. (2007). Risk factors associated with the incidence rate of clinical mastitis in smallholder dairy cows in the Dar es Salaam region of Tanzania. *Veterinary Journal (London, England : 1997)*, *173*(3), 623–629.

https://doi.org/10.1016/j.tvjl.2006.01.009

Cross-sectional survey of 182 smallholder dairy cows from 62 herds in Dar es Salaam region of Tanzania.

- Clinical inspection indicated that 3.8% of the lactating cows had clinical mastitis. Subclinical mastitis was detected in 90.3% of lactating cows screened.
- Farm inspection revealed that water scarcity, barn size, residual suckling, single udder-towel and dairy labourers as the most substantial risk indicators.
- 5% of dairy workers were aware of the presence of subclinical mastitis.

Mekonnen, S. A. A., Koop, G., Melkie, S. T. T., Getahun, C. D. D., Hogeveen, H., & Lam, T. J. G. M. J. G. M. (2017). Prevalence of subclinical mastitis and associated risk factors at cow and herd level in dairy farms in North-West Ethiopia. *Preventive Veterinary Medicine*, *145*, 23–31. https://doi.org/10.1016/j.prevetmed.2017.06.009

Cross-sectional study of 167 urban and per-urban dairy farms with cross-breed herds in North-west Ethiopia.

- On average, 33% of the quarters and 62% of the cows were California Mastitis Test (CMT) positive, but the within herd quarter level prevalence ranged between 0 and 100%.
- A total of 1,543 milk samples, being 27 quarters that showed signs of CM, 606 CMT positive quarters and 910 CMT negative quarters were cultured, respectively 40%, 67% and 47% was positive on bacteriological culture.
- Coagulase negative staphylococci (CNS) (31%) followed by *Staphylococcus aureus* (9%) were the pathogens most frequently isolated.

Abebe, R., Hatiya, H., Abera, M., Megersa, B., & Asmare, K. (2016). Bovine mastitis: Prevalence, risk factors and isolation of Staphylococcus aureus in dairy herds at Hawassa milk shed, South Ethiopia. *BMC Veterinary Research*, *12*(1), 1–11. https://doi.org/10.1186/s12917-016-0905-3

Cross-sectional, prevalence study of mastitis in 529 lactating cows from 94 small to large dairy herds in South Ethiopia.

- The prevalence of mastitis at herd-level was 74.7% (95% CI: 64.5, 82.8). The corresponding cow-level prevalence was 62.6% (95% CI: 58.3, 66.7), of which 59.2 and 3.4% were sub-clinical and clinical mastitis cases, respectively.
- *S. aureus* was isolated from 51.2% of the milk samples cultured and 73.2% of the herds affected with mastitis.

Sitawa, R., Mbogoh, S. G., Gathuma, J. M., & Kairu, S. (2016). An evaluation of economic returns from east coast fever control through infection and treatment method at household level in Nandi and Uasin-Gishu counties of Kenya. *International Journal of Agricultural Policy and Research*, *4*(8), 149–156.

A cross-sectional study of 330 households with small-scale dairies in two counties in Kenya to look at the economic impact of adopting ECF vaccines.

- ECF-vaccinating households realized an overall net economic return of 44,575 Kenyan shillings (Kshs) (about US$ 450) per cow per year while the ECF non-vaccinating households realized a net loss of Kshs 9,975 (about US$ 100) per cow per year.
- Losses associated with ECF disease include being unable to market dairy products at the estimated market price, slaughtering of animals, and milk loss (on average 64% production loss).
- Gains associated with ECF vaccination include increased milk yield and reduced expenses on ECF treatment and related charges, particularly dipping (acaricide application).
- Study estimates that ECF non-vaccinated dairy animals are twice more likely to die from ECF.

Kerario, I. I., Simuunza, M. C., Chenyambuga, S. W., Koski, M., Hwang, S.-G., & Muleya, W. (2017). Prevalence and risk factors associated with Theileria parva infection in cattle in three regions of Tanzania. *Tropical Animal Health and Production*, *49*(8), 1613–1621. https://doi.org/10.1007/s11250-017-1367-8

Cross-sectional serosurvey for *Theileria parva* infection (East Coast fever) in 648 cattle randomly chosen across three purposively selected regions in Tanzania. Cattle from agro-pastoralists and pastoralists represented.

- The prevalence of *T. parva* across the three regions was 14.2%.
- There was high variation in tick burden and *T. parva* prevalence across the regions.

Malak, A. K., Mpoke, L., Banak, J., Muriuki, S., Skilton, R. A., Odongo, D., Kiara, H. (2012). Prevalence of livestock diseases and their impact on livelihoods in Central Equatorial State, southern Sudan. *Preventive Veterinary Medicine*, *104*(3–4), 216–223. https://doi.org/10.1016/j.prevetmed.2011.12.001

Seroprevalence and questionnaire administered to agro-pastoralists in two purposively selected counties in South Sudan reported to have a high burden of tick-borne disease. Some analyses done only for one county. Questionnaire covered small ruminants but seroprevalence was cattle only.

- Diseases ranked as important by farmers included diarrhoea in small ruminants, ECF, ticks (destructive in their own right rather than as vectors for TBDs), and a syndrome at various times consistent with heartwater, blackquarter, and contagious bovine pleuropneumonia or contagious caprine pleuropneumonia.
- Prevalence of *B. bovis* was found to be low (4.0% and 7.4% in Kajo Keji and Yei, respectively). 35% of the samples screened with the *T. parva* gene nested PCR assay were positive.
- For Kajo Keji country only: East Coast fever was estimated to have an annual relative incidence of 19.2% and a case fatality rate of 70%. Mortality due to ECF was estimated at 13.5%. Therefore, ECF was responsible for 26.7% and 34.6% of all morbidity and mortality, respectively.

Wanyoike, F., & Rich, K. M. (2010). An Assessment of the Regional and National Socio-Economic Impacts of the 2007 Rift Valley Fever Outbreak in Kenya. *The American Journal of Tropical Medicine and Hygiene*, *83*(2_Suppl), 52–57. https://doi.org/10.4269/ajtmh.2010.09-0291

The study assesses and quantifies the multi-dimensional socio-economic impacts of the 2007 Rift Valley Fever outbreak in Kenya based on a rapid assessment of livestock value chains in the northeast part of the country and a national macroeconomic analysis using social accounting methods. Social accounting methods are an input- output model in which economy-wide interactions between different economic sectors are modelled in an accounting framework. This allows supply and demand shocks to be considered and gives quantified impacts for different stakeholders, such as livestock producers (farmers) or butchers.

- The 2007 RVF outbreak in Kenya had wide-ranging impacts on the livestock sector and other segments of the economy that are often overlooked in the analysis of animal disease.
- The main negative effects on producers were caused by the loss of animals that died of RVF, which in turn had impacts on food security and future income (e.g., from the loss of future stock caused by animal abortions). Total economic losses from livestock mortality in these two regions were calculated at over US$9.3 million.
- Estimated that RVF-induced abortions in sheep caused a 22% reduction in potential flock sizes.
- Producers in Garissa District lost nearly 2.3 million liters in potential milk production valued at over US$77,000.
- Idling of the Garissa and Mwingi slaughterhouses resulted in economic losses of US$2,917 and an additional US$812 per month, respectively. This includes losses of the more than 100 households involved in tea sales, scrap sale, and cart transport of meat.

- As a result of the RVF outbreak, the value of total domestic supply in a variety of areas from petroleum to food staples to tourism fell by US$32 million. As one would expect, the bulk of the impacts were felt in the livestock sector. Interestingly, the value of other crops fell by over 0.5%, due in part to lower demand for feed crops. The SAM analysis further highlighted the aggregate losses faced by non- agricultural sectors, notably transportation, trade, chemicals, and petroleum. Shocks to the tourism sector, as proxied by the hotel sector, were relatively small on a percentage basis (less than 0.1%), but valued at over US$430,000.

Shabani, S. S., Ezekiel, M. J., Mohamed, M., & Moshiro, C. S. (2015). Knowledge, attitudes and practices on Rift Valley fever among agro pastoral communities in Kongwa and Kilombero districts, Tanzania. *BMC Infectious Diseases*, *15*(1), 363. https://doi.org/10.1186/s12879-015-1099-1

Cross-sectional survey focused on knowledge, attitudes and practices relating to Rift Valley fever among members of agropastoral communities in Kongwa and Kilombero districts, Tanzania. Community members (463) responded to a questionnaire.

- Ninety percent (90%) agreed that RVF posed a threat to public health as well as the local livestock economy (5 point Likert scale).
- Most of the respondents (97.6 %) had heard about RVF. Overall knowledge regarding RVF vector, modes of transmission, symptoms and prevention among respondents was low, with only 11.3% recorded as knowledgeable.

Nyakarahuka, L., de St. Maurice, A., Purpura, L., Ervin, E., Balinandi, S., Tumusiime, A., Shoemaker, T. R. (2018). Prevalence and risk factors of Rift Valley fever in humans and animals from Kabale district in Southwestern Uganda, 2016. *PLOS Neglected Tropical Diseases*, *12*(5), e0006412. https://doi.org/10.1371/journal.pntd.0006412

Following the diagnosis of two acute human Rift Valley fever cases in Kabale district, Uganda, serosurvey of 655 humans and 1,051 livestock (cattle, sheep, goats) were conducted to estimate RVFV seroprevalence in humans and livestock and to identify associated risk factors. Samples were collected at abattoirs and villages in Kabale district.

- The overall animal seropositivity was 13%, with 27% of cattle, 7% of goats, and 4% of sheep seropositive.
- 78% of humans tested positive for anti RVFV antibodies.

Nanyingi, M. O., Muchemi, G. M., Thumbi, S. M., Ade, F., Onyango, C. O., Kiama, S. G., & Bett, B. (2017). Seroepidemiological Survey of Rift Valley Fever Virus in Ruminants in Garissa,

Kenya. ***Vector-Borne and Zoonotic Diseases*****,** ***17*****(2), 141–146. https://doi.org/10.1089/vbz.2016.1988**

Cross-sectional serosurvey of 370 cattle, goats, and sheep for Rift Valley fever in eight purposively selected RVF-prone areas of Garissa county, Kenya.

- Sheep, cattle, and goats had seroprevalences of 32.2% (95% CI [20.6–31]), 33.3% (95% CI [6.7–60]), and 25.8% (95% CI [22.4–42]), respectively.

Hassan, O. A., Ahlm, C., Sang, R., & Evander, M. (2011). The 2007 Rift Valley Fever Outbreak in Sudan. ***PLoS Neglected Tropical Diseases*****,** ***5*****(9), e1229. https://doi.org/10.1371/journal.pntd.0001229**

The article summarizes the knowledge gathered on Rift Valley fever activity in Sudan focusing on the 2007 outbreak and emphasizes the essential role of the policy maker in the prevention and control of RVF.

- The 2007 outbreak was the first reported RVF outbreak in Sudan for 36 years, although studies indicate that the virus has been circulating in Sudan for a long time.
- In the outbreak, humans, rather than livestock, served as sentinels.

Onono, J. O., Wieland, B., & Rushton, J. (2014). Estimation of impact of contagious bovine pleuropneumonia on pastoralists in Kenya. ***Preventive Veterinary Medicine*****,** ***115*****(3–4), 122–129. https://doi.org/10.1016/j.prevetmed.2014.03.022**

Analysis of costs associated with CBPP using data available from previous studies in Kenya with goal of identifying a cost-effective control strategy.

- The impact of CBPP on pastoralists in Kenya is estimated at US$ 7.6 million per year. If all pastoralists whose cattle are at risk of infection adopt vaccination as a control strategy, the impact will only be US$ 3.3 million per year with savings amounting to US$ 4.3 million.

Kairu-Wanyoike, S. W., Kaitibie, S., Heffernan, C., Taylor, N. M., Gitau, G. K., Kiara, H., & McKeever, D. (2014). Willingness to pay for contagious bovine pleuropneumonia vaccination in Narok South District of Kenya. ***Preventive Veterinary Medicine*****,** ***115*****(3–4), 130–142. https://doi.org/10.1016/j.prevetmed.2014.03.028**

A conjoint cross-sectional contingent valuation study carried out with 190 smallholder households in Narok South District of Kenya to measure willingness to pay and demand for CBPP vaccine and vaccination as well as factors affecting willingness to pay.

- 87% of respondents had some knowledge of CBPP, 61.6% gave CBPP priority with regard to control.
- 70.0% saw a high likelihood of their cattle suffering CBPP.
- 54% of households had experienced adverse CBPP post-vaccination reactions in their herds.
- Only 34.4% of farmers were willing to pay an amount greater than zero for the current vaccine and vaccination. The remaining 65.6% were not willing to pay (zero and negative amount) and would need compensation to vaccinate their cattle against CBPP using the current vaccine.

Carlson, C. J., Kracalik, I. T., Ross, N., Alexander, K. A., Hugh-Jones, M. E., Fegan, M., Blackburn, J. K. (2019). The global distribution of Bacillus anthracis and associated anthrax risk to humans, livestock and wildlife. *Nature Microbiology*. https://doi.org/10.1038/s41564-019-0435-4

Global incidence database of human, wildlife, and livestock anthrax cases from multiple sources spanning 71 countries from 1914-2018.

- Using past occurrence as a proxy for risk, in Sub-Saharan Africa, an estimated 30.5 million cattle, 22.4 million goats, and 14.5 million sheep are at risk for anthrax.
- Vaccination rates in Sub-Saharan Africa are 0-6%.
- Vaccination may be less effective for the 31 million livestock and 4.6 million poor livestock keepers in West Africa, where an endemic lineage of *B. anthracis* shares an anthrose-deficiency mutation that has been hypothesized to lead to a vaccine escape.

Mohamed, S. B., Alagib, A., AbdElkareim, T. B., Hassan, M. M., Johnson, W. C., Hussein, H. E., Ueti, M. W. (2018). Molecular detection and characterization of Theileria spp. infecting cattle in Sennar State, Sudan. *Parasitology Research*, *117*(4), 1271–1276. https://doi.org/10.1007/s00436-018-5775-0

Cross-sectional serosurvey of 180 cattle from 11 sites in Sennar State, Sudan for molecular detection and characterization of Theileria species. The production system was not specified.

- Prevalence rate of hemoparasite infection was 63%.
- Nineteen of 44 animals were co-infected with more than one species of Theileria.
- Phylogenetic analysis revealed three Theileria spp. that were predominant in cattle including pathogenic *T. annulata* and apathogenic *T. velifera* and *T. mutans.*

Nyirenda, S. S., Sakala, M., Moonde, L., Kayesa, E., Fandamu, P., Banda, F., & Sinkala, Y. (2019). Prevalence of bovine fascioliasis and economic impact associated with liver condemnation in abattoirs in Mongu district of Zambia. *BMC Veterinary Research*, *15*(1),

(21 January 2019). https://doi.org/10.1186/s12917-019-1777-0

Abattoir study from 2013-2017 in Mongu district of Zambia measuring prevalence of bovine fascioliasis in 69,152 cattle carcasses and estimating economic impact of condemned liver meat.

- 64% of carcasses (44,511) were positive for adult *Fasciola* worms. According to the intensity of pathological lesions, 55.3% constituted severely affected livers, 30.3% were moderately affected livers and 14.4% were lightly affected livers.
- Most prevalent was *Fasciola gigantica* (56%) and it mostly affected animals with poor body condition (71%).
- The economic loss was estimated to be $3.60 USD/kg of liver multiplied by 164,600 kg condemned and destroyed between 2013 and 2017, for a total of $592,560 USD.

Assenga, J. A., Matemba, L. E., Muller, S. K., Mhamphi, G. G., & Kazwala, R. R. (2015). Predominant leptospiral serogroups circulating among humans, livestock and wildlife in Katavi-Rukwa ecosystem, Tanzania. *PLoS Neglected Tropical Diseases*, *9*(3), e0003607. https://doi.org/10.1371/journal.pntd.0003607

A cross-sectional epidemiological study, to determine the seroprevalence of *Leptospira spp* in humans, domestic ruminants and wildlife in the Katavi region, Southwest Tanzania. Sample size included 1,103 cattle, 248 goats, and 267 humans.

- The prevalence of leptospiral antibodies was 30% in cattle, 8% in goats, and 30% in humans.
- Study identified similar leptospiral serogroups circulating in humans, domestic ruminants, wildlife, rodents and shrews sharing the same ecosystem.

Schoonman, L., & Swai, E. S. (2010). Herd- and animal-level risk factors for bovine leptospirosis in Tanga region of Tanzania. *Tropical Animal Health and Production*, *42*(7), 1565–1572. https://doi.org/10.1007/s11250-010-9607-1

Cross-sectional serosurvey investigating bovine leptospirosis in Tanga, Tanzania. A sampling frame of 1,730 smallholder dairy and traditional herds, comprising 12,500 cattle, was used to randomly select 130 herds to participate in the study.

- Positive titres were detected in 30.3% [95% confidence intervals (CI) =26.7– 33.9] of cattle and 58.5% (95% CI=49.5–67.1) of herds, respectively.

Ndhlovu, D. N., & Masika, P. J. (2015). Risk factors associated with clinical dermatophilosis in smallholder sector cattle herds of Zimbabwe at the Amblyomma variegatum and Amblyomma hebraeum interface. *Tropical Animal Health and Production*, *47*(2), 353–

360. https://doi.org/10.1007/s11250-014-0727-x

A cross-sectional study was conducted to assess 185 smallholder cattle owners' awareness, perceptions, attitudes and drug-usage practices with regard to bovine dermatophilosis in north-west Zimbabwe.

- 45.4% of the herds were clinically positive for dermatophilosis.
- Concerns were raised by farmers about the effectiveness of these drugs against bovine dermatophilosis. 74.1% of the farmers treated their cattle using antibiotics. Tetracyclines were the antibiotics used by most farmers (79.3%), with 19.1% using penicillin.
- A small proportion (12.4%) of the farmers regarded bovine dermatophilosis as a potentially zoonotic disease.
- Tick bites were perceived by farmers as a major (45.4%) cause of bovine dermatophilosis.

Thumbi, S. M., Njenga, M. K., Marsh, T. L., Noh, S., Otiang, E., Munyua, P., Palmer, G. H. (2015). Linking Human Health and Livestock Health : A "One-Health" Platform for Integrated Analysis of Human Health, Livestock Health, and Economic Welfare in Livestock Dependent Communities. *PLoS ONE*, *10*(3), 1–18. https://doi.org/10.1371/journal.pone.0120761

1500 rural households in Western Kenya were enrolled in a syndromic surveillance study covering human and animal syndromes. Data were collected bi-weekly.

- Of the illness cases reported among each livestock species, gastro-intestinal tract syndrome cases were the most common, comprising of 52%, 53%, and 34% of total cases in cattle, goats, and sheep respectively. For these three species, respiratory syndromes were the second most commonly observed.

Nonga, H. E., & Karimuribo, E. D. (2009). A retrospective survey of hydatidosis in livestock in Arusha, Tanzania, based on abattoir data during 2005 - 2007. *Tropical Animal Health and Production*, *41*(7), 1253–1257. https://doi.org/10.1007/s11250-009-9308-9

Retrospective study of hydatidosis based on abattoir data from 2005-2007 in Arusha, Tanzania. A total of 115,186 cattle and 99,401 sheep and goats were investigated for cystic echinococcosis (CE).

- 6.0% infection rate in goats/ sheep and 4.2% infection rate in cattle.
- No significant difference between infection rates in rainy and dry season.

Dorny, P., Phiri, I., Gabriel, S., Speybroeck, N., & Vercruysse, J. (2002). A sero-epidemiological

study of bovine cysticercosis in Zambia. *Veterinary Parasitology*, *104*(3), 211–215.

Cross-sectional seroprevalence survey of 628 cattle from smallholder, dairy, and commercial enterprises.

- 6.1% seroprevalence of cysticercosis. Cysticercosis was significantly more prevalent in feedlots and traditional farming systems than in dairy farms.
- Economic significance of this parasite may be considerable due to downgrading and condemnation of carcasses. Does not quantify within survey but cites estimated losses of US$ 3,300,000 per year in South African feedlots.

Wardrop, N. A., Thomas, L. F., Cook, E. A. J., de Glanville, W. A., Atkinson, P. M., Wamae, C. N., & Fèvre, E. M. (2016). The Sero-epidemiology of Coxiella burnetii in Humans and Cattle, Western Kenya: Evidence from a Cross-Sectional Study. *PLOS Neglected Tropical Diseases*, *10*(10), e0005032. https://doi.org/10.1371/journal.pntd.0005032

A cross-sectional serological survey with a clustered sampling design was carried out in both humans and cattle to analyse the correlation and spatial-cross correlation and assess links between cattle and human seroprevalence. The study was conducted in parts of Western and Nyanza provinces in western Kenya (following restructuring of administrative areas in Kenya, the area covered includes parts of Busia, Siaya and Bungoma counties). Serum samples were obtained from all participants and evidence of exposure to *C. burnetii* was assessed using antibody ELISA methods.

- Overall seroprevalence of *C. burnetii* was 2.5% in humans and 10.5% in cattle.
- No evidence of correlation between cattle and human seroprevalence either within households, or when incorporating spatial proximity to other households in the survey.
- Cattle obtained from market (as opposed to those bred in the homestead) and those residing in areas with lower precipitation levels had the highest seroprevalence.
- Results illustrate endemicity of *C. burnetii* in western Kenya, although prevalence is relatively low.

Getaw, A., Beyene, D., Ayana, D., Megersa, B., & Abunna, F. (2010). Hydatidosis: Prevalence and its economic importance in ruminants slaughtered at Adama municipal abattoir, Central Oromia, Ethiopia. *Acta Tropica*, *113*(3), 221–225. https://doi.org/10.1016/j.actatropica.2009.10.019

A cross-sectional study was conducted from November 2007 to April 2008 to estimate the prevalence of hydatidosis in ruminants slaughtered at Adama municipal abattoir in Ethiopia with the goal of estimating the annual economic loss due to condemnation of organs during

meat inspection. A total of 1,152 ruminants (852 cattle, 92 sheep and 208 goats) were inspected following slaughter.

- Hydatidosis was prevalent in 47% of cattle, 29% of sheep, and 7% of goats.
- In cattle, 326 (55%) of the lung, 219 (37%) of the liver, 21 (4%) of the spleen, 15 (3%) of the heart and 10 (2%) of the kidney were found to be infected with hydatid cysts.
- According to the retrospective data, a total of 107,333 cattle were slaughtered and during this period 13,519 of the liver, 18,304 of the lung, 1,142 of the kidneys, 537 of the hearts and 150 of the spleens were found to be infected with hydatidosis. The total annual economic loss incurred due to organ meat condemnation at meat inspection from hydatidosis in ruminants slaughtered at Adama municipal abattoir was estimated to be to $5,870 USD.

Ibrahim, A. M. E., Elfahal, A. M., & El Hussein, A. R. M. (2012). First report of Neospora caninum infection in cattle in Sudan. *Tropical Animal Health and Production*, *44*(4), 769–772. https://doi.org/10.1007/s11250-011-9963-5

Cross-sectional serosurvey of 262 dairy cows from 25 herds in Sudan.

- The prevalence rates of *N. caninum* antibodies in cattle were high both at herd level (44%) and at individual animal level (10.7%).
- This study represents the first serological evidence for presence of *N. caninum* infection in Sudan.

Gelaye, E., Mekonnen, G., Jenberie, S., & Ayelet, G. (2013). Detection of sheep-associated malignant catarrhal fever from clinical cases in Ethiopian cattle. *Revue Scientifique et Technique de l'OIE*, *32*(2), 851–856. https://doi.org/10.20506/rst.32.2.2211

Report of a diagnostic investigation cattle disease problem that occurred in the Arbe Gona district of southern Ethiopia. Samples were collected from recently deceased animals.

- First report of a diagnostic investigation resulting in the detection of ovine OvHV-2 in cattle and confirming the existence of sheep-associated malignant catarrhal fever in Ethiopia.
- Disease outbreak caused morbidity and mortality in 18 cattle out of a total of more than 2,000 kept by farmers in the village and surrounding area.

Pandey, G. S., Simulundu, E., Mwiinga, D., Samui, K. L., Mweene, A. S., Kajihara, M., Takada, A. (2017). Clinical and subclinical bovine leukemia virus infection in a dairy cattle herd in Zambia. *Archives of Virology*, *162*(4), 1051–1056. https://doi.org/10.1007/s00705-016-3205-0

Case report confirmed with PCR in dairy cattle in Zambia.

- Two clinical cases of EBL were confirmed in a dairy cattle herd in Zambia. This is the first report of confirmed EBL in Zambia.
- Economic impact is perceived to be minimal however BLV is likely underdiagnosed.
