# Supplementary material for: Prioritizing smallholder animal health needs in East Africa, West Africa, and South Asia using three approaches: Literature review, expert workshops, and practitioner surveys
Source: Prev Vet Med. 2021 Apr;189:105279. doi: 10.1016/j.prevetmed.2021.105279 (PMC8024747; doi:10.1016/j.prevetmed.2021.105279)
Supplement: Supplementary file 4 [file mmc4.pdf]

Smallholder Animal Health Needs Assessment
East Africa Poultry

GALVmed

August 5, 2019

Assembled by Zoë Campbell, Thembinkosi Ramuthivheli, Tom Osebe, and Paul Coleman

Meta-analysis summary

The literature review yielded 29 articles, 12 of which focused on impact, incidence, and prevalence, and are summarized below. The table below shows the number of articles in which each animal health concern appears in the literature about poultry in East Africa (many articles mention multiple concerns). Only one article was given a green ranking for focus on both incidence/ prevalence and impact. The summarized articles are in the bibliography within this document. The full list of articles considered in the East Africa meta-analysis is available as a separate document.

The most mentioned concerns are Newcastle disease (ND) and infectious bursal disease (IBD). Secondary concerns include fowl pox, ectoparasites, endoparasites, coccidiosis, and Marek's disease.

Animal health concern	Frequency of mention (meta-analysis)	Frequency of mention (impact articles)	Main topic of green rank article
Newcastle disease	16	10	1
IBD	10	3	0
Fowl pox	4	4	0
Ectoparasites	4	3	0
Endoparasites	4	3	0
Coccidiosis	4	3	0
Marek's disease	4	0	0
Chronic respiratory disease	3	3	0
Helminth	3	2	0

Pasteurellosis	3	3	0
Avian influenza	2	1	0
Fowl cholera	2	2	0
Fowl typhoid	2	2	0
Salmonellosis	2	2	0

Table of articles

Abbreviations:

ND – Newcastle disease

IBD – Infectious bursal disease (Gumboro)

CRD – Chronic respiratory disease

Note: Clicking on the article number links to an expanded summary of the article below. The "web" link connects to the abstract of the article online or to full text for open access articles.

#	Citation	Country	Study design	Pathogen/ disease	Findings
1<br>1<br>web	(Msami, 2007)	Tanzania	Country review, Commercial, Smallholder	ND<br>IBD<br>Fowl Pox<br>Marek's disease<br>Salmonellosis<br>Colibacillosis<br>Mycoplasmosis<br>Ectoparasites	**Incidence/ Prevalence:** The diseases most prevalent in commercial poultry farms are ND, infectious bursal disease (IBD/Gumboro disease), Fowl pox, Marek's disease, Salmonellosis, colibacillosis and mycoplasmosis. ND is the major constraint to local poultry development. Other diseases mentioned to limit local poultry development in order of importance were coughing/sneezing (Infectious coryza), fowl pox, mites, ticks, and fleas.<br>**Impact:** It is estimated that in Tanzania 30 - 80% of the 32,559,208 village chickens die annually from ND. Village chickens sell for around USD 2.00 to 3.00 per bird. At a 30% mortality rate the minimum direct annual cost of

					uncontrolled ND is 19.5 million USD. At 80% mortality, the maximum loss is in the order of 78.1 million USD.
2<br>2<br>web	(Campbell, Otieno, Shirima, Marsh, & Palmer, 2019)	Tanzania	Cross-sectional, Household survey, Willingness to pay activity, Smallholder, 535 households	ND	**Incidence:** 91% of households believed they had seen NDV in their flock at some time. 77% of all households reported mortality of a third or more of their chickens within the last six months. 89% of these households believed NDV was the cause of mortality (356/400 households).<br>**Perceptions:** The smallholder farmers have high willingness to pay for ND vaccines.
3<br>3<br>web	(Fentie, Abebe, & Kassa, 2013)	Ethiopia	Cross-sectional Questionnaire 180 households Smallholders	ND	**Perceptions:** Newcastle disease was acknowledged as the biggest constraint of family chicken production.<br>**Incidence/ Prevalence:** Poultry disease (46.2%) and predation (27.1%) were claimed to be predominant causes of chicken loss.
4<br>3<br>web	(P. Bessell et al., 2017)	Tanzania India Nepal	Intervention analysis, Questionnaire, Smallholder farmers, 394 households in Tanzania	Newcastle disease	**Impact:** Intervention increased vaccination rates for ND to over 75% uptake in all areas, reaching 98% in India. In all areas flock sizes doubled, the numbers of eggs that were set for hatching and that hatched increased by 25–50% and there was an increase in the frequency with which chicken meat was consumed and chickens were sold. The increases in production after increasing vaccination coverage reflect the high impact of ND in the project areas.
5<br>2<br>web	(Meskerem, 2017)	Ethiopia	Cross-sectional survey, Smallholders,	Newcastle disease	**Incidence/ Prevalence:** The overall frequency of diseases reported as the main health constraint affecting chicken production in the study areas were, Newcastle disease (64%), followed by gastro intestinal infection (34%),

			162 households in 5 regions		respiratory syndrome (22%), internal and external parasites (16%), coccidiosis (15%) and fowl pox (5%).
6 3 web	(Campbell, Marsh, Mpolya, Thumbi, & Palmer, 2018)	Tanzania	Cross-sectional, Household survey, Smallholder, 535 households	ND	**Perceptions:** 80% of households were aware of ND vaccines. 57% had previously vaccinated and 26% recently vaccinated. Knowing someone who vaccinated increased the odds of a household previously vaccinating. Larger flock size was also associated with higher odds of previous vaccination. Usage of traditional medicine decreased the odds of previously vaccination.
7 3 web	(Mubamba, Ramsay, Abolnik, Dautu, & Gummow, 2016)	Zambia	Retrospective epidemiological study using reports from 1989-2014	Newcastle disease	**Incidence/ Prevalence:** The median apparent annual ND incidence rate (the number of new cases per population at risk) for the study period 1989–2014 was calculated to be 0.48%. Livestock disease control programs implemented in this region through major economic policies that existed from 1989 to 2014 probably had little impact on ND trends. Furthermore, the ND control strategies implemented during this period seem to have failed to halt the spread of the disease from affected districts to those that were previously ND free.
8 2 web	(Sambo et al., 2015)	Ethiopia	Cross-sectional, Participatory rural appraisal methods, Focus groups, 41 smallholders and 30 small commercial producers	Newcastle disease, Diarrhoea, Chronic respiratory disease, Pasteurellosis, Fowl pox	**Impact**: Newcastle disease was the most frequently identified disease problem causing bird mortality for both types of farmers. Semi-intensive and backyard farmers believe that a number of other non-specific syndromes and specific pathogens are impacting chicken productivity and mortality, including diarrhoea, chronic respiratory diseases, pasteurellosis and fowl pox. Some potentially significant diseases, such as Marek's disease and infectious bursal disease, were not

					mentioned, although outbreaks of these are known to have occurred on commercial farms in Ethiopia, including in Debre Zeit, in recent years.
9<br>2<br>web	(Habte et al., 2017)	Ethiopia	Country review, Seroprevalence, Commercial, Smallholder	ND<br>IBD<br>Marek's disease<br>Fowl pox<br>Fowl typhoid<br>Fowl cholera<br>Coccidiosis<br>Endoparasites<br>Ectoparasites<br>Avian influenza<br>Infectious coryza<br>Colibacillosis<br>Mycoplasmosis	**Incidence/ Prevalence:** ND is primarily an epidemic disease in Ethiopia with outbreaks causing high mortality, but with low levels between disease outbreaks. Seroprevalence reports range from 0.5% (Jarso, n=615) to 64.1% (Amhara, 2015, n = 729).<br>IBD is endemic in most parts of Ethiopia. Authors report seroprevalence of 5.1% in Horro (n=604) and 2.1% in Jarso (n = 604), much lower than seroprevalences ranging from 51-94% from studies focused on outbreaks.<br>Marek's disease virus has not previously been reported in village chickens in Ethiopia. 20–30% of birds were seropositive indicating the disease is endemic and circulating in villages.<br>Fowl pox is endemic in poultry in Ethiopia, mainly observed in commercial farms and among exotic chickens kept under a scavenging system.<br>(Continued in full below.)
10<br>2<br>web	(Bettridge et al., 2014)	Ethiopia	Cross-sectional, Serosurvey, Smallholder, 1,056 chickens	Newcastle disease, Infectious Bursal Disease, Salmonellosis, Pasteurellosis, Marek's disease,	**Incidence/ Prevalence:** Overall prevalence: 3.6% (IBD – Infectious Bursal Disease); (86%) *Salmonella*; (69%) *Pasteurella*; 32% (MDV - Marek's disease virus).<br>Overall detection prevalence parasitic diseases: 17% (*Ascaridida*); 56% (*Eimeria spp*); 34% (lice).<br>Hyperkeratosis was seen in 41% of birds.

				Ectoparasites, Endoparasites	
11<br>2<br>web	(Komba et al., 2013)	Tanzania	Cross-sectional 252 chickens Smallholders	Coccidia Helminths	**Incidence/ Prevalence:** 222 chickens (87%) were infested with helminth species and 28 birds (11%) were infected with coccidia. 25 birds (10%) had conjoint infestations with helminth species and coccidia.
12<br>3<br>web	(Hutton, Bettridge, Christley, Habte, & Ganapathy, 2017)	Ethiopia	Cross-sectional, Commercial, Pathogen detection survey, 117 chickens	Infectious bronchitis, Respiratory disease	**Incidence/ Prevalence:** Variant infectious bronchitis virus (793B genotype), avian metapneumovirus subtype B and *Mycoplasma synoviae, Mycoplasma gallisepticum* were found to be present.

Article summaries

Msami, H. (2007). Poultry sector country review: Tanzania.

Country review of commercial and smallholder poultry in Tanzania.

- The diseases most prevalent in commercial poultry farms are ND, infectious bursal disease (IBD/Gumboro disease), Fowl pox, Marek's disease, Salmonellosis, colibacillosis and mycoplasmosis.
- ND is the major constraint to local poultry development.
- Other diseases mentioned to limit local poultry development in order of importance were coughing/sneezing (Infectious coryza), fowl pox, mites, ticks, and fleas (Msami, 2000). Experience in Tanzania has shown that other diseases become more important in the longer term after ND is controlled.
- It is estimated that in Tanzania 30 - 80% of the 32,559,208 village chickens die annually from ND. Village chickens sell for around USD 2.00 to 3.00 per bird. At a 30% mortality rate the minimum direct annual cost of uncontrolled ND is 19.5 million USD. At 80% mortality, the maximum loss is in the order of 78.1 million USD.

Campbell, Z. A., Otieno, L., Shirima, G. M., Marsh, T. L., & Palmer, G. H. (2019). Drivers of vaccination preferences to protect a low-value livestock resource: Willingness to pay for Newcastle disease vaccines by smallholder households. *Vaccine*, *37*(1), 11–18. https://doi.org/10.1016/j.vaccine.2018.11.058

A household survey with a contingent valuation activity administered to 535 smallholder households across six villages in Arusha, Singida, and Mbeya regions of Tanzania to learn about willingness to pay for Newcastle disease vaccines for indigenous/ local chickens.

- 91% of households believed they had seen NDV in their flock at some time.
- 77% of all households reported mortality of a third or more of their chickens within the last six months.
- 89% of these households believed NDV was the cause of mortality (356/400 households).
- The willingness to pay (WTP) estimate was 5853 Tanzanian shillings ($2.64) to vaccinate ten chickens given the vaccine was protective for a period of three months.
- This estimate is about twice the market price reported by households in the study areas suggesting chicken-owning households value and benefit from NDV vaccines, but face other barriers to vaccination.

Fentie, T., Abebe, B., & Kassa, T. (2013). Small-scale family poultry production in north Gondar: characteristics, productivity and constraints. *Livestock Research for Rural*

Development, *25*(161).

Cross-sectional study with questionnaire administered to 180 households across four districts in Ethiopia (sampling strategy not specified further).

- Newcastle disease was acknowledged as the biggest constraint of family chicken production.
- Poultry disease (46.2%) and predation (27.1%) were claimed to be predominant causes of chicken loss.

Bessell, P., Kushwaha, P., Mosha, R., Woolley, R., Al-Riyami, L., & Gammon, N. (2017). Assessing the impact of a novel strategy for delivering animal health interventions to smallholder farmers. *Preventive Veterinary Medicine*, *147*(August), 108–116. https://doi.org/10.1016/j.prevetmed.2017.08.022

Cross-sectional questionnaire administered to 384 smallholder households in Morogoro region of Tanzania to assess effect of an intervention to deliver Newcastle disease vaccines and sensitize farmers.

- Intervention increased vaccination rates for ND to over 75% uptake in all areas, reaching 98% in India. In all areas flock sizes doubled, the numbers of eggs that were set for hatching and that hatched increased by 25–50% and there was an increase in the frequency with which chicken meat was consumed and chickens were sold.
- The increases in production after increasing vaccination coverage reflect the high impact of ND in the project areas.

Meskerem, A. (2017). Major health constraints and ethno-veterinary practices of small scale and backyard chicken production in some selected regions of Ethiopia. *Revue De Medecine Veterinaire*, *168*(1–3), 63–71.

Questionnaire administered to 162 smallholder households in 5 regions of Ethiopia in a cross-sectional survey designed to learn about major health constraints in poultry production.

- The overall frequency of diseases reported as the main health constraint affecting chicken production in the study areas were, Newcastle disease (64%), followed by gastro intestinal infection (34%), respiratory syndrome (22%), internal and external parasites (16%), coccidiosis (15%) and fowl pox (5%).

Campbell, Z., Marsh, T., Mpolya, E., Thumbi, S., & Palmer, G. (2018). Newcastle disease vaccine adoption by smallholder households in Tanzania: Identifying determinants and barriers. *PLoS ONE*, *13*(10), e0206058. https://doi.org/10.1371/journal.pone.0206058

A household survey administered to 535 smallholder households across six villages in Arusha, Singida, and Mbeya regions of Tanzania to learn about adoption of Newcastle disease vaccines for indigenous/ local chickens.

- 80% of households were aware of ND vaccines.
- 57% had previously vaccinated and 26% recently vaccinated within four months.
- Knowing someone who vaccinated increased the odds of a household previously vaccinating.
- Larger flock size was associated with higher odds of previous vaccination.
- Usage of traditional medicine to treat or prevent ND decreased the odds of previously vaccination.

Mubamba, C., Ramsay, G., Abolnik, C., Dautu, G., & Gummow, B. (2016). A retrospective study and predictive modelling of Newcastle Disease trends among rural poultry of eastern Zambia. *Preventive Veterinary Medicine*, *133*, 97–107. https://doi.org/10.1016/j.prevetmed.2016.09.017

- The median apparent annual ND incidence rate (the number of new cases per population at risk) for the study period 1989–2014 was calculated to be 0.48%.
- Livestock disease control programs implemented in this region through major economic policies that existed from 1989 to 2014 probably had little impact on ND trends. Furthermore, the ND control strategies implemented during this period seem to have failed to halt the spread of the disease from affected districts to those that were previously ND free.

Sambo, E., Bettridge, J., Dessie, T., Amare, A., Habte, T., Wigley, P., & Christley, R. M. (2015). Participatory evaluation of chicken health and production constraints in Ethiopia. *Preventive Veterinary Medicine*, *118*(1), 117–127. https://doi.org/10.1016/j.prevetmed.2014.10.014

Study used participatory rural appraisal methods to investigate the constraints facing both backyard and semi-intensive chicken farmers in and around Debre Zeit, Ethiopia, with a focus on the disease problems, farmers' perceptions regarding disease risk factors and the biosecurity measures in place on these farms. Sample size of 41 smallholders and 30 small commercial producers.

- Newcastle disease was the most frequently identified disease problem causing bird mortality for both types of farmers.
- Semi-intensive and backyard farmers believe that a number of other non-specific syndromes and specific pathogens are impacting chicken productivity and mortality, including diarrhoea, chronic respiratory diseases, pasteurellosis and fowl pox.

- Some potentially significant diseases, such as Marek's disease and infectious bursal disease, were not mentioned, although outbreaks of these are known to have occurred on commercial farms in Ethiopia, including in Debre Zeit, in recent years.
- Key finding of the study was the limited access to veterinary services reported by both semi-intensive and backyard producers.
- Poultry vaccines were reported by some respondents to be only intermittently available and then only in inappropriate volumes.
- There was widespread use of ethnoveterinary medicine by both groups of producers, although their use appeared somewhat more common among backyard producers
- Poor quality and cost of feed was an important production constraint to farmers in this study, especially for semi-intensive producers.

Habte, T., Amare, A., Bettridge, J., Collins, M., Robert, C., & Wigley, P. (2017). ***Guide to chicken health and management in Ethiopia*****.**

Comprehensive report/ review hybrid with serosurvey results from a four-year project called Chicken Health for Development (CH4D) project. Sample size/ sampling strategy for serosurveys not always described fully but most findings described further in published articles.

- ND is primarily an epidemic disease in Ethiopia with outbreaks causing high mortality, but with low levels between disease outbreaks. Seroprevalence reports range from 0.5% (Jarso, n=615) to 64.1% (Amhara, 2015, n = 729).
- IBD is endemic in most parts of Ethiopia. Authors report seroprevalence of 5.1% in Horro (n=604) and 2.1% in Jarso (n = 604), much lower than seroprevalences ranging from 51-94% from studies focused on outbreaks.
- Marek's disease virus has not previously been reported in village chickens in Ethiopia. 20–30% of birds were seropositive indicating the disease is endemic and circulating in villages.
- Fowl pox is endemic in poultry in Ethiopia, mainly observed in commercial farms and among exotic chickens kept under a scavenging system.
- Fowl typhoid: Seroprevalence rates of between 75–90% for Salmonella; much higher than rates of around 40% previously recorded. A small number of birds had very high antibody levels which may reflect carrier birds within flocks.
- Fowl cholera: Seroprevalence rates of between 80–90% for Pasteurella - higher than rates of around 65% previously recorded. This suggests Fowl cholera is a larger endemic problem than reported and the acute form may contribute to mortality.
- Coccidiosis: *Eimeria* oocysts (eggs) found in around 55% of faecal samples examined. All seven *Eimeria* species were found, in some cases more than one in an individual bird. Generally, the numbers of eggs were low. In most cases, we believe *Eimeria* is a problem leading to reduced productivity and increased susceptibility to other infections.

- Endoparasites: Both ascarid worm and tapeworm eggs were identified on faecal examination, with ascarid eggs detected in 17% and tapeworm eggs detected in 23% of the birds sampled.
- Ectoparasites: We identified a large selection of ectoparasites in Ethiopian village poultry, in particular, chewing lice and Scaly leg mite, with 60% of birds examined infested with one or both. Thirteen species of ectoparasite were identified, with many known to cause morbidity and mortality in flocks, particularly in young birds. Stick tight fleas were common in lowland areas with warmer climates, and body lice and scaly leg mites common to all regions. Farmers in all the regions studied reported ectoparasites to be a significant disease concern.
- Other diseases of concern include avian influenza and bacterial diseases including infectious coryza, colibacillosis caused by *E. coli*, and mycoplasmosis.

Bettridge, J. M., Lynch, S. E., Brena, M. C., Melese, K., Dessie, T., Terfa, Z. G., Christley, R. M. (2014). Infection-interactions in Ethiopian village chickens. *Preventive Veterinary Medicine*, *117*(2), 358–366. https://doi.org/10.1016/j.prevetmed.2014.07.002

Cross-sectional study investigated the epidemiology and ecology of co-infection with a range of pathogens in Ethiopian backyard chickens, with the main objectives being (i) to assess patterns of coinfection, and (ii) to identify common risk factors for co-infections. Sample size was 1,280 chickens from smallholder farmers with 1,056 chickens with complete data for all laboratory tests.

- Overall prevalence: 3.6% (IBD – Infectious Bursal Disease); (86%) *Salmonella*; (69%) *Pasteurella*; 32% (MDV - Marek's disease virus).
- Overall detection prevalence parasitic diseases: 17% (*Ascaridida*); 56% (*Eimeria spp*); 34% (lice).
- Hyperkeratosis was seen in 41% of birds; identification of hyperkeratosis is strongly associated with a positive identification of scaly leg mites from a skin scrape.
- Positive relationships were identified between *Pasteurella* and *Salmonella* titres; and between Marek's disease and parasitic infections, and these two groups of diseases were correlated with females and males, respectively.

Komba, E. V. G., Mkupasi, E. M., Mwesiga, G. K., Mbyuzi, A. O., Busagwe, Z., Mzula, A., Nzalawahe, J. (2013). Occurrence of helminths and coccidia in apparently healthy free range local chickens slaughtered at Morogoro live bird market. *Tanzania Veterinary Journal*, *28*(2), 55–61.

A cross-sectional convenience sample of 252 was taken of apparently healthy chickens at a live meat market in Tanzania. Light microscopy was used to detect coccidian oocysts.

- Two-hundred twenty two chickens (87%) were infested with helminth species and 28 birds (11%) were infected with coccidia. Twenty-five birds (10%) had conjoint infestations with helminth and coccidia.
- Nine helminth species were reported.
- Helminths and coccidia are common and pose health problems in free range local chickens.

Hutton, S., Bettridge, J., Christley, R., Habte, T., & Ganapathy, K. (2017). Detection of infectious bronchitis virus 793B, avian metapneumovirus, Mycoplasma gallisepticum and Mycoplasma synoviae in poultry in Ethiopia. *Tropical Animal Health and Production*, *49*(2), 317–322. https://doi.org/10.1007/s11250-016-1195-2

A survey was conducted into respiratory infectious diseases of poultry on a chicken breeder farm located in Debre Zeit, Ethiopia. Oropharyngeal swabs were collected from 117 randomly selected birds, and blood was taken from a subset of 73 of these birds. Serological and molecular methods was used for detection of pathogens.

- Variant infectious bronchitis virus (793B genotype), avian metapneumovirus subtype B and *Mycoplasma synoviae, Mycoplasma gallisepticum* were found to be present.
