# Supplementary material for: Prioritizing smallholder animal health needs in East Africa, West Africa, and South Asia using three approaches: Literature review, expert workshops, and practitioner surveys
Source: Prev Vet Med. 2021 Apr;189:105279. doi: 10.1016/j.prevetmed.2021.105279 (PMC8024747; doi:10.1016/j.prevetmed.2021.105279)
Supplement: Supplementary file 5 [file mmc5.pdf]

East Africa References

The references below were used for meta-analyses for all livestock types (cattle, small ruminants, and poultry). The sub-set of summarized articles are also included.

Abbas, B., Yuusif, M. A., & Nur, H. M. (2014). Animal health constraints to livestock exports from the Horn of Africa. *Revue Scientifique et Technique de l'OIE*, *33*(3), 711–721. https://doi.org/10.20506/rst.33.3.2314

Abdela, N. (2017). Sero-prevalence, risk factors and distribution of foot and mouth disease in Ethiopia. *Acta Tropica*, *169*, 125–132. https://doi.org/10.1016/j.actatropica.2017.02.017

Abdilatif, M. H., Onono, J. O., & Mutua, F. K. (2018). Analysis of pastoralists' perception on challenges and opportunities for sheep and goat production in Northern Kenya. *Tropical Animal Health and Production*, *50*(7), 1701–1710. https://doi.org/10.1007/s11250-018-1613-8

Abebe, R., Hatiya, H., Abera, M., Megersa, B., & Asmare, K. (2016). Bovine mastitis: Prevalence, risk factors and isolation of Staphylococcus aureus in dairy herds at Hawassa milk shed, South Ethiopia. *BMC Veterinary Research*, *12*(1), 1–11. https://doi.org/10.1186/s12917-016-0905-3

Abera, T., Bitew, M., Gebre, D., Mamo, Y., Deneke, Y., & Nandi, S. (2018). Bluetongue disease in small ruminants in south western Ethiopia: cross-sectional sero-epidemiological study. *BMC Research Notes*, *11*(1), 112. https://doi.org/10.1186/s13104-018-3222-z

Abera, Z., Degefu, H., Gari, G., & Kidane, M. (2015). Sero-prevalence of lumpy skin disease in selected districts of West Wollega zone, Ethiopia. *BMC Veterinary Research*, *11*(1), 135. https://doi.org/10.1186/s12917-015-0432-7

Abunna, F., Tilahun, G., Megersa, B., Regassa, A., & Kumsa, B. (2008). Bovine cysticercosis in cattle slaughtered at Awassa municipal abattoir, Ethiopia: prevalence, cyst viability, distribution and its public health implication. *Zoonoses and Public Health*, *55*(2), 82–88. https://doi.org/10.1111/j.1863-2378.2007.01091.x

Adam, I. A., Abdalla, M. A., Mohamed, M. E., & Aradaib, I. E. (2014). Prevalence of bluetongue virus infection and associated risk factors among cattle in North Kordufan State, Western Sudan. *BMC Veterinary Research*, *10*(1), 94. https://doi.org/10.1186/1746-6148-10-94

Addy, F., Alakonya, A., Wamae, N., Magambo, J., Mbae, C., Mulinge, E., Romig, T. (2012). Prevalence and diversity of cystic echinococcosis in livestock in Maasailand, Kenya. *Parasitology Research*, *111*(6), 2289–2294. https://doi.org/10.1007/s00436-012-3082-8

Agegnehu, A., Bogale, B., Tesfaye, S., & Dagnachew, S. (2018). Status of mange infestation in indigenous sheep and goats and their control practices in Wag-Himra zone, Ethiopia. *Journal of Veterinary Medicine and Animal Health*, *10*(5), 128–134.

Ahmadu, B., Lovelace, C. E. A., Samui, K. L., & Mahan, S. (2004). Some observations on the sero-prevalence of heartwater and tick infestation in Zambian goats. *The Onderstepoort Journal of Veterinary Research*, *71*(2), 161–164.

Alemayehu, G., Leta, S., & Hailu, B. (2015). Sero-prevalence of Contagious Bovine Pleuropneumonia (CBPP) in bulls originated from Borena pastoral area of Southern Ethiopia. *Tropical Animal Health and Production*, *47*(5), 983–987. https://doi.org/10.1007/s11250-015-0820-9

Alemu, B., Gari, G., Libeau, G., Kwiatek, O., Kidane, M., Belayneh, R., Abdi, R. D. (2019). Molecular detection and phylogenetic analysis of Peste des petits ruminants virus circulating in small ruminants in eastern Amhara region, Ethiopia. *BMC Veterinary Research*, *15*(1), 84. https://doi.org/10.1186/s12917-019-1828-6

Alingu, R. A., Muhanguzi, D., MacLeod, E., Waiswa, C., & Fyfe, J. (2014). Bovine trypanosome species prevalence and farmers' trypanosomiasis control methods in south-western Uganda. *Journal of the South African Veterinary Association*, *85*(1). https://doi.org/10.4102/jsava.v85i1.1094

Allan, K. J., Halliday, J. E. B., Moseley, M., Carter, R. W., Ahmed, A., Goris, M. G. A., Cleaveland, S. (2018). Assessment of animal hosts of pathogenic Leptospira in northern Tanzania. *PLOS Neglected Tropical Diseases*, *12*(6), e0006444. https://doi.org/10.1371/journal.pntd.0006444

Allepuz, A., Stevenson, M., Kivaria, F., Berkvens, D., Casal, J., & Picado, A. (2015). Risk Factors for Foot-and-Mouth Disease in Tanzania, 2001-2006. *Transboundary and Emerging Diseases*, *62*(2), 127–136. https://doi.org/10.1111/tbed.12087

Almaw, G., DUGUMA, M., Wubetie, A., Tuli, G., & Koran, T. (2016). A contagious bovine pleuropneumonia outbreak on a research farm in Ethiopia, and its dynamics over an eight-month period. *Revue Scientifique et Technique de l'OIE*, *35*(3), 787–793. https://doi.org/10.20506/rst.35.3.2569

Almaw, G., Zerihun, A., & Asfaw, Y. (2008). Bovine mastitis and its association with selected risk factors in smallholder dairy farms in and around Bahir Dar, Ethiopia. *Tropical Animal Health and Production*, *40*(6), 427–432. https://doi.org/10.1007/s11250-007-9115-0

Ameni, G., & Erkihun, A. (2007). Bovine tuberculosis on small-scale dairy farms in Adama Town, central Ethiopia, and farmer awareness of the disease. *Revue Scientifique et Technique (International Office of Epizootics)*, *26*(3), 711–719.

Ameni, G., Tadesse, K., Hailu, E., Deresse, Y., Medhin, G., Aseffa, A., Berg, S. (2013). Transmission of Mycobacterium tuberculosis between Farmers and Cattle in Central Ethiopia. *PLoS ONE*, *8*(10), e76891. https://doi.org/10.1371/journal.pone.0076891

Ameni, G., Tafess, K., Zewde, A., Eguale, T., Tilahun, M., Hailu, T., Vordermeier, H. M. (2018). Vaccination of calves with Mycobacterium bovis Bacillus Calmette-Guerin reduces the frequency and severity of lesions of bovine tuberculosis under a natural transmission setting in Ethiopia. *Transboundary and Emerging Diseases*, *65*(1), 96–104. https://doi.org/10.1111/tbed.12618

Amenu, K., Szonyi, B., Grace, D., & Wieland, B. (2017). Important knowledge gaps among pastoralists on causes and treatment of udder health problems in livestock in southern Ethiopia: results of qualitative investigation. *BMC Veterinary Research*, *13*(1), 303. https://doi.org/10.1186/s12917-017-1222-1

Angwech, H., Nyeko, J. H. P., Opiyo, E. A., Okello-Onen, J., Opiro, R., Echodu, R., Skilton, R. A. (2015). Heterogeneity in the prevalence and intensity of bovine trypanosomiasis in the districts of Amuru and Nwoya, Northern Uganda. *BMC Veterinary Research*, *11*(1), 255. https://doi.org/10.1186/s12917-015-0567-6

Anyango, G., Mutua, F., Kagera, I., Andang'O, P., Grace, D., & Lindahl, J. F. (2018). A survey of aflatoxin M1 contamination in raw milk produced in urban and peri-urban areas of Kisumu County, Kenya. *Infection Ecology & Epidemiology Epidemiology*, *8*, 1547094.

Asakura, S., Makingi, G., Kazwala, R., & Makita, K. (2018). Brucellosis Risk in Urban and Agro-pastoral Areas in Tanzania. *EcoHealth*, *15*(1), 41–51. https://doi.org/10.1007/s10393-017-1308-z

Asakura, S., Makingi, G., Kazwala, R., & Makita, K. (2018). Herd-level risk factors associated with Brucella sero-positivity in cattle, and perception and behaviours on the disease control among agro-pastoralists in Tanzania. *Acta Tropica*, *187*, 99–107. https://doi.org/10.1016/j.actatropica.2018.07.010

Ashenafi, F., Teshale, S., Agga, G., Fikru, R., & Laikemariam, Y. (2016). Distribution of brucellosis among small ruminants in the pastoral region of Afar, eastern Ethiopia. *Revue Scientifique et Technique de l'OIE*, *26*(3), 731–739. https://doi.org/10.20506/rst.26.3.1781

Asmare, K., Megersa, B., Denbarga, Y., Abebe, G., Taye, A., Bekele, J., Skjerve, E. (2013). A study on seroprevalence of caprine brucellosis under three livestock production systems in southern and central Ethiopia. *Tropical Animal Health and Production*, *45*(2), 555–560. https://doi.org/10.1007/s11250-012-0258-2

Asmare, K., Sibhat, B., Ayelet, G., Gebremedhin, E. Z., Lidete, K. A., & Skjerve, E. (2018). Serological evidence of Bovine herpesvirus-1, Bovine Viral Diarrhea virus and Schmallenberg v irus infections in relation to reproductive disorders in dairy cattle in Ethiopia. *Acta Tropica*, *178*, 236–241. https://doi.org/10.1016/j.actatropica.2017.12.005

Assefa, G. A., & Kelkay, M. Z. (2018). Goat pasteurellosis: serological analysis of circulating Pasteurella serotypes in Tanqua Aberegelle and Kola Tembien Districts, Northern Ethiopia. *BMC Research Notes*, *11*(1), 485. https://doi.org/10.1186/s13104-018-3606-0

Assefa, H., Mulate, B., Nazir, S., & Alemayehu, A. (2015). Cystic echinococcosis amongst small ruminants and humans in central Ethiopia. *The Onderstepoort Journal of Veterinary Research*, *82*(1), E1-7. https://doi.org/10.4102/ojvr.v82i1.949

Assenga, J. A., Matemba, L. E., Muller, S. K., Mhamphi, G. G., & Kazwala, R. R. (2015). Predominant leptospiral serogroups circulating among humans, livestock and wildlife in Katavi-Rukwa ecosystem, Tanzania. *PLoS Neglected Tropical Diseases*, *9*(3), e0003607. https://doi.org/10.1371/journal.pntd.0003607

Awad, H., Al-Hamidhi, S., El Hussein, A.-R. M., Yousif, Y. M. Z., Taha, K. M., Salih, D. A., Babiker, H. A. (2018). Theileria lestoquardi in Sudan is highly diverse and genetically distinct from that in Oman. *Infection, Genetics and Evolution*, *62*, 46–52. https://doi.org/10.1016/j.meegid.2018.04.014

Ayebazibwe, C., Tjørnehøj, K., Mwiine, F. N., Muwanika, V. B., Okurut, A. R. A., Siegismund, H. R., … Alexandersen, S. (2010). Patterns, risk factors and characteristics of reported and perceived foot-and-mouth disease (FMD) in Uganda. *Tropical Animal Health and Production*, *42*(7), 1547–1559. https://doi.org/10.1007/s11250-010-9605-3

Ayelet, G., Haftu, R., Jemberie, S., Belay, A., Gelaye, E., Sibhat, B., Asmare, K. (2014). Lumpy skin disease in cattle in central Ethiopia: outbreak investigation and isolation and molecular detection of the virus. *Revue Scientifique et Technique (International Office of Epizootics)*, *33*(3), 877–887.

Ayelet, G., Mahapatra, M., Gelaye, E., Egziabher, B. G., Rufeal, T., Sahle, M., Knowles, N. J. (2009). Genetic characterization of foot-and-mouth disease viruses, Ethiopia, 1981-2007. *Emerging Infectious Diseases*, *15*(9), 1409–1417. https://doi.org/10.3201/eid1509.090091

Baluka, S. A. (2016). Economic effects of foot and mouth disease outbreaks along the cattle marketing chain in Uganda. *Veterinary World*, *9*(6), 544–553.

Bessell, P. R., Sargison, N. D., Mirende, K., Dash, R., Prasad, S., Al-Riyami, L., Wambura, P. (2018). The impact of anthelmintic drugs on weight gain of smallholder goats in subtropical regions. *Preventive Veterinary Medicine*, *159*, 72–81. https://doi.org/10.1016/j.prevetmed.2018.08.014

Bessell, P., Kushwaha, P., Mosha, R., Woolley, R., Al-Riyami, L., & Gammon, N. (2017). Assessing the impact of a novel strategy for delivering animal health interventions to smallholder farmers. *Preventive Veterinary Medicine*, *147*(August), 108–116. https://doi.org/10.1016/j.prevetmed.2017.08.022

Bett, B., Jost, C., Allport, R., & Mariner, J. (2009). Using participatory epidemiological techniques to estimate the relative incidence and impact on livelihoods of livestock diseases amongst nomadic pastoralists in Turkana South District, Kenya. *Preventive Veterinary Medicine*, *90*(3–4), 194–203. https://doi.org/10.1016/j.prevetmed.2009.05.001

Bett, B., Mariner, J. C., Kihu, S., Swai, E. S., Jost, C. C., Njogu, G., Mariner, J. C. (2010). Epidemiological assessment of the Rift Valley fever outbreak in Kenya and Tanzania in 2006 and 2007. *The American Journal of Tropical Medicine and Hygiene*, *83*(2_Suppl), 65–72. https://doi.org/10.4269/ajtmh.2010.09-0290

Bettridge, J. M., Lynch, S. E., Brena, M. C., Melese, K., Dessie, T., Terfa, Z. G., Christley, R. M. (2014). Infection-interactions in Ethiopian village chickens. *Preventive Veterinary Medicine*, *117*(2), 358–366. https://doi.org/10.1016/j.prevetmed.2014.07.002

Beyene, T. J., Eshetu, A., Abdu, A., Wondimu, E., Beyi, A. F., Tufa, T. B., Revie, C. W. (2017). Assisting differential clinical diagnosis of cattle diseases using smartphone-based technology in low resource settings: a pilot study. *BMC Veterinary Research*, *13*(1), 323. https://doi.org/10.1186/s12917-017-1249-3

Biffa, D., Bogale, A., Godfroid, J., & Skjerve, E. (2012). Factors associated with severity of bovine tuberculosis in Ethiopian cattle. *Tropical Animal Health and Production*, *44*(5), 991–998. https://doi.org/10.1007/s11250-011-0031-y

Blomström, A.-L., Scharin, I., Stenberg, H., Figueiredo, J., Nhambirre, O., Abilio, A., Fafetine, J. (2016). Seroprevalence of Rift Valley fever virus in sheep and goats in Zambézia,

Mozambique. *Infection Ecology & Epidemiology*, *6*(1), 31343. https://doi.org/10.3402/iee.v6.31343

Bronsvoort, B. M. de C., Thumbi, S. M., Poole, E. J., Kiara, H., Tosas Auguet, O., Handel, I. G., Woolhouse, M. E. (2013). Design and descriptive epidemiology of the Infectious Diseases of East African Livestock (IDEAL) project, a longitudinal calf cohort study in western Kenya. *BMC Veterinary Research*, *9*(1), 171. https://doi.org/10.1186/1746-6148-9-171

Bugeza, J., Muwonge, A., Munyeme, M., Lasuba, P., Godfroid, J., & Kankya, C. (2018). Seroprevalence of bovine brucellosis and associated risk factors in Nakasongola district, Uganda. *Tropical Animal Health and Production*. https://doi.org/10.1007/s11250-018-1631-6

Byarugaba, D. K., Mugimba, K. K., Omony, J. B., Okitwi, M., Wanyana, A., Otim, M. O., Ducatez, M. F. (2014). High pathogenicity and low genetic evolution of avian paramyxovirus type I (Newcastle disease virus) isolated from live bird markets in Uganda. *Virology Journal*, *11*(1), 173. https://doi.org/10.1186/1743-422X-11-173

Byaruhanga, C., Oosthuizen, M. C., Collins, N. E., & Knobel, D. (2015). Using participatory epidemiology to investigate management options and relative importance of tick-borne diseases amongst transhumant zebu cattle in Karamoja Region, Uganda. *Preventive Veterinary Medicine*, *122*(3), 287–297. https://doi.org/10.1016/j.prevetmed.2015.10.011

Campbell, Z., Marsh, T., Mpolya, E., Thumbi, S., & Palmer, G. (2018). Newcastle disease vaccine adoption by smallholder households in Tanzania: Identifying determinants and barriers. *PLoS ONE*, *13*(10), e0206058. https://doi.org/10.1371/journal.pone.0206058

Campbell, Z., Otieno, L., Shirima, G., Marsh, T., & Palmer, G. (2019). Drivers of vaccination preferences to protect a low-value livestock resource: Willingness to pay for Newcastle disease vaccines by smallholder households. *Vaccine*, *37*(1), 11–18. https://doi.org/10.1016/j.vaccine.2018.11.058

Carlson, C. J., Kracalik, I. T., Ross, N., Alexander, K. A., Hugh-Jones, M. E., Fegan, M., Blackburn, J. K. (2019). The global distribution of Bacillus anthracis and associated anthrax risk to humans, livestock and wildlife. *Nature Microbiology*. https://doi.org/10.1038/s41564-019-0435-4

Catalano, D., Biasibetti, E., Lynen, G., Di Giulio, G., De Meneghi, D., Tomassone, L., Capucchio, M. T. (2015). "Ormilo disease" a disorder of zebu cattle in Tanzania: bovine cerebral theileriosis or new protozoan disease? *Tropical Animal Health and Production*, *47*(5), 895–901. https://doi.org/10.1007/s11250-015-0805-8

Catley, A., Admassu, B., Bekele, G., & Abebe, D. (2014). Livestock mortality in pastoralist herds in Ethiopia and implications for drought response. *Disasters*, *38*(3), 500–516. https://doi.org/10.1111/disa.12060

Catley, A., Chibunda, R. T. T., Ranga, E., Makungu, S., Magayane, F. T. T., Magoma, G., Vosloo, W. (2004). Participatory diagnosis of a heat-intolerance syndrome in cattle in Tanzania and association with foot-and-mouth disease. *Preventive Veterinary Medicine*, *65*(1–2), 17–30. https://doi.org/10.1016/j.prevetmed.2004.06.007

Catley, A., Irungu, P., Simiyu, K., Dadye, J., Mwakio, W., Kiragu, J., & Nyamwaro, S. O. (2002). Participatory investigations of bovine trypanosomiasis in Tana River District, Kenya. *Medical and Veterinary Entomology*, *16*(1), 55–66. https://doi.org/10.1046/j.0269-283x.2002.00346.x

Chaka, H., Aboset, G., Garoma, A., Gumi, B., & Thys, E. (2018). Cross-sectional survey of brucellosis and associated risk factors in the livestock–wildlife interface area of Nechisar National Park, Ethiopia. *Tropical Animal Health and Production*, *50*(5), 1041–1049. https://doi.org/10.1007/s11250-018-1528-4

Chang'A, J. S., Robertson, L. J., Mtambo, M. M. A., Mdegela, R. H., Løken, T., Reksen, O., …Reksen, O. (2011). Unexpected results from large-scale cryptosporidiosis screening study in calves in Tanzania. *Annals of Tropical Medicine & Parasitology*, *105*(7), 513–519. https://doi.org/10.1179/2047773211Y.0000000007

Chatikobo, P., Choga, T., Ncube, C., & Mutambara, J. (2013). Participatory diagnosis and prioritization of constraints to cattle production in some smallholder farming areas of Zimbabwe. *Preventive Veterinary Medicine*, *109*(3–4), 327–333. https://doi.org/10.1016/j.prevetmed.2012.10.013

Chazya, R., Muma, J. B., Mwacalimba, K. K., Karimuribo, E., Mkandawire, E., & Simuunza, M. (2014). A Qualitative Assessment of the Risk of Introducing Peste des Petits Ruminants into Northern Zambia from Tanzania. *Veterinary Medicine International*, *2014*, 1–10. https://doi.org/10.1155/2014/202618

Chikerema, S. M., Matope, G., & Pfukenyi, D. M. (2013). Awareness and Attitude Toward Zoonoses with Particular Reference to Anthrax Among Cattle Owners in Selected Rural Communities of Zimbabwe. *Vector-Borne and Zoonotic Diseases*, *13*(4), 243–249. https://doi.org/10.1089/vbz.2011.0916

Chikerema, S. M., Pfukenyi, D. M., Matope, G., & Bhebhe, E. (2012). Temporal and spatial distribution of cattle anthrax outbreaks in Zimbabwe between 1967 and 2006. *Tropical Animal Health and Production*, *44*(1), 63–70. https://doi.org/10.1007/s11250-011-9888-z

Chimana, H. M., Muma, J. B., Samui, K. L., Hangombe, B. M., Munyeme, M., Matope, G., Tryland, M. (2010). A comparative study of the seroprevalence of brucellosis in commercial and small-scale mixed dairy-beef cattle enterprises of Lusaka province and Chibombo district, Zambia. *Tropical Animal Health and Production*, *42*(7), 1541–1545. https://doi.org/10.1007/s11250-010-9604-4

Cleaveland, S., Shaw, D. J., Mfinanga, S. G., Shirima, G., Kazwala, R. R., Eblate, E., & Sharp, M. (2007). Mycobacterium bovis in rural Tanzania: risk factors for infection in human and cattle populations. *Tuberculosis (Edinburgh, Scotland)*, *87*(1), 30–43. https://doi.org/10.1016/j.tube.2006.03.001

Clifford, DL, Kazwala RR, Sadiki, H, Roug, A, Muse EA, Coppolillo, PC, Mazet, JAK. (2013). Tuberculosis infection in wildlife from the Ruaha ecosystem Tanzania: implications for wildlife, domestic animals, and human health. *Epidemiology and Infection*, *141*(7), 1371–1381. https://doi.org/10.1017/S0950268813000836

Coffin, J. L., Monje, F., Asiimwe-Karimu, G., Amuguni, H. J., & Odoch, T. (2015). A One Health, participatory epidemiology assessment of anthrax (Bacillus anthracis) management in Western Uganda. *Social Science & Medicine*, *129*, 44–50. https://doi.org/10.1016/j.socscimed.2014.07.037

Debele, G., Guru, M., Hundessa, F., & Duguma, M. (2013). Assessment of farmers' management practices and factors affecting goats' production system in Adami Tulu Jido Kombolcha district of East Shawa Zone, Ethiopia. *Agriculture and Biology Journal of North America*, *4*(5), 520–526.

Demeke, B., Jenberie, S., Tesfaye, B., Ayelet, G., Yami, M., Lamien, C. E., & Gelaye, E. (2017). Investigation of Marek's disease virus from chickens in central Ethiopia. *Tropical Animal Health and Production*, *49*(2), 403–408. https://doi.org/10.1007/s11250-016-1208-1

Dhikusooka, M. T., Ayebazibwe, C., Namatovu, A., Belsham, G. J., Siegismund, H. R., Wekesa, S. N., … Tjørnehøj, K. (2016). Unrecognized circulation of SAT 1 foot-and-mouth disease virus in cattle herds around Queen Elizabeth National Park in Uganda. *BMC Veterinary Research*, *12*(1), 5. https://doi.org/10.1186/s12917-015-0616-1

Duguma, A., Abera, S., Zewdie, W., Belina, D., & Haro, G. (2017). Status of bovine tuberculosis and its zoonotic implications in Borana zone, Southern Ethiopia. *Tropical Animal Health and Production*, *49*(3), 445–450. https://doi.org/10.1007/s11250-016-1213-4

Edao, B. M., Hailegebreal, G., Berg, S., Zewude, A., Zeleke, Y., Sori, T., Wood, J. L. N. (2018). Brucellosis in the Addis Ababa dairy cattle: the myths and the realities. *BMC Veterinary Research*, *14*(1), 396. https://doi.org/10.1186/s12917-018-1709-4

Elbrissi, A., Sabeil, Y. A., Khalifa, K. A., Enan, K., Khair, O. M., & El Hussein, A. M. (2017). Isolation, identification and differentiation of Campylobacter spp. using multiplex PCR assay from goats in Khartoum State, Sudan. *Tropical Animal Health and Production*, *49*(3), 575–581. https://doi.org/10.1007/s11250-017-1231-x

Eshetu, L., Yigezu, L., & Asfaw, Y. (2007). A study on contagious caprine pleuropneumonia (CCPP) in goats at an export oriented abattoir, Debrezeit, Ethiopia. *Tropical Animal Health and Production*, *39*(6), 427–432.

Ezama, A., Gonzalez, J.-P., Majalija, S., & Bajunirwe, F. (2018). Assessing short evolution brucellosis in a highly brucella endemic cattle keeping population of Western Uganda: a complementary use of Rose Bengal test and IgM rapid diagnostic test. *BMC Public Health*, *18*(1), 315. https://doi.org/10.1186/s12889-018-5228-9

Fafetine, J. M., Coetzee, P., Mubemba, B., Nhambirre, O., Neves, L., Coetzer, J. A. W., & Venter, E. H. (2016). Rift Valley Fever Outbreak in Livestock, Mozambique, 2014. *Emerging Infectious Diseases*, *22*(12), 2165–2167. https://doi.org/10.3201/eid2212.160310

Fafetine, J., Neves, L., Thompson, P. N., Paweska, J. T., Rutten, V. P. M. G., & Coetzer, J. A. W. (2013). Serological Evidence of Rift Valley Fever Virus Circulation in Sheep and Goats in Zambézia Province, Mozambique. *PLoS Neglected Tropical Diseases*, *7*(2), e2065. https://doi.org/10.1371/journal.pntd.0002065

Fandamu, P., Thys, E., Duchateau, L., & Berkvens, D. (2006). Perception of cattle farmers of the efficacy of east coast fever immunization in Southern Zambia. *Tropical Animal Health and Production*, *38*(1), 9–16. https://doi.org/10.1007/s11250-006-4341-4

Faris, D., Yilkal, A., Berhe, G., & Kelay, B. (2012). Seroprevalence and sero-conversion after vaccination against Peste des Petits Ruminants in sheep and goats from Awash Fentale District, Afar, Ethiopia. *Preventive Veterinary Medicine*, *103*(2/3), 157–162.

Fentie, T., Abebe, B., & Kassa, T. (2013). Small-scale family poultry production in north Gondar: characteristics, productivity and constraints. *Livestock Research for Rural Development*, *25*(161).

Fentie, T., Fenta, N., Leta, S., Molla, W., Ayele, B., Teshome, Y., Assefa, A. (2017). Sero-prevalence, risk factors and distribution of sheep and goat pox in Amhara Region, Ethiopia. *BMC Veterinary Research*, *13*(1), 385. https://doi.org/10.1186/s12917-017-1312-0

Fetene, T., & Kebede, N. (2009). Bovine tuberculosis of cattle in three districts of northwestern Ethiopia. *Tropical Animal Health and Production*, *41*(2), 273–277. https://doi.org/10.1007/s11250-008-9186-6

Gachohi, J. M. M., Ngumi, P. N. N., Kitala, P. M. M., & Skilton, R. A. A. (2010). Estimating seroprevalence and variation to four tick-borne infections and determination of associated risk factors in cattle under traditional mixed farming system in Mbeere District, Kenya. *Preventive Veterinary Medicine*, *95*(3–4), 208–223. https://doi.org/10.1016/j.prevetmed.2010.03.015

Gachohi, J. M., Njenga, M. K., Kitala, P., & Bett, B. (2016). Modelling Vaccination Strategies against Rift Valley Fever in Livestock in Kenya. *PLOS Neglected Tropical Diseases*, *10*(12), e0005049. https://doi.org/10.1371/journal.pntd.0005049

Gakuya, D. W., Mulei, C. M., & Wekesa, S. B. (2011). Use of ethnoveterinary remedies in the management of foot and mouth disease lesions in a dairy herd. *African Journal of Traditional, Complementary, and Alternative Medicines : AJTCAM*, *8*(2), 165–169. https://doi.org/10.4314/ajtcam.v8i2.63204

Gakuya, F., Ombui, J., Heukelbach, J., Maingi, N., Muchemi, G., Ogara, W., Alasaad, S. (2012). Knowledge of Mange among Masai Pastoralists in Kenya. *PLoS ONE*, *7*(8), e43342. https://doi.org/10.1371/journal.pone.0043342

Gari, G., Abie, G., Gizaw, D., Wubete, A., Kidane, M., Asgedom, H., Tuppurainen, E. S. M. (2015). Evaluation of the safety, immunogenicity and efficacy of three capripoxvirus vaccine strains against lumpy skin disease virus. *Vaccine*, *33*(28), 3256–3261. https://doi.org/10.1016/j.vaccine.2015.01.035

Gari, G., Bonnet, P., Roger, F., & Waret-Szkuta, A. (2011). Epidemiological aspects and financial impact of lumpy skin disease in Ethiopia. *Preventive Veterinary Medicine*, *102*(4), 274–283. https://doi.org/10.1016/j.prevetmed.2011.07.003

Gari, G., Grosbois, V., Waret-Szkuta, A., Babiuk, S., Jacquiet, P., & Roger, F. (2012). Lumpy skin disease in Ethiopia: Seroprevalence study across different agro-climate zones. *Acta Tropica*, *123*(2), 101–106. https://doi.org/10.1016/j.actatropica.2012.04.009

Gari, G., Warer-Szkuta, A., Grosbois, V., Jacquiet, P., & Roger, F. (2010). Risk factors associated with observed clinical lumpy skin disease in Ethiopia. *Epidemiology and Infection*, *138*(11), 1657–1666. https://doi.org/10.1017/S0950268810000506

Gebremedhin, E., Agonafir, A., Tessema, T., Tilahun, G., Medhin, G., Vitale, M., Dorny, P. (2013). Seroepidemiological study of ovine toxoplasmosis in East and West Shewa Zones of Oromia Regional State, Central Ethiopia. *BMC Veterinary Research*, *9*(1), 117. https://doi.org/10.1186/1746-6148-9-117

Gebremedhin, E., Tesfamaryam, G., Yunus, H., Duguma, R., Tilahun, G., Di Marco, V., & Vitale, M. (2015). Seroepidemiology of Toxoplasma gondii infection in free-range chickens (Gallus domesticus) of Central Ethiopia. *Epidemiology and Infection*, *143*(3), 608–617. https://doi.org/10.1017/S0950268814000971

Gelaye, E., Belay, A., Ayelet, G., Jenberie, S., Yami, M., Loitsch, A., Lamien, C. E. (2015). Capripox disease in Ethiopia: Genetic differences between field isolates and vaccine strain, and implications for vaccination failure. *Antiviral Research*, *119*, 28–35. https://doi.org/10.1016/j.antiviral.2015.04.008

Gelaye, E., Mekonnen, G., Jenberie, S., & Ayelet, G. (2013). Detection of sheep-associated malignant catarrhal fever from clinical cases in Ethiopian cattle. *Revue Scientifique et Technique de l'OIE*, *32*(2), 851–856. https://doi.org/10.20506/rst.32.2.2211

Getaneh, A. M., & Gebremedhin, E. Z. (2017). Meta-analysis of the prevalence of mastitis and associated risk factors in dairy cattle in Ethiopia. *Tropical Animal Health and Production*, *49*(4), 697–705. https://doi.org/10.1007/s11250-017-1246-3

Getaw, A., Beyene, D., Ayana, D., Megersa, B., & Abunna, F. (2010). Hydatidosis: Prevalence and its economic importance in ruminants slaughtered at Adama municipal abattoir, Central Oromia, Ethiopia. *Acta Tropica*, *113*(3), 221–225. https://doi.org/10.1016/j.actatropica.2009.10.019

Giday, M., & Teklehaymanot, T. (2013). Ethnobotanical study of plants used in management of livestock health problems by Afar people of Ada'ar District, Afar Regional State, Ethiopia. *Journal of Ethnobiology and Ethnomedicine*, *9*(1), 8. https://doi.org/10.1186/1746-4269-9-8

Girmay, G., Arega, B., Berkvens, D., Altaye, S. Z., & Muleta, G. (2018). Community-based tsetse fly control minimizes the effect of trypanosomosis on livestock in Metekel zone, Ethiopia. *Tropical Animal Health and Production*, *50*(3), 621–627. https://doi.org/10.1007/s11250-017-1478-2

Gitonga, P. N., Gachene, C. K., Njoroge, E., & Thumbi, S. M. (2016). Small ruminant husbandry practices amongst Kajiado and Marsabit pastoralists and their effects on Peste des petits ruminants control strategies. *Livestock Research for Rural Development*, *28*(2).

Gomo, C., Kanonhuwa, K., Godobo, F., Tada, O., & Makuza, S. M. (2017). Temporal and spatial distribution of lumpy skin disease (LSD) outbreaks in Mashonaland West Province of Zimbabwe from 2000 to 2013. *Tropical Animal Health and Production*, *49*(3), 509–514. https://doi.org/10.1007/s11250-017-1222-y

Gurmesa Umeta. (2011). Participatory analysis of problems limiting goat production at selected districts of East Showa zone, Ethiopia. *African Journal of Agricultural Research*, *6*(26). https://doi.org/10.5897/AJAR11.314

Gutiérrez-Expósito, D., Ferre, I., Ortega-Mora, L. M., & Álvarez-García, G. (2017). Advances in the diagnosis of bovine besnoitiosis: current options and applications for control. *International Journal for Parasitology*, *47*(12), 737–751. https://doi.org/10.1016/j.ijpara.2017.08.003

Habitu, T., Areda, D., Muwonge, A., Tessema, G. T., Skjerve, E., & Gebrehiwot, T. (2019). Prevalence and risk factors analysis of bovine tuberculosis in cattle raised in mixed crop-livestock farming system in Tigray region, Ethiopia. *Transboundary and Emerging Diseases*, *66*(1), 488–496. https://doi.org/10.1111/tbed.13050

Habte, A., & Ibrahim, N. (2018). Prevalence of Haemonchus contortus infection in sheep slaughtered at Jimma town municipal abattoir, Ethiopia. *Tropical Animal Health and Production*, *50*(8), 1865–1870. https://doi.org/10.1007/s11250-018-1637-0

Habte, T., Amare, A., Bettridge, J., Collins, M., Robert, C., & Wigley, P. (2017). *Guide to chicken health and management in Ethiopia*.

Hailu, B., Alemayehu, G., & Sied, N. (2015). Participatory epidemiological studies of major trade constraint diseases of goats in selected districts of Afar region. *Journal of Biology, Agriculture and Healthcare*, *5*(11), 140–148.

Hailu, B., Tolosa, T., Gari, G., Teklue, T., & Beyene, B. (2014). Estimated prevalence and risk factors associated with clinical Lumpy skin disease in north-eastern Ethiopia. *Preventive Veterinary Medicine*, *115*(1–2), 64–68. https://doi.org/10.1016/j.prevetmed.2014.03.013

Hamill, L., Picozzi, K., Fyfe, J., von Wissmann, B., Wastling, S., Wardrop, N., Welburn, S. C. (2017). Evaluating the impact of targeting livestock for the prevention of human and animal trypanosomiasis, at village level, in districts newly affected with T. b. rhodesiense in Uganda. *Infectious Diseases of Poverty*, *6*(1), 16. https://doi.org/10.1186/s40249-016-0224-8

Hassan, O. A., Ahlm, C., Sang, R., & Evander, M. (2011). The 2007 Rift Valley Fever Outbreak in Sudan. *PLoS Neglected Tropical Diseases*, *5*(9), e1229. https://doi.org/10.1371/journal.pntd.0001229

Hiko, A., & Agga, G. E. (2011). First-time detection of mycobacterium species from goats in Ethiopia. *Tropical Animal Health and Production*, *43*(1), 133–139. https://doi.org/10.1007/s11250-010-9665-4

Hørning, G., Rasmussen, S., Permin, A., & Bisgaard, M. (2003). Investigations on the influence of helminth parasites on vaccination of chickens against Newcastle disease virus under village conditions. *Tropical Animal Health and Production*, *35*(5), 415–424.

Hove, T., Lind, P., & Mukaratirwa, S. (2005). Seroprevalence of Toxoplasma gondii infection in goats and sheep in Zimbabwe. *The Onderstepoort Journal of Veterinary Research*, *72*(4), 267–272.

Howell, A., Mugisha, L., Davies, J., LaCourse, E., Claridge, J., Williams, D. J., Stothard, J. (2012). Bovine fasciolosis at increasing altitudes: Parasitological and malacological sampling on the slopes of Mount Elgon, Uganda. *Parasites & Vectors*, *5*(1), 196. https://doi.org/10.1186/1756-3305-5-196

Howson, E. L. A., Armson, B., Lyons, N. A., Chepkwony, E., Kasanga, C. J., Kandusi, S., Fowler, V. L. (2018). Direct detection and characterization of foot-and-mouth disease virus in East Africa using a field-ready real-time PCR platform. *Transboundary and Emerging Diseases*, *65*(1), 221–231. https://doi.org/10.1111/tbed.12684

Hutton, S., Bettridge, J., Christley, R., Habte, T., & Ganapathy, K. (2017). Detection of infectious bronchitis virus 793B, avian metapneumovirus, Mycoplasma gallisepticum and Mycoplasma synoviae in poultry in Ethiopia. *Tropical Animal Health and Production*, *49*(2), 317–322. https://doi.org/10.1007/s11250-016-1195-2

Ibrahim, A. M. E., Elfahal, A. M., & El Hussein, A. R. M. (2012). First report of Neospora caninum infection in cattle in Sudan. *Tropical Animal Health and Production*, *44*(4), 769–772. https://doi.org/10.1007/s11250-011-9963-5

Iraguha, B., Hamudikuwanda, H., & Mushonga, B. (2015). Bovine mastitis prevalence and associated risk factors in dairy cows in Nyagatare District, Rwanda. *Journal of the South African Veterinary Association*, *86*(1), 1228. https://doi.org/10.4102/jsava.v86i1.1228

Ishag, O. M., Saeed, I. K., & Ali, Y. H. (2015). Peste des petits ruminants outbreaks in White Nile State, Sudan. *The Onderstepoort Journal of Veterinary Research*, *82*(1), E1-4. https://doi.org/10.4102/ojvr.v82i1.897

Jemberu, W. T., Mourits, M. C. M., Sahle, M., Siraw, B., Vernooij, J. C. M., & Hogeveen, H. (2016). Epidemiology of Foot and Mouth Disease in Ethiopia: a Retrospective Analysis of District Level Outbreaks, 2007-2012. *Transboundary and Emerging Diseases*, *63*(6), e246–e259. https://doi.org/10.1111/tbed.12338

Jemberu, W. T., Mourits, M. C. M., Woldehanna, T., & Hogeveen, H. (2014). Economic impact of foot and mouth disease outbreaks on smallholder farmers in Ethiopia. *Preventive Veterinary Medicine*, *116*(1–2), 26–36. https://doi.org/10.1016/j.prevetmed.2014.06.004

Jemberu, W. T., Mourits, M., Rushton, J., & Hogeveen, H. (2016). Cost-benefit analysis of foot and mouth disease control in Ethiopia. *Preventive Veterinary Medicine*, *132*, 67–82. https://doi.org/10.1016/j.prevetmed.2016.08.008

Jemberu, W., Mourits, M., & Hogeveen, H. (2015). Farmers' Intentions to Implement Foot and Mouth Disease Control Measures in Ethiopia. *PloS One*, *10*(9), e0138363. https://doi.org/10.1371/journal.pone.0138363

Jenberie, S., Lynch, S. E., Kebede, F., Christley, R. M., Gelaye, E., Negussie, H., Ayelet, G. (2014). Genetic characterisation of infectious bursal disease virus isolates in Ethiopia. *Acta Tropica*, *130*, 39–43. https://doi.org/10.1016/j.actatropica.2013.09.025

Jenbreie, S., Ayelet, G., Gelaye, E., Kebede, F., Lynch, S. E., & Negussie, H. (2012). Infectious bursal disease: seroprevalence and associated risk factors in major poultry rearing areas of Ethiopia. *Tropical Animal Health and Production*, *45*(1), 75–79. https://doi.org/10.1007/s11250-012-0176-3

Kabi, F., Masembe, C., Muwanika, V., Kirunda, H., & Negrini, R. (2014). Geographic distribution of non-clinical Theileria parva infection among indigenous cattle populations in contrasting agro-ecological zones of Uganda: implications for control strategies. *Parasites & Vectors*, *7*(1), 414. https://doi.org/10.1186/1756-3305-7-414

Kabi, F., Muwanika, V., & Masembe, C. (2015). Spatial distribution of Brucella antibodies with reference to indigenous cattle populations among contrasting agro-ecological zones of Uganda. *Preventive Veterinary Medicine*, *121*(1–2), 56–63. https://doi.org/10.1016/j.prevetmed.2015.06.007

Kagira, J. M., & Kanyari, P. W. N. (2012). Questionnaire survey on urban and peri-urban livestock farming practices and disease control in Kisumu municipality, Kenya. *Journal of the South African Veterinary Association*, *81*(2), 82–86. https://doi.org/10.4102/jsava.v81i2.110

Kairu-Wanyoike, S. W., Kaitibie, S., Heffernan, C., Taylor, N. M., Gitau, G. K., Kiara, H., & McKeever, D. (2014). Willingness to pay for contagious bovine pleuropneumonia vaccination in Narok South District of Kenya. *Preventive Veterinary Medicine*, *115*(3–4), 130–142. https://doi.org/10.1016/j.prevetmed.2014.03.028

Kakandelwa, C., Siwila, J., Nalubamba, K. S., Muma, J. B., & Phiri, I. G. K. (2016). Prevalence of Giardia in dairy cattle in Lusaka and Chilanga districts, Zambia. *Veterinary Parasitology*, *215*, 114–116. https://doi.org/10.1016/j.vetpar.2015.11.004

Kamboyi, H. K., Garine-Wichatitsky, M., Hang'ombe, M. B., & Munyeme, M. (2019). Risk mapping and eco-anthropogenic assessment of anthrax in the upper Zambezi basin. *Veterinary Medicine and Science*, vms3.168. https://doi.org/10.1002/vms3.168

Kang'ethe, E. K., & Lang'a, K. A. (2009). Aflatoxin B1 and M1 contamination of animal feeds and milk from urban centers in Kenya. *African Health Sciences*, *9*(4), 218–226.

Karimuribo, E. D., Fitzpatrick, J. L., Bell, C. E., Swai, E. S., Kambarage, D. M., Ogden, N. H., … French, N. P. (2006). Clinical and subclinical mastitis in smallholder dairy farms in Tanzania: risk, intervention and knowledge transfer. *Preventive Veterinary Medicine*, *74*(1), 84–98. https://doi.org/10.1016/j.prevetmed.2006.01.009

Kasanga, C. J., Sallu, R., Kivaria, F., Mkama, M., Masambu, J., Yongolo, M., Rweyemamu, M. M. (2012). Foot-and-mouth disease virus serotypes detected in Tanzania from 2003 to 2010: Conjectured status and future prospects. *Onderstepoort J Vet Res*, *79*(2). https://doi.org/10.4102/ojvr.v79i2.462

Kasanga, C. J., Wadsworth, J., Mpelumbe-Ngeleja, C. A. R., Sallu, R., Kivaria, F., Wambura, P. N., King, D. P. (2015). Molecular Characterization of Foot-and-Mouth Disease Viruses Collected in Tanzania Between 1967 and 2009. *Transboundary and Emerging Diseases*, *62*(5), e19–e29. https://doi.org/10.1111/tbed.12200

Kasanga, C. J., Yamaguchi, T., Munang'andu, H. M., Ohya, K., & Fukushi, H. (2013). Molecular epidemiology of infectious bursal disease virus in Zambia. *Journal of the South African Veterinary Association*, *84*(1). https://doi.org/10.4102/jsava.v84i1.908

Kassaye, E., Moser, I., & Woldemeskel, M. (2003). Epidemiological study on clinical bovine dermatophilosis in northern Ethiopia. *DTW. Deutsche Tierarztliche Wochenschrift*, *110*(10), 422–425.

Kassian, E. N., Simuunza, M. C., Silayo, R. S., Moonga, L., Ndebe, J., Sugimoto, C., & Namangala, B. (2017). Prevalence and risk factors of bovine trypanosomosis in Kilwa district, Lindi region of southern Tanzania. *Veterinary Parasitology: Regional Studies and Reports*, *9*, 1–5. https://doi.org/10.1016/j.vprsr.2017.03.004

Katale, B. Z., Mbugi, E. V, Karimuribo, E. D., Keyyu, J. D., Kendall, S., Kibiki, G. S., Matee, M. I. (2013). Prevalence and risk factors for infection of bovine tuberculosis in indigenous cattle

in the Serengeti ecosystem, Tanzania. *BMC Veterinary Research*, *9*(1), 267. https://doi.org/10.1186/1746-6148-9-267

Kazoora, H. B., Majalija, S., Kiwanuka, N., & Kaneene, J. B. (2014). Prevalence of Mycobacterium bovis skin positivity and associated risk factors in cattle from Western Uganda. *Tropical Animal Health and Production*, *46*(8), 1383–1390. https://doi.org/10.1007/s11250-014-0650-1

Kemal, J., Sibhat, B., Abraham, A., Terefe, Y., Tulu, K. T., Welay, K., & Getahun, N. (2019). Bovine tuberculosis in eastern Ethiopia: prevalence, risk factors and its public health importance. *BMC Infectious Diseases*, *19*(1), 39. https://doi.org/10.1186/s12879-018-3628-1

Kerario, I. I., Simuunza, M. C., Chenyambuga, S. W., Koski, M., Hwang, S.-G., & Muleya, W. (2017). Prevalence and risk factors associated with Theileria parva infection in cattle in three regions of Tanzania. *Tropical Animal Health and Production*, *49*(8), 1613–1621. https://doi.org/10.1007/s11250-017-1367-8

Kerfua, S. D., Shirima, G., Kusiluka, L., Ayebazibwe, C., Martin, E., Arinaitwe, E., Haydon, D. T. (2019). Low topotype diversity of recent foot-and-mouth disease virus serotypes O and A from districts located along the Uganda and Tanzania border. *Journal of Veterinary Science*, *20*(2), e4. https://doi.org/10.4142/jvs.2019.20.e4

Keyyu, J. ., Kyvsgaard, N. ., Kassuku, A. ., & Willingham, A. (2003). Worm control practices and anthelmintic usage in traditional and dairy cattle farms in the southern highlands of Tanzania. *Veterinary Parasitology*, *114*(1), 51–61. https://doi.org/10.1016/S0304-4017(03)00111-0

Kihu, S. M., Gachohi, J. M., Ndungu, E. K., Gitao, G. C., Bebora, L. C., John, N. M., Ireri, R. (2015). Sero-epidemiology of Peste des petits ruminants virus infection in Turkana County, Kenya. *BMC Veterinary Research*, *11*(1), 87. https://doi.org/10.1186/s12917-015-0401-1

Kim, T. Y., Kwak, Y. S., Kim, J. Y., Nam, S.-H., Lee, I.-Y., Mduma, S., Yong, T.-S. (2018). Prevalence of Tick-Borne Pathogens from Ticks Collected from Cattle and Wild Animals in Tanzania in 2012. *The Korean Journal of Parasitology*, *56*(3), 305–308. https://doi.org/10.3347/kjp.2018.56.3.305

Kimaro, E. G., Mor, S. M., Gwakisa, P., & Toribio, J.-A. (2017). Seasonal occurrence of Theileria parva infection and management practices amongst Maasai pastoralist communities in Monduli District, Northern Tanzania. *Veterinary Parasitology*, *246*, 43–52. https://doi.org/10.1016/j.vetpar.2017.08.023

Kimaro, E. G., Toribio, J.-A. L. M. L. M. L., & Mor, S. M. (2017). Climate change and cattle vector-borne diseases: Use of participatory epidemiology to investigate experiences in pastoral communities in Northern Tanzania. *Preventive Veterinary Medicine*, *147*, 79–89. https://doi.org/10.1016/j.prevetmed.2017.08.010

Kimaro, E. G., Toribio, J.-A. L. M. L. M. L., Gwakisa, P., & Mor, S. M. (2018). Occurrence of trypanosome infections in cattle in relation to season, livestock movement and management practices of Maasai pastoralists in Northern Tanzania. *Veterinary Parasitology: Regional Studies and Reports*, *12*, 91–98. https://doi.org/10.1016/j.vprsr.2018.02.007

Kipronoh, A. K., Ombui, J. N., Kiara, H. K., Binepal, Y. S., Gitonga, E., & Wesonga, H. O. (2016). Prevalence of contagious caprine pleuro-pneumonia in pastoral flocks of goats in the Rift Valley region of Kenya. *Tropical Animal Health and Production*, *48*(1), 151–155. https://doi.org/10.1007/s11250-015-0934-0

Kipronoh, K. A., Ombui, J. N., Binepal, Y. S., Wesonga, H. O., Gitonga, E. K., Thuranira, E., & Kiara, H. K. (2016). Risk factors associated with contagious caprine pleuro-pneumonia in goats in pastoral areas in the Rift Valley region of Kenya. *Preventive Veterinary Medicine*, *132*, 107–112. https://doi.org/10.1016/j.prevetmed.2016.08.011

Kivaria, F. M. (2006). Estimated direct economic costs associated with tick-borne diseases on cattle in Tanzania. *Tropical Animal Health and Production*, *38*(4), 291–299. https://doi.org/10.1007/s11250-006-4181-2

Kivaria, F. M., Noordhuizen, J. P. T. M., & Kapaga, A. M. (2004). Risk indicators associated with subclinical mastitis in smallholder dairy cows in Tanzania. *Tropical Animal Health and Production*, *36*(6), 581–592.

Kivaria, F. M., Noordhuizen, J. P. T. M., & Msami, H. M. (2007). Risk factors associated with the incidence rate of clinical mastitis in smallholder dairy cows in the Dar es Salaam region of Tanzania. *Veterinary Journal (London, England : 1997)*, *173*(3), 623–629. https://doi.org/10.1016/j.tvjl.2006.01.009

Koka, H., Sang, R., Kutima, H. L., & Musila, L. (2018). Coxiella burnetii Detected in Tick Samples from Pastoral Communities in Kenya. *BioMed Research International*, *2018*, 1–5. https://doi.org/10.1155/2018/8158102

Komba, E. V. G., Mkupasi, E. M., Mwesiga, G. K., Mbyuzi, A. O., Busagwe, Z., Mzula, A., Nzalawahe, J. (2013). Occurrence of helminths and coccidia in apparently healthy free range local chickens slaughtered at Morogoro live bird market. *Tanzania Veterinary Journal*, *28*(2), 55–61.

Laing, G., Aragrande, M., Canali, M., Savic, S., & De Meneghi, D. (2018). Control of Cattle Ticks and Tick-Borne Diseases by Acaricide in Southern Province of Zambia: A Retrospective Evaluation of Animal Health Measures According to Current One Health Concepts. *Frontiers in Public Health*, *6*, 45. https://doi.org/10.3389/fpubh.2018.00045

Laisser, E. L. K., Chenyambuga, S. W., Karimuribo, E. D., Msalya, G., Kipanyula, M. J., Mwilawa, A. J., … Kusiluka, L. J. M. (2017). A review on prevalence, control measure, and tolerance of Tanzania Shorthorn Zebu cattle to East Coast fever in Tanzania. *Tropical Animal Health and Production*, *49*(4), 813–822. https://doi.org/10.1007/s11250-017-1266-z

Laisser, E. L. K., Kipanyula, M. J., Msalya, G., Mdegela, R. H., Karimuribo, E. D., Mwilawa, A. J., Chenyambuga, S. W. (2014). Tick burden and prevalence of Theileria parva infection in Tarime zebu cattle in the lake zone of Tanzania. *Tropical Animal Health and Production*, *46*(8), 1391–1396. https://doi.org/10.1007/s11250-014-0651-0

Lankester, F., Russell, G. C., Lugelo, A., Ndabigaye, A., Mnyambwa, N., Keyyu, J., Cleaveland, S. (2016). A field vaccine trial in Tanzania demonstrates partial protection against malignant catarrhal fever in cattle. *Vaccine*, *34*(6), 831–838. https://doi.org/10.1016/j.vaccine.2015.12.009

Larson, P. S., Espira, L., Grabow, C., Wang, C. A., Muloi, D., Browne, A. S., Eisenberg, J. N. S. (2019). The sero-epidemiology of Coxiella burnetii (Q fever) across livestock species and herding contexts in Laikipia County, Kenya. *Zoonoses and Public Health*, *66*(3), 316–324. https://doi.org/10.1111/zph.12567

Lasecka-Dykes, L., Wright, C., Di Nardo, A., Logan, G., Mioulet, V., Jackson, T., King, D. (2018). Full Genome Sequencing Reveals New Southern African Territories Genotypes Bringing Us Closer to Understanding True Variability of Foot-and-Mouth Disease Virus in Africa. *Viruses*, *10*(4), 192. https://doi.org/10.3390/v10040192

Lee, S.-H., Mossaad, E., Ibrahim, A. M., Ismail, A. A., Adjou Moumouni, P. F., Liu, M., Xuan, X. (2018). Detection and molecular characterization of tick-borne pathogens infecting sheep and goats in Blue Nile and West Kordofan states in Sudan. *Ticks and Tick-Borne Diseases*, *9*(3), 598–604. https://doi.org/10.1016/j.ttbdis.2018.01.014

Legesse, M., Medhin, G., Bayissa, M., & Mamo, G. (2018). Knowledge and perception of pastoral community members about brucellosis as a cause of abortion in animals and its zoonotic importance in Amibara district, Afar Region, Ethiopia. *PLOS ONE*, *13*(11), e0206457. https://doi.org/10.1371/journal.pone.0206457

Legesse, Y., Asfaw, Y., Sahle, M., Ayelet, G., Jenberie, S., & Negussie, H. (2013). First confirmation of foot and mouth disease virus serotype SAT-1 in cattle and small ruminants

in Ethiopia in 2007/08. *Tropical Animal Health and Production*, *45*(5), 1265–1267. https://doi.org/10.1007/s11250-012-0339-2

Lesnoff, M., Laval, G., Bonnet, P., Abdicho, S., Workalemahu, A., Kifle, D., Thiaucourt, F. (2004). Within-herd spread of contagious bovine pleuropneumonia in Ethiopian highlands. *Preventive Veterinary Medicine*, *64*(1), 27–40. https://doi.org/10.1016/j.prevetmed.2004.03.005

Lindahl, J. F., Young, J., Wyatt, A., Young, M., Alders, R., Bagnol, B., Grace, D. (2019). Do vaccination interventions have effects? A study on how poultry vaccination interventions change smallholder farmer knowledge, attitudes, and practice in villages in Kenya and Tanzania. *Tropical Animal Health and Production*, *51*(1), 213–220.

Lobago, F., & Woldemeskel, M. (2004). An outbreak of Marek's disease in chickens in central Ethiopia. *Tropical Animal Health and Production*, *36*(4), 397–406.

Luu, L., Bettridge, J., Christley, R. M., Melese, K., Blake, D., Dessie, T., Lynch, S. E. (2013). Prevalence and molecular characterisation of Eimeria species in Ethiopian village chickens. *BMC Veterinary Research*, *9*(1), 208. https://doi.org/10.1186/1746-6148-9-208

Lyons, N. A., Alexander, N., Stärk, K. D. C., Dulu, T. D., Rushton, J., & Fine, P. E. M. (2015). Impact of foot-and-mouth disease on mastitis and culling on a large-scale dairy farm in Kenya. *Veterinary Research*, *46*(1), 41. https://doi.org/10.1186/s13567-015-0173-4

Lyons, N. A., Jemberu, W. T., Chaka, H., Salt, J. S., & Rushton, J. (2019). Field-derived estimates of costs for Peste des Petits Ruminants vaccination in Ethiopia. *Preventive Veterinary Medicine*, *163*, 37–43. https://doi.org/10.1016/j.prevetmed.2018.12.007

Machila, N., Wanyangu, S. W., McDermott, J., Welburn, S. C., Maudlin, I., & Eisler, M. C. (2003). Cattle owners' perceptions of African bovine trypanosomiasis and its control in Busia and Kwale Districts of Kenya. *Acta Tropica*, *86*(1), 25–34. https://doi.org/10.1016/S0001-706X(02)00288-7

Madut, N. A., Muwonge, A., Nasinyama, G. W., Muma, J. B., Godfroid, J., Jubara, A. S., … Kankya, C. (2018). The sero-prevalence of brucellosis in cattle and their herders in Bahr el Ghazal region, South Sudan. *PLOS Neglected Tropical Diseases*, *12*(6), e0006456. https://doi.org/10.1371/journal.pntd.0006456

Mafirakureva, P., Saidi, B., & Mbanga, J. (2017). Incidence and molecular characterisation of lumpy skin disease virus in Zimbabwe using the P32 gene. *Tropical Animal Health and Production*, *49*(1), 47–54. https://doi.org/10.1007/s11250-016-1156-9

Magona J. S. P.; Olaho-Mukani, W.; Coleman, P. G.; Jonsson, N. N.; Welburn, S. C.; Eisler, M. C., J. W. . M. (2003). A comparative study on the clinical, parasitological and molecular diagnosis of bovine trypanosomosis in Uganda. *Onderstepoort Journal of Veterinary Research*, *70*(3), 213–218.

Magona, J. W., Walubengo, J., & Odimin, J. T. (2008). Acute haemorrhagic syndrome of bovine trypanosomosis in Uganda. *Acta Tropica*, *107*(2), 186–191. https://doi.org/10.1016/j.actatropica.2008.05.019

Maitho, T., & Kinyua, J. W. (2015). Factors and diseases influencing dairy goats production among small scale farmers in Laikipia East District, Kenya. *International Journal of Livestock Research*, *5*(12), 43–48.

Makita, K., Fèvre, E. M., Waiswa, C., Eisler, M. C., Thrusfield, M., & Welburn, S. C. (2011). Herd prevalence of bovine brucellosis and analysis of risk factors in cattle in urban and peri-urban areas of the Kampala economic zone, Uganda. *BMC Veterinary Research*, *7*(1), 60. https://doi.org/10.1186/1746-6148-7-60

Malak, A. K., Mpoke, L., Banak, J., Muriuki, S., Skilton, R. A., Odongo, D., Kiara, H. (2012). Prevalence of livestock diseases and their impact on livelihoods in Central Equatoria State, southern Sudan. *Preventive Veterinary Medicine*, *104*(3–4), 216–223. https://doi.org/10.1016/j.prevetmed.2011.12.001

Matiko, M. K., Salekwa, L. P., Kasanga, C. J., Kimera, S. I., Evander, M., & Nyangi, W. P. (2018). Serological evidence of inter-epizootic/inter-epidemic circulation of Rift Valley fever virus in domestic cattle in Kyela and Morogoro, Tanzania. *PLOS Neglected Tropical Diseases*, *12*(11), e0006931. https://doi.org/10.1371/journal.pntd.0006931

Matope, G., Bhebhe, E., Muma, J. B., Oloya, J., Madekurozwa, R. L., Lund, A., & Skjerve, E. (2011). Seroprevalence of brucellosis and its associated risk factors in cattle from smallholder dairy farms in Zimbabwe. *Tropical Animal Health and Production*, *43*(5), 975–982. https://doi.org/10.1007/s11250-011-9794-4

Matos, C. A., Gonçalves, L. R., de Souza Ramos, I. A., Mendes, N. S., Zanatto, D. C. S., André, M. R., & Machado, R. Z. (2019). Molecular detection and characterization of Ehrlichia ruminantium from cattle in Mozambique. *Acta Tropica*, *191*, 198–203. https://doi.org/10.1016/j.actatropica.2019.01.007

Mbyuzi, A. O., Komba, E. V. G., Kimera, S. I., & Kambarage, D. M. (2014). Sero-prevalence and associated risk factors of peste des petits ruminants and contagious caprine pleuro-pneumonia in goats and sheep in the Southern Zone of Tanzania. *Preventive Veterinary Medicine*, *116*(1–2), 138–144. https://doi.org/10.1016/j.prevetmed.2014.06.013

Meas, S., Nakayama, M., Usui, T., Nakazato, Y., Yasuda, J., Ohashi, K., & Onuma, M. (2004). Evidence for bovine immunodeficiency virus infection in cattle in Zambia. *The Japanese Journal of Veterinary Research*, *52*(1), 3–8.

Mebrahtu, K., Teshale, S., Esatu, W., Habte, T., & Gelaye, E. (2018). Evaluation of spray and oral delivery of Newcastle disease I2 vaccine in chicken reared by smallholder farmers in central Ethiopia. *BMC Veterinary Research*, *14*(1), 48. https://doi.org/10.1186/s12917-018-1355-x

Mekonnen, S. A. A., Koop, G., Melkie, S. T. T., Getahun, C. D. D., Hogeveen, H., & Lam, T. J. G. M. J. G. M. (2017). Prevalence of subclinical mastitis and associated risk factors at cow and herd level in dairy farms in North-West Ethiopia. *Preventive Veterinary Medicine*, *145*, 23–31. https://doi.org/10.1016/j.prevetmed.2017.06.009

Mekonnen, S. A., Koop, G., Lam, T. J. G. M., & Hogeveen, H. (2017). The intention of North-Western Ethiopian dairy farmers to control mastitis. *PLoS ONE*, *12*(8), e0182727.

Mekuriaw, A., Bitew, M., Gelaye, E., Mamo, B., & Ayelet, G. (2017). Infectious bursal disease: outbreak investigation, molecular characterization, and vaccine immunogenicity trial in Ethiopia. *Tropical Animal Health and Production*, *49*(6), 1295–1302. https://doi.org/10.1007/s11250-017-1328-2

Meskerem, A. (2017). Major health constraints and ethno-veterinary practices of small scale and backyard chicken production in some selected regions of Ethiopia. *Revue De Medecine Veterinaire*, *168*(1–3), 63–71.

Meunier, N. V., Sebulime, P., White, R. G., & Kock, R. (2017). Wildlife-livestock interactions and risk areas for cross-species spread of bovine tuberculosis. *Onderstepoort Journal of Veterinary Research*, *84*(1). https://doi.org/10.4102/ojvr.v84i1.1221

Miller, R., Nakavuma, J. L., Ssajjakambwe, P., Vudriko, P., Musisi, N., & Kaneene, J. B. (2016). The Prevalence of Brucellosis in Cattle, Goats and Humans in Rural Uganda: A Comparative Study. *Transboundary and Emerging Diseases*, *63*(6), e197–e210. https://doi.org/10.1111/tbed.12332

Mkama, M., Kasanga, C. J., Sallu, R., Ranga, E., Yongolo, M., Mulumba, M., Wambura, P. (2014). Serosurveillance of foot-and-mouth disease virus in selected livestock-wildlife interface areas of Tanzania. *Onderstepoort J Vet Res*, *81*(2). https://doi.org/10.4102/ojvr.v81i2.718

Mlilo, D., Mhlanga, M., Mwembe, R., Sisito, G., Moyo, B., & Sibanda, B. (2015). The epidemiology of malignant catarrhal fever (MCF) and contribution to cattle losses in farms

around Rhodes Matopos National Park, Zimbabwe. *Tropical Animal Health and Production*, *47*(5), 989–994. https://doi.org/10.1007/s11250-015-0821-8

Mohamed, S. B., Alagib, A., AbdElkareim, T. B., Hassan, M. M., Johnson, W. C., Hussein, H. E., Ueti, M. W. (2018). Molecular detection and characterization of Theileria spp. infecting cattle in Sennar State, Sudan. *Parasitology Research*, *117*(4), 1271–1276. https://doi.org/10.1007/s00436-018-5775-0

Mohammed, S., Munissi, J. J. E., & Nyandoro, S. S. (2016). Aflatoxin M1 in raw milk and aflatoxin B1 in feed from household cows in Singida, Tanzania. *Food Additives & Contaminants. Part B, Surveillance*, *9*(2), 85–90. https://doi.org/10.1080/19393210.2015.1137361

Moiane, I., Machado, A., Santos, N., Nhambir, A., Inlamea, O., Hattendorf, J., Correia-Neves, M. (2014). Prevalence of Bovine Tuberculosis and Risk Factor Assessment in Cattle in Rural Livestock Areas of Govuro District in the Southeast of Mozambique. *PLoS ONE*, *9*(3), e91527. https://doi.org/10.1371/journal.pone.0091527

Molla, B., & Delil, F. (2015). Mapping of major diseases and devising prevention and control regimen to common diseases in cattle and shoats in Dassenech district of South Omo Zone, South-Western Ethiopia. *Tropical Animal Health and Production*, *47*(1), 45–51. https://doi.org/10.1007/s11250-014-0681-7

Molla, W., de Jong, M. C. M. M., Gari, G., & Frankena, K. (2017). Economic impact of lumpy skin disease and cost effectiveness of vaccination for the control of outbreaks in Ethiopia. *Preventive Veterinary Medicine*, *147*, 100–107. https://doi.org/10.1016/j.prevetmed.2017.09.003

Molla, W., de Jong, M. C. M., & Frankena, K. (2017). Temporal and spatial distribution of lumpy skin disease outbreaks in Ethiopia in the period 2000 to 2015. *BMC Veterinary Research*, *13*(1), 310. https://doi.org/10.1186/s12917-017-1247-5

MOLLA, W., FRANKENA, K., & DE JONG, M. C. M. (2017). Transmission dynamics of lumpy skin disease in Ethiopia. *Epidemiology and Infection*, *145*(13), 2856–2863. https://doi.org/10.1017/S0950268817001637

Molla, W., Frankena, K., Gari, G., & de Jong, M. C. M. (2017). Field study on the use of vaccination to control the occurrence of lumpy skin disease in Ethiopian cattle. *Preventive Veterinary Medicine*, *147*, 34–41. https://doi.org/10.1016/j.prevetmed.2017.08.019

Molla, W., Frankena, K., Gari, G., Kidane, M., Shegu, D., & de Jong, M. C. M. (2018). Seroprevalence and risk factors of lumpy skin disease in Ethiopia. *Preventive Veterinary Medicine*, *160*, 99–104. https://doi.org/10.1016/j.prevetmed.2018.09.029

Moti, Y., Deken, R. de, Thys, E., Abbeele, J. van den, Duchateau, L., & Delespaux, V. (2015). PCR and microsatellite analysis of diminazene aceturate resistance of bovine trypanosomes correlated to knowledge, attitude and practice of livestock keepers in South-Western Ethiopia. *Acta Tropica*, *146*, 45–52.

Msami, H. (2007). *Poultry sector country review: Tanzania*.

Msoffe, P. L. M. M., Bunn, D., Muhairwa, A. P., Mtambo, M. M. A. A., Mwamhehe, H., Msago, A., … Cardona, C. J. (2010). Implementing poultry vaccination and biosecurity at the village level in Tanzania: A social strategy to promote health in free-range poultry populations. *Tropical Animal Health and Production*, *42*(2), 253–263. https://doi.org/10.1007/s11250-009-9414-8

Mubamba, C., Ramsay, G., Abolnik, C., Dautu, G., & Gummow, B. (2016). A retrospective study and predictive modelling of Newcastle Disease trends among rural poultry of eastern Zambia. *Preventive Veterinary Medicine*, *133*, 97–107. https://doi.org/10.1016/j.prevetmed.2016.09.017

Muema, J., Thumbi, S. M., Obonyo, M., Wanyoike, S., Nanyingi, M., Osoro, E., Karanja, S. (2017). Seroprevalence and Factors Associated with *Coxiella burnetii* Infection in Small Ruminants in Baringo County, Kenya. *Zoonoses and Public Health*, *64*(7), e31–e43. https://doi.org/10.1111/zph.12342

Mugabi, K. N., Mugisha, A., & Ocaido, M. (2010). Socio-economic factors influencing the use of acaricides on livestock: a case study of the pastoralist communities of Nakasongola District, Central Uganda. *Tropical Animal Health and Production*, *42*(1), 131–136. https://doi.org/10.1007/s11250-009-9396-6

Mugisha, A., McLeod, A., Percy, R., & Kyewalabye, E. (2005). Strategies, Effectiveness and Rationale of Vector-borne Disease Control in the Pastoralist System of South-western Uganda. *Tropical Animal Health and Production*, *37*(6), 479–489. https://doi.org/10.1007/s11250-005-2174-1

Mugisha, A., McLeod, A., Percy, R., & Kyewalabye, E. (2008). Socio-economic factors influencing control of vector-borne diseases in the pastoralist system of south western Uganda. *Tropical Animal Health and Production*, *40*(4), 287–297. https://doi.org/10.1007/s11250-007-9093-2

Mugizi, D. R., Boqvist, S., Nasinyama, G. W., Waiswa, C., Ikwap, K., ROCK, K., Erume, J. (2015). Prevalence of and factors associated with Brucella sero-positivity in cattle in urban and peri-urban Gulu and Soroti towns of Uganda. *Journal of Veterinary Medical Science*, *77*(5), 557–564. https://doi.org/10.1292/jvms.14-0452

Muhanguzi, D., Mugenyi, A., Bigirwa, G., Kamusiime, M., Kitibwa, A., Akurut, G. G., Tweyongyere, R. (2017). African animal trypanosomiasis as a constraint to livestock health and production in Karamoja region: a detailed qualitative and quantitative assessment. *BMC Veterinary Research*, *13*(1), 355. https://doi.org/10.1186/s12917-017-1285-z

Muhanguzi, D., Okello, W. O., Kabasa, J. D., Waiswa, C., Welburn, S. C., & Shaw, A. P. M. (2015). Cost analysis of options for management of African Animal Trypanosomiasis using interventions targeted at cattle in Tororo District; south-eastern Uganda. *Parasites & Vectors*, *8*(1), 387. https://doi.org/10.1186/s13071-015-0998-8

Muleme, M., Barigye, R., Khaitsa, M. L., Berry, E., Wamono, A. W., & Ayebazibwe, C. (2012). Effectiveness of vaccines and vaccination programs for the control of foot-and-mouth disease in Uganda, 2001–2010. *Tropical Animal Health and Production*, *45*(1), 35–43. https://doi.org/10.1007/s11250-012-0254-6

Mulugeta, Y., Yacob, H. T., & Ashenafi, H. (2010). Ectoparasites of small ruminants in three selected agro-ecological sites of Tigray Region, Ethiopia. *Tropical Animal Health and Production*, *42*(6), 1219–1224. https://doi.org/10.1007/s11250-010-9551-0

Muma, J. B., Godfroid, J., Samui, K. L., & Skjerve, E. (2007). The role of Brucella infection in abortions among traditional cattle reared in proximity to wildlife on the Kafue flats of Zambia. *Revue Scientifique et Technique (International Office of Epizootics)*, *26*(3), 721–730.

Muma, J. B., Syakalima, M., Munyeme, M., Zulu, V. C., Simuunza, M., & Kurata, M. (2013). Bovine Tuberculosis and Brucellosis in Traditionally Managed Livestock in Selected Districts of Southern Province of Zambia. *Veterinary Medicine International*, *2013*, 1–7. https://doi.org/10.1155/2013/730367

Munang'andu, H. M., Banda, F., Chikampa, W., Mutoloki, S., Syakalima, M., & Munyeme, M. (2012). Risk analysis of an anthrax outbreak in cattle and humans of Sesheke district of Western Zambia. *Acta Tropica*, *124*(2), 162–165. https://doi.org/10.1016/j.actatropica.2012.07.008

Mungube, E. O., Tenhagen, B. A., Kassa, T., Regassa, F., Kyule, M. N., Greiner, M., & Baumann, M. P. O. (2004). Risk factors for dairy cow mastitis in the central highlands of Ethiopia. *Tropical Animal Health and Production*, *36*(5), 463–472.

Munyeme, M., Munang'andu, H. M., Nambota, A., Muma, J. B., Phiri, A. M., & Nalubamba, K. S. (2012). The Nexus between Bovine Tuberculosis and Fasciolosis Infections in Cattle of the Kafue Basin Ecosystem in Zambia: Implications on Abattoir Surveillance. *Veterinary Medicine International*, *2012*, 1–6. https://doi.org/10.1155/2012/921869

Muraguri, G. R., McLeod, A., McDermott, J. J., & Taylor, N. (2005). The incidence of calf morbidity and mortality due to vector-borne infections in smallholder dairy farms in Kwale District, Kenya. *Veterinary Parasitology*, *130*(3–4), 305–315. https://doi.org/10.1016/j.vetpar.2004.11.026

Musinguzi, S., Suganuma, K., Asada, M., Laohasinnarong, D., Sivakumar, T., Yokoyama, N., Inoue, N. (2016). A PCR-based survey of animal African trypanosomosis and selected piroplasm parasites of cattle and goats in Zambia. *Journal of Veterinary Medical Science*, *78*(12), 1819–1824. https://doi.org/10.1292/jvms.16-0240

Mutebi, F., Krücken, J., Mencke, N., Feldmeier, H., von Samson-Himmelstjerna, G., & Waiswa, C. (2016). Two Severe Cases of Tungiasis in Goat Kids in Uganda. *Journal of Insect Science (Online)*, *16*(1), 34. https://doi.org/10.1093/jisesa/iew016

Mutinda, W. U., Nyaga, P. N., Mbuthia, P. G., Bebora, L. C., & Muchemi, G. (2014). Risk factors associated with infectious bursal disease vaccination failures in broiler farms in Kenya. *Tropical Animal Health and Production*, *46*(4), 603–608. https://doi.org/10.1007/s11250-013-0533-x

Mutua, E. N., Bukachi, S. A., Bett, B. K., Estambale, B. A., & Nyamongo, I. K. (2017). "We do not bury dead livestock like human beings": Community behaviors and risk of Rift Valley Fever virus infection in Baringo County, Kenya. *PLOS Neglected Tropical Diseases*, *11*(5), e0005582. https://doi.org/10.1371/journal.pntd.0005582

Muyobela, J., Nkunika, P. O. Y., & Mwase, E. T. (2015). Resistance status of ticks (Acari; Ixodidae) to amitraz and cypermethrin acaricides in Isoka District, Zambia. *Tropical Animal Health and Production*, *47*(8), 1599–1605. https://doi.org/10.1007/s11250-015-0906-4

Mwakapuja, R. S., Makondo, Z. E., Malakalinga, J., Moser, I., Kazwala, R. R., & Tanner, M. (2013). Molecular characterization of Mycobacterium bovis isolates from pastoral livestock at Mikumi-Selous ecosystem in the eastern Tanzania. *Tuberculosis*, *93*(6), 668–674. https://doi.org/10.1016/j.tube.2013.08.002

Mwebe, R., Nakavuma, J., & Moriyón, I. (2011). Brucellosis seroprevalence in livestock in Uganda from 1998 to 2008: a retrospective study. *Tropical Animal Health and Production*, *43*(3), 603–608. https://doi.org/10.1007/s11250-010-9739-3

Mwenda, R., Changula, K., Hang'ombe, B. M., Chidumayo, N., Mangani, A. S., Kaira, T., Simulundu, E. (2018). Characterization of field infectious bursal disease viruses in Zambia: evidence of co-circulation of multiple genotypes with predominance of very virulent strains. *Avian Pathology*, *47*(3), 300–313. https://doi.org/10.1080/03079457.2018.1449941

Nabukenya, I., Rubaire-Akiiki, C., Olila, D., Ikwap, K., & Höglund, J. (2014). Ethnopharmacological practices by livestock farmers in Uganda: Survey experiences from Mpigi and Gulu districts. *Journal of Ethnobiology and Ethnomedicine*, *10*(1), 9. https://doi.org/10.1186/1746-4269-10-9

Namatovu, A., Belsham, G. J., Ayebazibwe, C., Dhikusooka, M. T., Wekesa, S. N., Siegismund, H. R., … Tjørnehøj, K. (2015). Challenges for Serology-Based Characterization of Foot-and-Mouth Disease Outbreaks in Endemic Areas; Identification of Two Separate Lineages of Serotype O FMDV in Uganda in 2011. *Transboundary and Emerging Diseases*, *62*(5), 522–534. https://doi.org/10.1111/tbed.12170

Namatovu, A., Tjørnehøj, K., Belsham, G. J., Dhikusooka, M. T., Wekesa, S. N., Muwanika, V. B., … Ayebazibwe, C. (2015). Characterization of foot-and-mouth disease viruses (FMDVs) from Ugandan cattle outbreaks during 2012-2013: evidence for circulation of multiple serotypes. *PLOS ONE*, *10*(2), e0114811. https://doi.org/10.1371/journal.pone.0114811

Nanyingi, M. O., Muchemi, G. M., Thumbi, S. M., Ade, F., Onyango, C. O., Kiama, S. G., & Bett, B. (2017). Seroepidemiological Survey of Rift Valley Fever Virus in Ruminants in Garissa, Kenya. *Vector-Borne and Zoonotic Diseases*, *17*(2), 141–146. https://doi.org/10.1089/vbz.2016.1988

Ndeereh, D., Muchemi, G., & Thaiyah, A. (2016). Knowledge, attitudes and practices towards spotted fever group rickettsioses and Q fever in Laikipia and Maasai Mara, Kenya. *Journal of Public Health in Africa*, *7*(1). https://doi.org/10.4081/jphia.2016.545

Ndengu, M., de Garine-Wichatitsky, M., Pfukenyi, D., Tivapasi, M., Mukamuri, B., & Matope, G. (2017). Assessment of community awareness and risk perceptions of zoonotic causes of abortion in cattle at three selected livestock–wildlife interface areas of Zimbabwe. *Epidemiology and Infection*, *145*(7), 1304–1319. https://doi.org/10.1017/S0950268817000097

Ndhlovu, D. N., & Masika, P. J. (2015). Risk factors associated with clinical dermatophilosis in smallholder sector cattle herds of Zimbabwe at the Amblyomma variegatum and Amblyomma hebraeum interface. *Tropical Animal Health and Production*, *47*(2), 353–360. https://doi.org/10.1007/s11250-014-0727-x

Ndhlovu, D. N., & Masika, P. J. (2016). Bovine dermatophilosis: Awareness, perceptions and attitudes in the small-holder sector of north-west Zimbabwe. *Onderstepoort J Vet Res*, *83*(1), 1004. https://doi.org/10.4102/ojvr.v83i1.1004

Ndhlovu, F., Ndhlovu, D. N., Chikerema, S. M., Masocha, M., Nyagura, M., & Pfukenyi, D. M. (2017). Spatiotemporal patterns of clinical bovine dermatophilosis in Zimbabwe 1995–

2014. *Onderstepoort Journal of Veterinary Research*, *84*(1). https://doi.org/10.4102/ojvr.v84i1.1386

Negusssie, H., Kyule, M. N., Yami, M., Ayelet, G., & Jenberie T, S. (2011). Outbreak investigations and genetic characterization of foot-and-mouth disease virus in Ethiopia in 2008/2009. *Tropical Animal Health and Production*, *43*(1), 235–243. https://doi.org/10.1007/s11250-010-9683-2

Ngonyoka, A., Gwakisa, P. S., Estes, A. B., Salekwa, L. P., Nnko, H. J., Hudson, P. J., & Cattadori, I. M. (2017). Patterns of tsetse abundance and trypanosome infection rates among habitats of surveyed villages in Maasai steppe of northern Tanzania. *Infectious Diseases of Poverty*, *6*(1), 126. https://doi.org/10.1186/s40249-017-0340-0

Nguhiu-Mwangi, J., Mbithi, P., Wabacha, J., & Mbuthia, P. (2008). Retrospective study of foot conditions in dairy cows in urban and periurban areas of Kenya. *Israel Journal of Veterinary Medicine*, *63*(2), 40–45.

Nigussie, Z., Mesfin, T., Sertse, T., Fulasa, T. T., Regassa, F., Tolosa Fulasa, T., & Regassa, F. (2010). Seroepidemiological study of bovine viral diarrhea (BVD) in three agroecological zones in Ethiopia. *Tropical Animal Health and Production*, *42*(3), 319–321. https://doi.org/10.1007/s11250-009-9445-1

Njeru, J., Wareth, G., Melzer, F., Henning, K., Pletz, M. W., Heller, R., & Neubauer, H. (2016). Systematic review of brucellosis in Kenya: disease frequency in humans and animals and risk factors for human infection. *BMC Public Health*, *16*(1), 853. https://doi.org/10.1186/s12889-016-3532-9

Njuguna, J. N., Gicheru, M. M., Kamau, L. M., & Mbatha, P. M. (2017). Incidence and knowledge of bovine brucellosis in Kahuro district, Murang'a County, Kenya. *Tropical Animal Health and Production*, *49*(5), 1035–1040. https://doi.org/10.1007/s11250-017-1296-6

Nnko, H. J., Gwakisa, P. S., Ngonyoka, A., Saigilu, M., Ole-Neselle, M., Kisoka, W., Estes, A. (2017). Pastoralists' Vulnerability to Trypanosomiasis in Maasai Steppe. *EcoHealth*, *14*(4), 718–731. https://doi.org/10.1007/s10393-017-1275-4

Noah, E. Y., Kimera, S. I., Kusiluka, L. J. M., & Wambura, P. (2015). Abattoir surveillance demonstrates contagious bovine pleuropneumonia is widespread in Tanzania. *Tropical Animal Health and Production*, *47*(8), 1607–1613. https://doi.org/10.1007/s11250-015-0907-3

Nonga, H. E., & Karimuribo, E. D. (2009). A retrospective survey of hydatidosis in livestock in Arusha, Tanzania, based on abattoir data during 2005 - 2007. *Tropical Animal Health and Production*, *41*(7), 1253–1257. https://doi.org/10.1007/s11250-009-9308-9

Ntirandekura, J.-B., Matemba, L. E., Kimera, S. I., Muma, J. B., & Karimuribo, E. D. (2018). Association of Brucellosis with Abortion Prevalence in Humans and Animals in Africa: A Review. *African Journal of Reproductive Health*, *22*(3), 120–136. https://doi.org/10.29063/ajrh2018/v22i3.13

Nuru, A., Mamo, G., Zewude, A., Mulat, Y., Yitayew, G., Admasu, A., Ameni, G. (2017). Preliminary investigation of the transmission of tuberculosis between farmers and their cattle in smallholder farms in northwestern Ethiopia: a cross-sectional study. *BMC Research Notes*, *10*(1), 31. https://doi.org/10.1186/s13104-016-2349-z

Nyaguthii, D. M., Armson, B., Kitala, P. M., Sanz-Bernardo, B., Di Nardo, A., & Lyons, N. A. (2019). Knowledge and risk factors for foot-and-mouth disease among small-scale dairy farmers in an endemic setting. *Veterinary Research*, *50*(1), 33. https://doi.org/10.1186/s13567-019-0652-0

Nyakarahuka, L., de St. Maurice, A., Purpura, L., Ervin, E., Balinandi, S., Tumusiime, A., Shoemaker, T. R. (2018). Prevalence and risk factors of Rift Valley fever in humans and animals from Kabale district in Southwestern Uganda, 2016. *PLOS Neglected Tropical Diseases*, *12*(5), e0006412. https://doi.org/10.1371/journal.pntd.0006412

Nyirenda, S. S., Sakala, M., Moonde, L., Kayesa, E., Fandamu, P., Banda, F., & Sinkala, Y. (2019). Prevalence of bovine fascioliasis and economic impact associated with liver condemnation in abattoirs in Mongu district of Zambia. *BMC Veterinary Research*, *15*(1), (21 January 2019). https://doi.org/10.1186/s12917-019-1777-0

NZALAWAHE, J., KASSUKU, A. A., STOTHARD, J. R., COLES, G. C., & EISLER, M. C. (2015). Associations between trematode infections in cattle and freshwater snails in highland and lowland areas of Iringa Rural District, Tanzania. *Parasitology*, *142*(11), 1430–1439. https://doi.org/10.1017/S0031182015000827

Nzalawahe, J., Kassuku, A. A., Stothard, J., Coles, G. C., & Eisler, M. C. (2014). Trematode infections in cattle in Arumeru District, Tanzania are associated with irrigation. *Parasites & Vectors*, *7*(1), 107. https://doi.org/10.1186/1756-3305-7-107

Ochwo, S., VanderWaal, K., Munsey, A., Ndekezi, C., Mwebe, R., Okurut, A. R. A., Mwiine, F. N. (2018). Spatial and temporal distribution of lumpy skin disease outbreaks in Uganda (2002–2016). *BMC Veterinary Research*, *14*(1), 174. https://doi.org/10.1186/s12917-018-1503-3

Odoi, A., Gathuma, J. M., Gachuiri, C. K., & Omore, A. (2007). Risk factors of gastrointestinal nematode parasite infections in small ruminants kept in smallholder mixed farms in Kenya. *BMC Veterinary Research*, *3*, 6. https://doi.org/10.1186/1746-6148-3-6

Odongo, D. O., Tiampati, C. M., Mulinge, E., Mbae, C. K., Bishop, R. P., Zeyhle, E., Romig, T. (2018). Prevalence and genotyping of Echinococcus granulosus in sheep in Narok County, Kenya. *Parasitology Research*, *117*(7), 2065–2073. https://doi.org/10.1007/s00436-018-5889-4

Ogendo, A., Obonyo, M., Wasswa, P., Bitek, A., Mbugua, A., & Thumbi, S. M. (2017). Cryptosporidium infection in calves and the environment in Asembo, Western Kenya: 2015. *Pan African Medical Journal*, *28*. https://doi.org/10.11604/pamj.supp.2017.28.1.9313

Ohaga, S. O., Kokwaro, E. D., Ndiege, I. O., Hassanali, A., & Saini, R. K. (2007). Livestock farmers' perception and epidemiology of bovine trypanosomosis in Kwale District, Kenya. *Preventive Veterinary Medicine*, *80*(1), 24–33. https://doi.org/10.1016/j.prevetmed.2007.01.007

Okumu, T. A., John, N. M., Wabacha, J. K., Tsuma, V., & VanLeeuwen, J. (2019). Seroprevalence of antibodies for bovine viral diarrhoea virus, Brucella abortus and Neospora caninum, and their roles in the incidence of abortion/foetal loss in dairy cattle herds in Nakuru District, Kenya. *BMC Veterinary Research*, *15*(1), 95. https://doi.org/10.1186/s12917-019-1842-8

Okuni, J. B., Reinacher, M., Loukopoulos, P., & Ojok, L. (2013). Prevalence and spectrum of Johne's disease lesions in cattle slaughtered at two abattoirs in Kampala, Uganda. *Tropical Animal Health and Production*, *45*(5), 1197–1202. https://doi.org/10.1007/s11250-012-0346-3

Omony, J. B., Wanyana, A., Mugimba, K. K., Kirunda, H., Nakavuma, J. L., Otim-Onapa, M., & Byarugaba, D. K. (2016). Disparate thermostability profiles and HN gene domains of field isolates of Newcastle disease virus from live bird markets and waterfowl in Uganda. *Virology Journal*, *13*(1), 103. https://doi.org/10.1186/s12985-016-0560-0

Onono, J. O., Wieland, B., & Rushton, J. (2014). Estimation of impact of contagious bovine pleuropneumonia on pastoralists in Kenya. *Preventive Veterinary Medicine*, *115*(3–4), 122–129. https://doi.org/10.1016/j.prevetmed.2014.03.022

Onzima, R. B., Mukiibi, R., Ampaire, A., Benda, K. K., & Kanis, E. (2017). Between-breed variations in resistance/resilience to gastrointestinal nematodes among indigenous goat breeds in Uganda. *Tropical Animal Health and Production*, *49*(8), 1763–1769. https://doi.org/10.1007/s11250-017-1390-9

Orono, S. A., Gitao, G. C., Mpatswenumugabo, J. P., Chepkwony, M., Mutisya, C., Okoth, E., Cook, E. A. J. (2019). Field validation of clinical and laboratory diagnosis of wildebeest associated malignant catarrhal fever in cattle. *BMC Veterinary Research*, *15*(1), 69. https://doi.org/10.1186/s12917-019-1818-8

Ortiz-Pelaez, A., Pfeiffer, D. U., Tempia, S., Otieno, F. T., Aden, H. H., & Costagli, R. (2010). Risk mapping of Rinderpest sero-prevalence in Central and Southern Somalia based on spatial and network risk factors. *BMC Veterinary Research*, *6*(1), 22. https://doi.org/10.1186/1746-6148-6-22

Pandey, G. S., Simulundu, E., Mwiinga, D., Samui, K. L., Mweene, A. S., Kajihara, M., Takada, A. (2017). Clinical and subclinical bovine leukemia virus infection in a dairy cattle herd in Zambia. *Archives of Virology*, *162*(4), 1051–1056. https://doi.org/10.1007/s00705-016-3205-0

Picado, A., Speybroeck, N., Kivaria, F., Mosha, R. M., Sumaye, R. D., Casal, J., & Berkvens, D. (2011). Foot-and-Mouth Disease in Tanzania from 2001 to 2006. *Transboundary and Emerging Diseases*, *58*(1), 44–52. https://doi.org/10.1111/j.1865-1682.2010.01180.x

Rajeev, M., Mutinda, M., & Ezenwa, V. O. (2017). Pathogen Exposure in Cattle at the Livestock-Wildlife Interface. *EcoHealth*, *14*(3), 542–551. https://doi.org/10.1007/s10393-017-1242-0

Regassa, F., & Araya, M. (2012). In vitro antimicrobial activity of Combretum molle (Combretaceae) against Staphylococcus aureus and Streptococcus agalactiae isolated from crossbred dairy cows with clinical mastitis. *Tropical Animal Health and Production*, *44*(6), 1169–1173. https://doi.org/10.1007/s11250-011-0054-4

Romha, G., Gebru, G., Asefa, A., & Mamo, G. (2018). Epidemiology of Mycobacterium bovis and Mycobacterium tuberculosis in animals: Transmission dynamics and control challenges of zoonotic TB in Ethiopia. *Preventive Veterinary Medicine*, *158*, 1–17. https://doi.org/10.1016/j.prevetmed.2018.06.012

Salih, D. A., El Hussein, A. M., Kyule, M. N., Zessin, K.-H., Ahmed, J. S., & Seitzer, U. (2007). Determination of potential risk factors associated with Theileria annulata and Theileria parva infections of cattle in the Sudan. *Parasitology Research*, *101*(5), 1285–1288. https://doi.org/10.1007/s00436-007-0634-4

Sambo, E., Bettridge, J., Dessie, T., Amare, A., Habte, T., Wigley, P., & Christley, R. M. (2015). Participatory evaluation of chicken health and production constraints in Ethiopia. *Preventive Veterinary Medicine*, *118*(1), 117–127. https://doi.org/10.1016/j.prevetmed.2014.10.014

Schoonman, L. B., Wilsmore, T., & Swai, E. S. (2010). Sero-epidemiological investigation of bovine toxoplasmosis in traditional and smallholder cattle production systems of Tanga Region, Tanzania. *Tropical Animal Health and Production*, *42*(4), 579–587. https://doi.org/10.1007/s11250-009-9460-2

Schoonman, L., & Swai, E. S. (2010). Herd- and animal-level risk factors for bovine leptospirosis in Tanga region of Tanzania. *Tropical Animal Health and Production*, *42*(7), 1565–1572. https://doi.org/10.1007/s11250-010-9607-1

Seyoum, Z., Terefe, G., & Ashenafi, H. (2013). Farmers' perception of impacts of bovine trypanosomosis and tsetse fly in selected districts in Baro-Akobo and Gojeb river basins, Southwestern Ethiopia. *BMC Veterinary Research*, *9*(1), 214. https://doi.org/10.1186/1746-6148-9-214

Shabani, S. S., Ezekiel, M. J., Mohamed, M., & Moshiro, C. S. (2015). Knowledge, attitudes and practices on Rift Valley fever among agro pastoral communities in Kongwa and Kilombero districts, Tanzania. *BMC Infectious Diseases*, *15*(1), 363. https://doi.org/10.1186/s12879-015-1099-1

Shiferaw, T. J., Moses, K., & Manyahilishal, K. E. (2010). Participatory appraisal of foot and mouth disease in the Afar pastoral area, northeast Ethiopia: implications for understanding disease ecology and control strategy. *Tropical Animal Health and Production*, *42*(2), 193–201. https://doi.org/10.1007/s11250-009-9405-9

Shirima, G. M., Masola, S. N., Malangu, O. N., & Schumaker, B. A. (2014). Outbreak investigation and control case report of brucellosis: experience from livestock research centre, Mpwapwa, Tanzania. *The Onderstepoort Journal of Veterinary Research*, *81*(1). https://doi.org/10.4102/ojvr.v81i1.818

Sibhat, B., Asmare, K., Demissie, K., Ayelet, G., Mamo, G., & Ameni, G. (2017). Bovine tuberculosis in Ethiopia: A systematic review and meta-analysis. *Preventive Veterinary Medicine*, *147*, 149–157. https://doi.org/10.1016/j.prevetmed.2017.09.006

Sijapenda, J. E., Komba, E. V. G., & Nonga, H. E. (2017). Studies on seroprevalence and risk factors for occurrence of bovine brucellosis in cattle in Lindi District, Tanzania. *Tanzania Veterinary Journal*, *35*(Special Issue), 82–89.

Simukoko, H., Marcotty, T., Vercruysse, J., & Van den Bossche, P. (2011). Bovine trypanosomiasis risk in an endemic area on the eastern plateau of Zambia. *Research in Veterinary Science*, *90*(1), 51–54. https://doi.org/10.1016/j.rvsc.2010.04.021

Simulundu, E., Mtine, N., Kapalamula, T. F., Kajihara, M., Qiu, Y., Ngoma, J., Mweene, A. S. (2017). Genetic characterization of orf virus associated with an outbreak of severe orf in goats at a farm in Lusaka, Zambia (2015). *Archives of Virology*, *162*(8), 2363–2367. https://doi.org/10.1007/s00705-017-3352-y

Simwango, M., Ngonyoka, A., Nnko, H. J., Salekwa, L. P., Ole-Neselle, M., Kimera, S. I., & Gwakisa, P. S. (2017). Molecular prevalence of trypanosome infections in cattle and tsetse flies in the Maasai Steppe, northern Tanzania. *Parasites & Vectors*, *10*(1), 507. https://doi.org/10.1186/s13071-017-2411-2

Sitali, D. C., Twambo, M. C., Chisoni, M., Bwalya, M. J., & Munyeme, M. (2018). Lay perceptions, beliefs and practices linked to the persistence of anthrax outbreaks in cattle in the Western Province of Zambia. *Onderstepoort Journal of Veterinary Research*, *85*(1). https://doi.org/10.4102/ojvr.v85i1.1615

Sitawa, R., Mbogoh, S. G., Gathuma, J. M., & Kairu, S. (2016). An evaluation of economic returns from east coast fever control through infection and treatment method at household level in Nandi and Uasin-Gishu counties of Kenya. *International Journal of Agricultural Policy and Research*, *4*(8), 149–156.

Specht, E. (2008). Prevalence of bovine trypanosomosis in Central Mozambique from 2002 to 2005. *Onderstepoort J Vet Res*, *75*, 73–81. https://doi.org/10.1520/D0850-11.1

Spiegel, K. A., & Havas, K. A. (2019). The socioeconomic factors surrounding the initial emergence of peste des petits ruminants in Kenya, Uganda, and Tanzania from 2006 through 2008. *Transboundary and Emerging Diseases*, *66*(2), 627–633. https://doi.org/10.1111/tbed.13116

Stothard, J. R., Lockyer, A. E., Kabatereine, N. B., Tukahebwa, E. M., Kazibwe, F., Rollinson, D., & Fenwick, A. (2004). Schistosoma bovis in western Uganda. *Journal of Helminthology*, *78*(3), 281–284.

Sulayeman, M., Dawo, F., Mammo, B., Gizaw, D., & Shegu, D. (2018). Isolation, molecular characterization and sero-prevalence study of foot-and-mouth disease virus circulating in central Ethiopia. *BMC Veterinary Research*, *14*(1), 110. https://doi.org/10.1186/s12917-018-1429-9

Sumaye, R. D., Geubbels, E., Mbeyela, E., & Berkvens, D. (2013). Inter-epidemic Transmission of Rift Valley Fever in Livestock in the Kilombero River Valley, Tanzania: A Cross-Sectional Survey. *PLoS Neglected Tropical Diseases*, *7*(8), e2356. https://doi.org/10.1371/journal.pntd.0002356

Swai, E. S., Hayghaimo, A. A., Hassan, A. A., & Mhina, B. S. (2015). The slaughter of increased numbers of pregnant cows in Tanga abattoir, Tanzania: A cause for concern? *The Onderstepoort Journal of Veterinary Research*, *82*(1), E1-5. https://doi.org/10.4102/ojvr.v82i1.947

Swai, E. S., Karimuribo, E. D., & Kambarage, D. M. (2012). Risk factors for smallholder dairy cattle mortality in Tanzania. *Journal of the South African Veterinary Association*, *81*(4), 241–246. https://doi.org/10.4102/jsava.v81i4.155

Swai, E. S., Karimuribo, E. D., Ogden, N. H., French, N. P., Fitzpatrick, J. L., Bryant, M. J., & Kambarage, D. M. (2005). Seroprevalence estimation and risk factors for A. marginale on smallholder dairy farms in Tanzania. *Tropical Animal Health and Production*, *37*(8), 599–610.

Swai, E. S., Kessy, M. J., Sanka, P. N., & Mtui, P. F. (2011). A serological survey for infectious bursal disease virus antibodies in free-range village chickens in northern Tanzania. *Journal of the South African Veterinary Association*, *82*(1), 32–35. https://doi.org/10.4102/jsava.v82i1.30

Swiswa, S., Masocha, M., Pfukenyi, D. M., Dhliwayo, S., & Chikerema, S. M. (2017). Long-term changes in the spatial distribution of lumpy skin disease hotspots in Zimbabwe. *Tropical Animal Health and Production*, *49*(1), 195–199. https://doi.org/10.1007/s11250-016-1180-9

Syakalima, M., Simuunza, M., & Zulu, V. C. (2018). Ethnoveterinary treatments for common cattle diseases in four districts of the Southern Province, Zambia. *Veterinary World*, *11*(2), 141–145. https://doi.org/10.14202/vetworld.2018.141-145

Taha, K. M., Salih, D. A., Ahmed, B. M., Enan, K. A., Ali, A. M., & Elhussein, A. M. (2011). First confirmed report of outbreak of malignant ovine theileriosis among goats in Sudan. *Parasitology Research*, *109*(6), 1525–1527. https://doi.org/10.1007/s00436-011-2428-y

Tebug, S. F., Njunga, G. R., Chagunda, M. G. G., Mapemba, J. P., Awah-Ndukum, J., & Wiedemann, S. (2014). Risk, knowledge and preventive measures of smallholder dairy farmers in northern Malawi with regard to zoonotic brucellosis and bovine tuberculosis. *Onderstepoort J Vet Res*, *81*(1). https://doi.org/10.4102/ojvr.v81i1.594

Tedla, M., & Degefa, K. (2017). Bacteriological study of calf colisepticemia in Alage Dairy Farm, Southern Ethiopia. *BMC Research Notes*, *10*(1), 710. https://doi.org/10.1186/s13104-017-3038-2

Tedla, M., Mehari, F., & Kebede, H. (2018). A cross-sectional survey and follow up study on major dairy health problems in large and small scale urban farms in Mekelle, Tigray, Ethiopia. *BMC Research Notes*, *11*(1), 236. https://doi.org/10.1186/s13104-018-3347-0

Tekle, T., Terefe, G., Cherenet, T., Ashenafi, H., Akoda, K. G., Teko-Agbo, A., Delespaux, V. (2018). Aberrant use and poor quality of trypanocides: a risk for drug resistance in south western Ethiopia. *BMC Veterinary Research*, *14*(1), 4. https://doi.org/10.1186/s12917-017-1327-6

Terefe, Y., Girma, S., Mekonnen, N., & Asrade, B. (2017). Brucellosis and associated risk factors in dairy cattle of eastern Ethiopia. *Tropical Animal Health and Production*, *49*(3), 599–606. https://doi.org/10.1007/s11250-017-1242-7

Terfa, Z. G. G., Garikipati, S., Kassie, G., Bettridge, J. M. M., & Christley, R. M. M. (2018). Eliciting preferences for attributes of Newcastle disease vaccination programmes for village poultry in Ethiopia. *Preventive Veterinary Medicine*, *158*, 146–151. https://doi.org/10.1016/j.prevetmed.2018.08.004

Tesfaye, G., Tsegaye, W., Chanie, M., & Abinet, F. (2011). Seroprevalence and associated risk factors of bovine brucellosis in Addis Ababa dairy farms. *Tropical Animal Health and Production*, *43*(5), 1001–1005. https://doi.org/10.1007/s11250-011-9798-0

Teshome, D., Sori, T., Sacchini, F., & Wieland, B. (2019). Epidemiological investigations of contagious caprine pleuropneumonia in selected districts of Borana zone, Southern Oromia, Ethiopia. *Tropical Animal Health and Production*, *51*(3), 703–711. https://doi.org/10.1007/s11250-018-1744-y

Tibbo, M., Mukasa-Mugerwa, E., Woldemeskel, M., & Rege, J. E. O. (2003). Risk factors for mortality associated with respiratory disease among Menz and Horro sheep in Ethiopia. *Veterinary Journal (London, England : 1997)*, *165*(3), 276–287.

Tolosa, T., Verbeke, J., Piepers, S., Supré, K., & De Vliegher, S. (2013). Risk factors associated with subclinical mastitis as detected by California Mastitis Test in smallholder dairy farms in Jimma, Ethiopia using multilevel modelling. *Preventive Veterinary Medicine*, *112*(1–2), 68–75. https://doi.org/10.1016/j.prevetmed.2013.06.009

Toye, P. G., Batten, C. A., Kiara, H., Henstock, M. R., Edwards, L., Thumbi, S., Oura, C. A. L. (2013). Bluetongue and Epizootic Haemorrhagic Disease virus in local breeds of cattle in Kenya. *Research in Veterinary Science*, *94*(3), 769–773. https://doi.org/10.1016/j.rvsc.2012.11.001

Tschopp, R., Hattendorf, J., Roth, F., Choudhoury, A., Shaw, A., Aseffa, A., & Zinsstag, J. (2012). Cost Estimate of Bovine Tuberculosis to Ethiopia (pp. 249–268). https://doi.org/10.1007/82_2012_245

Tsegay, A., Tuli, G., Kassa, T., & Kebede, N. (2015). Seroprevalence and risk factors of Brucellosis in small ruminants slaughtered at Debre Ziet and Modjo export abattoirs, Ethiopia. *The Journal of Infection in Developing Countries*, *9*(04), 373–380. https://doi.org/10.3855/jidc.4993

VanderWaal, K., Omondi, G. P., & Obanda, V. (2014). Mixed-host aggregations and helminth parasite sharing in an East African wildlife–livestock system. *Veterinary Parasitology*, *205*(1–2), 224–232. https://doi.org/10.1016/j.vetpar.2014.07.015

Vhoko, K., Iannetti, S., Burumu, J., Ippoliti, C., Bhebhe, B., & De Massis, F. (2018). Estimating the prevalence of brucellosis in cattle in Zimbabwe from samples submitted to the central veterinary laboratory between 2010 and 2014. *Veterinaria Italiana*, *54*(1), 21–27. https://doi.org/10.12834/VetIt.1111.6191.2

Vordermeier, M., Ameni, G., Berg, S., Bishop, R., Robertson, B. D., Aseffa, A., Young, D. B. (2012). The influence of cattle breed on susceptibility to bovine tuberculosis in Ethiopia. *Comparative Immunology, Microbiology and Infectious Diseases*, *35*(3), 227–232. https://doi.org/10.1016/j.cimid.2012.01.003

Vudriko, P., Okwee-Acai, J., Byaruhanga, J., Tayebwa, D. S., Omara, R., Muhindo, J. B., Suzuki, H. (2018). Evidence-based tick acaricide resistance intervention strategy in Uganda: Concept and feedback of farmers and stakeholders. *Ticks and Tick-Borne Diseases*, *9*(2), 254–265. https://doi.org/10.1016/j.ttbdis.2017.09.011

Vudriko, P., Okwee-Acai, J., Tayebwa, D. S., Byaruhanga, J., Kakooza, S., Wampande, E., Suzuki, H. (2016). Emergence of multi-acaricide resistant Rhipicephalus ticks and its implication on chemical tick control in Uganda. *Parasites & Vectors*, *9*(1), 4. https://doi.org/10.1186/s13071-015-1278-3

Wambua, L., Wambua, P. N., Ramogo, A. M., Mijele, D., & Otiende, M. Y. (2016). Wildebeest-associated malignant catarrhal fever: perspectives for integrated control of a lymphoproliferative disease of cattle in sub-Saharan Africa. *Archives of Virology*, *161*(1), 1–10. https://doi.org/10.1007/s00705-015-2617-6

Wanyoike, F., & Rich, K. M. (2010). An Assessment of the Regional and National Socio-Economic Impacts of the 2007 Rift Valley Fever Outbreak in Kenya. *The American Journal of Tropical Medicine and Hygiene*, *83*(2_Suppl), 52–57. https://doi.org/10.4269/ajtmh.2010.09-0291

Wardrop, N. A., Thomas, L. F., Cook, E. A. J., de Glanville, W. A., Atkinson, P. M., Wamae, C. N., & Fèvre, E. M. (2016). The Sero-epidemiology of Coxiella burnetii in Humans and Cattle, Western Kenya: Evidence from a Cross-Sectional Study. *PLOS Neglected Tropical Diseases*, *10*(10), e0005032. https://doi.org/10.1371/journal.pntd.0005032

Wekesa, S. N., Muwanika, V. B., Siegismund, H. R., Sangula, A. K., Namatovu, A., Dhikusooka, M. T., … Belsham, G. J. (2015). Analysis of Recent Serotype O Foot-and-Mouth Disease Viruses from Livestock in Kenya: Evidence of Four Independently Evolving Lineages. *Transboundary and Emerging Diseases*, *62*(3), 305–314. https://doi.org/10.1111/tbed.12152

Welay, G. M., Tedla, D. G., Teklu, G. G., Weldearegay, S. K., Shibeshi, M. B., Kidane, H. H., … Abraha, T. H. (2018). A preliminary survey of major diseases of ruminants and management practices in Western Tigray province, northern Ethiopia. *BMC Veterinary Research*, *14*(1), 293. https://doi.org/10.1186/s12917-018-1621-y

Weny, G., Okwee-Acai, J., Okech, S. G., Tumwine, G., Ndyanabo, S., Abigaba, S., & Goldberg, T. L. (2017). Prevalence and Risk Factors Associated with Hemoparasites in Cattle and Goats at the Edge of Kibale National Park, Western Uganda. *Journal of Parasitology*, *103*(1), 69–74. https://doi.org/10.1645/16-33

Wesonga, F. D., Gachohi, J. M., Kitala, P. M., Gathuma, J. M., & Njenga, M. J. (2017). Seroprevalence of Anaplasma marginale and Babesia bigemina infections and associated risk factors in Machakos County, Kenya. *Tropical Animal Health and Production*, *49*(2), 265–272. https://doi.org/10.1007/s11250-016-1187-2

Wesonga, F. D., Wesongah, J. O., Chemuliti, J., Wanjala, K., Munga, L., & Gitau, P. (2006). Seroprevalence of Ehrlichia Ruminantium (Heartwater) in small ruminants in a pastoral production system in Narok District, Kenya. *Bulletin of Animal Health and Production in Africa*, *54*(1), 23–33.

Woldemeskel, M., & Mersha, G. (2010). Study on caprine and ovine dermatophilosis in Wollo, Northeast Ethiopia. *Tropical Animal Health and Production*, *42*(1), 41–44. https://doi.org/10.1007/s11250-009-9383-y

Wolff, C., Boqvist, S., Stǻhl, K., Masembe, C., Sternberg-Lewerin, S., Ståhl, K., Sternberg-Lewerin, S. (2017). Biosecurity aspects of cattle production in Western Uganda, and associations with seroprevalence of brucellosis, salmonellosis and bovine viral diarrhoea. *BMC Veterinary Research*, *13*(1), (6 December 2017). https://doi.org/10.1186/s12917-017-1306-y

Yamada, S., Konnai, S., Imamura, S., Simuunza, M., Chembensofu, M., Chota, A., Ohashi, K. (2009). PCR-based detection of blood parasites in cattle and adult Rhipicephalus

appendiculatus ticks. *Veterinary Journal (London, England : 1997)*, *182*(2), 352–355. https://doi.org/10.1016/j.tvjl.2008.06.007

Yasine, A., Kumsa, B., Hailu, Y., & Ayana, D. (2015). Mites of sheep and goats in Oromia Zone of Amhara Region, North Eastern Ethiopia: species, prevalence and farmers awareness. *BMC Veterinary Research*, *11*(1), 122. https://doi.org/10.1186/s12917-015-0433-6

Yihunie, A., Urga, B., & Alebie, G. (2019). Prevalence and risk factors of bovine schistosomiasis in Northwestern Ethiopia. *BMC Veterinary Research*, *15*(1), 12. https://doi.org/10.1186/s12917-018-1757-9

Zvinorova, P. I., Halimani, T. E., Muchadeyi, F. C., Katsande, S., Gusha, J., & Dzama, K. (2017). Management and control of gastrointestinal nematodes in communal goat farms in Zimbabwe. *Tropical Animal Health and Production*, *49*(2), 361–367.
