# Supplementary material for: Prioritizing smallholder animal health needs in East Africa, West Africa, and South Asia using three approaches: Literature review, expert workshops, and practitioner surveys
Source: Prev Vet Med. 2021 Apr;189:105279. doi: 10.1016/j.prevetmed.2021.105279 (PMC8024747; doi:10.1016/j.prevetmed.2021.105279)
Supplement: Supplementary file 6 [file mmc6.pdf]

Smallholder Animal Health Needs Assessment
East Africa Small Ruminants

GALVmed

August 5, 2019

Assembled by Zoë Campbell, Thembinkosi Ramuthivheli, Tom Osebe, and Paul Coleman

Meta-analysis summary

The literature review yielded 100 articles, 35 of which focused on impact, incidence, and prevalence, and are summarized below. The table below shows the number of articles in which each animal health concern appears in the literature about small ruminants in East Africa (many articles mention multiple concerns). No articles were given a green ranking for focus on both incidence/ prevalence and impact. The summarized articles are in the bibliography within this document. The full list of articles considered in the East Africa meta-analysis is available as a separate document.

The most mentioned concerns are Peste des Petits Ruminants (PPR), ectoparasites, mange, helminths, endoparasites, Rift Valley fever (RVF), brucellosis, and contagious caprine pleuropneumonia.

Animal health concern	Frequency of mention (meta-analysis)	Frequency of mention (impact article)
PPR	20	10
Ectoparasites	19	9
Mange	16	9
Helminth	12	5
Endoparasites	12	6
RVF	12	6
Brucellosis	10	4
CCPP	10	5
Tick-borne disease	6	2
FMD	6	2
Goat pox	6	2

AAT	5	3
Pasteurellosis	5	1
Sheep pox	5	3
Abortion	4	1
Theileriosis	4	2
Q fever	4	1
Diarrhoea	4	2
Heartwater	4	3
Hydatidosis	4	2
Dermatophilosis	3	1
Anaplasmosis	3	3
Toxoplasmosis	3	1
Orf	3	2
Anthrax	3	1
Mastitis	3	1
Babesiosis	2	2
Leptospirosis	2	1
Tuberculosis	2	1
Haemonchosis	1	0

Table of articles

Abbreviations:

PPR – Peste des Petits ruminants

CCPP – Contagious caprine pleuropneumonia

TBD – Tick-borne diseases

SGP – Sheep and goat pox

TB – Tuberculosis

Note: Clicking on the article number links to an expanded summary of the article below. The "web" link connects to the abstract of the article online or to full text for open access articles.

#	Citation	Country	Study design	Pathogen/ disease	Findings
1<br>2<br>web	(Hailu, Alemayehu, & Sied, 2015)	Ethiopia	Qualitative<br>12 focus groups with stakeholders, 291 interviews with pastoralists keeping goats and sheep	PPR<br>Pasteurellosis<br>Goat pox<br>CCPP<br>Ectoparasites	**Perceptions:** Pastoralists motioned PPR, Pasteurellosis, goat pox, External parasites and CCPP as most important diseases in terms of impact on livelihoods. Quarantine centres listed PPR, pasteurellosis, CCPP, goat pox and external parasites most important diseases in terms of impact on businesses.<br>**Impact:** Animal characteristics in terms of health, quality and other criteria required by exporters were known only by 8% of the producers.

					Occurrence of such diseases impacts livestock producers by marginalizing them from higher price livestock markets and restricting their capacity for value-added trade.
2<br>2<br>web	(Abdilatif, Onono, & Mutua, 2018)	Kenya	Mixed-methods<br>Focus groups<br>Interviews<br>Questionnaire<br>Purposive sample<br>~ 160 pastoralists	PPR<br>CCPP<br>Goat pox<br>TBD<br>Pneumonia<br>Mange<br>Helminths	**Perceptions:** Vaccination perceived to be most effective disease control strategy. PPR was considered a relatively new disease, only having been introduced within the last 10 years.<br>**Impact:** Pastoralists ranked diseases amongst sheep and goats by impact to livelihood as follows: PPR, CCPP, Goat Pox, tick-borne diseases (TBD), pneumonia, mange, helminths.
3<br>2<br>web	(Faris, Yilkal, Berhe, & Kelay, 2012)	Ethiopia	Cross-sectional, seroprevalence, post-vaccination sero-conversion rate,<br>1096 ruminants<br>Pastoralists	PPR	**Incidence/ Prevalence:** Post-vaccination sero-conversion rate of 1096 sheep and goats vaccinated with a thermolabile PPR vaccine was 61%, indicating weak herd immunity. No statistically significant difference in vaccination by veterinary professionals and CAHWs, Community Animal Health Workers.
4<br>2<br>web	(Bett, Jost, Allport, & Mariner, 2009)	Kenya	Cross-sectional, Participatory epidemiology, Qualitative methods,<br>~320 focus group members,<br>Pastoralists	PPR<br>CCPP<br>Mange	**Incidence/ Prevalence:** For goats, the overall relative incidence scores of peste des petits ruminants (PPR), contagious caprine pleuropneumonia (CCPP) and mange were 23.5% (15, 34), 25% (21, 45) and 20% (19, 28), respectively (10th and 90th percentiles in parentheses).<br>**Impact:** The respective median scores for case fatality rates were 66% (45, 76.5), 62.5% (25, 100) and 73.2% (21.4, 85.7). Disease impact matrix

					scores indicated that mange was the most important disease of goats.<br>*Note:* Relative scores are achieved by pastoralists ranking diseases and should not be confused with epidemiology terms.
5<br>3<br>web	(Chazya et al., 2014)	Tanzania	Cross-sectional, Qualitative risk assessment, Smallholders	PPR	The probability of occurrence of PPR in northern Zambia from Tanzania via live goat trade was rated as "high" and the economic consequences were also rated as "high." Finally, the overall risk of introducing PPR into northern Zambia from Tanzania at the time of the assessment was rated "high."
6<br>3<br>web	(Debele, Guru, Hundessa, & Duguma, 2013)	Ethiopia	Cross-sectional Survey<br>66 farmers<br>Smallholders	PPR, Copper deficiency, Sheep Pox, parasites	**Perceptions:** Eighty-eight percent of interviewed farmers felt there was a “high” pre-weaning mortality rate for small ruminants.<br>**Impact:** PPR, copper deficiency, Sheep Pox and parasites mentioned as main issues in study area although it is not clear how this conclusion was reached.
7<br>3<br>web	(Agegnehu, Bogale, Tesfaye, & Dagnachew, 2018)	Ethiopia	Cross-sectional, Skin scraping examination, 384 small ruminants<br>Agro-pastoralists	Mange	**Incidence/ Prevalence:** 27% positive for mange mite infestation (105/384).
8<br>2	(Gitonga, Gachene,	Kenya	Cross-sectional Purposive sample of two counties	Helminthiasis/ diarrhoea in young,	**Perceptions:** Prevalent sheep diseases were helminthiasis/ diarrhoea in young (ranked first by 32% of pastoralists), pneumonia (25%), and SGP

GALVmed

Protecting Livestock – Improving Human Lives

web	Njoroge, & Thumbi, 2016)		Survey of 67 pastoralists	Pneumonia, SGP, CCPP	(25%). Prevalent goat diseases were CCPP (35%) and helminthiasis/ diarrhoea in young (24%).
9<br>3<br>web	(Zvinorova et al., 2017)	Zimbabwe	Cross-sectional Survey<br>135 households<br>Smallholders	Gastrointestinal nematodes, internal parasites, pulpy kidney, skin problems, tick-borne diseases	**Perceptions:** The survey demonstrated that the level of awareness in gastrointestinal nematode disease, transmission methods, and control was very low.<br>**Incidence/ Prevalence:** Goat farmers reported internal parasites (90%), pulpy kidney (63%), skin problems (47%), tick-borne diseases (41%), orf (28%), and eye problems (24%).
10<br>2<br>web	(P. R. Bessell et al., 2018)	Tanzania & India	Randomized control trial of effect of antihelminthics on weight gain, 487 goats, Smallholders	Helminths	**Impact:** Goats in groups treated with local available antihelminthics in India gained a mean of 25.22 g per day more than non-treated goats. In Tanzania treated goats gained a mean of 9.878 g per day more than non-treated goats.
11<br>3<br>web	(Shabani et al., 2015)	Tanzania	Cross-sectional, Questionnaire, 463 community members, Pastoralists	Rift Valley fever (RVF)	**Impact:** Ninety percent (90%) agreed that RVF posed a threat to public health as well as the local livestock economy (5 point Likert scale).<br>**Perceptions:** Most of the respondents (97.6 %) had heard about RVF. Overall knowledge regarding RVF vector, modes of transmission, symptoms and prevention among respondents was low, with only 11.3% recorded as knowledgeable.

12<br>2<br>web	(Nyakarahuka et al., 2018)	Uganda	Abattoir study, Serosurvey of humans (655) and cattle, goats, sheep (1,051)	RVF	**Incidence/ prevalence:** The overall animal seropositivity was 13%, with 27% of cattle, 7% of goats, and 4% of sheep seropositive.
13<br>3<br>web	(Nanyingi et al., 2017)	Kenya	Cross-sectional, Serosurvey, 370 cattle, sheep, and goats, Purposive selection of RVF-prone area, Agro-pastoralists	RVF	**Incidence/ Prevalence:** Sheep, cattle, and goats had seroprevalences of 32.2% (95% CI [20.6–31]), 33.3% (95% CI [6.7–60]), and 25.8% (95% CI [22.4–42]), respectively.
14<br>2<br>web	(Blomström et al., 2016)	Mozambique	RVF	RVF	**Incidence/ Prevalence:** 37.5% (95% CI 29.8 - 39.5) of 368 analysed serum samples were positive for RVFV antibodies.
15<br>2<br>web	(Fafetine et al., 2013)	Mozambique	Cross-sectional, Serosurvey, Smallholders/ Pastoralists, 449 serum samples from goats and 313 from sheep	RVF	**Incidence/ Prevalence:** Seroprevalence estimates were 9.2% in sheep and 11.6% in goats.
16	(Ashenafi, Teshale, Agga,	Ethiopia	Cross-sectional, Serosurvey,	Brucellosis	**Incidence/ Prevalence:** The seroprevalence of *Brucella* infection was found to be 5.8% (n = 58) in

2<br>web	Fikru, & Laikemariam, 2016)		Smallholders/ Pastoralists, 1,568 serum (563 from sheep, 1,005 from goats)		goats and 3.2% (n = 18) in sheep. A prevalence rate of 5.3% was observed in adult animals and 1.6% in younger sheep and goats.
17<br>3<br>web	(Maitho & Kinyua, 2015)	Kenya	Cross-sectional Survey<br>170 farmers<br>Smallholders<br>Dairy	Diarrhoea<br>Eye infection<br>Pneumonia<br>Bloat<br>Mastitis<br>Heartwater<br>CCPP	**Incidence/ Prevalence:** 53% of the respondents had encountered diseases in their dairy goat flocks within one year. The important diseases found in the district included diarrhoea, eye infections, pneumonia, bloat, mastitis, heartwater and contagious caprine pleuropneumonia (CCPP).
18<br>3<br>web	(Eshetu, Yigezu, & Asfaw, 2007)	Ethiopia	Cross-sectional, Serosurvey, Abattoir study, 300 goats, Multiple production systems	CCPP	**Incidence/ Prevalence:** 31% of samples tested positive for contagious caprine pleuropneumonia antibodies.
19<br>2<br>web	(Fentie et al., 2017)	Ethiopia	Cross-sectional, Seroprevalence, Retrospective temporal and spatial analyses, 672 serum samples of goats and sheep	Sheep and goat pox	**Incidence/ Prevalence:** 15.5% of serum samples (104/672) tested positive for sheep and goat pox virus antibody, from which 17% (56) were sheep and 14% (48) were goats.<br>**Impact:** From January 2010 to December 2014, a total of 366 outbreaks, 12,822 cases and 1,480 deaths due to sheep pox and 182 outbreaks, 10,066

					cases and 997 deaths due to goat pox were recorded in Amhara National Regional State.
20 2 web	(Musinguzi et al., 2016)	Zambia	Cross-sectional, PCR-based survey, Pastoralists, 472 cattle and 53 goats	Animal African trypanosomiasis (AAT), Anaplasmosis, Theileriosis, Babesiosis	**Incidence/ Prevalence:** Hemoparasites detected in cattle and goats were of the genera Theileria, Anaplasma, and Trypanosoma with a prevalence of 15.1%, 1.6%, and 4.3% respectively in cattle, and 10%, 6.0%, and 0.0%, respectively in goats.
21 2 web	(Weny et al., 2017)	Uganda	Cross-sectional, Serosurvey, 186 cattle and 317 goats from 40 households, Pastoralists	AAT, Anaplasmosis, Theileriosis, Babesiosis	**Incidence/ Prevalence:** Hemoparasite infection prevalence of 16.4% in goats. Prevalence of specific genera of hemoparasites in goats was: *Theileria*, 10.4%; *Trypanosoma*, 0.0%; and *Anaplasma* 5.68%.
22 2 web	(Muema et al., 2017)	Kenya	Cross-sectional, Serosurvey, 508 goats and 332 sheep from 104 households, Agro-pastoralists, Pastoralists	Q fever	**Incidence/ Prevalence:** County chosen after human outbreaks of Q fever in 2014. Overall ruminant seroprevalence was 20.5% (95% CI: 17.8%, 23.3%). Goats had 26.0% (95% CI: 22.2%, 30.0%) compared to sheep 12.2% (95% CI: 8.7%, 16.0%). Nomadic pastoralism, goats and older animals (>1 year) were associated with greater risk of *C. burnetii* seropositivity (P = ≤0.05).
23 2	(Malak et al., 2012)	South Sudan	Cross-sectional, Participatory epidemiology,	Diarrhoea	**Perceptions:** In Yei county, diarrhoea in small ruminants was reported by farmers as the most important livestock disease.

web			Seroprevalence for cattle, Pastoralists?		
24<br>2<br>web	(Wesonga et al., 2006)	Kenya	Cross-sectional Seroprevalence 296 ruminants Pastoralists	Heartwater	**Incidence/ Prevalence:** Sero-prevalence of *E. ruminantium* ranged from 62-83% in sheep and 43-52% in goats.<br>**Solution:** A regular tick control program will prevent susceptible animals from getting infected with heartwater.
25<br>2<br>web	(Getaw et al., 2010)	Ethiopia	Cross-sectional, Abattoir study, Retrospective data analysis, 1,152 ruminants (cattle, sheep, goats)	Hydatidosis	**Incidence/ Prevalence:** Hydatidosis was prevalent in 47% of cattle, 29% of sheep, and 7% of goats. In sheep, hydatid cysts were recovered from 22 (55%) of the lung, 16 (40%) of the liver and 2 (5%) of the spleen while none of the heart and kidney were recorded positive. In goats, the degree of infection was 6 (33%) of lung, 10 (56%) of liver, 1 (6%) of spleen and kidney each.<br>**Impact:** The total annual economic loss incurred due to condemnation of organ meat during inspection due to hydatidosis in ruminants slaughtered at Adama municipal abattoir was estimated to be to $5,870 USD.
26<br>2<br>web	(Nonga & Karimuribo, 2009)	Tanzania	Longitudinal, 99,401 sheep/ goats, Abattoir study,	Hydatidosis	**Incidence/ Prevalence:** 6% infection rate of Cystic echinococcosis (CE) in sheep/ goats.

			Multiple production systems		
27<br>2<br>web	(Romha et al., 2018)	Ethiopia	Systematic literature review of epidemiology of *M. bovis* and *M. tuberculosis* in livestock	Tuberculosis (TB)	**Incidence/ Prevalence:** PPD reactors (positive TB skin test) in small ruminants occur in low prevalence ranging from 0.6% to 7.6%. The main strain was found to be SB1176.
28<br>3<br>web	(Hiko & Agga, 2011)	Ethiopia	Cross-sectional, Abattoir study, Multiple production systems, 1,536 goats	Tuberculosis	**Incidence/ Prevalence:** The overall slaughterhouse prevalence of caprine tuberculosis based on detailed post mortem examination was 4.2% in apparently healthy male goats. 20 samples were confirmed mycobacterium positive on culture, out of which 18 were *Mycobacterium bovis* and two were *Mycobacterium tuberculosis***.** "To the best of our knowledge, this is the first report of caprine tuberculosis from Ethiopia."
29<br>2<br>web	(Woldemeskel & Mersha, 2010)	Ethiopia	Cross-sectional, Prevalence study, 2,560 sheep and goats, Agro-pastoralists	Dermatophilosis	**Incidence/ Prevalence:** Out of 2,560 examined animals, 55 (2.14%) had clinical dermatophilosis. The respective prevalence in sheep and goats were 1.5% and 2.9%.
30<br>2	(Thumbi et al., 2015)	Kenya	Longitudinal, Syndromic surveillance, 1500 households, Smallholder	Syndromes	**Incidence/ Prevalence:** Of the illness cases reported among each livestock species, gastro-intestinal tract syndrome cases were the most common, comprising of 52%, 53%, and 34% of total cases in cattle, goats, and sheep respectively. For these

web					three species, respiratory syndromes were the second most commonly observed.
31<br>3<br>web	(Amenu, Szonyi, Grace, & Wieland, 2017)	Ethiopia	Cross-sectional, Qualitative methods, Participatory epidemiology, 40 female pastoralists	Udder health problems, Mastitis	**Perceptions:** It was stated that udder health problems are common in cows compared with goats.
32<br>2<br>web	(Carlson et al., 2019)	Sub-Saharan Africa	Global incidence database of human, wildlife, and livestock anthrax cases from multiple sources spanning 71 countries from 1914-2018	Anthrax	**Incidence/ Prevalence:** Using past occurrence as a proxy for risk, in Sub-Saharan Africa 30.5 million cattle, 22.4 million goats, and 14.5 million sheep are at risk.<br>**Impact:** Vaccination rates in Sub-Saharan Africa are 0-6%.
33<br>3<br>web	(Gebremedhin et al., 2013)	Ethiopia	Cross-sectional, Serosurvey, Agro-pastoralists, 1,130 sheep from 227 households	Ovine toxoplasmosis	**Incidence/ Prevalence:** Overall seroprevalence of 70.48% (160/227) at flock level and 31.59% (357/1,130) at animal level.
34<br>2<br>web	(Assenga et al., 2015)	Tanzania	Cross-sectional, Serosurvey, Agro-pastoralists,	Leptospirosis	**Incidence/ prevalence:** The prevalence of leptospiral antibodies was 30% in cattle, 8% in goats, and 30% in humans.

			1,103 cattle, 248 goats, 267 humans		
35<br>3<br>web	(Gelaye et al., 2013)	Ethiopia	Cross-sectional, Outbreak intectigation, Pastoralist	Sheep-associated malignant catarrhal fever in cattle	**Incidence/ prevalence:** First report of a diagnostic investigation resulting in the detection of ovine OvHV-2 in cattle and confirming the existence of sheep-associated malignant catarrhal fever in Ethiopia.<br>**Impact:** Disease outbreak caused morbidity and mortality in 18 cattle out of a total of more than 2,000 kept by farmers in the village and surrounding area.

Summary of articles

Hailu, B., Alemayehu, G., & Sied, N. (2015). Participatory epidemiological studies of major trade constraint diseases of goats in selected districts of Afar region. *Journal of Biology, Agriculture and Healthcare*, *5*(11), 140–148.

Qualitative study of goat and sheep diseases that constrain trade for pastoralists in Ethiopia. Methods included semi-structured interviews with 291 pastoralists, 12 focus groups, and interviews with stakeholders including veterinarians and four quarantine centres.

- Pastoralists motioned PPR, Pasteurellosis, goat pox, External parasites and CCPP as most important diseases in terms of impact on livelihoods. Quarantine centres listed PPR, pasteurellosis, CCPP, goat pox and external parasites most important diseases in terms of impact on businesses.
- Animal characteristics in terms of health, quality and other criteria required by exporters were known only by 8% of the producers. Afars' sheep are the most demanded by exporters next to Somali black head, but sheep account for only 20% of all small ruminant sales.
- Occurrence of such diseases impacts livestock producers by marginalizing them from higher price livestock markets and restricting their capacity for value-added trade.

Abdilatif, M. H., Onono, J. O., & Mutua, F. K. (2018). Analysis of pastoralists' perception on challenges and opportunities for sheep and goat production in Northern Kenya. *Tropical Animal Health and Production*, *50*(7), 1701–1710. https://doi.org/10.1007/s11250-018-1613-8

Mixed methods study of pastoralists raising sheep and goats in two purposively chosen sub-counties in northern Kenya.

- Pastoralists ranked diseases amongst sheep and goats by impact to livelihood as follows: PPR, CCPP, Goat Pox, TBD, pneumonia, mange, helminths (mentioned by all 16 focus groups). Rarely mentioned were diarrhoea in young goats, increased mortality, orf, and abortion/still births.
- PPR was considered a relatively new disease, only having been introduced within the last 10 years. The disease was said to have been introduced by herders who crossed borders (Somalia and Ethiopia) in search of water and pasture.
- Vaccination perceived to be most effective disease control strategy.

Faris, D., Yilkal, A., Berhe, G., & Kelay, B. (2012). Seroprevalence and sero-conversion after vaccination against Peste des Petits Ruminants in sheep and goats from Awash Fentale District, Afar, Ethiopia. *Preventive Veterinary Medicine*, *103*(2/3), 157–162.

Seroprevalence and sero-conversion after vaccination against PPR with a thermolabile vaccine was conducted in a pastoral community in Ethiopia. Sera was collected from 1239 unvaccinated sheep and goats and 1096 small ruminants were resampled from the vaccinated population fourteen days after vaccination.

- Post-vaccination sero-conversion rate was 61%, indicating weak herd immunity.
- No statistically significant difference in vaccination by veterinary professionals and CAHWs, Community Animal Health Workers.
- A thermostable vaccine formulation could potentially increase herd immunity. CAHWs should participate in vaccination campaigns, especially in rural areas such as Afar with few veterinary professionals.

Bett, B., Jost, C., Allport, R., & Mariner, J. (2009). Using participatory epidemiological techniques to estimate the relative incidence and impact on livelihoods of livestock diseases amongst nomadic pastoralists in Turkana South District, Kenya. *Preventive Veterinary Medicine*, *90*(3–4), 194–203. https://doi.org/10.1016/j.prevetmed.2009.05.001

Participatory epidemiology techniques with pastoralists, key informants, and veterinary experts in 32 randomly selected sub-locations (smallest administrative unit). Focus groups with at least 10 participants conducted in each sub-location. *Note:* Relative scores are achieved by pastoralists ranking diseases and should not be confused with epidemiology terms.

- For goats, the overall relative incidence scores of peste des petits ruminants (PPR), contagious caprine pleuropneumonia (CCPP) and mange were 23.5% (15, 34), 25% (21, 45) and 20% (19, 28), respectively (10th and 90th percentiles in parentheses).
- The respective median scores for case fatality rates were 66% (45, 76.5), 62.5% (25, 100) and 73.2% (21.4, 85.7).
- Disease impact matrix scores indicated that mange was the most important disease of goats

Chazya, R., Muma, J. B., Mwacalimba, K. K., Karimuribo, E., Mkandawire, E., & Simuunza, M. (2014). A Qualitative Assessment of the Risk of Introducing Peste des Petits Ruminants into Northern Zambia from Tanzania. *Veterinary Medicine International*, *2014*, 1–10. https://doi.org/10.1155/2014/202618

A qualitative risk assessment was performed to evaluate the risk of introducing Peste des petits ruminants (PPR) virus into northern
Zambia from Tanzania via live goat trade. Methods included literature review, expert interviews, and risk modelling.

- The probability of occurrence of PPRV in northern Zambia from Tanzania via live goat trade was rated as "high" and the economic consequences were also rated as "high." Finally, the overall risk of introducing PPRV into northern Zambia from Tanzania at the time of the assessment was rated "high."

Debele, G., Guru, M., Hundessa, F., & Duguma, M. (2013). Assessment of farmers' management practices and factors affecting goats' production system in Adami Tulu Jido Kombolcha district of East Shawa Zone, Ethiopia. *Agriculture and Biology Journal of North America*, *4*(5), 520–526.

Cross-sectional survey of 66 goat farmers randomly selected from within two administrative districts in Ethiopia and asked about their management practices for keeping goats.

- Main issues mentioned within study area are PPR, copper deficiency, Sheep Pox and parasites although it is not clear how this conclusion was reached.
- Eighty-eight percent of interviewed farmers felt there was a "high" pre-weaning mortality rate for small ruminants.

Agegnehu, A., Bogale, B., Tesfaye, S., & Dagnachew, S. (2018). Status of mange infestation in indigenous sheep and goats and their control practices in Wag-Himra zone, Ethiopia. *Journal of Veterinary Medicine and Animal Health*, *10*(5), 128–134.

Cross-sectional survey of 120 sheep and 264 goats across three agro-ecological zones in Ethiopia.

- Skin scraping examinations showed 27% of the small ruminants were positive for mange mite infestation (105/384).
- 87% of farmers reported using modern acaricides to treat mange. The study does not examine whether farmer who report acaricide use were less likely to animals positive for mange mite infection.

Gitonga, P. N., Gachene, C. K., Njoroge, E., & Thumbi, S. M. (2016). Small ruminant husbandry practices amongst Kajiado and Marsabit pastoralists and their effects on Peste des petits ruminants control strategies. *Livestock Research for Rural Development*, *28*(2).

Cross-sectional survey of 67 pastoralists in two purposively chosen counties in Kenya with goal of learning about small ruminant husbandry practices and preferred animal health interventions.

- Prevalent sheep diseases were helminthiasis/ diarrhoea in young (ranked first by 32% of pastoralists), pneumonia (25%), and SGP (25%). Prevalent goat diseases were CCPP (35%) and helminthiasis/ diarrhoea in young (24%).

- 49% of pastoralists indicated lack of quality drugs (anthelmintics) was the major constraint in disease control yet 83% preferred regular veterinary services as an intervention rather than drug outlets or improved markets.

Zvinorova, P. I., Halimani, T. E., Muchadeyi, F. C., Katsande, S., Gusha, J., & Dzama, K. (2017). Management and control of gastrointestinal nematodes in communal goat farms in Zimbabwe. *Tropical Animal Health and Production*, *49*(2), 361–367.

Cross-sectional survey of 135 goat farmers in Zimbabwe across five agro-ecological zones with focus on gastrointestinal nematodes.

- Goat farmers reported internal parasites (90%), pulpy kidney (enterotoxaemia) (63%), skin problems (47%), tick-borne diseases (41%), orf (28%), and eye problems (24%).
- The survey demonstrated that the level of awareness in gastrointestinal nematode disease, transmission methods, and control was very low. Majority of the farmers were not treating their goats and some were using non-anthelmintic drugs to treat infections.

Bessell, P. R., Sargison, N. D., Mirende, K., Dash, R., Prasad, S., Al-Riyami, L., Wambura, P. (2018). The impact of anthelmintic drugs on weight gain of smallholder goats in subtropical regions. *Preventive Veterinary Medicine*, *159*, 72–81. https://doi.org/10.1016/j.prevetmed.2018.08.014

Randomized control trial of 234 goats from 92 smallholder households from Odisha state in India and 253 goats from 15 households from Dodoma region in Tanzania to test the effect of antihelminthics on weight gain over 56 days.

- Goats in groups treated with local available antihelminthics in India gained a mean of 25.22 g per day more than non-treated goats. In Tanzania treated goats gained a mean of 9.878 g per day more than non-treated goats.

Shabani, S. S., Ezekiel, M. J., Mohamed, M., & Moshiro, C. S. (2015). Knowledge, attitudes and practices on Rift Valley fever among agro pastoral communities in Kongwa and Kilombero districts, Tanzania. *BMC Infectious Diseases*, *15*(1), 363. https://doi.org/10.1186/s12879-015-1099-1

Cross-sectional survey focused on knowledge, attitudes and practices relating to Rift Valley fever among members of agropastoral communities in Kongwa and Kilombero districts, Tanzania. Community members (463) responded to a questionnaire.

- Ninety percent (90%) agreed that RVF posed a threat to public health as well as the local livestock economy (5 point Likert scale).

- Most of the respondents (97.6 %) had heard about RVF. Overall knowledge regarding RVF vector, modes of transmission, symptoms and prevention among respondents was low, with only 11.3% recorded as knowledgeable.

Nyakarahuka, L., de St. Maurice, A., Purpura, L., Ervin, E., Balinandi, S., Tumusiime, A., Shoemaker, T. R. (2018). Prevalence and risk factors of Rift Valley fever in humans and animals from Kabale district in Southwestern Uganda, 2016. *PLOS Neglected Tropical Diseases*, *12*(5), e0006412. https://doi.org/10.1371/journal.pntd.0006412

Following the diagnosis of two acute human Rift Valley fever cases in Kabale district, Uganda, serosurvey of 655 humans and 1,051 livestock (cattle, sheep, and goats) were conducted to estimate RVFV seroprevalence in humans and livestock and to identify associated risk factors. Samples were collected at abattoirs and villages in Kabale district.

- The overall animal seropositivity was 13%, with 27% of cattle, 7% of goats, and 4% of sheep seropositive.
- 78% of humans tested positive for anti RVFV antibodies.

Nanyingi, M. O., Muchemi, G. M., Thumbi, S. M., Ade, F., Onyango, C. O., Kiama, S. G., & Bett, B. (2017). Seroepidemiological Survey of Rift Valley Fever Virus in Ruminants in Garissa, Kenya. *Vector-Borne and Zoonotic Diseases*, *17*(2), 141–146. https://doi.org/10.1089/vbz.2016.1988

Cross-sectional serosurvey of 370 cattle, goats, and sheep for Rift Valley fever in eight purposively selected RVF-prone areas of Garissa county, Kenya.

- Sheep, cattle, and goats had seroprevalences of 32.2% (95% CI [20.6–31]), 33.3% (95% CI [6.7–60]), and 25.8% (95% CI [22.4–42]), respectively.

Blomström, A.-L., Scharin, I., Stenberg, H., Figueiredo, J., Nhambirre, O., Abilio, A., Fafetine, J. (2016). Seroprevalence of Rift Valley fever virus in sheep and goats in Zambézia, Mozambique. *Infection Ecology & Epidemiology*, *6*(1), 31343. https://doi.org/10.3402/iee.v6.31343

Cross-sectional study to investigate the seroprevalence of Rift Valley fever virus in sheep and goats in Zambézia, Mozambique. Blood samples were collected from 187 goats and 181 sheep in eight localities of three districts and analysed for the presence of antibodies against RVFV using a commercial competitive ELISA.

- 37.5% (95% CI 29.8 - 39.5) of 368 analysed serum samples were positive for RVFV antibodies.
- The overall seroprevalence in sheep was significantly higher than in goats ($p < 0.001$), with 44.2 and 25.1% seropositive animals, respectively.

- The highest seroprevalence for both goat and sheep was observed in adult animals (> 12 months).
- The high level of RVFV seroprevalence in small ruminants in Zambézia suggests viral circulation, even without reports of clinical signs in susceptible hosts.

Fafetine, J., Neves, L., Thompson, P. N., Paweska, J. T., Rutten, V. P. M. G., & Coetzer, J. A. W. (2013). Serological Evidence of Rift Valley Fever Virus Circulation in Sheep and Goats in Zambézia Province, Mozambique. *PLoS Neglected Tropical Diseases*, *7*(2), e2065. https://doi.org/10.1371/journal.pntd.0002065

A cross-sectional survey to estimate the prevalence of Rift Valley fever in sheep and goats in smallholder and pastoral households in Mopeia and Nicoadala districts in Zambézia Province, Mozambique. Serum samples from 449 goats and 313 sheep were collected and analysed.

- Seroprevalence was estimated as 9.2% in sheep and 11.6% in goats.
- The presence of antibodies to RVFV in sheep and goats in different districts of Zambézia Province is evidence of inter-epidemic circulation of RVFV with mild or subclinical manifestation.

Ashenafi, F., Teshale, S., Agga, G., Fikru, R., & Laikemariam, Y. (2016). Distribution of brucellosis among small ruminants in the pastoral region of Afar, eastern Ethiopia. *Revue Scientifique et Technique de l'OIE*, *26*(3), 731–739. https://doi.org/10.20506/rst.26.3.1781

A cross-sectional study was conducted in the pastoral region of Afar, in eastern and central Ethiopia, to determine the distribution of brucellosis in small ruminants. Serum samples were collected from 563 sheep and 1,005 goats.

- The seroprevalence of *Brucella* infection was found to be 5.8% (n = 58) in goats and 3.2% (n = 18) in sheep.
- A prevalence rate of 5.3% was observed in adult animals and 1.6% in younger sheep and goats.

Maitho, T., & Kinyua, J. W. (2015). Factors and diseases influencing dairy goats production among small scale farmers in Laikipia East District, Kenya. *International Journal of Livestock Research*, *5*(12), 43–48.

Small scale dairy goat farmers surveyed to establish factors and disease that limit production within the district.

- 53% of the respondents had encountered diseases in their dairy goat flocks within one year.

- The important diseases found in the district included diarrhoea, eye infections, pneumonia, bloat, mastitis, heartwater, and Contagious Caprine Pleural Pneumonia.

Eshetu, L., Yigezu, L., & Asfaw, Y. (2007). A study on contagious caprine pleuropneumonia (CCPP) in goats at an export oriented abattoir, Debrezeit, Ethiopia. *Tropical Animal Health and Production*, *39*(6), 427–432.

Cross-sectional survey using a convenience sample of 300 goats at an abattoir in Ethiopia.

- 31% of samples tested positive for contagious caprine pleuropneumonia antibodies.

Fentie, T., Fenta, N., Leta, S., Molla, W., Ayele, B., Teshome, Y., Assefa, A. (2017). Sero-prevalence, risk factors and distribution of sheep and goat pox in Amhara Region, Ethiopia. *BMC Veterinary Research*, *13*(1), 385. https://doi.org/10.1186/s12917-017-1312-0

A cross-sectional study design was used to estimate the sero-prevalence and identify associated risk factors of sheep and goat pox in Amhara Region, Ethiopia, while retrospective study design was used to assess the temporal and spatial distribution of the disease. A total of 672 serum samples were collected from 30 Kebeles and tested using virus neutralization test.

- 15.5% of serum samples (104/672) tested positive for sheep and goat pox virus antibody, from which 17% (56) were sheep and 14% (48) were goats.
- From January 2010 to December 2014, a total of 366 outbreaks, 12,822 cases and 1,480 deaths due to sheep pox and 182 outbreaks, 10,066 cases and 997 deaths due to goat pox were recorded in Amhara National Regional State.

Musinguzi, S., Suganuma, K., Asada, M., Laohasinnarong, D., Sivakumar, T., Yokoyama, N., Inoue, N. (2016). A PCR-based survey of animal African trypanosomosis and selected piroplasm parasites of cattle and goats in Zambia. *Journal of Veterinary Medical Science*, *78*(12), 1819–1824. https://doi.org/10.1292/jvms.16-0240

In this study, the prevalence of AAT and babesiosis in the three districts of Chama, Monze and Mumbwa in Zambia was determined using PCR-based methods. A total of 472 cattle from Chama (n=292), Mumbwa (n=96) and Monze (n=84) and 53 goats (all from Chama) were examined for the presence of animal African trypanosomes (*Trypanozoon, T. congolense and T. vivax), B. bigemina* and *T. parva*.

- Hemoparasites detected in cattle and goats were of the genera Theileria, Anaplasma, and Trypanosoma with a prevalence of 15.1%, 1.6%, and 4.3% respectively in cattle, and 10%, 6.0%, and 0.0%, respectively in goats.
- Although babesiosis is recognized as an economically important disease, unlike theileriosis and trypanosomiosis, its control and eradication have not been a priority.

- Results showed that goats can be an important reservoir for trypanosomes and that the treatment of goats could lead to a reduction in the incidence of trypanosomiosis in Zambia and other African countries.

Weny, G., Okwee-Acai, J., Okech, S. G., Tumwine, G., Ndyanabo, S., Abigaba, S., & Goldberg, T. L. (2017). Prevalence and Risk Factors Associated with Hemoparasites in Cattle and Goats at the Edge of Kibale National Park, Western Uganda. *Journal of Parasitology*, *103*(1), 69–74. https://doi.org/10.1645/16-33

Cross-sectional study of 186 cattle and 317 goats from 40 pastoralist households in villages bordering Kibale National Park in Western Uganda area to assess the prevalence and risk factors associated with hemoparasites causing African Animal Trypanosomiasis, anaplasmosis, and theileriosis.

- Hemoparasite infection prevalence of 16.4% in goats (12.9% in communities at the edge and 2.2% in control communities).
- Prevalence of specific genera of hemoparasites in goats was: *Theileria*, 10.4%; *Trypanosoma*, 0.0%; and *Anaplasma* 5.68%.
- Cattle were more likely than goats to be infected with *Theileria*, whereas goats were more likely than cattle to be infected with *Anaplasma.*
- *Trypanosoma* was not detected in goats in any location
- Wildlife–livestock interface is a high-risk area for hemoparasite infection.

Muema, J., Thumbi, S. M., Obonyo, M., Wanyoike, S., Nanyingi, M., Osoro, E., Karanja, S. (2017). Seroprevalence and Factors Associated with *Coxiella burnetii* Infection in Small Ruminants in Baringo County, Kenya. *Zoonoses and Public Health*, *64*(7), e31–e43. https://doi.org/10.1111/zph.12342

Serosurvey of *Coxiella burnetii* infection (Q fever) in 508 goats and 332 sheep from 104 households in Baringo County, Kenya following human outbreaks of Q fever in 2014.

- Overall ruminant seroprevalence was 20.5% (95% CI: 17.8%, 23.3%).
- Goats had 26.0% (95% CI: 22.2%, 30.0%) compared to sheep 12.2% (95% CI: 8.7%, 16.0%).
- Nomadic pastoralism, goats and older animals (>1 year) were associated with greater risk of *C. burnetii* seropositivity (P = ≤0.05).

Malak, A. K., Mpoke, L., Banak, J., Muriuki, S., Skilton, R. A., Odongo, D., Kiara, H. (2012). Prevalence of livestock diseases and their impact on livelihoods in Central Equatorial State, southern Sudan. *Preventive Veterinary Medicine*, *104*(3–4), 216–223. https://doi.org/10.1016/j.prevetmed.2011.12.001

Seroprevalence and questionnaire administered to farmers (pastoralists?) in two purposively selected counties in South Sudan reported to have a high burden of tick-borne disease. Some analyses done only for one county. Questionnaire covered small ruminants but seroprevalence was cattle only.

- In Yei county, diarrhoea in small ruminants was reported by farmers as the most important livestock disease.

Wesonga, F. D., Wesongah, J. O., Chemuliti, J., Wanjala, K., Munga, L., & Gitau, P. (2006). Seroprevalence of Ehrlichia Ruminantium (Heartwater) in small ruminants in a pastoral production system in Narok District, Kenya. *Bulletin of Animal Health and Production in Africa*, *54*(1), 23–33.

Presence of heartwater was measured in Narok district with a cross-sectional survey of 147 sheep and 149 goats. The sera were tested for antibodies to *Ehrilichia ruminantium* by a MAP1 B ELISA.

- Sero-prevalence of *E. ruminantium* ranged from 62-83% in sheep and 43-52% in goats.
- A regular tick control program will prevent susceptible animals from getting infected with heartwater.

Getaw, A., Beyene, D., Ayana, D., Megersa, B., & Abunna, F. (2010). Hydatidosis: Prevalence and its economic importance in ruminants slaughtered at Adama municipal abattoir, Central Oromia, Ethiopia. *Acta Tropica*, *113*(3), 221–225. https://doi.org/10.1016/j.actatropica.2009.10.019

A cross-sectional study was conducted from November 2007 to April 2008 to estimate the prevalence of hydatidosis in ruminants slaughtered at Adama municipal abattoir in Ethiopia with the goal of estimating the annual economic loss due to condemnation of organs during meat inspection. A total of 1,152 ruminants (852 cattle, 92 sheep and 208 goats) were inspected following slaughter.

- Hydatidosis was prevalent in 47% of cattle, 29% of sheep, and 7% of goats.
- In goats, the degree of infection was 6 (33%) of lung, 10 (56%) of liver, 1 (6%) of spleen and kidney each.
- In sheep, hydatid cysts were recovered from 22 (55%) of the lung, 16 (40%) of the liver and 2 (5%) of the spleen while none of the heart and kidney were recorded positive.
- The total annual economic loss incurred due to condemnation of organs during meat inspection due to hydatidosis in ruminants slaughtered at Adama municipal abattoir was estimated to be to $5,870 USD.

Nonga, H. E., & Karimuribo, E. D. (2009). A retrospective survey of hydatidosis in livestock in

Arusha, Tanzania, based on abattoir data during 2005 - 2007. *Tropical Animal Health and Production*, *41*(7), 1253–1257. https://doi.org/10.1007/s11250-009-9308-9

Retrospective study of hydatidosis based on abattoir data from 2005-2007 in Arusha, Tanzania. A total of 115,186 cattle and 99,401 sheep and goats were investigated for cystic echinococcosis (CE).

- 6.0% infection rate in goats/ sheep and 4.2% infection rate in cattle.
- No significant difference between infection rates in rainy and dry season.

Romha, G., Gebru, G., Asefa, A., & Mamo, G. (2018). Epidemiology of Mycobacterium bovis and Mycobacterium tuberculosis in animals: Transmission dynamics and control challenges of zoonotic TB in Ethiopia. *Preventive Veterinary Medicine*, *158*, 1–17. https://doi.org/10.1016/j.prevetmed.2018.06.012

A systematic literature review of articles published from 1998-2017 focusing on tuberculosis in Ethiopian livestock.

- PPD reactors (positive TB skin test) in small ruminants occur in low prevalence ranging from 0.6% to 7.6%.
- The main strain was found to be SB1176.

Hiko, A., & Agga, G. E. (2011). First-time detection of mycobacterium species from goats in Ethiopia. *Tropical Animal Health and Production*, *43*(1), 133–139. https://doi.org/10.1007/s11250-010-9665-4

Abattoir study with convenience sample of 1,536 randomly selected male goats in Ethiopia (no justification for selecting males only).

- The overall slaughterhouse prevalence of caprine tuberculosis based on detailed post mortem examination was 4.2% in apparently healthy male goats.
- 20 samples were confirmed mycobacterium positive on culture, out of which 18 were *Mycobacterium bovis* and two were *Mycobacterium tuberculosis*.
- "To the best of our knowledge, this is the first report of caprine tuberculosis from Ethiopia."

Woldemeskel, M., & Mersha, G. (2010). Study on caprine and ovine dermatophilosis in Wollo, Northeast Ethiopia. *Tropical Animal Health and Production*, *42*(1), 41–44. https://doi.org/10.1007/s11250-009-9383-y

Cross-sectional study of the prevalence of dermatophilosis in 2,560 sheep and goats in northeast Ethiopia. Production system is not specified but is likely agro-pastoralists.

- Out of 2,560 examined animals, 55 (2.1%) had clinical dermatophilosis.

- The respective prevalence in sheep and goats were 1.5% and 2.9%.
- There was no significant difference (p>0.05) in prevalence between sheep and goats and different sexes in both species.
- In goats, the prevalence in young (8.7%) was significantly (p<0.05) higher than in adults (2.3%).
- Clinical disease was associated with orf (present in 45% of sheep with dermatophilosis and 12% of goats), pox (22% in sheep and 18% in goats) and ticks in goats (36%, 12/33).

Thumbi, S. M., Njenga, M. K., Marsh, T. L., Noh, S., Otiang, E., Munyua, P., Palmer, G. H. (2015). Linking Human Health and Livestock Health : A "One-Health" Platform for Integrated Analysis of Human Health, Livestock Health, and Economic Welfare in Livestock Dependent Communities. *PLoS ONE*, *10*(3), 1–18. https://doi.org/10.1371/journal.pone.0120761

1500 rural households in Western Kenya were enrolled in a syndromic surveillance study covering human and animal syndromes. Data were collected bi-weekly.

- Of the illness cases reported among each livestock species, gastro-intestinal tract syndrome cases were the most common, comprising of 52%, 53%, and 34% of total cases in cattle, goats, and sheep respectively. For these three species, respiratory syndromes were the second most commonly observed.

Amenu, K., Szonyi, B., Grace, D., & Wieland, B. (2017). Important knowledge gaps among pastoralists on causes and treatment of udder health problems in livestock in southern Ethiopia: results of qualitative investigation. *BMC Veterinary Research*, *13*(1), 303. https://doi.org/10.1186/s12917-017-1222-1

Cross-sectional, qualitative study of udder health problems kept by 40 female pastoralists in four villages in southern Ethiopia. Participatory data collection methodologies included individual semi-structured in-depth interviews, focus group discussions, informal discussions with key informants, and observations.

- Udder health problems was classified locally as three types (1) tick infestation, (2) swelling of udder often with pus discharge, and (3) acute mastitis caused by evil eye with 'bloody milk.

Carlson, C. J., Kracalik, I. T., Ross, N., Alexander, K. A., Hugh-Jones, M. E., Fegan, M., Blackburn, J. K. (2019). The global distribution of Bacillus anthracis and associated anthrax risk to humans, livestock and wildlife. *Nature Microbiology*. https://doi.org/10.1038/s41564-019-0435-4

Global incidence database of human, wildlife, and livestock anthrax cases from multiple sources spanning 71 countries from 1914-2018.

- Using past occurrence as a proxy for risk, in Sub-Saharan Africa, an estimated 30.5 million cattle, 22.4 million goats, and 14.5 million sheep are at risk for anthrax.
- Vaccination rates in Sub-Saharan Africa are 0-6%.
- Vaccination may be less effective for the 31 million livestock and 4.6 million poor livestock keepers in West Africa, where an endemic lineage of *B. anthracis* shares an anthrose-deficiency mutation that has been hypothesized to lead to a vaccine escape.

Gebremedhin, E., Agonafir, A., Tessema, T., Tilahun, G., Medhin, G., Vitale, M., Dorny, P. (2013). Seroepidemiological study of ovine toxoplasmosis in East and West Shewa Zones of Oromia Regional State, Central Ethiopia. *BMC Veterinary Research*, *9*(1), 117. https://doi.org/10.1186/1746-6148-9-117

A cross-sectional study was conducted in three districts of Oromia regional state, Central Ethiopia in order to estimate the flock and individual animal level seroprevalence of ovine toxoplasmosis. Sample size was 1,130 sheep from 227 households.

- Out of 227 flocks tested 160 (70.48%) flocks were positive. Fifty-five (24.23%), 50(22.03%), 30 (13.22%), 14 (6.17%) and 11(4.85%) flocks of sheep had one, two, three, four and five seropositive animals, respectively.
- Overall animal level seroprevalence of *T. gondii* infection was 31.59%
- Seroprevalence was 4.30 times higher in midland and 3.97 times higher in highland compared to the lowland
- Study concluded that *T. gondii* infection of sheep at flock and animal levels in the study districts is high and that altitude, sex, age, flock size and source of water are independent predictors of *T. gondii* seropositivity.

Assenga, J. A., Matemba, L. E., Muller, S. K., Mhamphi, G. G., & Kazwala, R. R. (2015). Predominant leptospiral serogroups circulating among humans, livestock and wildlife in Katavi-Rukwa ecosystem, Tanzania. *PLoS Neglected Tropical Diseases*, *9*(3), e0003607. https://doi.org/10.1371/journal.pntd.0003607

A cross-sectional epidemiological study, to determine the seroprevalence of *Leptospira spp* in humans, domestic ruminants and wildlife in the Katavi region, Southwest Tanzania. Sample size included 1,103 cattle, 248 goats, and 267 humans.

- The prevalence of leptospiral antibodies was 30% in cattle, 8% in goats, and 30% in humans.
- Study identified similar leptospiral serogroups circulating in humans, domestic ruminants, wildlife, rodents and shrews sharing the same ecosystem.

Gelaye, E., Mekonnen, G., Jenberie, S., & Ayelet, G. (2013). Detection of sheep-associated malignant catarrhal fever from clinical cases in Ethiopian cattle. *Revue Scientifique et*

Technique de l'OIE, *32*(2), 851–856. https://doi.org/10.20506/rst.32.2.2211

Report of a diagnostic investigation cattle disease problem that occurred in the Arbe Gona district of southern Ethiopia. Samples were collected from recently deceased animals.

- First report of a diagnostic investigation resulting in the detection of ovine OvHV-2 in cattle and confirming the existence of sheep-associated malignant catarrhal fever in Ethiopia.
- Disease outbreak caused morbidity and mortality in 18 cattle out of a total of more than 2,000 kept by farmers in the village and surrounding area.
