# Supplementary material for: Prioritizing smallholder animal health needs in East Africa, West Africa, and South Asia using three approaches: Literature review, expert workshops, and practitioner surveys
Source: Prev Vet Med. 2021 Apr;189:105279. doi: 10.1016/j.prevetmed.2021.105279 (PMC8024747; doi:10.1016/j.prevetmed.2021.105279)
Supplement: Supplementary file 7 [file mmc7.pdf]

Meta-analysis results

Geographic location

East Africa had a larger body of literature for smallholder animal health needs than West Africa (315 articles and 101 articles, respectively), even when literature in French was included. Cattle were discussed in almost two-thirds of the articles (307/423), small ruminants in about a third (134/423), and poultry in about a tenth (53/423). Only four articles addressed animal health needs in all three livestock categories. The number of articles considered in the meta-analysis by geographic location and livestock type is shown in Table 1. Note that many articles mention multiple livestock types.

	East Africa	West Africa	All
Cattle	240 (67)	67 (25)	307 (92)
Small ruminants	100 (35)	34 (14)	134 (49)
Poultry	29 (12)	24 (10)	53 (22)

Table 1: Number of articles considered in the meta-analysis by addressed geographic location and livestock type. In parentheses is the number of articles focusing on impact that are summarized more fully.

Animal health concerns

Across East and West Africa and all livestock types, the animal health concerns that appeared most frequently (in 20 or more articles) were ectoparasites, brucellosis, foot-and-mouth disease (FMD), African Animal trypanosomiosis (AAT), endoparasites, tick-borne-disease, helminths, Peste des Petits Ruminants (PPR), mange, Newcastle disease (ND), and tuberculosis (TB) (See Table 2). The most frequent health concerns in East Africa (mentioned in 20 or more articles) are all represented on this list. The most frequent animal health concerns in West Africa (mentioned in 10 or more articles) are also all represented with the addition of avian influenza. Tuberculosis, while appearing frequently in the East Africa literature, is mentioned by only two articles in the West Africa literature. The full list of all animal health concerns for all livestock types is presented as Appendix 1.

Overall		East Africa		West Africa	
Ectoparasites	49	Ectoparasites	34	Brucellosis	14
Brucellosis	47	Brucellosis	32	Ectoparasites	13
Foot and mouth disease	45	Foot and mouth disease	32	Foot and mouth disease	13
Trypanosomiosis	36	Trypanosomiosis	27	Newcastle disease	11
Endoparasites	33	Tick-borne disease	26	Helminths	10
Tick-borne disease	31	Endoparasites	24	PPR	10
Helminths	31	Tuberculosis	22	Avian influenza	10
PPR	29	Helminths	20		
Mange	27	PPR	20		
Newcastle disease	27				
Tuberculosis	24				

Table 2: Animal health concerns by frequency of mention in literature review. The numbers correspond to the number of articles mentioning the respective animal health concern. (n=423)

Impact articles

The comparison of the broad meta-analysis with the meta-analysis of impact articles only shows a good deal of overlap but some key differences. Ectoparasites, brucellosis, and Newcastle disease top both lists. Foot and mouth disease drops in ranking in the meta-analysis of impact articles. African Animal Trypanosomiasis, while not high ranking in the overall literature review, is present in the impact article meta-analysis for both East and West Africa. Lumpy skin disease in East Africa was not in the most frequently mentioned meta-analysis concerns, but had the distinction of two green-ranking papers quantifying its economic impact for smallholders. Lastly, though avian influenza (AI) tops the impact article meta-analysis, the content of the articles suggest that while the cost of an avian influenza outbreak is high, it is not a top concern for farmers because it is rare. The high frequency of avian influenza articles is likely a response to outbreaks in West Africa. Many articles focusing on other poultry health concerns took advantage of the surveillance conducted as a response to AI outbreaks.

Overall		East Africa		West Africa	
Ectoparasites	15	Newcastle disease	10	Avian influenza	5
Brucellosis	15	Ectoparasites	9	Newcastle disease	4
Newcastle disease	14	PPR	9	Ectoparasites	3
Endoparasites	13	Brucellosis	7	Brucellosis	3
PPR	12	Endoparasites	7	FMD	3
FMD	11	Tick-borne disease	6	AAT	3
Helminths	11	FMD	5	Helminths	3
AAT	10	AAT	5	PPR	3
Tick-borne disease	9	Helminths	5		
RVF	9	TB	5		
		RVF	5		
		Anaplasmosis	5		
		Lumpy skin disease	5		

Table 3: Animal health concerns by frequency of mention in subset of impact articles. The numbers correspond to the number of articles mentioning the respective animal health concern. (n=163)

Appendix 1

Summary of animal health concerns for all livestock types

Animal health concern	Frequency of mention Overall	Frequency of mention East Africa	Frequency of mention West Africa
Ectoparasites	49	34	13
Brucellosis	47	32	14
FMD	45	32	13
AAT	36	27	9
Endoparasites	33	24	9
Tick-borne disease	31	26	5
Helminth	31	20	10
PPR	29	20	10
Mange	27	19	6
Newcastle disease	27	16	11
TB	24	22	2
RVF	19	15	4

Mastitis	19	17	2
Lumpy skin disease	19	19	0
Abortion	18	13	5
Anaplasmosis	17	15	2
CBPP	16	8	8
East Coast fever	15	15	1
IBD	14	10	4
Pasteurellosis	14	12	2
Avian influenza	12	2	10
CCPP	11	10	1
Blackquarter	11	10	1
Heartwater	11	6	5
Anthrax	10	9	1
Theileriosis	10	9	1
Diarrhea	9	6	3
Fascioliasis	9	4	3
Babesiosis	9	6	3
Q fever	8	5	3
Dermatophilosis	7	5	2
Goat pox	7	6	1
Fowl Pox	7	4	3
Chronic respiratory disease	6	3	3
Leptospirosis	5	5	0
Bovine viral diarrhea virus	5	5	0
Coccidiosis	5	4	1
Hydatidosis	5	4	1
Schistosomiasis	5	3	2
Sheep pox	5	5	0
Malignant catarrhal fever	5	5	0
Marek's disease	4	4	0
Cryptosporidiosis	4	4	0
Toxoplasmosis	4	4	0
Fowl cholera	4	2	2
Acaricide resistance	3	3	0
Aflatoxin	3	3	0
Bluetongue	3	3	0
Colibacillosis	3	3	0
Haemonchosis	3	2	1
Infectious bronchitis	3	1	2

Orf	3	3	0
Fowl typhoid	3	2	1
Salmonellosis	3	3	0
Bloat	2	1	1
Colisepticemia	2	1	1
Copper deficiency	2	2	0
Cystic echinococcosis	2	2	0
Cysticercosis	2	1	1
Dystocia	2	2	0
Mycoplasmosis	2	2	0
Neospora caninum infection	2	2	0
Reproductive disorder	2	2	0
Retention fetal membrane	2	2	0
Tetanus	2	1	1
Trypanocide resistance	2	2	0

Animal health concerns listed in only one article are listed below.

Actinomycosis
Acute haemorrhagic syndrome
Arbovirus
Arthritis
Bartonellosis
Besnoitiosis
Biting fly
Bovine herpesvirus
Bovine immunodeficiency virus
Bovine spongiform encephalopathy
Campylobacteriosis
Chicken anemia virus
Delayed heat period
Dermatophytosis
Enzootic bovine leucosis
Eye infections
Foetal wastage
Foot conditions
Giardia
Haemorrhagic septicaemia
Hemoparasite

Histophilosis
Infectious coryza
Johne's disease
Lameness
Parasitic gastroenteritis
Prolapsed uterus
Rinderpest
Schmallenberg virus
Skin problems
Tungiasis
Vaginal and uterine prolepses
Paramphistomiasis
