# Supplementary material for: Prioritizing smallholder animal health needs in East Africa, West Africa, and South Asia using three approaches: Literature review, expert workshops, and practitioner surveys
Source: Prev Vet Med. 2021 Apr;189:105279. doi: 10.1016/j.prevetmed.2021.105279 (PMC8024747; doi:10.1016/j.prevetmed.2021.105279)
Supplement: Supplementary file 8 [file mmc8.pdf]

Project objective

The goal of this systematic literature review is to understand the current state of knowledge regarding smallholder animal health needs and the impact of diseases and syndromes. More specifically, the review focuses on incidence/ prevalence of animal health concerns and their impact.

Methods

Literature search

A systematic literature review was conducted in order to understand the current state of knowledge regarding smallholder animal health needs and the impact of disease and syndromes. We conducted searches in PubMed, CAB Abstracts, and Web of Science databases using the criteria below which yielded 423 documents. A subset of 163 articles with focus on impact are summarized more extensively and presented in a table and as a brief synopsis. The majority were scholarly articles but conference proceedings and trade journals are also represented. All articles were tagged with location, livestock type, and animal health tags for a meta-analysis. The animal health tags were standardized based on health concerns that came up in the paper. Systematically tagging allows for consideration of secondary topics, for example survey respondents noting the importance of tick-borne disease in a study originally designed to investigate the impact of anthrax. We chose not to tag production systems in part because in many studies, there was not a clear way to differentiate between smallholders, agro-pastoralists, pastoralists, and small commercial or business-oriented producers. Given the importance of production system in interpreting results, we do note in within the summarized articles.

Meta-analysis

A meta-analysis of a broader collection of articles allows for consideration of studies that are somewhat relevant but do not focus on incidence, prevalence, or impact. An example might be a report of an intervention to control disease in a smallholder context. It suggests that the disease is important but does not directly quantify impact. The meta-analysis involved quantifying the number of articles that address a given health topic for the broad set of articles and for the subset of summarized, impact-focused articles.

Ranking

Summarized articles were given a ranking (green, yellow, red) designed to help a reader identify articles that are most relevant to the project interests of incidence/ prevalence of animal health concerns and their impact. For example, incidence/ prevalence includes disease

seroprevalence, outbreak incidence, number of cases, or percentage of self-reported disease. Impact includes mortality, morbidity, production losses, economic losses, or any monetary quantification of disease burden. Ranking criteria are outlined below.

LOCATION

West Africa: Senegal, Mali, Ghana, Burkina Faso, Ivory Coast, Togo, Benin, Nigeria

East Africa: Tanzania, Kenya, Uganda, Ethiopia, South Sudan, Malawi, Mozambique, Zambia, *(Zimbabwe if relevant)*

LIVESTOCK

Dairy/ beef: Cattle

Poultry: Chickens

Small-ruminants: Goats, sheep

PRODUCTION SYSTEM

Smallholders, agro-pastoralists, pastoralists, small commercial.

TIME FRAME: Based on publication date of citation.

2002 - present

TOPIC INCLUSION CRITERIA

- Relevant location and livestock species
- Primary or secondary data, reviews only if relevant and well-written
- Written in English or French
- Addresses animal health needs of smallholder farmers

Excluded following topics:

- Breeding strategies
- Nutrition/ feeding strategies
- Anti-microbial resistance
- Production constraints (diseases not specified)
- Characterizations of production systems (diseases not specified)
- Ethno-veterinary practices (diseases not specified)
- Reports of zoonotic diseases focusing only on human health impact
- Genotyping or other basic research on animal health diseases / vectors if smallholder impact not specified

- Vaccine trials
- Human serosurveys of zoonoses (unless accompanied by animal serosurveys)

Included following topics:

- Participatory epidemiology
- Ethno-veterinary practices (diseases specified)
- Aflatoxins

RANKING CRITERIA

	Criteria for green rank:<br>• Excellent study design and writing<br>• Addresses <u>incidence/ prevalence</u> **AND** <u>impact</u> of animal health concerns **OR** addresses impact thoroughly from a smallholder perspective<br>• Smallholder perspectives well-represented<br>• May cover multiple species or multiple geographies<br>• Replicable study design
	Criteria for yellow rank:<br>• Addresses <u>incidence/ prevalence</u> **OR** <u>impact</u> of animal health concerns<br>• Relevant to smallholders but smallholder perspectives may not be directly assessed<br>• Smallholder perspectives directly assessed but limited sample size or geography
	Criteria for red rank:<br>• Partially addresses frequency or impact of animal health concerns<br>• Smallholder perspectives directly assessed<br>• Small sample size and limited geography<br>• Study design not clear enough to be replicable
