# Supplementary material for: Prioritizing smallholder animal health needs in East Africa, West Africa, and South Asia using three approaches: Literature review, expert workshops, and practitioner surveys
Source: Prev Vet Med. 2021 Apr;189:105279. doi: 10.1016/j.prevetmed.2021.105279 (PMC8024747; doi:10.1016/j.prevetmed.2021.105279)
Supplement: Supplementary file 9 [file mmc9.pdf]

Smallholder Animal Health Needs Assessment
South Asia Bovids

GALVmed

August 19, 2019

Assembled by Zoë Campbell, Peetambar Kushwaha, and Paul Coleman

Meta-analysis summary

The literature review yielded 61 articles, 37 of which focused on impact, incidence, and prevalence, and are summarized below. The table below shows the number of articles in which each animal health concern appears in the literature about cattle, buffalo, mithun (*Bos frontalis)*, and yak (bovids) in South Asia. Many articles mention multiple concerns. The summarized articles are in the bibliography within this document. The full list of articles considered in the South Asia meta-analysis is available as a separate document.

The most mentioned concerns in the meta-analysis are ectoparasites, foot and mouth disease (FMD), brucellosis, theileriosis, diarrhoea, and reproductive disorders (mentioned in 7+ articles). In the impact articles, FMD is the top concern, mentioned in ten articles, followed by ectoparasites, brucellosis, diarrhoea, reproductive disorders, and abortion. Theileriosis is mentioned in only one of the impact articles. Only two articles were assigned a green ranking for focusing on both incidence/ prevalence and disease impact. These two articles addressed brucellosis and trypanosomiasis, respectively.

Animal health concern	Frequency of mention (meta-analysis)	Frequency of mention (impact articles)	Main topic of green rank article
Ectoparasites	12	8	0
FMD	12	10	0
Brucellosis	11	8	1
Mastitis	10	6	0
Theileriosis	8	1	0
Diarrhoea	7	7	0
Reproductive disorders	7	6	0
Abortion	6	6	0
Bloat	6	6	0
Constipation	6	5	0
Haemorrhagic septicaemia	6	5	0

Helminth	6	3	0
Pneumonia	6	3	0
Respiratory disease	5	4	0
Retention foetal membrane	5	5	0
Tick-borne disease	5	1	0
Trypanosomiasis	5	2	1
Anthrax	4	4	0
Black quarter	4	4	0
Digestive disorders	4	4	0
Endoparasites	4	4	0
Lameness	4	3	0
Dystocia	3	3	0
Rabies	3	3	0
Ringworm	3	3	0
Acidosis	2	2	0
Arthritis	2	2	0
Babesiosis	2	1	0
Fascioliasis	2	2	0
Johne's disease	2	2	0
Skin problems	2	2	0
Tuberculosis	2	1	0
Warble fly infestation	2	1	0
Acaricide resistance	1	1	0
Actinobacillosis	1	1	0
Anoestrus	1	1	0

Anaplasmosis	1	1	0
Bovine viral diarrhoea virus	1	1	0
Cryptosporidiosis	1	1	0
Foot conditions	1	1	0
Laminitis	1	1	0
Linguatula serrata	1	1	0
Mange	1	1	0
Paramphistomiasis	1	0	0
Pasteurellosis	1	0	0
Vaginal and uterine prolapses	1	1	0

Table of articles

Abbreviations:<br>FMD – Foot and mouth disease<br>HS - Haemorrhagic septicaemia<br>BQ – Black quarter<br>TB – Tuberculosis

Note: Clicking on the article number links to an expanded summary of the article below. The "web" link connects to the abstract of the article online or to full text for open access articles.

#	Citation	Country	Study design	Pathogen/ disease	Findings
1<br>2<br>web	(Chhetri, Perez, & Thurmond, 2010)	Nepal	Statistical model to identify factors associated with FMD outbreak in different clusters of Nepal, Questionnaire for district veterinary	FMD	**Incidence/ prevalence:** Of the 75 districts, 29 (39%) reported at least one VDC reported at least one FMD case in 2004. Altogether, 214 (5.5%) of the 3913 VDCs reported one or more outbreaks of FMD.<br>**Impact:** FMD losses in Nepal have been estimated to be about 5.36 million USD per year (Gongal and Karki 2000).

			officers to report FMD cases by village development committees (VDCs) in 2014		
2<br>2<br>web	(Sharma et al., 2017)	India	Longitudinal, one-year serosurvey, 115,963 serum samples from cattle in 295 districts of the 18 states	FMD	**Incidence/ prevalence:** The mean antibody titre against the serotypes O, A and Asia1 was estimated as log10 1.93 (95% CI 1.92–1.93), 2.02 (2.02–2.02) and 2.02 (2.02–2.02), respectively, in the states covered under the control programme. However, in other states herd immunity was significantly low [mean titre log10 1.68 (95% CI 1.67–1.69), 1.77 (1.76–1.78) and 1.85 (1.84–1.86) against the three serotypes].<br>**Impact:** Inverse relationship between the herd immunity and FMD incidences was observed in the states following different vaccination practices.
3<br>3<br>web	(Mishra, Sahu, Mishra, & Behera, 2015)	India	Literature review of herbal treatments commonly used by smallholders in India for treatment of	FMD<br>Haemorrhagic septicaemia (HS), Black quarter (BQ), Endoparasites, Helminths, Anthrax, Actinobacillosis,	**Perceptions:** 14 plant species are described for treatment of FMD, 9 plant species each for treatment of HS and BQ, 5 plant species for round worm infestation, 4 plant species for anthrax, 3 plant species each for actinobacillosis and ringworm infection, and 1 plant species for fluke infestation.

			cattle and small ruminants	Ringworm	
4<br>3<br>web	(Borah et al., 2018)	India	Viral isolation, Outbreak investigation, 15 tissue samples from FMD-suspected mithun	FMD	Despite the geographical inaccessibility, infection by FMD virus has emerged as the single most devastating disease among mithun after the eradication of rinderpest from north-east India. The results indicate the presence of FMDV serotype "O." The sequencing and molecular phylogenies have revealed close relationships in the lineage of type "O" isolates from Bangladesh.
5<br>1<br>web	(B. B. B. Singh, Dhand, & Gill, 2015)	India	Economic model using official records and epidemiological surveys, Multiple production systems, Cattle, buffalo, small ruminants	Brucellosis	**Impact:** The analysis revealed that brucellosis in livestock is responsible for a median loss of US $ 3.4 billion (5th–95th percentile 2.8–4.2 billion). The disease in cattle and buffalo accounted for 95.6% of the total losses occurring due to brucellosis in livestock populations. The disease is responsible for a loss of US $6.80 per cattle and US $18.20 per buffalo.
6<br>3<br>web	(Kant et al., 2018)	India	Cross-sectional, Questionnaire, Purposive sample of 1,200 cattle sheds in Delhi which	Brucellosis, Abortions, Retention foetal membrane	**Perceptions:** Amongst all the 11 districts of Delhi, there was 0% awareness about brucellosis or the S19 vaccine, available at minimal cost from Indian Veterinary Research Institute.

			included data of 5,550 buffaloes		
7<br>3<br>web	(Rajkumar et al., 2016)	India	Cross-sectional questionnaire with 250 randomly selected livestock farmers from 8 villages	Brucellosis<br>TB<br>Anthrax<br>Avian influenza	**Perceptions:** Only 4.8%, 3.6%, 6.8%, and 22.4% of respondents knew about the zoonotic potential of diseases such as brucellosis, tuberculosis (TB), anthrax, and avian flu, respectively. Only 18% of the respondents were aware about zoonotic diseases from cattle. Only 0.8 % of the respondents knew about vaccination against brucellosis. This may be due to lack of awareness of brucellosis; however, many respondents reported third trimester abortion in their cattle.
8<br>2<br>web	(Priyadarshini et al., 2013)	India	Cross-sectional, Serosurvey, 258 cattle, 87 milk samples, 346 serum samples, Dairy	Brucellosis	**Incidence/ prevalence:** Cattle seropositivity by I-ELISA, RBPT and STAT were found to be 8.14%, 4.26% and 2.32% respectively. Milk sample analysis showed 3.44% positivity in MRT.
9<br>2<br>web	(Islam, Khatun, Werre, Sriranganathan, & Boyle, 2013)	Bangladesh	Meta-analyses of published papers on brucellosis in cattle, buffalo, cattle, buffalo, sheep and goats	Brucellosis	**Incidence/Prevalence:** Seroprevalences were estimated as 3.7% for cattle and 4% for buffalo.
10	(Jackson, Nydam, & Altier, 2014)	Nepal	Cross-sectional, Serosurvey,	Brucellosis	**Incidence/ prevalence:** Estimated prevalence was 0.22 (95% CI: 0.17; 0.28).

3<br><br>web			297 yak from 61 herds, Pastoralists		
11<br><br>2	(Satisha, Tiwari, & Roy, 2018)	India	Cross-sectional, Questionnaire, 135 respondents from 3 districts representing small, medium, and large commercial dairies	Ectoparasites<br>Mastitis<br>FMD<br>HS<br>Black quarter<br>Milk fever<br>Anthrax	**Incidence/ prevalence:** As reported by farmers for the previous two years, mastitis had the highest incidence rate (47%) followed by FMD (32%), HS (10%), and black quarter (4%). Only 5% and 2% of animals were affected with milk fever and anthrax. 40% of dairy farms had animals affected with ectoparasites at the time of the survey.
12<br><br>3<br><br>web	(Sathiyabarathi et al., 2016)	India	Report of diagnostic technology	Mastitis	**Impact:** INR 60 Billion (848 million USD) annual loss due to mastitis in India. 100-500 kg milk loss due to mastitis per lactation.<br>**Perception:** Infrared thermography can be used to detect sub-clinical mastitis to reduce the loss due to mastitis in dairy cattle.
13<br><br>2<br><br>web	(Yogesh Chandrakant Bangar, Singh, Dohare, & Verma, 2015)	India	Systematic review of prevalence of subclinical mastitis in dairy cows for the period 1995–2014, 6,344 dairy cattle across 25 studies	Sub-clinical mastitis	**Incidence/ prevalence:** 47% cows were found positive for sub-clinical mastitis. Quarter-wise prevalence was 23%.<br>**Impact:** Sub-clinical mastitis could be a contributing factor behind low milk production from cows in India.

14<br>2	(Meena, Ram, Sahoo, & Rasool, 2008)	India	Cross-sectional questionnaire, 300 smallholders dairy farmers, Knowledge, attitudes, practices about cattle and small ruminant production	Diarrhoea,<br>Respiratory disease,<br>Haemorrhagic septicaemia,<br>FMD,<br>Sheep and goat pox,<br>PPR,<br>Reproductive disorders	**Impact:** The principal causes of livestock mortality as reported by farmers were infectious diseases like diarrhoea, pneumonia, haemorrhagic septicaemia (HS), foot and mouth disease, pox, PPR (30% of mortalities). Reproductive disorders such as difficulty in parturition, abortions, and retained placenta made up 25% of mortalities.<br>**Perceptions:** Local remedies used for common diseases including diarrhoea, common cold, pneumonia, and bloat. Only 27% of farmers knew about vaccination against diseases like HS, black quarter, FMD, PPR, and pox.
15<br>3<br>web	(Rajkhowa, Rajkhowa, & Hazarika, 2006)	India	Cross-sectional, Faecal samples from 154 mithun in semi-intensive system of management	Cryptosporidiosis<br>Diarrhoea	Overall prevalence of *Cryptosporidium parvum* in mithun was 56% (95% CI = 48–64). Highest (prevalence rate = 81, 95% CI = 67–95) prevalence was found in mithun of 1–6 months of age and lowest (prevalence rate = 42, 95% CI = 31–53) in mithun above 2 years of age. The prevalence was found to be higher (94%) in diarrhoeic animals in comparison to the non-diarrhoeic group (51%).

16<br>3<br>web	(Prasad, Ramachandran, & Raju, 2004)	India	Mortality records of 1,115 dairy cows measured twice cross-sectionally in a research herd after a 9 year interval	Digestive disorders, Respiratory disorder	**Impact:** The overall mortality was 14.17%. Digestive problems followed by respiratory disorders together accounted for 70-80% of total deaths.
17<br>2<br>web	(Kulangara et al., 2015)	India	Cross-sectional, Serosurvey, 385 dairy cattle, Smallholders	Bovine viral diarrhoea	**Incidence/ prevalence:** Prevalence was 24.7 % among the total population, but was higher (52%) when 85 animals having infertility problems alone were considered.
18<br>3<br>web	(Jagadeeswary, Reddy, & Satyanarayan, 2014)	India	Cross-sectional, Qualitative methods including semi-structured interviews, 20 practitioners of ethnoveterinary medicine	Digestive disorders, Diarrhoea, Bloat, Endoparasites, Respiratory disease, Pneumonia, FMD, Retention foetal membrane, Dystocia, Mastitis, Ectoparasites, Mange	**Perceptions:** Ethnoveterinary practices covered ailments covered including extra teeth, anorexia, stomatitis, bloat, constipation, diarrhoea, endoparasites, dysentery, cold and cough, ephemeral fever, drenching, pneumonia, FMD, repeat breeding, retained placenta, dystocia, metritis, mastitis, corneal opacity, ticks and lice, mange, fracture, leg pain, tetanus, horn avulsion, snake bite, poisonous plant consumption, yoke gall, abscess, tail gangrene, and allergic lesions.
19	(Panda & Mishra, 2016)	India	Cross-sectional, Qualitative methods	Multiple	**Perceptions:** The primary ailments of livestock are wounds, diarrhoea, injury, fever, gastrointestinal problems and

3<br><br>web			including participant observation and interview, Ethnoveterinary		gynaecological problems, which are commonly treated with medicinal plants.
20<br><br>3<br><br>web	(Bhatt, B. Pandya, Patel, Patel, & Modi, 2019)	India	Cross-sectional, Ethnoveterinary, Semi-structured questionnaire with 82 male livestock owners	Gastrointestinal problems, Multiple	**Perceptions:** A total of 13 medicinal plants were cited 47 times for gastrointestinal problems, which were the highest. Other animal health concerns treated with ethnoveterinary medicine included retention foetal membrane, enteritis, cough and respiratory disease, wounds, skin problems, ringworm, foot conditions, vaginal and uterine prolapses, and bloat.
21<br><br>2<br><br>web	(Khan, Manoj, & Pramod, 2008)	India	Longitudinal 3-year study of 576 cross-bred dairy cows under semi-intensive management	Reproductive disorder, Retention foetal membrane, Abortion, Anoestrus	**Incidence/ Prevalence:** Repeat breeding (RB), anoestrus, retention of foetal membrane, and abortion were found to be the major clinical reproductive problems. Out of the total animal affected with reproductive disorders, the incidence of anoestrus, RB, retention of foetal membrane, and abortion was found to be 31.79% (n=62), 24.61% (n=48), 14.35% (n=28), and 11.25% (n=22), respectively. In addition, dystocia (5.12%), prolapse (1.53%), endometritis (4.61%), and pyometra (6.66%) were minor clinical reproductive problems.

22<br>3<br>web	(Y.C. Bangar, Dohare, Kolekar, Avhad, & Khan, 2015)	India	Longitudinal one-year study of disease seasonality amongst 1,538 dairy and beef cattle, Multiple production systems	Reproductive disorders Gastrointestinal disease	**Incidence/ prevalence:** Overall incidence of all diseases was high in winter (41.23%) followed by rainy season (39.18%) than that of in summer (19.59%). Overall incidences of reproductive diseases (17.84%) and digestive diseases (11.11%) were major problems in whole study period. A log-linear modelling showed that the odds of incidences of reproductive diseases were significantly higher in summer as well as in rainy season than in winter season. Winter season was dominated by occurrences of digestive diseases as compared to any other diseases.
23<br>3<br>web	(Thakur, Thakur, & Dogra, 2018)	India	Participatory rural appraisal technique (qualitative methods including focus groups and key informant interviews) with rural dairy farmers keeping buffalo	Reproductive disorders, Retention foetal membrane, Dystocia	**Perceptions:** Documents feeding practices used by rural farmers to prevent or treat common reproductive disorders including repeat breeding, early embryonic mortality, inability to carry the pregnancy full term, prolapse, dystocia, and retained placenta.
24	(Malik & Verma, 2018)	India	Cross-sectional study in Punjab	Haemorrhagic septicaemia	**Incidence/ prevalence:** The overall incidence and case fatality rate of HS among

2<br>web			with 720 dairy farmers with small, medium, and large herd sizes,<br>Cattle and buffalo		dairy animals was 5.45 and 45.15% respectively. Incidence risk of HS on the species basis varied significantly and was observed highest in buffaloes (6.27%), followed by crossbred (4.67%) and indigenous cattle (3.91%). Within different herd sizes, the incidence risk of HS varied significantly and was highest in small herd size (10.52%) followed by medium (5.44%) and large herd size (3.01%). Incidence risk varied significantly between the different agro-climatic zones of Punjab within small and large sized herds but not in medium sized herds. CFR among the different herds was highest in small herds (49.28%) followed by medium herds (44.32%) and large herds (39.15%).
25<br>3<br>web	(Wright & Thrusfield, 2016)	India	Cross-sectional, Participatory epidemiology with 4 pastoralist groups of 35-55 participants per group,<br>Main livestock was buffalo for dairy.	Trypanosomiasis<br>Helminths<br>Endoparasites<br>FMD<br>Diarrhoea	**Perceptions:** Animal disease of most concern to pastoralists included trypanosomiasis, worms, foot and mouth disease, and diarrhoea.

26<br>2<br>web	(Kelly et al., 2018)	Nepal	Cross-sectional survey of 90 smallholder farmers, focus on buffalo and cattle and zoonotic diseases	Fasciolosis<br>Ectoparasites<br>Ringworm<br>FMD<br>Mastitis<br>Diarrhoea<br>Respiratory disease<br>Lameness<br>Babesiosis<br>Anaplasmosis	**Perceptions:** Across the study districts, several farmers reported health problems affecting their buffaloes (58%) and cattle (36%) over the previous year. The most commonly reported diseases in cattle and buffaloes by the farmers were fasciolosis (27%), tick infestation (12%), ringworm (10%), foot-and- mouth disease (FMD) (10%), and mastitis (7%). When unable to name a specific disease, farmers reported a wide range of clinical manifestations, including diarrhoea, respiratory disease, weight loss, skin lesions, haemorrhage, and lameness. Economically important tick-borne diseases (babesiosis and anaplasmosis) have recently been identified in cattle in western Nepal (Bohara 2014).
27<br>1<br>web	(Kumar et al., 2017)	India	Risk and retrospective analyses to estimate economic loss	Trypanosomosis	**Incidence/ prevalence:** Cites Kundu et al., 2013, reporting the prevalence of trypanosomosis in cattle as 10.66% (95% CI: 8.19, 13.13) in three different states namely, Kerala, Odisha, and Uttar Pradesh covering three different agro-climatic zones.<br>For buffalo, authors calculated the aggregate of different prevalence values published in different journals. Using recorded data from the published reports covering maximum regions of India and in

					all, 320 buffaloes were found positive out of 7,703 screened, showing a prevalence of 4.15% (95% CI: 3.7, 4.6) in India.<br>**Impact:** A total annual loss (direct and indirect) caused by animal trypanosomosis was estimated to US $ 671.1 million (US $ 344–US $ 1209 million at 95% confidence interval), at present valuation. The mortality losses were estimated to 16% of the total loss. Among morbidity losses, the reduction in milk yield and reproductive losses components were 36% and 26% of total loss, respectively. Other parameters like reduction in growth (10%), reduction in draught power (8%), and additional opportunity cost (3%) also yielded considerable loss.
28<br>3<br>web	(Mondal, Sarma, & Saravanan, 2013)	India	Literature review	Tick-borne diseases, Acaricide resistance	**Impact:** A recent estimate calculated the costs of control of tick-borne diseases affecting Indian livestock as 498.7 million USD per annum (Minjauw and McLeod, 2003). As per a leather industry report, the leather sector of India is suffering from a huge shortfall of 3 billion pieces of hide and skin per year and causing 20–30% depreciation in normal value in the market. It has been estimated that India produces only 9.8%, 63.3%, 9.2%, and 6.0% of world

					cattle, buffalo, goat, and sheep hides, respectively, although the country possesses the highest livestock population.
29<br>3<br>web	(Brookes et al., 2019)	India	Cross-sectional, Retrospective outbreak investigation, Knowledge, attitudes, practices survey about rabies prevention	Rabies	**Impact:** Rabies outbreak in bovines reported in August 2015 in a Punjab village. The number of affected bovines was unusually high; 15 cattle and buffalo died on 13 smallholder farms (attack rate 4%).
30<br>3<br>web	(Sangma & Manohara, 2018)	India	Cross-sectional, Qualitative methods including group discussion and interview, Ethnoveterinary	Multiple	**Perceptions:** Animal health concerns with the highest consensus of appropriate ethnoveterinary treatment were arthritis, rabies, reproductive diseases, retained placenta, and swollen body parts. Other health concerns commonly treated include diarrhoea, wounds, and ectoparasites.
31<br>2<br>web	(Whittington et al., 2019)	48 countries including India	Cross-sectional survey of collaborators in multiple countries using regional-level data in respective	Johne's disease (Paratuberculosis)	**Incidence/ prevalence:** Johne's disease was not considered notifiable for India, meaning it was not reported for any region in India from 2012-2018.

			countries from 2012–2018, Cattle		
32<br>2<br>web	(S. V. Singh et al., 2008)	India	Cross-sectional, Serosurvey, Serum samples from 726 cattle and buffalo from 33 randomly selected villages	Johne's Disease caused by *Mycobacterium avium subsp. paratuberculosis*	**Incidence/ prevalence:** Seroprevalence was 23.3% (12.1% in young and 24.4% in adults) and 26.9% (27.2% in young and 26.8% in adults) in buffaloes and cattle, respectively. **Perceptions:** Major livestock problems reported include reproductive problems, FMD, HS, internal and external parasites, flukes, and mastitis.
33<br>3<br>web	(Yadav, Panadero, Katoch, Godara, & Cabanelas, 2017)	Mediterranean and Indian subcontinent	Review, Multiple production systems	Myiasis (Warble fly infestation)	**Incidence/ prevalence:** Bovine infestation is mainly reported in the northern states of India with prevalence ranging from 9.73% (Yadav et al., 2013) to 72.46% (Kumar et al., 1990).
34<br>2<br>web	(Sudan, Jaiswal, & Shanker, 2014)	India	Cross-sectional, Abattoir study, Prevalence, 480 buffalo	*Linguatula serrata*	**Incidence/ prevalence:** Results revealed 88 (18.3%) buffaloes and 288 (20.0%) mesenteric lymph nodes having parasite's nymphs.
35<br>2<br>web	(Murugeswari, Valli, Karunakaran, Leela, & Pandian, 2018)	India	Cross-sectional, Questionnaire and prevalence survey, 200 randomly selected dairy farmers, Smallholders	Acidosis<br>Laminitis	**Incidence/Prevalence:** 71.5% of farmers feed cooked rice to their cattle. Incidence of acidosis progressively increased from 29% in cows fed with 0.5 kg of cooked rice to 69% in cows fed with more than 2.5 kg of cooked rice. Among cattle fed a cooked rice-based diet, the incidence of laminitis increased progressively ($p<0.05$) from 9.2% to 37.9%

					with the increase in the quantity of cooked rice in the diet.
36<br><br>2	(Kathiravan, Thirunavukkarasu, & Selvakumar, 2005)	India	Cross-sectional, 320 respondents seeking services from public veterinary centres, Multiple production systems, Cattle, buffalo, small ruminants	Cost of animal health services	**Impact:** The mean cost of treatment of a chronic medical case in cattle at a public veterinary centre was INR 20.83, in which the labour cost alone accounted to INR 17.35, with the remaining amount for the drugs purchased outside. However, the mean costs of treating a chronic medical condition in buffalo and small ruminant at public veterinary centres were only INR 13.34 and INR 10.80, respectively. Although no charges were made for animal health care services rendered at public veterinary centres, the charges in terms of imputed labour cost for bringing the animal to the centre was incurred. Service fee accounted for more than 60 per cent of cost of treatment for home service by a veterinarian or a para-veterinarian.
37<br><br>3<br><br>web	(Kathiravan, Thirunavukkarasu, & Michaelraj, 2007)	India	Willingness to pay survey, 320 small ruminant farmers from four districts	Annual health care services	At a service centre, the estimated willingness to pay (WTP) was 56 INR for sheep and 61 INR for goats. At the farmer's doorstep, the estimated WTP was 87 IND for sheep and 95 INR for goats.

Article summaries

Chhetri, B. K., Perez, A. M., & Thurmond, M. C. (2010). Factors associated with spatial clustering of foot-and-mouth disease in Nepal. *Tropical Animal Health and Production*, *42*(7). https://doi.org/10.1007/s11250-010-9573-7

Statistical model to identify factors associated with foot and mouth disease outbreaks in Nepal. A questionnaire was administered to district veterinary officers requesting reports of FMD cases by village development committees (VDCs) in 2014.

- Of the 75 districts, 29 (39%) reported at least one VDC reported at least one FMD case in 2004. Altogether, 214 (5.5%) of the 3913 VDCs reported one or more outbreaks of FMD.
- The results of this study indicate that the FMD risk in Nepal in 2004 was spatially clustered in specific areas of the country, and that the elevated risk in those areas was largely associated with the size of human and buffalo populations and with the number of technicians. Further studies should be directed to clarify the reasons for these associations.
- FMD losses in Nepal have been estimated to be about USD 5.36 million per year (Gongal and Karki 2000).

Sharma, G. K., Mahajan, S., Matura, R., Biswal, J. K., Ranjan, R., Subramaniam, S., Pattnaik, B. (2017). Herd Immunity Against Foot-and-Mouth Disease Under Different Vaccination Practices in India. *Transboundary and Emerging Diseases*, *64*(4), 1133–1147. https://doi.org/10.1111/tbed.12478

Longitudinal, one-year serosurvey of foot and mouth disease using 115,963 serum samples from cattle in 295 districts of the 18 states of India with the goal of comparing herd immunity under different vaccination practices.

- The mean antibody titre against the serotypes O, A and Asia1 was estimated as log10 1.93 (95% CI 1.92–1.93), 2.02 (2.02–2.02) and 2.02 (2.02–2.02), respectively, in the states covered under the control programme. However, in other states herd immunity was significantly low [mean titre log10 1.68 (95% CI 1.67–1.69), 1.77 (1.76–1.78) and 1.85 (1.84–1.86) against the three serotypes].
- Inverse relationship between the herd immunity and FMD incidences was observed the states following different vaccination practices.
- The study helped in demarcation of FMD risk zones in the country with low herd immunity.

Mishra, D., Sahu, R., Mishra, N., & Behera, A. (2015). Herbal treatment for common diseases in ruminants: an overview. *Journal of Livestock Science*, *6*, 36–43.

Literature review of herbal treatments commonly used by smallholders in India for treatment of cattle and small ruminants.

- Fourteen plant species are described for treatment of FMD, 9 plant species each for treatment of HS, BQ, 5 plant species for round worm infestation, 4 plant species for anthrax, 3 plant species each for actinobacillosis and ringworm infection, and 1 plant species for fluke infestation.
- The description of herbal treatments gives an indication of livestock diseases that affect smallholder farmers in India.

Borah, B., Deka, P., Sharma, K., Baro, S., Hazarika, A. K., Das, C., Ltu, K. (2018). Isolation, identification and retrospective study of foot-and-mouth disease virus from affected Mithun (Bos frontalis) in north-eastern India. *Transboundary and Emerging Diseases*, *65*(1), e63–e69. https://doi.org/10.1111/tbed.12678

Viral isolation of the foot and mouth disease virus in mithun in north-eastern India with samples collected from FMD-suspected mithun following outbreaks in 2015-2016.

- Despite the geographical inaccessibility, infection by FMD virus has emerged as the single most devastating disease among mithun after the eradication of rinderpest from north-east India.
- The results indicate the presence of FMDV serotype "O." The sequencing and molecular phylogenies have revealed close relationships in the lineage of type "O" isolates from Bangladesh.

Singh, B. B. B., Dhand, N. K. K., & Gill, J. P. S. P. S. (2015). Economic losses occurring due to brucellosis in Indian livestock populations. *Preventive Veterinary Medicine*, *119*(3–4), 211–215. https://doi.org/10.1016/j.prevetmed.2015.03.013

Economic model used to estimate the losses due to brucellosis in Indian livestock including cattle, buffalo, sheep, and goats. Data sources included prevalence data from epidemiological surveys conducted in India and data for livestock populations from official records. Probability distributions were used for many of the input parameters to account for uncertainty and variability.

- The analysis revealed that brucellosis in livestock is responsible for a median loss of 3.4 billion USD (5th–95th percentile 2.8–4.2 billion).
- The disease in cattle and buffalo accounted for 95.6% of the total losses occurring due to brucellosis in livestock populations.
- The disease is responsible for a loss of $6.80 USD per head of cattle and $18.20 USD per buffalo.

Kant, N., Kulshreshtha, P., Singh, R., Mal, A., Dwivedi, A., Ahuja, R., Bhatnagar, R. (2018). A study to identify the practices of the buffalo keepers which inadvertently lead to the spread of brucellosis in Delhi. *BMC Veterinary Research*, *14*(1), 329. https://doi.org/10.1186/s12917-018-1670-2

Cross-sectional questionnaire administered to buffalo keepers in Delhi to learn about knowledge, attitudes, and practices around brucellosis. Purposive sample of 1,200 cattle sheds in Delhi which included data of 5,550 buffaloes.

- Amongst all the 11 districts of Delhi, there was 0% awareness about brucellosis and also about the S19 vaccine, available at minimal cost from Indian Veterinary Research Institute.
- While about 20 to 27% of respondents reported a history of abortions and retained placenta, disposed of the placenta with bare hands, and applied raw milk on cracked lips.
- It was surprising to note that majority of them never reared small ruminants like sheep and goat with buffaloes or *Bos* species as they were aware of the rapid spread of disease from small to big ruminants.

Rajkumar, K., Bhattacharya, A., David, S., Balaji, S. H., Hariharan, R., Jayakumar, M., & Balaji, N. (2016). Socio-demographic study on extent of knowledge, awareness, attitude, and risks of zoonotic diseases among livestock owners in Puducherry region. *Veterinary World*, *9*(9), 1018–1024.

Cross-sectional questionnaire with 250 randomly selected livestock farmers from 8 villages in Puducherry region, India.

- Only 4.8%, 3.6%, 6.8%, and 22.4% of respondents knew about the zoonotic potential of diseases such as brucellosis, tuberculosis (TB), anthrax, and avian flu, respectively.
- Only 18% of the respondents were aware about zoonotic diseases from cattle.
- Only 0.8 % of the respondents knew about vaccination against brucellosis. This may be due to lack of awareness of brucellosis; however, many respondents reported third trimester abortion in their cattle.

Priyadarshini, A., Sarangi, L., Palai, T., Panda, H., Mishra, R., & Behera, P. (2013). Brucellosis in Cattle and Occupationally Exposed Human Beings: A Serosurvey in Odisha, India. *Journal of Pure and Applied Microbiology*, *7*(4), 3255–3260.

Cross-sectional serosurvey of humans and 258 cattle in Odisha, India to estimate the prevalence of brucellosis. Seropositivity was assessed by Indirect ELISA, Rose Bengal Plate Test (RBPT), and Standard Tube Agglutination Test (STAT) and 87 milk samples were analysed using the Milk Ring Test (MRT). The production system was not specified

- Cattle seropositivity by I-ELISA, RBPT and STAT were found to be 8.14%, 4.26% and 2.32% respectively.
- Milk sample analysis showed 3.44% positivity in MRT.

Islam, M. A., Khatun, M. M., Werre, S. R., Sriranganathan, N., & Boyle, S. M. (2013). A review of Brucella seroprevalence among humans and animals in Bangladesh with special emphasis on epidemiology, risk factors and control opportunities. *Veterinary Microbiology*, *166*(3–4), 317–326. https://doi.org/10.1016/j.vetmic.2013.06.014

Meta-analyses of published papers on brucellosis in cattle, buffalo, sheep and goats in Bangladesh.

- Seroprevalences were estimated as 3.7% for cattle and 4% for buffalo.
- Different tests, of varying sensitivity and specificity, have been used in different studies. As a result, the meta-analysis just gives an indication of prevalence rates.

Jackson, D. S., Nydam, D. V, & Altier, C. (2014). Prevalence and risk factors for brucellosis in domestic yak Bos grunniens and their herders in a transhumant pastoralist system of Dolpo, Nepal. *Preventive Veterinary Medicine*, *113*(1), 47–58.

Serosurvey of 297 yak in 61 herds in Dolpo, Nepal and questionnaire administered to 61 pastoralists to assess the seroprevalence of brucellosis in yak and risk factors.

- Estimated prevalence of brucellosis in yak was 0.22 (95% CI: 0.17; 0.28).
- Yak in herds reporting abortion occurrence within the previous year were 2.3 times more likely to be seropositive than those in herds not reporting abortion (95% CI: 1.2; 4.2, p = 0.01). For every 10 animal increase in herd number, individual animal seropositivity risk increased by 30% (95% CI: 10%; 50%, p = 0.001). Male yak were 0.7 times as likely to be seropositive as female yak (95% CI: 0.5; 0.9, p = 0.01).

Satisha, M. C., Tiwari, R., & Roy, R. (2018). Performance of dairy animals in commercial dairy farms in Karnataka. *Indian Journal of Dairy Science*, *71*(6), 620–624.

A cross-sectional questionnaire was administered to 135 respondents from 3 districts representing small, medium, and large commercial dairies to learn about production performance and disease burden of dairy cattle in Karnataka, India. Karnataka was purposively selected as a leading area for milk production and commercial dairies. There is no known web access to the article.

- As reported by farmers for the previous two years, mastitis had the highest incidence rate (47%) followed by FMD (32%), HS (10%), and black quarter (4%). Only 5% and 2% of animals were affected with milk fever and anthrax.
- 40% of dairy farms had animals affected with ectoparasites at the time of the survey.

Sathiyabarathi, M., Jeyakumar, S., Manimaran, A., Jayaprakash, G., Pushpadass, H. A., Sivaram, M., Kumar, R. D. (2016). Infrared thermography: a potential noninvasive tool to monitor udder health status in dairy cows. *Veterinary World*, *9*(10), 1075–1081. https://doi.org/10.14202/vetworld.2016.1075-1081

This article reports on the potential for infrared thermography as a tool to monitory udder health status in dairy cows.

- INR 60 Billion (848 million USD) annual loss due to mastitis in India. 100-500 kg milk loss due to mastitis per lactation.
- Infrared thermography can be used to detect sub-clinical mastitis to reduce the loss due to mastitis in dairy cattle.

Bangar, Y. C., Singh, B., Dohare, A. K., & Verma, M. R. (2015). A systematic review and meta-analysis of prevalence of subclinical mastitis in dairy cows in India. *Tropical Animal Health and Production*, *47*(2), 291–297. https://doi.org/10.1007/s11250-014-0718-y

Systematic review of prevalence of subclinical mastitis in dairy cows in India for the period 1995–2014. The sample size was 6,344 dairy cattle across 25 studies.

- 47% cows were found positive for sub-clinical mastitis. Quarter-wise prevalence was 23%.
- Sub-clinical mastitis could be a contributing factor behind low milk production from cows in India.

Meena, H., Ram, H., Sahoo, A., & Rasool, T. (2008). Livestock husbandry scenario at high altitude Kumaon Himalaya. *Indian Journal of Animal Sciences*, *78*(8), 882–886.

Cross-sectional questionnaire administered to 300 smallholders dairy farmers focusing on knowledge, attitudes, and practices for livestock husbandry of cattle and small ruminants. Some lack of clarity about when primary versus secondary data are used. There is no known web access to the article.

- The principal causes of livestock mortality as reported by farmers were infectious diseases like diarrhoea, pneumonia, haemorrhagic septicaemia (HS), foot and mouth disease, pox, PPR (30% of mortalities).
- Reproductive disorders such as difficulty in parturition, abortions, and retained placenta made up 25% of mortalities.
- Local remedies used for common diseases including diarrhoea, common cold, pneumonia, and bloat.
- Only 27% of farmers knew about vaccination against diseases like HS, black quarter, FMD, PPR, and pox.

Rajkhowa, S., Rajkhowa, C., & Hazarika, G. C. (2006). Prevalence of Cryptosporidium parvum in mithuns (Bos frontalis) from India. *Veterinary Parasitology*, *142*(1–2), 146–149. https://doi.org/10.1016/j.vetpar.2006.06.026

A cross-sectional survey of 154 mithun under semi-intensive management system in India was undertaken to measure the prevalence and risk factors for *Cryptosporidium parvum.*

- Overall prevalence of *Cryptosporidium parvum* in mithun was 56% (95% CI = 48–64).
- Highest (prevalence rate = 81, 95% CI = 67–95) prevalence was found in mithun of 1–6 months of age and lowest (prevalence rate = 42, 95% CI = 31–53) in mithun above 2 years of age.
- The prevalence was found to be higher (94%) in diarrhoeic animals in comparison to the non-diarrhoeic group (51%).

Prasad, S., Ramachandran, N., & Raju, S. (2004). Mortality patterns in dairy animals under organized herd management conditions at Karnal India. *Tropical Animal Health and Production*, *36*(7), 645–654. https://doi.org/10.1023/B:TROP.0000042855.58026.bd

Mortality records of 1,115 dairy cows were measured twice cross-sectionally in a research herd after a 9 year interval with the goal of understanding mortality patterns across two breeds of cattle.

- The overall mortality was 14.17%. Digestive problems followed by respiratory disorders together accounted for 70-80% of total deaths.

Kulangara, V., Joseph, A., Thrithamarassery, N., Sivasailam, A., Kalappurackal, L., Mattappillil, S., Mapranath, S. (2015). Epidemiology of bovine viral diarrhoea among tropical small holder dairy units in Kerala, India. *Tropical Animal Health and Production*, *47*(3), 575–579. https://doi.org/10.1007/s11250-015-0766-y

Prevalence of bovine viral diarrhoea among 385 dairy cattle reared under a smallholder system in Trichur District of Kerala State in India was determined through an ELISA targeting antibodies against p80-p125 non-structural protein of the virus.

- Prevalence was 24.7 % among the total population, but was higher (52 %) when 85 animals having infertility problems alone were considered.

Jagadeeswary, V., Reddy, M. S., & Satyanarayan, K. (2014). Ethno-veterinary practices used by tribals of Chittoor district, Andhra Pradesh, India. *Indian Journal of Animal Research*, *48*(3), 251. https://doi.org/10.5958/j.0976-0555.48.3.054

Qualitative methods such as semi-structured interviews used to learn about ethno-veterinary practices for livestock from 20 elders/ traditional healers in Chittoor district, Andhra Pradesh, India.

- Ethno-veterinary practices covered ailments covered including extra teeth, anorexia, stomatitis, bloat, constipation, diarrhoea, endoparasites, dysentery, cold and cough, ephemeral fever, drenching, pneumonia, FMD, repeat breeding, retained placenta, dystocia, metritis, mastitis, corneal opacity, ticks and lice, mange, fracture, leg pain, tetanus, horn avulsion, snake bite, poisonous plant consumption, yoke gall, abscess, tail gangrene, and allergic lesions.
- Ethno-veterinary studies are useful because they highlight common problems faced by smallholder farmers.

Panda, T., & Mishra, N. (2016). Indigenous Knowledge on Animal Health Care Practices in Kendrapara District of Odisha, India. *International Letters of Natural Sciences*, *53*, 10–27. https://doi.org/10.18052/www.scipress.com/ILNS.53.10

Cross-sectional study of ethnoveterinary practices in Kendrapara District of Odisha, India. Qualitative methods including participant observation and interview. Study mentions cattle, buffalo, sheep, and goats are commonly kept but does not match animal health concerns to species.

- The primary ailments of livestock are wound, diarrhoea, injury, fever, gastrointestinal problems and gynaecological problems, which are commonly treated with medicinal plants.

Bhatt, P. R., B. Pandya, K., Patel, U. D., Patel, H. B., & Modi, C. M. (2019). Survey on Ethnoveterinary Practices around Junagadh, Gujarat, India. *Indian Journal of Pharmaceutical Sciences*, *81*(1). https://doi.org/10.4172/pharmaceutical-sciences.1000493

Cross-sectional study of ethnoveterinary practices around Junagadh, Gujarat, India. A semi-structured questionnaire was administered to 82 male livestock owners. Study mentions cattle, buffalo, sheep, and goats are commonly kept but does not match animal health concerns to species.

- A total of 13 medicinal plants were cited 47 times for gastrointestinal problems, which were the highest.
- Other animal health concerns treated with ethnoveterinary medicine included retention foetal membrane, enteritis, cough and respiratory disease, wounds, skin problems, ringworm, foot conditions, vaginal and uterine prolapses, and bloat.

Khan, M. H., Manoj, K., & Pramod, S. (2008). Reproductive disorders in dairy cattle under semi-intensive system of rearing in North-Eastern India. *Veterinary World*, *9*(5), 512–518.

Longitudinal three-year study of 576 cross-bred dairy cows under semi-intensive management in North-eastern India to measure reproductive disorders.

- Repeat breeding (RB), anoestrus, retention of foetal membrane, and abortion were found to be the major clinical reproductive problems.
- Out of the total animal affected with reproductive disorders, the incidence of anoestrus, RB, retention of foetal membrane, and abortion was found to be 31.79% (n=62), 24.61% (n=48), 14.35% (n=28), and 11.25% (n=22), respectively.
- In addition, dystocia (5.12%), prolapse (1.53%), endometritis (4.61%), and pyometra (6.66%) were minor clinical reproductive problems.

Bangar, Y.C., Dohare, A. K., Kolekar, D. V., Avhad, S. R., & Khan, T. A. (2015). Seasonal variation in morbidity pattern in cattle by log-linear model approach. *Journal of Applied Animal Research*, *43*(3), 283–286. https://doi.org/10.1080/09712119.2014.963100

Longitudinal one-year study of disease seasonality amongst 1,538 dairy and beef cattle in Pune division of Maharashtra state of India.

- Overall incidence of all diseases was higher in winter (41.23%) followed by rainy season (39.18%) than in summer (19.59%).
- Overall incidences of reproductive diseases (17.84%) and digestive diseases (11.11%) were major problems throughout the study period.
- A log-linear modelling showed that the odds of incidences of reproductive diseases were significantly higher in summer as well as in rainy season than in winter season. Winter season was dominated by occurrences of digestive diseases as compared to any other diseases.

Thakur, R., Thakur, D., & Dogra, P. (2018). Indigenous feeding practices for better reproductive performance in buffaloes in Himachal Pradesh, India. *Buffalo Bulletin*, *37*(3), 361–368.

Participatory rural appraisal technique (qualitative methods including focus groups and key informant interviews) with rural dairy farmers keeping buffalo in Himachal Pradesh, India.

- Documents feeding practices used by rural farmers to prevent or treat common reproductive disorders including repeat breeding, early embryonic mortality, inability to carry the pregnancy full term, prolapse, dystocia, and retained placenta.

Malik, M., & Verma, H. (2018). Epidemiological aspect and major constraints in controlling haemorrhagic septicemia in dairy animals of Punjab. *The Indian Journal of Animal Sciences*, *88*(10), 1112–1117.

The cross-sectional study of haemorrhagic septicemia in dairy animals was conducted in all the different agro-climatic zones of Punjab by multistage stratified random sampling of 720 dairy farmers and categorized them on the basis of herd size of dairy animals, viz. small (5-10), medium (11-50) and large (>50).

- The overall incidence and case fatality rate of HS among dairy animals was 5.45 and 45.15% respectively.
- Incidence risk of HS on the species basis varied significantly and was observed highest in buffaloes (6.27%), followed by crossbred (4.67%) and indigenous cattle (3.91%).
- Within different herd sizes, the incidence risk of HS varied significantly and was highest in small herd size (10.52%) followed by medium (5.44%) and large herd size (3.01%).
- Incidence risk varied significantly between the different agro-climatic zones of Punjab within small and large sized herds but not in medium sized herds. CFR among the different herds was highest in small herd (49.28%) followed by medium herd (44.32%) and large herd (39.15%).
- The major constraints faced by farmers for controlling HS estimated by using Garrett's ranking technique included failure of prophylactic vaccination, cost of treatment, strict biosafety measures and hygienic sanitary conditions not followed, inadequate early disease detection and poor availability of veterinary and extension services.

Wright, A., & Thrusfield, M. (2016). Perceptions of zoonotic and animal diseases in the Van Gujjar community of North India. *Preventive Veterinary Medicine*, *123*, 143–153. https://doi.org/10.1016/j.prevetmed.2015.11.012

Cross-sectional participatory epidemiology study with four pastoralist groups of 35-55 participants per group in North India. The main livestock species was buffalo and many households economically depend on dairy. Some disease names remained untranslated in the article.

- Animal disease of most concern to pastoralists included trypanosomiasis, worms, foot and mouth disease, and diarrhoea.
- The community did not have a wide appreciation of zoonotic diseases, apart from rabies and potentially zoonotic skin diseases.

Kelly, T. R., Bunn, D. A., Joshi, N. P., Grooms, D., Devkota, D., Devkota, N. R., Mazet, J. A. K. (2018). Awareness and Practices Relating to Zoonotic Diseases Among Smallholder Farmers in Nepal. *EcoHealth*, *15*(3), 656–669. https://doi.org/10.1007/s10393-018-1343-4

Cross-sectional survey of 90 smallholder farmers, with a focus on buffalo, cattle, and zoonotic diseases.

- Across the study districts, several farmers reported health problems affecting their buffaloes (58%) and cattle (36%) over the previous year. The most commonly reported diseases in cattle and buffaloes by the farmers were fasciolosis (27%), tick infestation (12%), ringworm (10%), foot-and- mouth disease (FMD) (10%), and mastitis (7%). When unable to name a specific disease, farmers reported a wide range of clinical manifestations, including diarrhoea, respiratory disease, weight loss, skin lesions, haemorrhage, and lameness.
- Awareness of zoonotic pathogens was limited, especially in informally educated and illiterate farmers, the majority of which were women.
- Economically important tick-borne diseases (babesiosis and anaplasmosis) have recently been identified in cattle in western Nepal (Bohara 2014).

Kumar, R., Jain, S., Kumar, S. S., Sethi, K., Kumar, S. S., & Tripathi, B. N. (2017). Impact estimation of animal trypanosomosis (surra) on livestock productivity in India using simulation model: Current and future perspective. *Veterinary Parasitology, Regional Studies and Reports*, *10*, 1–12. https://doi.org/10.1016/j.vprsr.2017.06.008

The economic losses on livestock productivity were assessed resulting from animal trypanosomosis (surra) in India, considering all possible direct and indirect losses in major six livestock species: cattle, buffalo, goat, equine, camel and pig. The contemplative risk and retrospective analyses were performed using various official records and scientific literature complemented with expert data for evaluation of impact of surra on livestock productivity in India. Most of the information were derived using the secondary data published in scientific journals, and the official data reported by Basic Animal Husbandry and Fisheries Statistics, the Government of India, and other scientific reports.

- The true prevalence data for animal trypanosomosis are not available in cattle from the Indian subcontinent because cattle mostly carry a subclinical form of infection. Cites Kundu et al., 2013, reporting prevalence of trypanosomosis in cattle as 10.66% (95% CI: 8.19, 13.13) in three different States namely, Kerala, Odisha, and Uttar Pradesh covering three different agro-climatic zones.
- For buffalo, authors calculated aggregate of different prevalence values published in different journals. Using recorded data from the published reports covering maximum regions of India and in all, 320 buffaloes were found positive out of 7,703 screened, showing a prevalence of 4.15% (95% CI: 3.7, 4.6) in India.
- A total annual loss (direct and indirect) caused by animal trypanosomosis was estimated to US $ 671.1 million (US $ 344–US $ 1209 million at 95% confidence interval), at present valuation.

* The mortality losses were estimated to 16% of the total loss. Among morbidity losses, the reduction in milk yield and reproductive losses components were 36% and 26% of total loss, respectively. Other parameters like reduction in growth (10%), reduction in draught power (8%) and additional opportunity cost (3%) also yielded considerable loss.

Mondal, D. B., Sarma, K., & Saravanan, M. (2013). Upcoming of the integrated tick control program of ruminants with special emphasis on livestock farming system in India. *Ticks and Tick-Borne Diseases*, *4*(1–2), 1–10. https://doi.org/10.1016/j.ttbdis.2012.05.006

A literature review of tick-borne disease and tick control for livestock in India.

* A recent estimate calculated the costs of control of tick-borne diseases affecting Indian livestock as 498.7 million US $ per annum (Minjauw and McLeod, 2003).
* As per leather industry report, the leather sector of India is suffering from a huge shortfall of 3 billion pieces of hide and skin per year and causing 20–30% depreciation in normal value in the market. It has been estimated that India produces only 9.8%, 63.3%, 9.2%, and 6.0% of world cattle, buffalo, goat, and sheep hides, respectively, although the country possesses the highest livestock population.

Brookes, V. J., Gill, G. S., Singh, B. B., Sandhu, B. S., Dhand, N. K., Aulakh, R. S., & Ward, M. P. (2019). Challenges to human rabies elimination highlighted following a rabies outbreak in bovines and a human in Punjab, India. *Zoonoses and Public Health*, *66*(3), 325–336. https://doi.org/10.1111/zph.12568

Retrospective investigation of rabies outbreak in bovines in Punjab, India and cross-sectional knowledge, attitudes, practices survey about rabies prevention focused on human health risks.

* Rabies outbreak in bovines reported in August 2015 in a Punjab village. The number of affected bovines was unusually high; 15 cattle and buffalo died on 13 smallholder farms (attack rate 4%).

Sangma, D. B., & Manohara, T. (2018). The role of Garo tribes of Meghalaya (India) in the conservation and management of medicinal plants diversity used in treating livestock diseases. *Plant Science Today*, *5*(4), 155. https://doi.org/10.14719/pst.2018.5.4.416

Cross-sectional study of ethnoveterinary practices of Garo tribes of Meghalaya (India). Qualitative methods included group discussion and semi-structured interviews. Study mentions cattle, buffalo, sheep, and goats are commonly kept but does not match animal health concerns to species.

* Animal health concerns with the highest consensus of appropriate ethnoveterinary treatment were arthritis, rabies, reproductive diseases, retained placenta, and swollen body parts.

- Other health concerns commonly treated include diarrhoea, wounds, and ectoparasites.

Whittington, R., Donat, K., Weber, M. F., Kelton, D., Nielsen, S. S., Eisenberg, S., de Waard, J. H. (2019). Control of paratuberculosis: who, why and how. A review of 48 countries. *BMC Veterinary Research*, *15*(1), 198. https://doi.org/10.1186/s12917-019-1943-4

Cross-sectional survey of collaborators in 48 countries including India requesting regional-level data in respective countries from 2012–2018 for Johne's disease (paratuberculosis) in cattle.

- Johne's disease was not considered notifiable for India, meaning it was not reported for any region in India from 2012-2018.

Singh, S. V., Singh, A. V., Singh, R., Sharma, S., Shukla, N., Misra, S., Sandhu, K. S. (2008). Sero-prevalence of Bovine Johne's disease in buffaloes and cattle population of North India using indigenous ELISA kit based on native Mycobacterium avium subspecies paratuberculosis 'Bison type' genotype of goat origin. *Comparative Immunology, Microbiology and Infectious Diseases*, *31*(5), 419–433. https://doi.org/10.1016/j.cimid.2007.06.002

Cross-sectional serosurvey to measure the prevalence of bovine Johne's disease in buffalo and cattle. Serum samples were analysed from 726 cattle and buffalo from 33 randomly selected villages in North India.

- Seroprevalence was 23.3% (12.1% in young and 24.4% in adults) and 26.9% (27.2% in young and 26.8% in adults) in buffaloes and cattle, respectively.
- Major livestock problems reported include reproductive problems, FMD, HS, internal and external parasites, flukes, and mastitis.

Yadav, A., Panadero, R., Katoch, R., Godara, R., & Cabanelas, E. (2017). Myiasis of domestic and wild ruminants caused by Hypodermatinae in the Mediterranean and Indian subcontinent. *Veterinary Parasitology*, *243*, 208–218. https://doi.org/10.1016/j.vetpar.2017.07.007

A review of myiasis (warble fly infestation) in cattle, sheep, and goats in the Mediterranean and India subcontinent.

- Bovine infestation is mainly reported in the northern states of India with prevalence ranging from 9.73% (Yadav et al., 2013) to 72.46% (Kumar et al., 1990).

Sudan, V., Jaiswal, A. K., & Shanker, D. (2014). Infection rates of *Linguatula serrata* nymphs in mesenteric lymph nodes from water buffaloes in North India. *Veterinary Parasitology*, *205*(1–2), 408–411. https://doi.org/10.1016/j.vetpar.2014.07.025

Cross-sectional abattoir study to measure the infection rates of Linguatula serrata nymphs in mesenteric lymph nodes of water buffalo. A total of 480 water buffalo were examined.

- Results revealed 88 (18.3%) buffaloes and 288 (20.0%) mesenteric lymph nodes having parasite's nymphs.

Murugeswari, R., Valli, C., Karunakaran, R., Leela, V., & Pandian, A. S. S. (2018). Prevalence and magnitude of acidosis sequelae to rice-based feeding regimen followed in Tamil Nadu, India. *Veterinary World*, *11*(4), 464–468. https://doi.org/10.14202/vetworld.2018.464-468

A cross-sectional survey was conducted with 200 randomly selected dairy farmers in all 32 districts of Tamil, Nadu, India. Dairy farmers belonged to the unorganized sector and owned one or two cross-bred dairy cows. Occurrence of acidosis and incidence of laminitis were ascertained by a veterinarian with the confirmative test to determine the impact of feeding cooked rice to cows.

- 71.5% of farmers feed cooked rice to their cattle.
- Incidence of acidosis progressively increased from 29% in cows fed with 0.5 kg of cooked rice to 69% in cows fed with more than 2.5 kg of cooked rice.
- Among cooked rice-based diet, the incidence of laminitis increased progressively ($p<0.05$) from 9.2% to 37.9% with the increase in the quantity of cooked rice in the diet.

Kathiravan, G., Thirunavukkarasu, & Michaelraj, P. (2007). Willingness to pay for annual health care services in small ruminants: The case of South India. *Journal of Applied Sciences*, *7*(16), 2361–2365.

Willingness to pay survey with 320 small ruminant farmers from four districts in South India.

- At a service centre, the estimated willingness to pay (WTP) was 56 INR for sheep and 61 INR for goats. At the farmer's doorstep, the estimated WTP was 87 IND for sheep and 95 INR for goats.

Kathiravan, G., Thirunavukkarasu, M., & Selvakumar, K. N. (2005). Cost of livestock services: The case of Tamil Nadu (India). *Journal of Applied Sciences Research*, (No.October), 1195–1205.

Cross-sectional survey of 320 respondents selected through multi-stage sampling procedure seeking services from public veterinary centres to learn about the prices paid for animal health services. The estimated exchange rate from June 15, 2007 is 40.6 INR to $1 USD. No known web access to article.

- The mean cost of treatment of a chronic medical case in cattle at a public veterinary centre was INR 20.83, in which the labour cost alone accounted to INR 17.35, with the remaining amount for the drugs purchased outside. However, the mean costs of treating a chronic medical condition in buffalo and small ruminant at public veterinary centres were only INR 13.34 and INR 10.80, respectively.
- Although no charges were made for animal health care services rendered at public veterinary centres, the charges in terms of imputed labour cost for bringing the animal to the centre was incurred.
- Service fee accounted for more than 60 per cent of cost of treatment for home service by a veterinarian or a para-veterinarian.
