# Supplementary material for: Prioritizing smallholder animal health needs in East Africa, West Africa, and South Asia using three approaches: Literature review, expert workshops, and practitioner surveys
Source: Prev Vet Med. 2021 Apr;189:105279. doi: 10.1016/j.prevetmed.2021.105279 (PMC8024747; doi:10.1016/j.prevetmed.2021.105279)
Supplement: Supplementary file 10 [file mmc10.pdf]

Smallholder Animal Health Needs Assessment
South Asia Poultry

GALVmed

August 19, 2019

Assembled by Zoë Campbell, Peetambar Kushwaha, and Paul Coleman

Meta-analysis summary

The literature review yielded 16 articles, 9 of which focused on impact, incidence, and prevalence, and are summarized below. The table below shows the number of articles in which each animal health concern appears in the literature about poultry in South Asia. Many articles mention multiple concerns. No articles were given a green ranking for focus on both incidence/ prevalence and impact. The summarized articles are in the bibliography within this document. The full list of articles considered in the South Asia meta-analysis is available as a separate document.

The most mentioned concerns in both the meta-analysis and the impact articles are Newcastle disease and avian influenza. Coccidiosis is a secondary concern. The research following highly pathogenic avian influenza outbreaks in Bangladesh within the time frame of this literature review may be giving it more weight than it deserves as a constraint to smallholder poultry production.

**Animal health concern**	**Frequency of mention (meta-analysis)**	**Frequency of mention (impact articles)**
Newcastle disease	5	2
Avian influenza	3	3
Coccidiosis	2	2
Salmonellosis	2	0
Acephate exposure	1	0
Diarrhea	1	1
Fowl cholera	1	0
Rabies	1	1
STEC*	1	1

*STEC - Shiga toxin producing *Escherichia coli*

Table of articles

Abbreviations:

ND – Newcastle disease

AI – Avian influenza

Note: Clicking on the article number links to an expanded summary of the article below. The “web” link connects to the abstract of the article online or to full text for open access articles.

#	Citation	Country	Study design	Pathogen/ disease	Findings
1<br>2<br>web	(Belgrad et al., 2018)	Bangladesh	Cross-sectional, Serosurvey, 245 chickens from 129 rural, smallholder households in two sub-districts	Newcastle disease	**Incidence/ prevalence:** The overall household level ND seroprevalence based on ELISA was 31.8% (41/129) (95% CI: 23.9–40.6%), whereas the overall bird level ND seroprevalence based on ELISA was 21.2% (52/245) (95% CI: 16.5–26.8%). ND prevalence based on RT-PCR was 12.5% (4/32) (95% CI: 3.5–29.0%).
2<br>2<br>web	(Bessell et al., 2017)	India<br>Nepal<br>Tanzania	Intervention analysis, Questionnaire, Smallholder farmers,	Newcastle disease	**Impact:** Intervention increased vaccination rates for ND to over 75% uptake in all areas, reaching 98% in India. In all areas flock sizes doubled, the numbers of eggs that were set for hatching and that hatched increased by 25–50% and there was an increase in the frequency with which chicken meat was consumed and chickens were sold. The

			420 households in India, 409 in Nepal		increases in production after increasing vaccination coverage reflect the high impact of ND in the project areas.
3<br>2<br>web	(G. Govindaraj et al., 2018)	Bangladesh	Cross-sectional survey of 91 poultry farms from multiple production systems, secondary government expense data	Avian influenza	**Impact:** The estimated loss (culling live birds, eggs and feed destruction) per duck farm was USD 9,181, USD 3,889 and USD 156 in case of commercial farms reared for meat, dual-purpose and backyard farms, respectively. The loss incurred by small-scale broiler and backyard poultry farms was USD 453 and USD 40, respectively. The loss incurred by large and small duck hatcheries was USD 11,963 and USD 5,790, respectively, due to culling of hatchlings, young birds and destroying eggs. The government invested USD 744,890 to contain the disease spread through massive culling, surveillance and monitoring of poultry and humans due to zoonotic nature of the disease. A sharp market reaction on own and substitute product prices and eight weeks' time lag in price recovery was observed.
4<br>2<br>web	(Biswas et al., 2011)	Bangladesh	Retrospective study of 33 commercial and 25 backyard farms recorded during 2007 outbreaks	Avian influenza	**Impact:** After the clinical onset, the incidence rates of mortality in breeder, commercial broiler, commercial layer, and backyard chickens were, respectively: 0.0215, 0.0341, 0.0179 and 0.0703 per chicken-day at risk.

5<br>3<br>web	(Osmani, Ward, Giasuddin, Islam, & Kalam, 2014)	Bangladesh	Outbreak reports from 2007-2014, Multiple production systems	Avian influenza	**Incidence/ prevalence:** Highly pathogenic avian influenza H5N1 was first reported in Bangladesh in 2007, and since then 546 outbreaks have been reported to the World Organization for Animal Health (OIE).
6<br>2<br>web	(Chengat Prakashbabu et al., 2017)	India	Cross-sectional prevalence survey of 240 commercial poultry farms in north and south India	Coccidiosis *Eimeria* species	**Incidence/ prevalence:** In the present study 79.4% (85/107) and 76% (101/133) of farms from northern and southern India were found to be positive for any *Eimeria* by oocyst microscopy and genus-specific PCR. All *Eimeria* species were detected in north India, and only *E. praecox* was undetected in the south. *Eimeria tenella*, followed by *E. mitis*, were the most common species detected in both sampled regions. *Eimeria acervulina* and *E. necatrix* were also common, with the former ranking higher in the north and the latter in the south.
7<br>3<br>web	(Hinsu et al., 2018)	India	Cross-sectional, Prevalence study within experimental study of new diagnostic tool, 600 chickens (Cobb400 and indigenous chickens raised in	Coccidiosis *Eimeria* species	**Incidence/ prevalence:** *Eimeria tenella* was detected in all groups, on average representing between 56 and 75% of reads from Cobb400 birds reared in mixed and single breed flocks respectively, and 60 and 70% of Kadaknath from mixed and single breed flocks.

			single and mixed breed flocks)		
8<br>3<br>web	(Dutta, Roychoudhury, Bandyopadhyay, Wani, & Hussain, 2011)	India	Outbreak investigation, Molecular characterization	Shiga toxin producing Escherichia coli (STEC), Diarrhoea	**Incidence/ prevalence:** This is the first report of the involvement of Shiga toxin producing Escherichia coli (STEC) in poultry in India.
9<br>3<br>web	(Baby et al., 2015)	India	Test of single chicken carcass bitten by stray dog brought to rabies diagnostic laboratory	Rabies	This case of naturally acquired rabies infection in a bird species, *Gallus domesticus*, being reported for the first time in India, was identified from an area which has a significant stray dog population and is highly endemic for canine rabies. It indicates that spillover of infection even to an unusual host is possible in highly endemic areas.

Article summaries

Belgrad, J. P., Rahman, M. A., Abdullah, M. S., Rashid, M. H., Sayeed, M. A., Anwer, M. S., & Hoque, M. A. (2018). Newcastle disease sero and viro-prevalence in rural poultry in Chittagong, Bangladesh. *Preventive Veterinary Medicine*, *160*, 18–25.

This cross-sectional study randomly sampled 129 rural households with 245 chickens for the seroprevalence and active infection rate of Newcastle disease amongst rural chickens in two selected sub-districts of the Chittagong district, Bangladesh.

- The overall household level ND seroprevalence based on ELISA was 31.8% (41/129) (95% CI: 23.9–40.6%), whereas the overall bird level ND seroprevalence based on ELISA was 21.2% (52/245) (95% CI: 16.5–26.8%). ND prevalence based on RT-PCR was 12.5% (4/32) (95% CI: 3.5–29.0%).

Bessell, P., Kushwaha, P., Mosha, R., Woolley, R., Al-Riyami, L., & Gammon, N. (2017). Assessing the impact of a novel strategy for delivering animal health interventions to smallholder farmers. *Preventive Veterinary Medicine*, *147*(August), 108–116. https://doi.org/10.1016/j.prevetmed.2017.08.022

Cross-sectional questionnaire administered to 420 smallholder households in India and 409 in Nepal to assess effect of an intervention to deliver Newcastle disease vaccines and sensitize farmers.

- Intervention increased vaccination rates for ND to over 75% uptake in all areas, reaching 98% in India. In all areas flock sizes doubled, the numbers of eggs that were set for hatching and that hatched increased by 25–50% and there was an increase in the frequency with which chicken meat was consumed and chickens were sold.
- The increases in production after increasing vaccination coverage reflect the high impact of ND in the project areas.

Govindaraj, G., Sridevi, R., Nandakumar, S. N., Vineet, R., Rajeev, P., Binu, M. K., Rahman, H. (2018). Economic impacts of avian influenza outbreaks in Kerala, India. *Transboundary and Emerging Diseases*, *65*(2), e361–e372. https://doi.org/10.1111/tbed.12766

Cross-sectional survey of 91 poultry farms from multiple production systems to retrospectively learn about the economic impacts of avian influenza from areas in Kerala, India after outbreaks of avian influenza in 2014. Secondary expense data were also used.

- The estimated loss (culling live birds, eggs and feed destruction) per duck farm was USD 9,181, USD 3,889 and USD 156 in case of commercial farms reared for meat, dual-purpose and backyard farms, respectively.

- The loss incurred by small-scale broiler and backyard poultry farms was USD 453 and USD 40, respectively.
- The loss incurred by large and small duck hatcheries was USD 11,963 and USD 5,790, respectively, due to culling of hatchlings, young birds and destroying eggs.
- The government invested USD 744,890 to contain the disease spread through massive culling, surveillance and monitoring of poultry and humans due to zoonotic nature of the disease.
- A sharp market reaction on own and substitute product prices and eight weeks' time lag in price recovery was observed.

Biswas, P., Christensen, J., Ahmed, S., Barua, H., Das, A., Rahman, M., Debnath, N. (2011). Mortality rate and clinical features of highly pathogenic avian influenza in naturally infected chickens in Bangladesh. *Scientific and Technical Review of the Office International Des Epizooties*, *30*(3), 871–878.

Retrospective study of all 33 commercial and 25 backyard farms recorded during the January-November, 2007 outbreaks of highly pathogenic avian influenza in Bangladesh.

- After the clinical onset, the incidence rates of mortality in breeder, commercial broiler, commercial layer, and backyard chickens were, respectively: 0.0215, 0.0341, 0.0179 and 0.0703 per chicken-day at risk.

Osmani, M. G., Ward, M. P., Giasuddin, M., Islam, M. R., & Kalam, A. (2014). The spread of highly pathogenic avian influenza (subtype H5N1) clades in Bangladesh, 2010 and 2011. *Preventive Veterinary Medicine*, *114*(1), 21–27. https://doi.org/10.1016/j.prevetmed.2014.01.010

Outbreak data reported to the World Organization for Animal Health (OIE) for avian influenza in Bangladesh from 2007-2014 was summarized.

- Highly pathogenic avian influenza H5N1 was first reported in Bangladesh in 2007, and since then 546 outbreaks have been reported to the World Organization for Animal Health (OIE).
- The disease has apparently become endemic in Bangladesh.

Chengat Prakashbabu, B., Thenmozhi, V., Limon, G., Kundu, K., Kumar, S., Garg, R., Blake, D. P. (2017). Eimeria species occurrence varies between geographic regions and poultry production systems and may influence parasite genetic diversity. *Veterinary Parasitology*, *233*, 62–72. https://doi.org/10.1016/j.vetpar.2016.12.003

Cross-sectional prevalence survey of *Eimeria* species in chickens in 240 commercial poultry farms in north and south India.

- In the present study 79.4% (85/107) and 76% (101/133) of farms from northern and southern India were found to be positive for any *Eimeria* by oocyst microscopy and genus-specific PCR.
- All *Eimeria* species were detected in north India, and only *E. praecox* was undetected in the south.
- *Eimeria tenella*, followed by *E. mitis*, were the most common species detected in both sampled regions. *Eimeria acervulina* and *E. necatrix* were also common, with the former ranking higher in the north and the latter in the south.

Hinsu, A. T., Thakkar, J. R., Koringa, P. G., Vrba, V., Jakhesara, S. J., Psifidi, A., Blake, D. P. (2018). Illumina Next Generation Sequencing for the Analysis of Eimeria Populations in Commercial Broilers and Indigenous Chickens. *Frontiers in Veterinary Science*, *5*, 176. https://doi.org/10.3389/fvets.2018.00176

Cross-sectional, prevalence study within experimental study of new diagnostic tool (Illumina next generation sequencing). 600 chickens (Cobb400 and indigenous chickens raised in single and mixed breed flocks) were raised by 30 participating farms using standard husbandry protocols.

- *Eimeria tenella* was detected in all groups, on average representing between 56 and 75% of reads from Cobb400 birds reared in mixed and single breed flocks respectively, and 60 and 70% of Kadaknath from mixed and single breed flocks.

Dutta, T. K., Roychoudhury, P., Bandyopadhyay, S., Wani, S. A., & Hussain, I. (2011). Detection and characterization of Shiga toxin producing Escherichia coli (STEC) and enteropathogenic Escherichia coli (EPEC) in poultry birds with diarrhoea. *The Indian Journal of Medical Research*, *133*, 541–545.

An outbreak of acute diarrhoea in poultry birds at Aizawl, Mizoram was investigated for detection and characterization of STEC and enteropathogenic *E. coli* (EPEC).

- This is the first report of the involvement of Shiga toxin producing *Escherichia coli* (STEC) in poultry in India.

Baby, J., Mani, R. S., Abraham, S. S., Thankappan, A. T., Pillai, P. M., Anand, A. M., Sreekumar, S. (2015). Natural Rabies Infection in a Domestic Fowl (Gallus domesticus): A Report from India. *PLoS Neglected Tropical Diseases*, *9*(7), e0003942. https://doi.org/10.1371/journal.pntd.0003942

Report of a single chicken carcass bitten by stray dog brought to rabies diagnostic laboratory in India.

- This case of naturally acquired rabies infection in a bird species, *Gallus domesticus*, being reported for the first time in India, was identified from an area which has a significant stray dog population and is highly endemic for canine rabies. It indicates that spill over of infection even to an unusual host is possible in highly endemic areas.
