# Supplementary material for: Prioritizing smallholder animal health needs in East Africa, West Africa, and South Asia using three approaches: Literature review, expert workshops, and practitioner surveys
Source: Prev Vet Med. 2021 Apr;189:105279. doi: 10.1016/j.prevetmed.2021.105279 (PMC8024747; doi:10.1016/j.prevetmed.2021.105279)
Supplement: Supplementary file 11 [file mmc11.pdf]

South Asia References

The references below were used for meta-analyses for all livestock types (bovids such as cattle, buffalo, mithun, yak; small ruminants; and poultry). The sub-set of summarized articles are also included.

Ahir, V. B., Roy, A., Jhala, M. K., Bhanderi, B. B., Mathakiya, R. A., Bhatt, V. D., Joshi, C. G. (2011). Genome Sequence of Pasteurella multocida subsp. gallicida Anand1_poultry. *Journal of Bacteriology*, *193*(19), 5604–5604. https://doi.org/10.1128/JB.05706-11

Baby, J., Mani, R. S., Abraham, S. S., Thankappan, A. T., Pillai, P. M., Anand, A. M., Sreekumar, S. (2015). Natural Rabies Infection in a Domestic Fowl (Gallus domesticus): A Report from India. *PLoS Neglected Tropical Diseases*, *9*(7), e0003942. https://doi.org/10.1371/journal.pntd.0003942

Balamurugan, V., Das, S., Raju, D. S. N., Chakravarty, I., Nagalingam, M., Hemadri, D., Rahman, H. (2014). Prevalence of peste des petits ruminants in goats in North-East India. *VirusDisease*, *25*(4), 488–492. https://doi.org/10.1007/s13337-014-0237-5

Balamurugan, V., Saravanan, P., Sen, A., Rajak, K. K., Bhanuprakash, V., Krishnamoorthy, P., & Singh, R. K. (2011). Sero-epidemiological study of peste des petits ruminants in sheep and goats in India between 2003 and 2009. *Revue Scientifique et Technique (International Office of Epizootics)*, *30*(3), 889–896.

Bangar, Y. C., Dohare, A. K., Kolekar, D. V., Avhad, S. R., & Khan, T. A. (2015). Seasonal variation in morbidity pattern in cattle by log-linear model approach. *Journal of Applied Animal Research*, *43*(3), 283–286. https://doi.org/10.1080/09712119.2014.963100

Bangar, Y. C., Singh, B., Dohare, A. K., & Verma, M. R. (2015). A systematic review and meta-analysis of prevalence of subclinical mastitis in dairy cows in India. *Tropical Animal Health and Production*, *47*(2), 291–297. https://doi.org/10.1007/s11250-014-0718-y

Banumathi, B., Vaseeharan, B., Rajasekar, P., Prabhu, N. M., Ramasamy, P., Murugan, K., Benelli, G. (2017). Exploitation of chemical, herbal and nanoformulated acaricides to control the cattle tick, Rhipicephalus (Boophilus) microplus - A review. *Veterinary Parasitology*, *244*, 102–110. https://doi.org/10.1016/j.vetpar.2017.07.021

Belgrad, J. P., Rahman, M. A., Abdullah, M. S., Rashid, M. H., Sayeed, M. A., Anwer, M. S., & Hoque, M. A. (2018). Newcastle disease sero and viro-prevalence in rural poultry in Chittagong, Bangladesh. *Preventive Veterinary Medicine*, *160*, 18–25.

Bessell, P. P. R., Kushwaha, P., Mosha, R., Woolley, R., Al-Riyami, L., & Gammon, N. (2017). Assessing the impact of a novel strategy for delivering animal health interventions to smallholder farmers. *Preventive Veterinary Medicine*, *147*(August), 108–116. https://doi.org/10.1016/j.prevetmed.2017.08.022

Bhanotra, A., & Gupta, J. (2016). Mapping of indigenous technical knowledge (ITK) on animal healthcare and validation of ITKs used for treatment of pneumonia in dairy animals. *Indian Journal of Traditional Knowledge*, *15*(2), 297–303.

Bhanuprakash, V., Hosamani, M., & Singh, R. K. (2011). Prospects of control and eradication of capripox from the Indian subcontinent: A perspective. *Antiviral Research*, *91*(3), 225–232. https://doi.org/10.1016/j.antiviral.2011.06.004

Bhatt, P. R., B. Pandya, K., Patel, U. D., Patel, H. B., & Modi, C. M. (2019). Survey on Ethnoveterinary Practices around Junagadh, Gujarat, India. *Indian Journal of Pharmaceutical Sciences*, *81*(1). https://doi.org/10.4172/pharmaceutical-sciences.1000493

Biswas, P., Christensen, J., Ahmed, S., Barua, H., Das, A., Rahman, M., Debnath, N. (2011). Mortality rate and clinical features of highly pathogenic avian influenza in naturally infected chickens in Bangladesh. *Scientific and Technical Review of the Office International Des Epizooties*, *30*(3), 871–878.

Borah, B., Deka, P., Sharma, K., Baro, S., Hazarika, A. K., Das, C., Ltu, K. (2018). Isolation, identification and retrospective study of foot-and-mouth disease virus from affected Mithun (Bos frontalis) in north-eastern India. *Transboundary and Emerging Diseases*, *65*(1), e63–e69. https://doi.org/10.1111/tbed.12678

Brar, P., & Nanda, A. (2008). Postpartum Ovarian Activity in South Asian Zebu Cattle. *Reproduction in Domestic Animals*, *43*, 207–212. https://doi.org/10.1111/j.1439-0531.2008.01163.x

Brookes, V. J., Gill, G. S., Singh, B. B., Sandhu, B. S., Dhand, N. K., Aulakh, R. S., & Ward, M. P. (2019). Challenges to human rabies elimination highlighted following a rabies outbreak in bovines and a human in Punjab, India. *Zoonoses and Public Health*, *66*(3), 325–336. https://doi.org/10.1111/zph.12568

Chakraborty, S., Dhama, K., Tiwari, R., Iqbal Yatoo, M., Khurana, S. K., Khandia, R., Chaicumpa, W. (2019). Technological interventions and advances in the diagnosis of intramammary infections in animals with emphasis on bovine population -a review. *The Veterinary Quarterly*, 1–30. https://doi.org/10.1080/01652176.2019.1642546

Chauhan, H. C., Patel, B. K., Bhagat, A. G., Patel, M. V, Patel, S. I., Raval, S. H., Chandel, B. S. (2015). Comparison of molecular and microscopic technique for detection of Theileria

annulata from the field cases of cattle. *Veterinary World*, *8*(11), 1370–1374. https://doi.org/10.14202/vetworld.2015.1370-1374

Chauhan, H. C., Lambade, P. S., Sen, A., Dadawala, A. I., Ranaware, P. B., Chandel, B., Kher, H. N. (2011). The use of pathological and histopathological techniques in the diagnosis of peste des petits ruminants in India. *Veterinaria Italiana*, *47*(1), 41–47.

Chengat Prakashbabu, B., Thenmozhi, V., Limon, G., Kundu, K., Kumar, S., Garg, R., Blake, D. P. (2017). Eimeria species occurrence varies between geographic regions and poultry production systems and may influence parasite genetic diversity. *Veterinary Parasitology*, *233*, 62–72. https://doi.org/10.1016/j.vetpar.2016.12.003

Chhetri, B. K., Perez, A. M., & Thurmond, M. C. (2010). Factors associated with spatial clustering of foot-and-mouth disease in Nepal. *Tropical Animal Health and Production*, *42*(7). https://doi.org/10.1007/s11250-010-9573-7

Choudhary, M., Choudhary, B. K., Chandra Ghosh, R., Bhoyar, S., Chaudhari, S., & Barbuddhe, S. B. (2019). Cultivable microbiota and pulmonary lesions in polymicrobial bovine pneumonia. *Microbial Pathogenesis*, *134*, 103577. https://doi.org/10.1016/j.micpath.2019.103577

Choudhary, V., Garg, S., Chourasia, R., Hasnani, J. J., Patel, P. V, Shah, T. M., Joshi, C. G. (2015). Transcriptome analysis of the adult rumen fluke Paramphistomum cervi following next generation sequencing. *Gene*, *570*(1), 64–70. https://doi.org/10.1016/j.gene.2015.06.002

Dar, M. A., Ahmed, R., Urwat, U., Ahmad, S. M., Dar, P. A., Kushoo, Z. A., Heidari, M. (2018). Expression kinetics of natural resistance associated macrophage protein (NRAMP) genes in Salmonella Typhimurium-infected chicken. *BMC Veterinary Research*, *14*(1), 180. https://doi.org/10.1186/s12917-018-1510-4

Dar, M. A., Urwat, U., Ahmad, S. M., Ahmad, R., Kashoo, Z. A., Dar, T. A., Heidari, M. (2019). Gene expression and antibody response in chicken against Salmonella Typhimurium challenge. *Poultry Science*, *98*(5), 2008–2013. https://doi.org/10.3382/ps/pey560

Dar, P. A., Hajam, I. A., Suryanarayana, V. S., Kishore, S., & Kondabattula, G. (2015). Kinetics of cytokine expression in bovine PBMCs and whole blood after in vitro stimulation with foot-and-mouth disease virus (FMDV) antigen. *Cytokine*, *72*(1), 58–62. https://doi.org/10.1016/j.cyto.2014.12.011

Deb, R., Kumar, A., Chakraborty, S., Verma, A., Tiwari, R., Dhama, K., Kumar, S. (2013). Trends in diagnosis and treatment of bovine mastitis: A review. *Pakistan Journal of Biological Sciences*, *16*(23), 1653–1661.

Dutta, T. K., Roychoudhury, P., Bandyopadhyay, S., Wani, S. A., & Hussain, I. (2011). Detection and characterization of Shiga toxin producing Escherichia coli (STEC) and enteropathogenic Escherichia coli (EPEC) in poultry birds with diarrhoea. *The Indian Journal of Medical Research*, *133*, 541–545.

Govindaraj, G., Sridevi, R., Nandakumar, S. N., Vineet, R., Rajeev, P., Binu, M. K., Rahman, H. (2018). Economic impacts of avian influenza outbreaks in Kerala, India. *Transboundary and Emerging Diseases*, *65*(2), e361–e372. https://doi.org/10.1111/tbed.12766

Govindaraj, G. N., Roy, G., Mohanty, B. S., Balamurugan, V., Pandey, A. K., Sharma, V., Roy, P. (2019). Evaluation of effectiveness of Mass Vaccination Campaign against Peste des petits ruminants in Chhattisgarh state, India. *Transboundary and Emerging Diseases*, *66*(3), 1349–1359. https://doi.org/10.1111/tbed.13163

Gowda, R. N. S. (2005). An epidemiological study of sheep pox infection in Karnataka state, India. *OIE Revue Scientifique et Technique*, *24*(3), 909–920. https://doi.org/10.20506/rst.24.3.1621

Gururaj, K., Pawaiya, R. S., Gangwar, N. K., Mishra, A. K., Singh, D. D., Andani, D., Sharma, D. K. (2019). Comparative molecular characterization and phylogenetic analysis of cerebral and non-cerebral coenurosis in Indian goats. *Veterinary Parasitology, Regional Studies and Reports*, *15*, 100266. https://doi.org/10.1016/j.vprsr.2019.100266

Hegde, R., Gomes, A. R., Muniyellappa, H. K., Byregowda, S. M., Giridhar, P., & Renukaprasad, C. (2009). A short note on peste des petits ruminants in Karnataka, India. *Revue Scientifique et Technique (International Office of Epizootics)*, *28*(3), 1031–1035.

Hinsu, A. T., Thakkar, J. R., Koringa, P. G., Vrba, V., Jakhesara, S. J., Psifidi, A., Blake, D. P. (2018). Illumina Next Generation Sequencing for the Analysis of Eimeria Populations in Commercial Broilers and Indigenous Chickens. *Frontiers in Veterinary Science*, *5*, 176. https://doi.org/10.3389/fvets.2018.00176

Hota, A., Biswal, S., Sahoo, N., Venkatesan, G., Arya, S., Kumar, A., Rout, M. (2018). Seroprevalence of Capripoxvirus infection in sheep and goats among different agro-climatic zones of Odisha, India. *Veterinary World*, *11*(1), 66–70. https://doi.org/10.14202/vetworld.2018.66-70

Malik, M., & Verma, H. (2018). Epidemiological aspect and major constraints in controlling haemorrhagic septicemia in dairy animals of Punjab. *The Indian Journal of Animal Sciences*, *88*(10), 1112–1117.

Iqbal Yatoo, M., Raffiq Parray, O., Tauseef Bashir, S., Ahmed Bhat, R., Gopalakrishnan, A., Karthik, K., Vir Singh, S. (2019). Contagious caprine pleuropneumonia - a comprehensive review. *The Veterinary Quarterly*, *39*(1), 1–25. https://doi.org/10.1080/01652176.2019.1580826

Islam, M. A., Khatun, M. M., Werre, S. R., Sriranganathan, N., & Boyle, S. M. (2013). A review of Brucella seroprevalence among humans and animals in Bangladesh with special emphasis on epidemiology, risk factors and control opportunities. *Veterinary Microbiology*, *166*(3–4), 317–326. https://doi.org/10.1016/j.vetmic.2013.06.014

Jackson, D. S., Nydam, D. V, & Altier, C. (2014). Prevalence and risk factors for brucellosis in domestic yak Bos grunniens and their herders in a transhumant pastoralist system of Dolpo, Nepal. *Preventive Veterinary Medicine*, *113*(1), 47–58.

Jagadeeswary, V., Reddy, M. S., & Satyanarayan, K. (2014). Ethno-veterinary practices used by tribals of Chittoor district, Andhra Pradesh, India. *Indian Journal of Animal Research*, *48*(3), 251. https://doi.org/10.5958/j.0976-0555.48.3.054

Jaiswal, A. K., Sudan, V., Kumar, P., Srivastava, A., & Shanker, D. (2016). Bovine hypodermosis in indigenous cattle herd and its successful therapeutic management. *Journal of Parasitic Diseases : Official Organ of the Indian Society for Parasitology*, *40*(1), 166–168. https://doi.org/10.1007/s12639-014-0470-2

Jakhesara, S. J., Prasad, V. V. S. P., Pal, J. K., Jhala, M. K., Prajapati, K. S., & Joshi, C. G. (2016). Pathotypic and Sequence Characterization of Newcastle Disease Viruses from Vaccinated Chickens Reveals Circulation of Genotype II, IV and XIII and in India. *Transboundary and Emerging Diseases*, *63*(5), 523–539. https://doi.org/10.1111/tbed.12294

Kalleshamurthy, T., Shekar, R., Niranjanamurthy, H. H., Natesan, K., Shome, B. R., Bambal, R. G., Shome, R. (2018). Assessment of fluorescence polarization assay: a candid diagnostic tool in Brucella abortus strain 19 vaccinated areas. *Microbiology and Immunology*, *62*(11), 694–701. https://doi.org/10.1111/1348-0421.12654

Kamal, M., Mondal, S., Islam, S., & Islam, M. (2012). Present status of goat rearing under rural conditions in south-west regions of Bangladesh. In G. Rahmann & D. Godinho (Eds.), *Organic Animal Husbandry Challenges*. Hamburg/ Trenthorst, Germany.

Kanani, A., Dabhi, S., Patel, Y., Chandra, V., Kumar, O. R. V., & Shome, R. (2018). Seroprevalence of brucellosis in small ruminants in organized and unorganized sectors of Gujarat state, India. *Veterinary World*, *11*(8), 1030–1036. https://doi.org/10.14202/vetworld.2018.1030-1036

Kant, N., Kulshreshtha, P., Singh, R., Mal, A., Dwivedi, A., Ahuja, R., Bhatnagar, R. (2018). A study to identify the practices of the buffalo keepers which inadvertently lead to the spread

of brucellosis in Delhi. *BMC Veterinary Research*, *14*(1), 329. https://doi.org/10.1186/s12917-018-1670-2

Kathiravan, G., Thirunavukkarasu, M., & Selvakumar, K. N. (2005). Cost of livestock services: The case of Tamil Nadu (India). *Journal of Applied Sciences Research*, (No.October), 1195–1205.

Kathiravan, G., Thirunavukkarasu, & Michaelraj, P. (2007). Willingness to pay for annual health care services in small ruminants: The case of South India. *Journal of Applied Sciences*, *7*(16), 2361–2365.

Kelly, T. R., Bunn, D. A., Joshi, N. P., Grooms, D., Devkota, D., Devkota, N. R., Mazet, J. A. K. (2018). Awareness and Practices Relating to Zoonotic Diseases among Smallholder Farmers in Nepal. *EcoHealth*, *15*(3), 656–669. https://doi.org/10.1007/s10393-018-1343-4

Kerur, N., Jhala, M. K., & Joshi, C. G. (2008). Genetic characterization of Indian peste des petits ruminants virus (PPRV) by sequencing and phylogenetic analysis of fusion protein and nucleoprotein gene segments. *Research in Veterinary Science*, *85*(1), 176–183. https://doi.org/10.1016/j.rvsc.2007.07.007

Khan, M. H., Manoj, K., & Pramod, S. (2008). Reproductive disorders in dairy cattle under semi-intensive system of rearing in North-Eastern India. *Veterinary World*, *9*(5), 512–518.

Kulangara, V., Joseph, A., Thrithamarassery, N., Sivasailam, A., Kalappurackal, L., Mattappillil, S., Mapranath, S. (2015). Epidemiology of bovine viral diarrhoea among tropical small holder dairy units in Kerala, India. *Tropical Animal Health and Production*, *47*(3), 575–579. https://doi.org/10.1007/s11250-015-0766-y

Kumar, R., Jain, S., Kumar, S. S., Sethi, K., Kumar, S. S., & Tripathi, B. N. (2017). Impact estimation of animal trypanosomosis (surra) on livestock productivity in India using simulation model: Current and future perspective. *Veterinary Parasitology, Regional Studies and Reports*, *10*, 1–12. https://doi.org/10.1016/j.vprsr.2017.06.008

Kumaresan, A., Bujarbaruah, K. M., Pathak, K. A., Chhetri, B., Ahmed, S. K., & Haunshi, S. (2008). Analysis of a village chicken production system and performance of improved dual purpose chickens under a subtropical hill agro-ecosystem in India. *Tropical Animal Health and Production*, *40*(6), 395–402. https://doi.org/10.1007/s11250-007-9097-y

Kumbhakar, N. K., Sanyal, P. K., Rawte, D., Kumar, D., Kerketta, A. E., & Pal, S. (2016). Efficacy of pharmacokinetic interactions between piperonyl butoxide and albendazole against gastrointestinal nematodiasis in goats. *Journal of Helminthology*, *90*(5), 624–629. https://doi.org/10.1017/S0022149X15000930

Kundave, V. R., Patel, A. K., Patel, P. V, Hasnani, J. J., & Joshi, C. G. (2015). Detection of theileriosis in cattle and buffaloes by polymerase chain reaction. *Journal of Parasitic Diseases : Official Organ of the Indian Society for Parasitology*, *39*(3), 508–513. https://doi.org/10.1007/s12639-013-0386-2

Kundave, V. R., Patel, A. K., Patel, P. V., Hasnani, J. J., & Joshi, C. G. (2014). Qualitative and quantitative assessment of Theileria annulata in cattle and buffaloes Polymerase Chain Reaction. *Tropical Biomedicine*, *31*(4), 728–735.

Maan, S., Mor, S. K., Jindal, N., Joshi, V. G., Ravishankar, C., Singh, V. K., Goyal, S. M. (2019). Complete Genome Sequences of Newcastle Disease Virus Isolates from Backyard Chickens in Northern India. *Microbiology Resource Announcements*, *8*(27). https://doi.org/10.1128/MRA.00467-19

Manjeet, Pander, B. L., Sharma, R., Dhaka, S. S., Magotra, A., & Dev, K. (2017). Evaluation of genetic and non-genetic factors on foot and mouth disease (FMD) virus vaccine-elicited immune response in Hardhenu (Bos taurus x Bos indicus) cattle. *Tropical Animal Health and Production*, *49*(8), 1689–1695. https://doi.org/10.1007/s11250-017-1379-4

Meena, H., Ram, H., Sahoo, A., & Rasool, T. (2008). Livestock husbandry scenario at high altitude Kumaon Himalaya. *Indian Journal of Animal Sciences*, *78*(8), 882–886.

Mishra, D., Sahu, R., Mishra, N., & Behera, A. (2015). Herbal treatment for common diseases in ruminants: an overview. *Journal of Livestock Science*, *6*, 36–43.

Mondal, D. B., Sarma, K., & Saravanan, M. (2013). Upcoming of the integrated tick control program of ruminants with special emphasis on livestock farming system in India. *Ticks and Tick-Borne Diseases*, *4*(1–2), 1–10. https://doi.org/10.1016/j.ttbdis.2012.05.006

Murugeswari, R., Valli, C., Karunakaran, R., Leela, V., & Pandian, A. S. S. (2018). Prevalence and magnitude of acidosis sequelae to rice-based feeding regimen followed in Tamil Nadu, India. *Veterinary World*, *11*(4), 464–468. https://doi.org/10.14202/vetworld.2018.464-468

Nazki, S., Wani, S. A., Parveen, R., Ahangar, S. A., Kashoo, Z. A., Hamid, S., Dar, P. A. (2017). Isolation, molecular characterization and prevalence of Clostridium perfringens in sheep and goats of Kashmir Himalayas, India. *Veterinary World*, *10*(12), 1501–1507. https://doi.org/10.14202/vetworld.2017.1501-1507

Osmani, M. G., Ward, M. P., Giasuddin, M., Islam, M. R., & Kalam, A. (2014). The spread of highly pathogenic avian influenza (subtype H5N1) clades in Bangladesh, 2010 and 2011. *Preventive Veterinary Medicine*, *114*(1), 21–27. https://doi.org/10.1016/j.prevetmed.2014.01.010

Panda, T., & Mishra, N. (2016). Indigenous Knowledge on Animal Health Care Practices in Kendrapara District of Odisha, India. *International Letters of Natural Sciences*, *53*, 10–27. https://doi.org/10.18052/www.scipress.com/ILNS.53.10

Pandey, V., Nigam, R., Jaiswal, A. K., Sudan, V., Singh, R. K., & Yadav, P. K. (2015). Haemato-biochemical and oxidative status of buffaloes naturally infected with Trypanosoma evansi. *Veterinary Parasitology*, *212*(3–4), 118–122. https://doi.org/10.1016/j.vetpar.2015.07.025

Patil, M. P., Nagvekar, A. S., Ingole, S. D., Bharucha, S. V., & Palve, V. T. (2015). Somatic cell count and alkaline phosphatase activity in milk for evaluation of mastitis in buffalo. *Veterinary World*, *8*(3), 363–366. https://doi.org/10.14202/vetworld.2015.363-366

Prasad, S., Ramachandran, N., & Raju, S. (2004). Mortality patterns in dairy animals under organized herd management conditions at Karnal India. *Tropical Animal Health and Production*, *36*(7), 645–654. https://doi.org/10.1023/B:TROP.0000042855.58026.bd

Priyadarshini, A., Sarangi, L., Palai, T., Panda, H., Mishra, R., & Behera, P. (2013). Brucellosis in Cattle and Occupationally Exposed Human Beings: A Serosurvey in Odisha, India. *Journal of Pure and Applied Microbiology*, *7*(4), 3255–3260.

Rajkhowa, S., Rajkhowa, C., & Hazarika, G. C. (2006). Prevalence of Cryptosporidium parvum in mithuns (Bos frontalis) from India. *Veterinary Parasitology*, *142*(1–2), 146–149. https://doi.org/10.1016/j.vetpar.2006.06.026

Rajkumar, K., Bhattacharya, A., David, S., Balaji, S. H., Hariharan, R., Jayakumar, M., & Balaji, N. (2016). Socio-demographic study on extent of knowledge, awareness, attitude, and risks of zoonotic diseases among livestock owners in Puducherry region. *Veterinary World*, *9*(9), 1018–1024.

Rajkumar, K., Bhattacharya, A., David, S., Balaji, S. H., Hariharan, R., Jayakumar, M., & Balaji, N. (2016). Socio-demographic study on extent of knowledge, awareness, attitude, and risks of zoonotic diseases among livestock owners in Puducherry region. Veterinary World, 9(9), 1018–1024.

Ranabijuli, S., Mohapatra, J. K., Pandey, L. K., Rout, M., Sanyal, A., Dash, B. B., Pattnaik, B. Serological evidence of foot-and-mouth disease virus infection in randomly surveyed goat population of Orissa, India, 57 Transboundary and Emerging Diseases § (2010). https://doi.org/10.1111/j.1865-1682.2010.01161.x

Rao, K., Rao, K., Rao, S., Ravi, A., & Anitha, A. (2011). Ethnoveterinary practices in sheep of North coastal zone of Andhra Pradesh. Indian Journal of Small Ruminants, 17(2), 252–253.

Sangma, D. B., & Manohara, T. (2018). The role of Garo tribes of Meghalaya (India) in the

conservation and management of medicinal plants diversity used in treating livestock diseases. Plant Science Today, 5(4), 155. https://doi.org/10.14719/pst.2018.5.4.416

Sarma, K., Prasad, H., Das, G., Behera, P., Behera, S. K., Rajesh, J. B., & Borthakur, S. K. (2016). Theileriasis in crossbred cows and its therapeutic management: first report from Lushai hill district of Mizoram. Journal of Parasitic Diseases, 40(3), 605–610. https://doi.org/10.1007/s12639-014-0545-0

Sathiyabarathi, M., Jeyakumar, S., Manimaran, A., Jayaprakash, G., Pushpadass, H. A., Sivaram, M., Kumar, R. D. (2016). Infrared thermography: a potential noninvasive tool to monitor udder health status in dairy cows. Veterinary World, 9(10), 1075–1081. https://doi.org/10.14202/vetworld.2016.1075-1081

Satisha, M. C., Tiwari, R., & Roy, R. (2018). Performance of dairy animals in commercial dairy farms in Karnataka. Indian Journal of Dairy Science, 71(6), 620–624.

Sharma, G. K., Mahajan, S., Matura, R., Biswal, J. K., Ranjan, R., Subramaniam, S., Pattnaik, B. (2017). Herd Immunity Against Foot-and-Mouth Disease Under Different Vaccination Practices in India. Transboundary and Emerging Diseases, 64(4), 1133–1147. https://doi.org/10.1111/tbed.12478

Sharma, K. K., Kshirsagar, D. P., Kalyani, I. H., Patel, D. R., Vihol, P. D., & Patel, J. M. (2015). Diagnosis of peste des petits ruminants infection in small ruminants through in-house developed Indirect ELISA: Practical considerations. Veterinary World, 8(4), 443–448. https://doi.org/10.14202/vetworld.2015.443-448

Sherikar, A., & Waskar, V. (2005). Emerging zoonoses and social-economic impact in India - A review. Indian Journal of Animal Sciences, 76(6), 700–705.

Shrimali, R. G., Patel, M. D., & Patel, R. M. (2016). Comparative efficacy of anthelmintics and their effects on hemato-biochemical changes in fasciolosis of goats of South Gujarat. Veterinary World, 9(5), 524–529. https://doi.org/10.14202/vetworld.2016.524-529

Shyma, K. P., Gupta, J. P., & Singh, V. (2015). Breeding strategies for tick resistance in tropical cattle: a sustainable approach for tick control. Journal of Parasitic Diseases, 39(1), 1–6. https://doi.org/10.1007/s12639-013-0294-5

Singh, B. B. B., Dhand, N. K. K., & Gill, J. P. S. P. S. (2015). Economic losses occurring due to brucellosis in Indian livestock populations. Preventive Veterinary Medicine, 119(3–4), 211–215. https://doi.org/10.1016/j.prevetmed.2015.03.013

Singh, B. B., Kaur, R., Gill, G. S., Gill, J. P. S., Soni, R. K., & Aulakh, R. S. (2019). Knowledge, attitude and practices relating to zoonotic diseases among livestock farmers in Punjab, India. Acta Tropica, 189, 15–21. https://doi.org/10.1016/j.actatropica.2018.09.021

Singh, K., Chandel, B. S., Chauhan, H. C., Dadawala, A., Singh, S. V, & Singh, P. K. (2013). Efficacy of "indigenous vaccine" using native "Indian bison type" genotype of Mycobacterium avium subspecies paratuberculosis for the control of clinical Johne's disease in an organized goat herd. Veterinary Research Communications, 37(2), 109–114. https://doi.org/10.1007/s11259-013-9551-4

Singh, M., Dixit, A., Roy, A., & Singh, S. (2014). Analysis of prospects and problems of goat production in Bundelkhand region. Range Management and Agroforestry, 35(1), 163–168.

Singh, S. V., Singh, A. V., Singh, R., Sharma, S., Shukla, N., Misra, S., Sandhu, K. S. (2008). Sero-prevalence of Bovine Johne's disease in buffaloes and cattle population of North India using indigenous ELISA kit based on native Mycobacterium avium subspecies paratuberculosis 'Bison type' genotype of goat origin. Comparative Immunology, Microbiology and Infectious Diseases, 31(5), 419–433. https://doi.org/10.1016/j.cimid.2007.06.002

Sudan, V., Jaiswal, A. K., Parashar, R., & Shanker, D. (2015). A duplex PCR-based assay for simultaneous detection of Trypanosoma evansi and Theileria annulata infections in water buffaloes. Tropical Animal Health and Production, 47(5), 915–919. https://doi.org/10.1007/s11250-015-0808-5

Sudan, V., Jaiswal, A. K., & Shanker, D. (2014). Infection rates of Linguatula serrata nymphs in mesenteric lymph nodes from water buffaloes in North India. Veterinary Parasitology, 205(1–2), 408–411. https://doi.org/10.1016/j.vetpar.2014.07.025

Sudan, V., Shanker, D., Jaiswal, A., Singh, A., & Pandey, V. (2017). Standardization and validation of simple PCR, duplex PCR and RAPD in comparison to blood smear examination for diagnosing bovine tropical theileriosis. Biologicals : Journal of the International Association of Biological Standardization, 46, 88–91. https://doi.org/10.1016/j.biologicals.2017.01.003

Sudan, V., Singh, S. K., Jaiswal, A. K., Parashar, R., & Shanker, D. (2015). First molecular evidence of the transplacental transmission of Theileria annulata. Tropical Animal Health and Production, 47(6), 1213–1215. https://doi.org/10.1007/s11250-015-0835-2

Taku, A., Chhabra, R., & War, B. A. (2010). Footrot on a sheep breeding farm in the Himalayan state of Jammu and Kashmir. Revue Scientifique et Technique (International Office of Epizootics), 29(3), 671–675.

Thakur, R., Thakur, D., & Dogra, P. (2018). Indigenous feeding practices for better reproductive performance in buffaloes in Himachal Pradesh, India. Buffalo Bulletin, 37(3), 361–368.

Tripathi, S. M., Thaker, A. M., Joshi, C. G., & Sankhala, L. N. (2012). Acephate immunotoxicity in White Leghorn cockerel chicks upon experimental exposure. Environmental Toxicology and Pharmacology, 34(2), 192–199. https://doi.org/10.1016/j.etap.2012.04.002

Vijayasarathi, M. K., Sreekumar, C., Venkataramanan, R., & Raman, M. (2016). Influence of sustained deworming pressure on the anthelmintic resistance status in strongyles of sheep under field conditions. Tropical Animal Health and Production, 48(7), 1455–1462. https://doi.org/10.1007/s11250-016-1117-3

Whittington, R., Donat, K., Weber, M. F., Kelton, D., Nielsen, S. S., Eisenberg, S., Waard, J. H. (2019). Control of paratuberculosis: who, why and how. A review of 48 countries. BMC Veterinary Research, 15(1), 198. https://doi.org/10.1186/s12917-019-1943-4

Wright, A., & Thrusfield, M. (2016). Perceptions of zoonotic and animal diseases in the Van Gujjar community of North India. Preventive Veterinary Medicine, 123, 143–153. https://doi.org/10.1016/j.prevetmed.2015.11.012

Yadav, A., Panadero, R., Katoch, R., Godara, R., & Cabanelas, E. (2017). Myiasis of domestic and wild ruminants caused by Hypodermatinae in the Mediterranean and Indian subcontinent. Veterinary Parasitology, 243, 208–218. https://doi.org/10.1016/j.vetpar.2017.07.007

Yang, Z., Xu, G., Reboud, J., Ali, S. A., Kaur, G., McGiven, J., Cooper, J. M. (2018). Rapid Veterinary Diagnosis of Bovine Reproductive Infectious Diseases from Semen Using Paper-Origami DNA Microfluidics. ACS Sensors, 3(2), 403–409. https://doi.org/10.1021/acssensors.7b00825

Zahid, U. N., Randhawa, S. S., Hussain, S. A., Randhawa, S. S., Mahajan, V., & Dua, K. (2014). Claw lesions causing clinical lameness in lactating holstein frisian crossbred cows. Veterinary Medicine International, 2014, 764689. https://doi.org/10.1155/2014/764689
