# Supplementary material for: Prioritizing smallholder animal health needs in East Africa, West Africa, and South Asia using three approaches: Literature review, expert workshops, and practitioner surveys
Source: Prev Vet Med. 2021 Apr;189:105279. doi: 10.1016/j.prevetmed.2021.105279 (PMC8024747; doi:10.1016/j.prevetmed.2021.105279)
Supplement: Supplementary file 12 [file mmc12.pdf]

Smallholder Animal Health Needs Assessment
South Asia Small Ruminants

GALVmed

August 19, 2019

Assembled by Zoë Campbell, Peetambar Kushwaha, and Paul Coleman

Meta-analysis summary

The literature review yielded 35 articles, 24 of which focused on impact, incidence, and prevalence, and are summarized below. The table below shows the number of articles in which each animal health concern appears in the literature about sheep and goats (small ruminants) in South Asia. Many articles mention multiple concerns. The summarized articles are in the bibliography within this document. The full list of articles considered in the South Asia meta-analysis is available as a separate document.

The most mentioned concerns in the meta-analysis are peste des petits ruminants (PPR), endoparasites, helminths, and foot and mouth disease (mentioned in 7+ articles). Foot and mouth disease is the most mentioned concern in the impact articles, followed by PPR, endoparasites, diarrhea, goat pox, pneumonia, and bloat. Three articles were assigned a green ranking for focus on both incidence/ prevalence and impact. These articles focused on PPR, brucellosis, and trypanosomiasis.

Animal health concern	Frequency of mention (meta-analysis)	Frequency of mention (impact articles)	Main topic of green rank article
PPR	10	6	1
Endoparasites	7	4	0
Helminth	7	3	0
FMD	7	7	0
Diarrhea	5	4	0
Goat pox	4	4	0
Sheep pox	4	3	0
Pneumonia	4	4	0
Bloat	4	4	0
Brucellosis	4	3	1

Abortion	3	3	0
Respiratory disease	3	3	0
Arthritis	2	2	0
Fascioliasis	2	1	0
Foot conditions	2	2	0
Trypanosomiasis	2	2	1
Anthelmintic resistance	1	1	0
CCPP	1	1	0
Clostridium perfringens	1	1	0
Johne's disease	1	0	0

Table of articles

Abbreviations:

PPR – Peste des petits ruminants

FMD – Foot and mouth disease

HS – Haemorrhagic septicaemia

BQ – Black quarter

CCPP - Contagious caprine pleuropneumonia

Note: Clicking on the article number links to an expanded summary of the article below. The “web” link connects to the abstract of the article online or to full text for open access articles.

#	Citation	Country	Study design	Pathogen/ disease	Findings
1 2 web	(G. N. Govindaraj et al., 2019)	India	Economic analysis of vaccination, Multiple production systems	PPR	**Impact:** The estimated mean mortality loss was USD 45.2 and USD 16.5 per animal in goats and sheep, respectively, whereas the treatment and opportunity cost of labour was USD 1.9 and USD 2.5 per animal respectively. Under the low PPR incidence scenario, benefit: cost ratio, net present value and internal rate of return were 4.9:1, 48.9 million USD and 146.6%, whereas it was 12.4: 1,142.7 million USD and 430.4% and

					13.5: 1,156.7 million USD and 430.4% under medium and high incidence scenarios.
2<br>1<br>web	(Hegde et al., 2009)	India	Retrospective quantitative analysis of epidemiological data on peste des petits ruminants (PPR) as reported to the state from 1998-2007	PPR	**Incidence/ prevalence:** 624 outbreaks reported in 9 years.<br>**Impact:** 19,440 animals affected and 2,697 animals died. Mortality rate estimated as 13.8%.
3<br>2<br>web	(Balamurugan et al., 2014)	India	Cross-sectional serosurvey,<br>391 serum samples from goats (318 random and 73 outbreak/suspected) were collected from 28 districts in 7 states (Meghalaya, Assam, Manipur, Nagaland, Arunachal Pradesh, Tripura and Mizoram)	PPR	**Incidence/ prevalence**: Analysis of 318 serum samples indicates an overall seroprevalence of 11.63 % in random [CI 95 % 8.56–15.63] samples.
4<br>3	(Kamal, Mondal, Islam, & Islam, 2012)	Bangladesh	Cross-sectional, Questionnaire,<br>150 smallholders	PPR<br>Goat pox<br>FMD	**Incidence/ prevalence:** 40% of respondents listed PPR as the most common disease, 29% listed goat pox, and 22% listed FMD.

5<br>2<br>web	(Vijayasarathi, Sreekumar, Venkataramanan, & Raman, 2016)	India	Randomized control trial,<br>20 flocks of sheep,<br>Smallholders	Anthelmintic resistance	Internal parasite of sheep developed resistance to Fenbendazole while they were found less resistant to Tetramisole and Ivermectin.<br>**Impact:** Routine and regular use of fenbendazole for drenching caused sheep internal parasite to develop resistance.
6<br>2<br>web	(Ranabijuli et al., 2010)	India	Cross-sectional serosurvey,<br>544 goats in 12 villages in 6 districts,<br>Smallholders	FMD	**Incidence/ prevalence:** Serological investigations were carried out to generate estimates of antibody prevalence in goats of Orissa state to both non-structural (NSP-Ab) and structural proteins (SP-Ab) of FMD. The apparent overall NSP-Ab and SP-Ab seroprevalences were 38% and 20.7%, respectively, which signifies a very high level of FMD virus circulation in the goat population despite the lack of clinical signs in this species.
7<br>3<br>web	(Mishra et al., 2015)	India	Literature review of herbal treatments commonly used by smallholders in India for treatment of cattle and small ruminants	FMD<br>Haemorrhagic septicaemia (HS),<br>Black quarter (BQ),<br>Endoparasites,<br>Helminths,<br>Anthrax,<br>Actinobacillosis,<br>Ringworm	**Perceptions:** 14 plant species are described for treatment of FMD, 9 plant species each for treatment of HS and BQ, 5 plant species for round worm infestation, 4 plant species for anthrax, 3 plant species each for actinobacillosis and ringworm infection, and 1 plant species for fluke infestation.

8<br>3	(Meena et al., 2008)	India	Cross-sectional questionnaire, 300 smallholders dairy farmers, Knowledge, attitudes, practices about cattle and small ruminants	Diarrhoea, Respiratory disease, Haemorrhagic septicaemia, FMD, Sheep and goat pox, PPR, Reproductive disorders	**Impact:** The principal causes of livestock mortality as reported by farmers were infectious diseases like diarrhoea, pneumonia, haemorrhagic septicaemia (HS), foot and mouth disease, pox, PPR (30% of mortalities). Reproductive disorders such as difficulty in parturition, abortions, retained placenta, metritis made up 25% of mortalities.<br>**Perceptions:** Local remedies used for common diseases including diarrhoea, common cold, pneumonia, and bloat. Only 27% of farmers knew about vaccination against diseases like HS, black quarter, FMD, PPR, and pox.
9<br>2<br>web	(Hota et al., 2018)	India	Cross-sectional serosurvey of 214 sheep and 286 goats across 10 agro-climatic zones of Odisha	Sheep and goat pox	**Incidence/ prevalence:** Screening of 500 serum samples showed seropositivity of 8.88% and 31.47% in sheep and goats, respectively, for *Capripoxviruses.*
10<br>3<br>web	(Bhanuprakash, Hosamani, & Singh, 2011)	India	Perspective article on sheep and goat pox in India	Sheep and goat pox	**Incidence/ prevalence:** The estimated morbidity and mortality in Maharastra state are 63% and 50%, respectively.<br>**Impact:** By extrapolating from estimated losses in Maharastra state, the estimated annual losses nationally are $27 million USD.

11<br>3<br>web	(M. Singh, Dixit, Roy, & Singh, 2014)	India	Cross-sectional, Questionnaire, 194 households in 16 villages owning goats, Smallholders	Pneumonia<br>Diarrhoea<br>Endoparasites<br>PPR<br>FMD<br>Abortion	**Impact:** The annual mortality in kids (up to 6 months) and adult goats was 17.4 and 13.6%, respectively.<br>**Perceptions:** Pneumonia, diarrhoea, endoparasitic diseases, PPR, FMD, and abortions were major health problems.
12<br>1<br>web	(B. B. B. Singh et al., 2015)	India	Economic model using official records and epidemiological surveys, Multiple production systems, Cattle, buffalo, small ruminants	Brucellosis	**Impact:** The analysis revealed that brucellosis in livestock is responsible for a median loss of US $ 3.4 billion (5th–95th percentile 2.8–4.2 billion). The disease in cattle and buffalo accounted for 95.6% of the total losses occurring due to brucellosis in livestock populations. The disease is responsible for a loss of US $ 0.7 per sheep and US $ 0.5 per goat.
13<br>2<br>web	(Kanani et al., 2018)	India	Cross-sectional serosurvey, 2,444 sera samples (675 sheep and 1769 goat) from unorganized sector and 1310 sera samples (861 sheep and 449 goat) from seven organized farms	Brucellosis	**Incidence/ prevalence:** In the unorganized sector, 24% of sheep (160/675) and 16% of goats (283/1769) were positive by Rose Bengal Plate Test (RBPT) and 24% of sheep (165/675) and 17% of goats (305/1769) were positive by iELISA. The organized sector samples showed higher seroprevalence in goats (8 %, 35/449) than sheep (4 %, 35/861) by RBPT. Similarly, in iELISA, goat samples showed a higher seroprevalence (9%, 42/449) compared to sheep (8 %, 65/861).

14<br>2<br>web	(Islam et al., 2013)	Bangladesh	Meta-analyses of published papers on brucellosis in cattle, buffalo, sheep and goats	Brucellosis	**Incidence/Prevalence:** Seroprevalences were estimated as 3.6% for goats and 7.3% for sheep.
15<br>1<br>web	(Kumar et al., 2017)	India	Risk and retrospective analyses to estimate economic loss	Trypanosomosis	**Incidence/ prevalence:** Cites Krishnappa et al. (2002), reporting 2.15% prevalence of trypanosomosis in goats based on data obtained from 23 districts of Karnataka.<br>**Impact:** A total annual loss (direct and indirect) caused by animal trypanosomosis was estimated to US $ 671.1 million (US $ 344–US $ 1209 million at 95% confidence interval), at present valuation. The mortality losses were estimated to 16% of the total loss. Among morbidity losses, the reduction in milk yield and reproductive losses components were 36% and 26% of total loss, respectively. Other parameters like reduction in growth (10%), reduction in draught power (8%) and additional opportunity cost (3%) also yielded considerable loss.
16<br>3<br>web	(Mondal et al., 2013)	India	Literature review	Tick-borne diseases, Acaricide resistance	**Impact:** A recent estimate calculated the costs of control of tick-borne diseases affecting Indian livestock as 498.7 million US $ per annum (Minjauw and McLeod, 2003). As per leather industry report, the leather sector of India is suffering from a huge

					shortfall of 3 billion pieces of hide and skin per year and causing 20–30% depreciation in normal value in the market. It has been estimated that India produces only 9.8%, 63.3%, 9.2%, and 6.0% of world cattle, buffalo, goat, and sheep hides, respectively, although the country possesses the highest livestock population.
17 3	(Kathiravan et al., 2005)	India	Cross-sectional, 320 respondents seeking services from public veterinary centres, Multiple production systems	Cost of animal health services	**Impact:** The mean cost of treatment of a chronic medical case in cattle at a public veterinary centre was INR 20.83, in which the labour cost alone accounted to INR 17.35, with the remaining amount for the drugs purchased outside. However, the mean costs of treating a chronic medical condition in buffalo and small ruminant at public veterinary centres were only INR 13.34 and INR 10.80, respectively. Although no charges were made for animal health care services rendered at public veterinary centres, the charges in terms of imputed labour cost for bringing the animal to the centre was incurred. Service fee accounted for more than 60 per cent of cost of treatment for home service by a veterinarian or a para-veterinarian.

18<br>3<br>web	(Rao, Rao, Rao, Ravi, & Anitha, 2011)	India	Cross-sectional, Ethnoveterinary, Interview with 960 sheep farmers in migratory and semi-migratory production systems in 3 districts	Multiple	**Perceptions:** Reported health concerns of sheep with ethnoveterinary treatments/ preventions included burns, dehydration, shock, pyrexia, haemorrhage, inflammation, scorpion bites, colic, respiratory problems, and arthritis. Sheep farmers perceived their treatments to be effective 20-80% of the time.
19<br>3<br>web	(Bhatt et al., 2019)	India	Cross-sectional, Ethnoveterinary, Semi-structured questionnaire with 82 male livestock owners	Gastrointestinal problems, Multiple	**Perceptions:** A total of 13 medicinal plants were cited 47 times for gastrointestinal problems. Other animal health concerns treated with ethnoveterinary medicine included retention foetal membrane, enteritis, cough and respiratory disease, wounds, skin problems, ringworm, foot conditions, vaginal and uterine prolapses, and bloat.
20<br>3<br>web	(Panda & Mishra, 2016)	India	Cross-sectional, Qualitative methods including participant observation and interview, Ethnoveterinary	Multiple	**Perceptions:** The primary ailments of livestock are wound, diarrhoea, injury, fever, gastrointestinal problems and gynaecological problems, which are commonly treated with medicinal plants.
21<br>3<br>web	(Sangma & Manohara, 2018)	India	Cross-sectional, Qualitative methods including group discussion and interview,	Multiple	**Perceptions:** Animal health concerns with the highest consensus of appropriate ethnoveterinary treatment were arthritis, rabies, reproductive diseases, retained placenta, and swollen body parts. Other

			Ethnoveterinary		health concerns commonly treated include diarrhoea, wounds, and ectoparasites.
22<br>3<br>web	(Taku, Chhabra, & War, 2010)	India	Outbreak investigation, Confirmatory diagnosis of clinical case by culture and PCR	Footrot in sheep	Footrot case confirmed in Jammu and Kashmir.
23<br>2<br>web	(Iqbal Yatoo et al., 2019)	Multiple	Literature review	CCPP	**Incidence/ prevalence:** In naive and native herds, 100% morbidity and 80% mortality have been noted. Cites a reported seroprevalence of about 10% in Pashmina goats from Jammu and Kashmir State, India. **Impact:** It is estimated that the total yearly cost of CCPP is about US$507 million in endemic areas thus involving major economic losses.
24<br>3<br>web	(Nazki et al., 2017)	India	*C. perfringens* toxinotype screening amongst purposively chosen small ruminants (152 sheep and 25 goats)	*Clostridium perfringens*	**Incidence/ prevalence:** The toxinotypes A and D are prevalent in both sheep and goats. The highest prevalence of C. perfringens toxinotype D was observed in lambs (56.16%) and kids (46.16%) followed by 3.84% in adult sheep while it was absent in samples obtained from adult goats. This is NOT a representative survey as sample was purposive and included healthy and diarrheic

					animals, and morbid material of animals suspected to have died of enterotoxaemia.

Article summaries

Govindaraj, G. N., Roy, G., Mohanty, B. S., Balamurugan, V., Pandey, A. K., Sharma, V., Roy, P. (2019). Evaluation of effectiveness of Mass Vaccination Campaign against Peste des petits ruminants in Chhattisgarh state, India. *Transboundary and Emerging Diseases*, *66*(3), 1349–1359. https://doi.org/10.1111/tbed.13163

This study estimates the economic costs and benefits of mass PPR vaccination campaigns for sheep and goats in Chhattisgarh state, India.

- The estimated mean mortality loss was USD 45.2 and USD 16.5 per animal in goats and sheep, respectively, whereas the treatment and opportunity cost of labour was USD 1.9 and USD 2.5 per animal respectively.
- Under the low PPR incidence scenario, benefit: cost ratio, net present value and internal rate of return were 4.9:1, 48.9 million USD and 146.6%, whereas it was 12.4: 1,142.7 million USD and 430.4% and 13.5: 1,156.7 million USD and 430.4% under medium and high incidence scenarios.

Hegde, R., Gomes, A. R., Muniyellappa, H. K., Byregowda, S. M., Giridhar, P., & Renukaprasad, C. (2009). A short note on peste des petits ruminants in Karnataka, India. *Revue Scientifique et Technique (International Office of Epizootics)*, *28*(3), 1031–1035.

Retrospective quantitative analysis of epidemiological data on peste des petits ruminants (PPR) as reported to the government in Karnataka, India from 1998-2007.

- From 1998-2007, 624 outbreaks were reported.
- 19,440 animals were affected and 2,697 animals died. Mortality rate was estimated as 13.8%.
- Incidences were highest during the rainy season and in the dry agro-climatic zones. The density of the PPR-susceptible population in different districts of the state played a major role in disease incidences.

Balamurugan, V., Das, S., Raju, D. S. N., Chakravarty, I., Nagalingam, M., Hemadri, D., Rahman, H. (2014). Prevalence of peste des petits ruminants in goats in North-East India. *VirusDisease*, *25*(4), 488–492. https://doi.org/10.1007/s13337-014-0237-5

Cross-sectional serosurvey for peste des petits ruminants. Total of 391 serum samples from goats (318 random and 73 outbreak/suspected) were collected from 28 districts in 7 states (Meghalaya, Assam, Manipur, Nagaland, Arunachal Pradesh, Tripura and Mizoram).

- Analysis of 318 serum samples indicates an overall seroprevalence of 11.63% in random [CI 95 % 8.56–15.63] samples.

Kamal, M., Mondal, S., Islam, S., & Islam, M. (2012). Present status of goat rearing under rural conditions in south-west regions of Bangladesh. In G. Rahmann & D. Godinho (Eds.), *Organic Animal Husbandry Challenges*. Hamburg/ Trenthorst, Germany.

Questionnaire administered to 150 smallholders raising goats in south-west Bangladesh to characterize production system. No known web access to article.

- 40% of respondents listed PPR as the most common disease affecting goats, 29% listed goat pox, and 22% listed FMD.

Vijayasarathi, M. K., Sreekumar, C., Venkataramanan, R., & Raman, M. (2016). Influence of sustained deworming pressure on the anthelmintic resistance status in strongyles of sheep under field conditions. *Tropical Animal Health and Production*, *48*(7), 1455–1462. https://doi.org/10.1007/s11250-016-1117-3

This randomized control trial examined anthelmintic resistance status in strongyles of 15 herds of sheep from households that benefitted from a government initiative allowing access to deworming four times a year for the past 10 years compared to 5 herds of sheep owned by households not covered by the initiative.

- Internal parasites of sheep developed resistance to Fenbendazole while they were found less resistant to Tetramisole and Ivermectin.
- Routine and regular use of fenbendazole for drenching caused internal parasites to develop resistance.

Ranabijuli, S., Mohapatra, J. K., Pandey, L. K., Rout, M., Sanyal, A., Dash, B. B., Pattnaik, B. Serological evidence of foot-and-mouth disease virus infection in randomly surveyed goat population of Orissa, India, 57 Transboundary and Emerging Diseases (2010). https://doi.org/10.1111/j.1865-1682.2010.01161.x

A cross-sectional serosurvey of 544 goats owned by smallholders in 12 villages across six districts in Orissa state, India to generate estimates of antibody prevalence in goats to both non-structural (NSP-Ab) and structural proteins (SP-Ab) of foot and mouth disease.

- The apparent overall NSP-Ab and SP-Ab seroprevalences were 38% and 20.7%, respectively, which signifies a very high level of FMD virus circulation in the goat population despite the lack of clinical signs in this species.
- The results underscore the requirement to bring caprine species under comprehensive surveillance and vaccination campaigns to check silent amplification, excretion and transmission of the virus.

Mishra, D., Sahu, R., Mishra, N., & Behera, A. (2015). Herbal treatment for common diseases in ruminants: an overview. *Journal of Livestock Science*, *6*, 36–43.

Literature review of herbal treatments commonly used by smallholders in India for treatment of cattle and small ruminants.

- Fourteen plant species are described for treatment of FMD, 9 plant species each for treatment of HS and BQ, 5 plant species for round worm infestation, 4 plant species for anthrax, 3 plant species each for actinobacillosis and ringworm infection, and 1 plant species for fluke infestation.
- The description of herbal treatments gives an indication of livestock diseases that affect smallholder farmers in India.

Meena, H., Ram, H., Sahoo, A., & Rasool, T. (2008). Livestock husbandry scenario at high altitude Kumaon Himalaya. *Indian Journal of Animal Sciences*, *78*(8), 882–886.

Cross-sectional questionnaire administered to 300 smallholders dairy farmers focusing on knowledge, attitudes, and practices for livestock husbandry of cattle and small ruminants. Some lack of clarity about when primary versus secondary data are used. There is no known web access to the article.

- The principal causes of livestock mortality as reported by farmers were infectious diseases like diarrhoea, pneumonia, haemorrhagic septicaemia (HS), foot and mouth disease, pox, PPR (30% of mortalities).
- Reproductive disorders such as difficulty in parturition, abortions, and retained placenta made up 25% of mortalities.
- Local remedies used for common diseases including diarrhoea, common cold, pneumonia, and bloat.
- Only 27% of farmers knew about vaccination against diseases like HS, black quarter, FMD, PPR, and pox.

Hota, A., Biswal, S., Sahoo, N., Venkatesan, G., Arya, S., Kumar, A., Rout, M. (2018). Seroprevalence of Capripoxvirus infection in sheep and goats among different agro-climatic zones of Odisha, India. *Veterinary World*, *11*(1), 66–70. https://doi.org/10.14202/vetworld.2018.66-70

Cross-sectional serosurvey of 214 sheep and 286 goats across 10 agro-climatic zones of Odisha, India. Serosamples (n=500) were screened using whole virus antigen-based indirect ELISA for antibodies against *Capripoxviruses*. Production system was not specified.

- Screening of 500 serum samples showed seropositivity of 8.88% and 31.47% in sheep and goats, respectively, for Capripoxviruses.
- The prevalence rate according to agro-climatic zone ranged from 0% (North Eastern coastal plain zone) to 48.57% (North central plateau zone) for goat pox, and 0%

(Western undulating zone and North central plateau) to 22.22% (South Eastern ghat zone) for sheep pox. The difference in prevalence rates among the various agro-climatic zones was statistically significant ($p<0.05$) for goats, but not for sheep.

- Antibody prevalence rates among various districts were recorded to be the highest in Jagatsinghpur (30%) for sheep pox and Dhenkanal (80%) for goat pox.

Bhanuprakash, V., Hosamani, M., & Singh, R. K. (2011). Prospects of control and eradication of capripox from the Indian subcontinent: A perspective. *Antiviral Research*, *91*(3), 225–232. https://doi.org/10.1016/j.antiviral.2011.06.004

This perspective article summarizes selected literature on goat pox in India. The majority of studies focus on Maharastra state.

- The estimated morbidity and mortality in Maharastra state are 63% and 50%, respectively.
- By extrapolating from estimated losses in Maharastra state, the estimated annual losses nationally are $27 million USD.

Singh, M., Dixit, A., Roy, A., & Singh, S. (2014). Analysis of prospects and problems of goat production in Bundelkhand region. *Range Management and Agroforestry*, *35*(1), 163–168.

Questionnaire administered to 194 smallholder households owning goats in 16 villages in Bundelkhand, India to learn about prospects and problems of goat production.

- The annual mortality in kids (up to 6 months) and adult goats was 17.4 and 13.6%, respectively.
- Pneumonia, diarrhoea, endoparasitic diseases, PPR, FMD, and abortions were major health problems.

Singh, B. B. B., Dhand, N. K. K., & Gill, J. P. S. P. S. (2015). Economic losses occurring due to brucellosis in Indian livestock populations. *Preventive Veterinary Medicine*, *119*(3–4), 211–215. https://doi.org/10.1016/j.prevetmed.2015.03.013

Economic model used to estimate the losses due to brucellosis in Indian livestock including cattle, buffalo, sheep, and goats. Data sources included prevalence data from epidemiological surveys conducted in India and data for livestock populations from official records. Probability distributions were used for many of the input parameters to account for uncertainty and variability.

- The analysis revealed that brucellosis in livestock is responsible for a median loss of US $ 3.4 billion (5th–95th percentile 2.8–4.2 billion).

- The disease in cattle and buffalo accounted for 95.6% of the total losses occurring due to brucellosis in livestock populations.
- The disease is responsible for a loss of US $ 0.7 per sheep and US $ 0.5 per goat.

Kanani, A., Dabhi, S., Patel, Y., Chandra, V., Kumar, O. R. V., & Shome, R. (2018). Seroprevalence of brucellosis in small ruminants in organized and unorganized sectors of Gujarat state, India. *Veterinary World*, *11*(8), 1030–1036. https://doi.org/10.14202/vetworld.2018.1030-1036

Cross-sectional serosurvey to screen for brucellosis using 2,444 sera samples (675 sheep and 1769 goat) from unorganized sector and 1,310 sera samples (861 sheep and 449 goat) from seven organized farms in Gujarat state, India.

- In the unorganized sector, 24% of sheep (160/675) and 16% of goats (283/1769) were positive by Rose Bengal Plate Test (RBPT) and 24% of sheep (165/675) and 17% of goats (305/1769) were positive by iELISA.
- The organized sector samples showed higher seroprevalence in goats (8 %, 35/449) than sheep (4 %, 35/861) by RBPT. Similarly, in iELISA, goat samples showed a higher seroprevalence (9%, 42/449) compared to sheep (8 %, 65/861).
- The diagnostic sensitivity and specificity of RBPT with ELISA were 88.69% and 99.65%, respectively, and showed a significant difference (p≤0.0001).

Islam, M. A., Khatun, M. M., Werre, S. R., Sriranganathan, N., & Boyle, S. M. (2013). A review of Brucella seroprevalence among humans and animals in Bangladesh with special emphasis on epidemiology, risk factors and control opportunities. *Veterinary Microbiology*, *166*(3–4), 317–326. https://doi.org/10.1016/j.vetmic.2013.06.014

Meta-analyses of published papers on brucellosis in cattle, buffalo, sheep and goats in Bangladesh.

- Seroprevalences were estimated as 3.6% for goats and 7.3% for sheep.
- Different tests, of varying sensitivity and specificity, have been used in different studies. As a result, the meta-analysis just gives an indication of prevalence rates.

Kumar, R., Jain, S., Kumar, S. S., Sethi, K., Kumar, S. S., & Tripathi, B. N. (2017). Impact estimation of animal trypanosomosis (surra) on livestock productivity in India using simulation model: Current and future perspective. *Veterinary Parasitology, Regional Studies and Reports*, *10*, 1–12. https://doi.org/10.1016/j.vprsr.2017.06.008

The economic losses on livestock productivity were assessed resulting from animal trypanosomosis (surra) in India, considering all possible direct and indirect losses in major six livestock species: cattle, buffalo, goat, equine, camel and pig. The contemplative risk and

retrospective analyses were performed using various official records and scientific literature complemented with expert data for evaluation of impact of surra on livestock productivity in India. Most of the information were derived using the secondary data published in scientific journals, and the official data reported by Basic Animal Husbandry and Fisheries Statistics, the Government of India, and other scientific reports.

- Very few reports of occurrence of natural trypanosomosis in goat are available from different parts of the country. Krishnappa et al. (2002) reported 2.15% prevalence of trypanosomosis in goat based on data obtained from 23 districts of Karnataka.
- A total annual loss (direct and indirect) caused by animal trypanosomosis was estimated to US $ 671.1 million (US $ 344–US $ 1209 million at 95% confidence interval), at present valuation.
- The mortality losses were estimated to 16% of the total loss. Among morbidity losses, the reduction in milk yield and reproductive losses components were 36% and 26% of total loss, respectively. Other parameters like reduction in growth (10%), reduction in draught power (8%) and additional opportunity cost (3%) also yielded considerable loss.

Mondal, D. B., Sarma, K., & Saravanan, M. (2013). Upcoming of the integrated tick control program of ruminants with special emphasis on livestock farming system in India. *Ticks and Tick-Borne Diseases*, *4*(1–2), 1–10. https://doi.org/10.1016/j.ttbdis.2012.05.006

A literature review of tick-borne disease and tick control for livestock in India.

- A recent estimate calculated the costs of control of tick-borne diseases affecting Indian livestock as 498.7 million US $ per annum (Minjauw and McLeod, 2003).
- As per leather industry report, the leather sector of India is suffering from a huge shortfall of 3 billion pieces of hide and skin per year and causing 20–30% depreciation in normal value in the market. It has been estimated that India produces only 9.8%, 63.3%, 9.2%, and 6.0% of world cattle, buffalo, goat, and sheep hides, respectively, although the country possesses the highest livestock population.

Kathiravan, G., Thirunavukkarasu, M., & Selvakumar, K. N. (2005). Cost of livestock services: The case of Tamil Nadu (India). *Journal of Applied Sciences Research*, (No.October), 1195–1205.

Cross-sectional survey of 320 respondents selected through multi-stage sampling procedure seeking services from public veterinary centres to learn about the prices paid for animal health services. The estimated exchange rate from June 15, 2007 is 40.6 INR to $1 USD. No known web access to article.

- The mean cost of treatment of a chronic medical case in cattle at a public veterinary centre was INR 20.83, in which the labour cost alone accounted to INR 17.35, with the

remaining amount for the drugs purchased outside. However, the mean costs of treating a chronic medical condition in buffalo and small ruminant at public veterinary centres were only INR 13.34 and INR 10.80, respectively.

- Although no charges were made for animal health care services rendered at public veterinary centres, the charges in terms of imputed labour cost for bringing the animal to the centre was incurred.
- Service fee accounted for more than 60 per cent of cost of treatment for home service by a veterinarian or a para-veterinarian.

Rao, K., Rao, K., Rao, S., Ravi, A., & Anitha, A. (2011). Ethnoveterinary practices in sheep of North coastal zone of Andhra Pradesh. *Indian Journal of Small Ruminants*, *17*(2), 252–253.

Cross-sectional study of ethnoveterinary practices involving interviews with 960 sheep farmers in migratory and semi-migratory production systems in 3 districts in Andhra Pradesh, India.

- Reported health concerns of sheep with ethnoveterinary treatments/ preventions included burns, dehydration, shock, pyrexia, haemorrhage, inflammation, scorpion bites, colic, respiratory problems, and arthritis. Sheep farmers perceived their treatments to be effective 20-80% of the time.

Bhatt, P. R., B. Pandya, K., Patel, U. D., Patel, H. B., & Modi, C. M. (2019). Survey on Ethnoveterinary Practices around Junagadh, Gujarat, India. *Indian Journal of Pharmaceutical Sciences*, *81*(1). https://doi.org/10.4172/pharmaceutical-sciences.1000493

Cross-sectional study of ethnoveterinary practices around Junagadh, Gujarat, India. A semi-structured questionnaire was administered to 82 male livestock owners. Study mentions cattle, buffalo, sheep, and goats are commonly kept but does not match animal health concerns to species.

- A total of 13 medicinal plants were cited 47 times for gastrointestinal problems.
- Other animal health concerns treated with ethnoveterinary medicine included retention foetal membrane, enteritis, cough and respiratory disease, wounds, skin problems, ringworm, foot conditions, vaginal and uterine prolapses, and bloat.

Panda, T., & Mishra, N. (2016). Indigenous Knowledge on Animal Health Care Practices in Kendrapara District of Odisha, India. *International Letters of Natural Sciences*, *53*, 10–27. https://doi.org/10.18052/www.scipress.com/ILNS.53.10

Cross-sectional study of ethnoveterinary practices in Kendrapara District of Odisha, India. Qualitative methods including participant observation and interview. Study mentions cattle,

buffalo, sheep, and goats are commonly kept but does not match animal health concerns to species.

- The primary ailments of livestock are wound, diarrhoea, injury, fever, gastrointestinal problems and gynaecological problems, which are commonly treated with medicinal plants.

Sangma, D. B., & Manohara, T. (2018). The role of Garo tribes of Meghalaya (India) in the conservation and management of medicinal plants diversity used in treating livestock diseases. *Plant Science Today*, *5*(4), 155. https://doi.org/10.14719/pst.2018.5.4.416

Cross-sectional study of ethnoveterinary practices of Garo tribes of Meghalaya (India). Qualitative methods included group discussion and semi-structured interviews. Study mentions cattle, buffalo, sheep, and goats are commonly kept but does not match animal health concerns to species.

- Animal health concerns with the highest consensus of appropriate ethnoveterinary treatment were arthritis, rabies, reproductive diseases, retained placenta, and swollen body parts.
- Other health concerns commonly treated include diarrhoea, wounds, and ectoparasites.

Taku, A., Chhabra, R., & War, B. A. (2010). Footrot on a sheep breeding farm in the Himalayan state of Jammu and Kashmir. *Revue Scientifique et Technique (International Office of Epizootics)*, *29*(3), 671–675.

Outbreak investigation and confirmatory diagnosis of clinical case of footrot by culture and PCR on a sheep breeding farm in state of Jammu and Kashmir, India.

- Footrot case confirmed in Jammu and Kashmir.

Iqbal Yatoo, M., Raffiq Parray, O., Tauseef Bashir, S., Ahmed Bhat, R., Gopalakrishnan, A., Karthik, K., Vir Singh, S. (2019). Contagious caprine pleuropneumonia - a comprehensive review. *The Veterinary Quarterly*, *39*(1), 1–25.

Literature review of contagious caprine pleuropneumonia worldwide.

- In naive and native herds, 100% morbidity and 80% mortality have been noted. Cites a reported seroprevalence of about 10% in Pashmina goats from Jammu and Kashmir State, India.
- It is estimated that the total yearly cost of CCPP is about US$507 million in endemic areas thus involving major economic losses.

Nazki, S., Wani, S. A., Parveen, R., Ahangar, S. A., Kashoo, Z. A., Hamid, S., Dar, P. A. (2017).

Isolation, molecular characterization and prevalence of Clostridium perfringens in sheep and goats of Kashmir Himalayas, India. *Veterinary World*, *10*(12), 1501–1507. https://doi.org/10.14202/vetworld.2017.1501-1507

A screening for *C. perfringens* toxinotypes amongst purposively chosen small ruminants (152 sheep and 25 goats) from Kashmir Himalayas, India. This is NOT a representative survey as sample was purposive and included healthy and diarrheic animals, and morbid material of animals suspected to have died of enterotoxaemia.

- The toxinotypes A and D are prevalent in both sheep and goats. The highest prevalence of C. perfringens toxinotype D was observed in lambs (56.16%) and kids (46.16%) followed by 3.84% in adult sheep while it was absent in samples obtained from adult goats.
