# Supplementary material for: Prioritizing smallholder animal health needs in East Africa, West Africa, and South Asia using three approaches: Literature review, expert workshops, and practitioner surveys
Source: Prev Vet Med. 2021 Apr;189:105279. doi: 10.1016/j.prevetmed.2021.105279 (PMC8024747; doi:10.1016/j.prevetmed.2021.105279)
Supplement: Supplementary file 13 [file mmc13.pdf]

Smallholder Animal Health Needs Assessment
West Africa Cattle

GALVmed

August 5, 2019

Assembled by Zoë Campbell, Thembinkosi Ramuthivheli, Tom Osebe, and Paul Coleman

Meta-analysis summary

The literature review yielded 67 articles, 25 of which focused on impact, incidence, and prevalence, and are summarized below. The table below shows the number of articles in which each animal health concern appears in the literature about cattle in West Africa (many articles mention multiple concerns). The summarized articles are in the bibliography within this document. The full list of articles considered in the West Africa meta-analysis is available as a separate document.

The most mentioned concerns are foot and mouth disease, brucellosis, African Animal Trypanosomosis (AAT), ectoparasites, helminths, endoparasites, and contagious bovine pleuropneumonia (CBPP). Main topics of the green ranking articles that address impact and incidence/ prevalence include foot and mouth disease, brucellosis, AAT, CBPP, and heartwater.

Animal health concern	Frequency of mention (meta-analysis)	Frequency of mention (impact articles)	Main topic of green rank article
FMD	13	4	2
Brucellosis	12	5	1
AAT	9	3	1
Ectoparasites	9	2	0
Helminth	9	5	0
Endoparasites	8	4	0
CBPP	8	2	1
Tick-borne disease	5	2	0
Heartwater	5	3	1
Mange	4	1	0
Abortion	4	2	0
RVF	4	3	0
Fascioliasis	4	3	0
Babesiosis	3	1	0

TB	2	1	0
Anaplasmosis	2	1	0
Pasteurellosis	2	2	0
Dermatophilosis	2	1	0
Q fever	2	1	0
Schistosomiasis	2	1	0
Mastitis	1	1	0
Blackquarter	1	0	0
Anthrax	1	0	0
Theileriosis	1	1	0
Diarrhoea	1	0	0
Hydatidosis	1	1	0
Bloat	1	0	0
Colisepticemia	1	1	0
Cysticercosis	1	1	0
BSE	1	4	0
Histophilosis	1	5	0

*BSE – Bovine spongiform encephalopathy

Table of articles

Abbreviations:

RVF – Rift Valley fever

TBD – Tick-borne diseases

AAT – Animal African Trypanosomosis

FMD – Foot and mouth disease

TB – Tuberculosis

CBPP – Contagious bovine pleuropneumonia

Note: Clicking on the article number links to an expanded summary of the article below. The "web" link connects to the abstract of the article online or to full text for open access articles.

#	Citation	Country	Study design	Pathogen/ disease	Findings
1<br>1<br>web	(Souley Kouato et al., 2018)	Niger	Longitudinal, Outbreak investigation, Retrospective	Foot and mouth disease (FMD)	**Incidence/ Prevalence:** From 2007 to 2015, 791 clinical FMD outbreaks were reported from the eight regions of Niger.<br>**Impact:** The percentage of clinically affected cattle (morbidity) and dead animals (mortality) were respectively estimated at 70.30% and 3.59%. The average total costs of FMD at herd level were estimated at 499.34 euros (SD 196 euros). The cost

					of milk losses accounted for 33% of the total costs (average: 165.82 euros), while costs related to mortality of young bulls and heifer mortality were respectively 37.27% (average: 186.09 euros) and 29.52% (average: 147.42 euros) of the total costs of FMD at outbreak level.
2<br>3<br>web	(Pomeroy et al., 2015)	Cameroon	Longitudinal, Serosurvey, Pastoralists and smallholders, 498 cattle from 15 mobile herds and 15 sedentary herds	FMD	Five serotypes of FMDV affect cattle in the Far North Region of Cameroon. Viral serotypes can differ significantly in their epidemiological and immunological characteristics.
3<br>2<br>web	(Geidam et al., 2013)	Nigeria	Cross-sectional, Participatory epidemiology, Qualitative methods, 35 villages, Agro-pastoralists	FMD<br>CBPP<br>TB<br>Anthrax	**Incidence/ prevalence:** The most prevalent disease of cattle is foot and mouth disease (FMD). The transboundary animal diseases affecting cattle detected in the study area were FMD, contagious bovine pleuropneumonia (CBPP), bovine tuberculosis, and anthrax.
4<br>1<br>web	(Healy Profitós, Moritz, & Garabed, 2013)	Cameroon	Cross-sectional, Serosurvey, Ethnography with 21 pastoralists, 328 calves and 106 cows from	Brucellosis, AAT, FMD, Heartwater, Respiratory disease, CBPP	**Incidence/ prevalence:** 36% of the animals tested in the herds were infected with brucellosis. 21% of the calves on the calf rope and about 13% of their mothers had acute health problems. The main problems were diarrhoea, trypanosomiasis, and foot-and-mouth disease.

			10 mobile herds and 11 sedentary herds		**Perceptions:** Respondents were not overly concerned with brucellosis as a cause of fertility problems. Diseases held responsible for chronic sickness were trypanosomiasis (55% of all chronically sick animals in 21 herds), foot-and-mouth disease (17%), cough (17%), and brucellosis (11%). Pastoralists were more concerned about other infectious diseases like foot-and-mouth disease and contagious bovine pleuropneumonia.
5 2 web	(Kanouté, Gragnon, Schindler, Bonfoh, & Schelling, 2017)	Côte d'Ivoire	Cross-sectional Seroprevalence Agro-pastoralists	Brucellosis Q fever Rift Valley fever (RVF)	**Incidence/ prevalence:** The seroprevalence of *Brucella* spp. in cattle adjusted for clustering was 4.6%, for Q fever 13.9%, and for RVF 3.9%. Seroprevalences were within or below expected ranges for W. Africa. Small ruminants within the study were seronegative for *B. melitensis*. **Impact:** Abortions reported in most herds but not significantly associated with Q Fever or brucellosis seropositivity.
6 3 web	(Sylla, Sidimé, Sun, Doumbouya, & Cong, 2014)	Guinea	Cross-sectional Serosurvey 345 cattle Smallholders	Brucellosis	**Incidence/ Prevalence:** In both provinces, the prevalence mean was 8.67 %.
7 3 web	(Sanogo et al., 2013)	West Africa	Literature review, Multiple production systems	Brucellosis	*Brucella abortus* was the most prevalent species in cattle from West Africa. Biovars 1, 2, 3, 4, 6 and intermediate biovar 3/6 of *B. abortus* were reported in cattle in the region and biovars 3, recently identified in The Gambia and Ivory Coast, was the most commonly isolated. *Brucella*

					melitensis and/or *B. suis* have not been mentioned yet in cattle.
8<br>2<br>web	(Craighead et al., 2018)	14 West and Central African countries	Systematic literature review and expert workshops, Dairy	Brucellosis	**Incidence/ Prevalence:** Low individual seroprevalence and moderate to high herd seroprevalence is reported suggesting a state of endemicity with relatively low transmission rates. High seroprevalence estimates seen in developing systems such as those reported in peri-urban dairies in Dakar, Senegal (25% seroprevalence ± 4.9%) are concerning. There is insufficient data to conclude any meaningful estimates of the burden in small ruminant populations.
9<br>2<br>web	(Isaac, Ohiolei, Ebhodaghe, Igbinosa, & Eze, 2017)	Nigeria	Review, Economic estimates for 10 West and Central African countries, Multiple species	Animal African Trypanosomosis (AAT)	**Incidence/ Prevalence:** Reviews of serosurveys include ranges of seroprevalence in cattle from 0.5%-46.8%.<br>**Impact:** Estimates of impacts of AAT on agricultural GDP for 10 West and Central African countries ranges from 800 million USD to 1.6 billion USD (assuming 40% and 80% AAT impacts, respectively).
10<br>2<br>web	(Majekodunmi et al., 2013)	Nigeria	Longitudinal, Participatory rural appraisal, Prevalence, Pastoralists from 30 villages	AAT	**Incidence/ Prevalence:** High overall prevalence of AAT (46.8%) with showed significant seasonal variation between the dry and late wet season (3% {0.16 – 5.9%} difference in proportions) and a wide range of AAT prevalence at village level (88% - 95.6%)
11<br>3	(Nkegbe, Munkaila, &	Ghana	Cross-sectional Survey<br>77 cattle farms	Tick-borne diseases (TBD)<br>Helminths	**Impact:** Challenges to correct use of acaricides and anthelmintics include improper labelling (no acaricides within study displayed LC or lethal

web	Odoom-Sam, 2013)		Agro-pastoralists		concentration values) and low farmer literacy and education. Most formulation procedures were based on what colleague farmers related.
12<br>2<br>web	(Adjou Moumouni et al., 2018)	Benin	Cross-sectional, Serosurvey, Pastoralists, agro-pastoralists, and semi-extensive production, 207 cattle	TBD<br>Babesiosis<br>Theileriosis<br>Anaplasmosis<br>Heartwater	**Incidence/ Prevalence:** Out of 207 samples examined, 170 (82.1%), 109 (52.7%), 42 (20.3%) 24 (11.6%) and 1 (0.5%) were positive for *T. mutans, A. marginale, B. bigemina, B. bovis* and *E. ruminantium*, respectively. The characteristics of *Babesia* spp. and *A. marginale* infections in areas that are invaded and non-invaded by *R. microplus*, a major tick vector, are different.
13<br>3<br>web	(Squire et al., 2018)	Ghana	Cross-sectional Faecal samples 95 farmers Smallholders 1442 cattle, sheep, goats	Helminths	**Incidence/ Prevalence:** Gastrointestinal parasites were identified in a total of 64.3% (n = 534, CI = 61–67.6) of the 830 livestock, with strongylid nematodes the most prevalent (56.6%).
14<br>2<br>web	(Elelu, Ambali, Coles, & Eisler, 2016)	Nigeria	Cross-sectional, Faecal and serosurvey, 686 cattle from 65 households, Pastoralists	Fascioliasis, Paramphistomiasis, Trematode infections	**Incidence/ Prevalence:** Of the 686 faecal samples analysed, 74.9 %, 16.1 %, 7.3 % and 1.2 % were positive for infections with *Fasciola gigantica*, *paramphistomes*, *Dicrocoelium hospes* and *Schistosoma bovis* respectively.
15<br>3<br>web	(N.B. Alhaji & Babalobi, 2016)	Nigeria	Cross-sectional, Seroprevalence, Questionnaire, 765 cattle, 125 pastoralists,	CPBB	**Incidence/ Prevalence:** 16.2% (95% CI: 13.7, 19.0) of cattle seropositive. Highest seroprevalence of 25.3% was observed in Northern agro-ecological zone while lowest of 6.2% was in Eastern zone.

			Purposive sampling		
16<br>2<br>web	(Bell-Sakyi, Koney, Dogbey, & Walker, 2004)	Ghana	Longitudinal; Seroprevalence; 3,234 serum samples from goats, sheep, cattle; Smallholders and commercial	Heartwater	**Incidence/ Prevalence:** Virtually all cattle on the survey farms were exposed to *E. ruminantium* without suffering disease but a substantial proportion of sheep and goats escaped exposure and thus formed a susceptible population. *E. ruminantium* was detected in brains of 14, 36 and 4% of cattle, sheep and goats submitted for post mortem.
17<br>2<br>web	(Boussini et al., 2014)	Burkina Faso	Cross-sectional, Seroprevalence, Pastoralists, 520 serum samples of cattle, sheep, goats	Rift Valley fever	**Incidence/ Prevalence:** In the northern region, the overall seroprevalences were 15%, 8.33% and 6.66% for cattle, sheep and goats respectively, and in the central region overall seroprevalences were 15%, 5% and 0% respectively.
18<br>2<br>web	(Nma Bida Alhaji, Babalobi, Wungak, & Ularamu, 2018)	Nigeria	Cross-sectional, Serosurvey, Participatory epidemiology, 97 cattle, Pastoralists	RVF	**Incidence/ Prevalence:** 11.3% of the animals were seropositive for RVF.<br>**Perceptions:** This study highlighted the significant existing knowledge level about RVF contained in nomadic pastoralists, including clinical signs, viral pathway, and risk factors.
19<br>3<br>web	(Kelly et al., 2016)	Cameroon	Cross-sectional, Questionnaire, 112 pastoralists and 46 small scale dairy	Tuberculosis (TB)	**Incidence/ Prevalence:** Quarter of herdsmen in the North West Region reported cattle having died from tuberculosis or been informed about it from slaughter cases compared to <10% of those from the Vina Division.

			farmers across 2 regions		**Perceptions:** Dairy farmers (73.9%) and North West Region pastoralists (76.6%) were more aware of bovine TB than Vina Division pastoralists (40.8%).
20<br>2<br>web	(Nma Bida Alhaji & Isola, 2018)	Nigeria	Cross-sectional, Questionnaire, 384 pastoralists	Dermatophilosis	**Incidence/ Prevalence:** Overall clinical dermatophilosis burden was 3.6% (95%CI 3.46, 3.80).<br>**Impact:** The annual economic impact was estimated at $19.55 USD / head of cattle in the herd, the majority of which was due to losses from mortality.
21<br>2<br>web	(Shittu, Abdullahi, Jibril, Mohammed, & Fasina, 2012)	Nigeria	Cross-sectional, Prevalence study, Smallholders/ Pastoralists, 300 cows	Mastitis	**Incidence/ prevalence:** Overall herd-level prevalence rate for sub-clinical mastitis (SCM) was 85% (256/300).<br>The Rahaji breed had the highest prevalence of SCM with 66% (29/44), while the White Fulani breed had the least with 32% (57/176).<br>32% (97/300) had only one mammary quarter affected, 30% (91/300) had two quarters affected, 16% (48/300) had three quarters affected while 7% (20/300) had all four quarters affected.<br>**Perceptions:** Survey found that approximately 37% of the farmers do not wash their hands before milking.
22<br>2<br>web	(Carlson et al., 2019)	Sub-Saharan Africa	Global incidence database of human, wildlife, and livestock anthrax cases	Anthrax	**Incidence/ Prevalence:** Using past occurrence as a proxy for risk, in Sub-Saharan Africa 30.5 million cattle, 22.4 million goats, and 14.5 million sheep are at risk.

			from multiple sources spanning 71 countries from 1914-2018		**Impact:** Vaccination rates in Sub-Saharan Africa are 0-6%. Vaccination may be less effective for the 31 million livestock and 4.6 million poor livestock keepers in West Africa, where an endemic lineage of *B. anthracis* shares an anthrose-deficiency mutation that has been hypothesized to lead to a vaccine escape.
23 2 web	(Opara, Ukpong, Okoli, & Anosike, 2006)	Nigeria	Cross-sectional, Abattoir study, Multiple production systems, 25,800 cattle	Cysticercosis	**Incidence/ Prevalence:** 6,750 cattle (26.2%) were infected with *Cysticercus (C.) bovis*.
24 3 web	(Nwankiti, Ikeh, Asala, & Seuberlich, 2013)	Nigeria	Pilot disease surveillance study with 1,551 cattle presented for emergency slaughter	Bovine spongiform encephalopathy (BSE)	**Incidence/ Prevalence:** A total of 96 cattle, which correspond to 6.2%, presented clinical signs that supported a suspect of BSE. The caudal brainstem tissues of these animals were collected post-mortem and analysed for the disease-specific form of the prion protein using a rapid test None of the samples were positive for BSE.
25 3 web	(Odugbo et al., 2009)	Nigeria	Outbreak report Commercial dairy	Histophilosis	**Incidence/ Prevalence:** The outbreak occurred over a 30-day period, and attack and case-fatality rates were 0.4% and 50%, respectively. Histophilosis in cattle was confirmed for the first time in Nigeria.

Article summaries

Souley Kouato, B., Thys, E., Renault, V., Abatih, E., Marichatou, H., Issa, S., & Saegerman, C. (2018). Spatio-temporal patterns of foot-and-mouth disease transmission in cattle between 2007 and 2015 and quantitative assessment of the economic impact of the disease in Niger. *Transboundary and Emerging Diseases*, *65*(4), 1049–1066. https://doi.org/10.1111/tbed.12845

Study of spatiotemporal patterns and economic impact of foot-and-mouth disease (FMD) outbreaks in Niger based on the retrospective analysis of 9-year outbreak data.

- From 2007 to 2015, 791 clinical FMD outbreaks were reported from the eight regions of Niger.
- The percentage of clinically affected cattle (morbidity) and dead animals (mortality) were respectively estimated at 70.30% and 3.59%.
- The average total costs of FMD at herd level were estimated at 499.34 euros (SD 196 euros). The cost of milk losses accounted for 33% of the total costs (average: 165.82 euros), while costs related to mortality of young bulls and heifer mortality were respectively 37.27% (average: 186.09 euros) and 29.52% (average: 147.42 euros) of the total costs of FMD at outbreak level.

Pomeroy, L. W., Bjørnstad, O. N., Kim, H., Jumbo, S. D., Abdoulkadiri, S., & Garabed, R. (2015). Serotype-Specific Transmission and Waning Immunity of Endemic Foot-and-Mouth Disease Virus in Cameroon. *PloS One*, *10*(9), e0136642. https://doi.org/10.1371/journal.pone.0136642

A longitudinal study of serotype-specific transmission and waning immunity of endemic Foot-and-Mouth Disease Virus in Cameroon. Data were collected between 2010 and 2012 in four phases from 498 cattle from 15 mobile herds and 15 sedentary herds.

- Five serotypes of FMDV affect cattle in the Far North Region of Cameroon and viral serotypes can differ significantly in their epidemiological and immunological characteristics.
- Type O has the greatest force of infection and the longest duration of immunity.
- Serotypes SAT1 and O displayed endemic dynamics.
- Serotype A displayed epidemic dynamics.
- SAT2 and SAT3 did not sustain local chains of transmission.
- The result highlight important differences in transmission specific to each serotype and show that immunity wanes at rates specific to each serotype, which influences patterns of local persistence.

- FMDV endemicity is complex due to the presence of multiple co-circulating serotypes that differ in characteristics relating to transmission and immunity.

Geidam, Y. A., Ayi, V. K., Umar, I. I., Sunday, J., Musa, D., Goni, B., & Mwapu, D. N. (2013). Participatory disease surveillance in the detection of trans-boundary animal diseases (TADS) in Borno State of arid north-eastern Nigeria. *Bulletin of Animal Health and Production in Africa*, *61*(2), 231–239.

Participatory epidemiology methods including simple ranking, transect walk, and interview with key informants were employed in 35 communities in north-eastern Nigeria to detect animal diseases, particularly trans-boundary animal diseases.

- The most prevalent disease of cattle is foot and mouth disease (FMD). The transboundary animal diseases affecting cattle detected in the study area were FMD, contagious bovine pleuropneumonia (CBPP), bovine tuberculosis, and anthrax.

Healy Profitós, J. M., Moritz, M., & Garabed, R. B. (2013). What to do with chronically sick animals? Pastoralists' management strategies in the far north region of Cameroon. *Pastoralism*, *3*(1), 1–11. https://doi.org/10.1186/2041-7136-3-8

The objective of the ethnographic research project was to describe pastoralists' understandings of animal disease in north Cameroon and its impacts on fertility as well as their management strategies, in order to understand the apparent paradox that pastoralists do not remove chronically sick animals from their herd, even though this may negatively affect the long-term goal of pastoralists. Semi-structured interviews (ethnographies) were conducted with twenty-one pastoralists and 328 calves and 106 cows from 10 mobile herds and 11 sedentary herds were tested for brucellosis.

- 36% of the animals tested in the herds were infected with brucellosis. 21% of the calves on the calf rope and about 13% of their mothers had acute health problems. The main problems were diarrhoea, trypanosomosis, and foot-and-mouth disease.
- 8 of 11 sedentary herds had chronically sick animals with 8% of all sedentary cattle being sick versus 5 of 10 mobile herds with 3% of all mobile cattle being chronically sick.
- Respondents were not overly concerned with brucellosis as a cause of fertility problems.
- Diseases held responsible for chronic sickness were trypanosomosis (55% of all chronically sick animals in 21 herds), foot-and-mouth disease (17%), cough (17%), and brucellosis (11%).
- Pastoralists were more concerned about other infectious diseases like foot-and-mouth disease and contagious bovine pleuropneumonia.

Kanouté, Y. B., Gragnon, B. G., Schindler, C., Bonfoh, B., & Schelling, E. (2017). Epidemiology of brucellosis, Q Fever and Rift Valley Fever at the human and livestock interface in

northern Côte d'Ivoire. Acta Tropica, 165, 66–75.

A cross-sectional cluster survey conducted in three stages from 2012-2014 designed to measure the seroprevalence of brucellosis, Q Fever, and Rift Valley fever in cattle, small ruminants, and humans in northern Côte d'Ivoire. Sample of 633 cattle, 622 small ruminants, and 88 people across 63 villages.

- The seroprevalence of *Brucella* spp. in cattle adjusted for clustering was 4.6%, for Q fever 13.9%, and for RVF 3.9%.
- Seroprevalences were within or below expected ranges found in the literature for West Africa.
- Small ruminants within the study were seronegative for *B. melitensis*. Livestock brucellosis in West Africa has focused on cattle as a reservoir and *B. melitensis* (affecting small ruminants) has been reported in North, East, and Central Africa only.
- Abortions were reported in most of the herds but not significantly associated with Q fever or brucellosis seropositivity.
- Identifies a need to measure the economic impact of these three zoonoses.

Sylla, S., Sidimé, Y., Sun, Y., Doumbouya, S., & Cong, Y. (2014). Seroprevalence investigation of bovine brucellosis in Macenta and Yomou, Guinea. *Tropical Animal Health and Production*, *46*(7), 1185–1191. https://doi.org/10.1007/s11250-014-0625-2

Serosurvey of 345 purposively selected cattle farmers in two provinces in Guinea.

- In both provinces, the prevalence mean was 8.67 %.

Sanogo, M., Abatih, E., Thys, E., Fretin, D., Berkvens, D., & Saegerman, C. (2013). Importance of identification and typing of Brucellae from West African cattle: A review. *Veterinary Microbiology*, *164*(3–4), 202–211. https://doi.org/10.1016/j.vetmic.2013.02.009

A review to investigate available literature on strains of Brucella in order to provide knowledge on species and biovars reported in cattle from West Africa.

- *Brucella abortus* was the most prevalent species in cattle from West Africa
- Biovars 1, 2, 3, 4, 6 and intermediate biovar 3/6 of *B. abortus* were reported in cattle in the region and biovars 3, recently identified in The Gambia and Ivory Coast, was the most commonly isolated.
- *Brucella melitensis* and/or *B. suis* have not been mentioned yet in cattle.

Craighead, L., Meyer, A., Chengat, B., Musallam, I., Akakpo, J., Kone, P., Häsler, B. (2018). Brucellosis in West and Central Africa: A review of the current situation in a changing landscape of dairy cattle systems. *Acta Tropica*, *179*, 96–108. https://doi.org/10.1016/j.actatropica.2017.12.026

Systematic literature review of brucellosis in dairy cattle in fourteen West and Central African countries (Benin, Burkina Faso, Cameroon, Central African Republic, Côte d'Ivoire, Gabon, Ghana, Mali, Mauritania, Niger, Rwanda, Senegal, Chad & Togo). Review supplemented with opinions from expert workshops.

- Minimal surveillance and control activities for brucellosis taking place across the region.
- Low individual seroprevalence and moderate to high herd seroprevalence is reported suggesting a state of endemicity with relatively low transmission rates.
- Although variable and scarce, available literature suggests a reasonably high burden of brucellosis in the cattle populations of the 14 study countries. There is insufficient data to conclude any meaningful estimates of the burden in small ruminant populations.
- High seroprevalence estimates seen in developing systems such as those reported in peri-urban dairies in Dakar, Senegal (25% seroprevalence ± 4.9%) are concerning.
- Workshop attendees agreed that cattle vaccines were needed, but opinions were mixed on whether vaccination in small ruminants was necessary.

Isaac, C., Ohiolei, J. A. A., Ebhodaghe, F., Igbinosa, I. B. B., & Eze, A. A. A. (2017). Animal African Trypanosomiasis in Nigeria: A long way from elimination/eradication. *Acta Tropica*, *176*, 323–331. https://doi.org/10.1016/j.actatropica.2017.08.032

Review of AAT studies in Nigeria and other West and Central African countries. Many sources are pre-2002 and results with regards to seroprevalence vary widely with no attempt to address differing methods (eg: whether survey was in response to outbreak). The economic model incorporates two published assumptions about effect on GDP and output elasticity of livestock stock but could be more clearly explained (eg: Inconsistencies in figure column totals).

- Reviews of serosurveys include ranges of seroprevalence in cattle from 0.5%-46.8%.
- Estimates of impacts of AAT on agricultural GDP for 10 West and Central African countries ranges from 800 million USD to 1.6 billion USD (assuming 40% and 80% AAT impacts, respectively).

Majekodunmi, A. O., Fajinmi, A., Dongkum, C., Picozzi, K., Thrusfield, M. V, & Welburn, S. C. (2013). Trypanosomiasis in domestic cattle on the Jos Plateau, Nigeria: prevalence, distribution and risk factors. *Parasites & Vectors*, *6*(1), 239. https://doi.org/10.1186/1756-3305-6-239

A longitudinal two-stage cluster survey of pastoralists in 30 villages to investigate the epidemiology of trypanosomiasis as a re-emerging disease on the Jos plateau in North-Central Nigeria, examining the social factors that influence prevalence and seasonal variation of bovine trypanosomiasis.

- Overall prevalence of AAT across the Jos Plateau was very high (46.8%) and showed significant seasonal variation between the dry and late wet season (3% {0.16 – 5.9%} difference in proportions) and a wide range of AAT prevalence at village level (88% - 95.6%) prevalence at village level (88% - 95.6%).
- The influence of management factors on the epidemiology of trypanosomiasis highlights a growing trend in Nigerian cattle production; the traditional low input extensive production system is under threat due to expansion of human and livestock populations and arable farming.
- Pasture and water are becoming increasingly scarce and pastoralists are responding with management practices that increase risk of AAT.

Nkegbe, E., Munkaila, L., & Odoom-Sam, K. (2013). Regulatory compliance of small holder livestock farmers and herdsmen in the use of acaricides and gastrointestinal anthelmintics in sub-urban Accra, Ghana. *International Journal of Diseases and Disorders*, *1*, 39–44.

Seventy-seven livestock farms were visited in sub-urban Accra, Ghana to record labels on acaricides and anthelmintics and survey farmers about application.

- Lethal concentration (LC values) was not displayed on any of the acaricides. Labelling deficiencies for anthelmintics were mainly manufacture and expiration date.
- Application was done by herdsman in 56% of the farms, a population with the lowest numeracy and literacy compared to farm owners. Most formulation procedures were based on what colleague farmers related.
- Even though most of the acaricides and anthelmintics have satisfied the regulatory features, various levels of misapplication occurred in the study areas as a result of the low educational background of farmers, incomplete labelling information in some cases, as well as lack of adequate guidance from field Veterinary Officers.

Adjou Moumouni, P. F., Aplogan, G. L., Katahira, H., Gao, Y., Guo, H., Efstratiou, A., Xuan, X. (2018). Prevalence, risk factors, and genetic diversity of veterinary important tick-borne pathogens in cattle from Rhipicephalus microplus-invaded and non-invaded areas of Benin. *Ticks and Tick-Borne Diseases*, *9*(3), 450–464. https://doi.org/10.1016/j.ttbdis.2017.12.015

A cross-sectional seroprevalence survey of 207 cattle from multiple production systems to compare the prevalence of tick-borne diseases in *Rhipicephalus microplus*-invaded and non-invaded areas of Benin.

- Out of 207 samples examined, 170 (82.1%), 109 (52.7%), 42 (20.3%) 24 (11.6%) and 1 (0.5%) were positive for *T. mutans, A. marginale, B. bigemina, B. bovis* and *E. ruminantium*, respectively.
- Benin has been faced with an invasion of *Rhipicephalus microplus*, a major tick vector for babesiosis, theileriosis, and anaplasmosis over the last decade.
- The characteristics of *Babesia* spp. and *A. marginale* infections in areas that are invaded and non-invaded by *R. microplus*, a major tick vector, are different.

Squire, S. A., Yang, R., Robertson, I., Ayi, I., Squire, D. S., & Ryan, U. (2018). Gastrointestinal helminths in farmers and their ruminant livestock from the Coastal Savannah zone of Ghana. *Parasitology Research*, *117*(10), 3183–3194. https://doi.org/10.1007/s00436-018-6017-1

To identify the gastrointestinal helminths of veterinary, zoonotic and public health importance in farmers and their ruminant livestock in Ghana, faucal samples were collected from 95 farmers and their livestock (cattle = 328, sheep = 285 and goats = 217) and examined by microscopy and/or molecular techniques.

- Gastrointestinal parasites were identified in a total of 64.3% (n = 534, CI = 61–67.6) of the 830 livestock, with strongylid nematodes the most prevalent (56.6%), followed by *Paramphistomum* spp. (16.9%). Strongylid nematodes were the most prevalent helminths detected in cattle (65.9%), sheep (52.1%) and goats (49.5%).

Elelu, N., Ambali, A., Coles, G. C., & Eisler, M. C. (2016). Cross-sectional study of Fasciola gigantica and other trematode infections of cattle in Edu Local Government Area, Kwara State, north-central Nigeria. *Parasites & Vectors*, *9*(1), 470. https://doi.org/10.1186/s13071-016-1737-5

Cross-sectional study to investigate the prevalence of *Fasciola gigantica* and other trematode infections of cattle in Edu Local Government Area, Kwara State, north-central Nigeria. Two questionnaires were administered for household-level and individual cattle-level data. Faecal and blood samples were obtained from the 686 cattle from 65 households.

- Of the 686 faecal samples analysed, 74.9 %, 16.1 %, 7.3 % and 1.2 % were positive for infections with *Fasciola gigantica*, *paramphistomes*, *Dicrocoelium hospes* and *Schistosoma bovis* respectively.
- The most frequent species of trematode identified was *Fasciola gigantica,* present in 74.9 % of cattle.
- The high prevalence recorded for *F. gigantica* suggests that an anthelmintic resistance survey on the currently available drugs in the state would be advisable.

- Majority of co-infections were a combination of *F. gigantica* with *paramphistomes* 84/130 (64.6 %). High positive correlation between *F. gigantica* and *paramphistome* infections and hence likelihood of coinfections should be considered when carrying out anthelmintic therapy.

Alhaji, N.B., & Babalobi, O. O. (2016). Qualitative and quantitative impacts assessment of contagious bovine pleuropneumonia in Fulani pastoral herds of North-central Nigeria: The associated socio-cultural factors. *Preventive Veterinary Medicine*, *128*, 124–134. https://doi.org/10.1016/j.prevetmed.2016.04.004

Cross-sectional survey to investigate contatgious bovine pleuropneumonia (CPBB) in pastoral herds in North-central Nigeria. Involved serosurvey of 765 purposively chosen cattle and questionnaire of 125 pastoralists. Sampling strategy likely skews the seroprevalence rate up.

- 16.2% (95% CI: 13.7, 19.0) of cattle seropositive. Highest seroprevalence of 25.3% was observed in Northern agro-ecological zone while lowest of 6.2% was in Eastern zone.

Bell-Sakyi, L., Koney, E. B. M., Dogbey, O., & Walker, A. R. (2004). Ehrlichia ruminantium seroprevalence in domestic ruminants in Ghana; I. Longitudinal survey in the Greater Accra Region. *Veterinary Microbiology*, *100*(3–4), 175–188. https://doi.org/10.1016/j.vetmic.2004.02.010

Longitudinal survey (34 months) with collection of sera from 3,243 cattle, goats, and sheep from smallholder and commercial owners in Accra region of Ghana.

- Virtually all cattle on the survey farms were exposed to *E. ruminantium* without suffering disease but a substantial proportion of sheep and goats escaped exposure and thus formed a susceptible population.
- *Amblyomma variegatum* tick vectors were present on livestock in rural areas throughout the year, and first seroconversion occurred at any age, although the majority of calves seroconverted between 1 and 10 months old, sheep by 11 months, and goats by 7 months.
- *E. ruminantium* was detected in brains of 14, 36 and 4% of cattle, sheep and goats submitted for post mortem.

Boussini, H., Lamien, C. E., Nacoulma, O. G., Kaboré, A., Poda, G., & Viljoen, G. (2014). Prevalence of Rift Valley fever in domestic ruminants in the central and northern regions of Burkina Faso. *Revue Scientifique et Technique (International Office of Epizootics)*, *33*(3), 893–901.

Cross sectional study to determine the seroprevalence of Rift Valley fever in cattle, sheep and goats in selected areas of northern and central Burkina Faso. Sample size was 520 serum samples.

- The overall seroprevalence was 7.69% (40/520): the prevalence in the northern region was 9.37% and, in the central region, 5%.
- In the northern region, the overall seroprevalences were 15%, 8.33% and 6.66% for cattle, sheep and goats respectively, and in the central region overall seroprevalences were 15%, 5% and 0% respectively.
- Highest seroprevalence was found in the northern region around temporary ponds and dams.

Alhaji, Nma Bida, Babalobi, O. O., Wungak, Y., & Ularamu, H. G. (2018). Participatory survey of Rift Valley fever in nomadic pastoral communities of North-central Nigeria: The associated risk pathways and factors. *PLOS Neglected Tropical Diseases*, *12*(10), e0006858. https://doi.org/10.1371/journal.pntd.0006858

A cross-sectional participatory epidemiology survey of Rift Valley Fever was conducted in Fulani nomadic pastoral communities domiciled in Niger State. Ninety-seven cattle were randomly sampled from 15 purposively selected nomadic herds for a serosurvey.

- 11.3% of the animals were seropositive for RVF.
- This study highlighted the significant existing knowledge level about RVF contained in nomadic pastoralists, including clinical signs, viral pathway, and risk factors.

Kelly, R. F., Hamman, S. M., Morgan, K. L., Nkongho, E. F., Ngwa, V. N., Tanya, V., … Bronsvoort, B. M. de. C. (2016). Knowledge of Bovine Tuberculosis, Cattle Husbandry and Dairy Practices amongst Pastoralists and Small-Scale Dairy Farmers in Cameroon. *PLoS ONE*, *11*(1), e0146538. https://doi.org/10.1371/journal.pone.0146538

Cross-sectional study describes the knowledge of bovine tuberculosis, cattle management and milk processing practices amongst 112 pastoralists and 46 small-scale dairy farmers in two regions, North West Region and Vina Division, of Cameroon.

- Quarter of herdsmen in the North West Region reported cattle having died from tuberculosis or been informed about it from slaughter cases compared to <10% of those from the Vina Division.
- Dairy farmers (73.9%) and North West Region pastoralists (76.6%) were more aware of bovine TB than Vina Division pastoralists (40.8%).
- Awareness of disease transmission via milk was reported by about a quarter of pastoralists in the North West Region (28.3%) and Vina Division (26.1%) and about half of the dairy farmers (56.5%).
- A small proportion of North West Region (9.7%) and Vina Division (2.0%) pastoralists understood that bovine TB could be transmitted to people through milk, compared with 21.7% of dairy farmers.

Alhaji, N. B., & Isola, T. O. (2018). Pastoralists' knowledge and practices towards clinical bovine dermatophilosis in cattle herds of North-Central Nigeria: the associated factors, burden and economic impact. *Tropical Animal Health and Production*, *50*(2), 381–391. https://doi.org/10.1007/s11250-017-1445-y

Questionnaire administered to 384 pastoralists in North-Central Nigeria selected with a two-stage sampling method. Production losses were estimated from mortalities (values of cattle that died or culled due to dermatophilosis) and morbidities (estimated losses from dropped in sales of milk of lactating cows with dermatophilosis signs, sales of emaciated cattle with the disease signs and decrease value of draught power in cattle with the disease). Bovine dermatophilosis control costs in this research included costs of treatment of all sick cattle with anti- biotics and/or ethno-veterinary management.

- Overall clinical dermatophilosis burden was 3.6% (95%CI 3.46, 3.80).
- The annual economic impact was estimated at $19.55 USD / head of cattle in the herd, the majority of which was due to losses from mortality ($908,463.90 total annual loss/ 46,475 cattle = $19.55 / animal per year)

Shittu, A., Abdullahi, J., Jibril, A., Mohammed, A. A., & Fasina, F. O. (2012). Sub-clinical mastitis and associated risk factors on lactating cows in the Savannah Region of Nigeria. *BMC Veterinary Research*, *8*(1), 134. https://doi.org/10.1186/1746-6148-8-134

In the study of 300 cows in the Savannah region of Nigeria, California mastitis test was used to determine the prevalence of quarter and herd-level sub-clinical mastitis for individual cows and the herds. It also investigated potential risk factors.

- Overall herd-level prevalence rate for SCM was 85% (256/300).
- The Rahaji breed had the highest prevalence of SCM with 66% (29/44), while the White Fulani breed had the least with 32% (57/176).
- 32% (97/300) had only one mammary quarter affected, 30% (91/300) had two quarters affected, 16% (48/300) had three quarters affected while 7% (20/300) had all four quarters affected.
- Survey found that approximately 37% of the farmers do not wash their hands before milking.

Carlson, C. J., Kracalik, I. T., Ross, N., Alexander, K. A., Hugh-Jones, M. E., Fegan, M., … Blackburn, J. K. (2019). The global distribution of Bacillus anthracis and associated anthrax risk to humans, livestock and wildlife. *Nature Microbiology*. https://doi.org/10.1038/s41564-019-0435-4

Global incidence database of human, wildlife, and livestock anthrax cases from multiple sources spanning 71 countries from 1914-2018.

- Using past occurrence as a proxy for risk, in Sub-Saharan Africa, an estimated 30.5 million cattle, 22.4 million goats, and 14.5 million sheep are at risk for anthrax.
- Vaccination rates in Sub-Saharan Africa are 0-6%.
- Vaccination may be less effective for the 31 million livestock and 4.6 million poor livestock keepers in West Africa, where an endemic lineage of *B. anthracis* shares an anthrose-deficiency mutation that has been hypothesized to lead to a vaccine escape.

Opara, M. N., Ukpong, U. M., Okoli, I. C., & Anosike, J. C. (2006). Cysticercosis of slaughter cattle in southeastern Nigeria. *Annals of the New York Academy of Sciences*, *1081*, 339–346. https://doi.org/10.1196/annals.1373.048

Abattoir study of incidence of cysticercosis in local and exotic breeds of cattle slaughtered at 10 major abattoirs in Nigeria.

- 6750 cattle (26.2%) were infected with *Cysticercus (C.) bovis*.
- Contradictory results with regards to prevalence of cysticercosis between local and exotic breed cattle.
- Potential impact on sale of meat but impact not directly addressed.

Nwankiti, O. O., Ikeh, E. I., Asala, O., & Seuberlich, T. (2013). A Pilot Study for Targeted Surveillance of Bovine Spongiform Encephalopathy in Nigeria. *Transboundary and Emerging Diseases*, *60*(3), 279–283. https://doi.org/10.1111/j.1865-1682.2012.01340.x

A pilot disease surveillance study with 1,551 cattle presented for emergency slaughter in Nigeria examined for clinical signs of Bovine Spongiform Encephalopathy with positive cases analysed for BSE using a rapid test.

- A total of 96 cattle, which correspond to 6.2%, presented clinical signs that supported a suspect of BSE.
- None of the samples were positive for BSE.

Odugbo, M. O., Ogunjumo, S. O., Chukwukere, S. C., Kumbish, P. R., Musa, A., Ekundayo, S. O., … Haruna, G. (2009). The first report of Histophilus somni pneumonia in Nigerian dairy cattle. *Veterinary Journal (London, England : 1997)*, *181*(3), 340–342. https://doi.org/10.1016/j.tvjl.2008.03.007

An outbreak report from a large commercial dairy in Nigeria.

- The outbreak occurred over a 30-day period, and attack and case-fatality rates were 0.4% and 50%, respectively.
- Histophilosis in cattle was confirmed for the first time in Nigeria.
