# Supplementary material for: Prioritizing smallholder animal health needs in East Africa, West Africa, and South Asia using three approaches: Literature review, expert workshops, and practitioner surveys
Source: Prev Vet Med. 2021 Apr;189:105279. doi: 10.1016/j.prevetmed.2021.105279 (PMC8024747; doi:10.1016/j.prevetmed.2021.105279)
Supplement: Supplementary file 14 [file mmc14.pdf]

Smallholder Animal Health Needs Assessment
West Africa Poultry

GALVmed

August 5, 2019

Assembled by Zoë Campbell, Thembinkosi Ramuthivheli, Tom Osebe, and Paul Coleman

Meta-analysis summary

The literature review yielded 24 articles, 10 of which focused on impact, incidence, and prevalence, and are summarized below. The table below shows the number of articles in which each animal health concern appears in the literature about poultry in West Africa (many articles mention multiple concerns). No articles were given a green ranking for focus on both incidence/ prevalence and impact. The summarized articles are in the bibliography within this document. The full list of articles considered in the West Africa meta-analysis is available as a separate document.

The most mentioned concerns are avian influenza, Newcastle disease (ND), and infectious bursal disease (IBD). Although mentioned in East Africa literature, endoparasites, helminths, Marek's disease, salmonellosis, and mycoplasmosis are not mentioned in the West Africa literature for poultry. Although the articles mentioning avian influenza are numerous, most received the lowest ranking (red) with regards to relevance to smallholders. Some more relevant studies take advantage of the surveillance surrounding avian influenza outbreaks.

**Animal health concern**	**Frequency of mention (meta-analysis)**	**Frequency of mention (impact articles)**
Avian influenza	9	5
Newcastle disease	8	4
Infectious bursal disease	4	1
Fowl Pox	2	1
Ectoparasites	2	1
Chronic respiratory disease	2	2
Fowl cholera	2	1
Infectious bronchitis	2	1
Coccidiosis	1	1

Pasteurellosis	1	1
Fowl typhoid	1	0
Diarrhea	1	0
Chicken anemia virus	1	1

Table of articles

Abbreviations:<br><br>ND – Newcastle disease<br><br>IBD – Infectious bursal disease (Gumboro)<br><br>CRD – Chronic respiratory disease

Note: Clicking on the article number links to an expanded summary of the article below. The “web” link connects to the abstract of the article online or to full text for open access articles.

#	Citation	Country	Study design	Pathogen/ disease	Findings
1<br>2<br>web	(Geidam et al., 2013)	Nigeria	Cross-sectional, Participatory epidemiology, Qualitative methods, 35 villages, Agro-pastoralists	Newcastle disease (ND)	**Incidence/ prevalence:** The most prevalent disease affecting chickens in the study area was Newcastle disease. Morbidity and mortality rates of 79 and 72% respectively and a case fatality rate of 91% were estimated. Other diseases ranked by prevalence are ectoparasitism, fowl pox, coccidiosis, fowl cholera, Gumboro disease (IBD), and helminthosis.
2<br>2<br>web	(Samuel et al., 2013)	Benin and Togo	Cross-sectional, Seroprevalence/ typing, Immunization challenge,	ND	Of 68 specimens from 34 apparently health chickens in a live-bird market, 37 were positive for NDV, providing a total of 27 new strains of virulent Newcastle disease virus. Although the commercial vaccine strain La Sota protected against disease, it did not prevent infection and shedding of the West African strains. Phylogenetic analyses reveal

			Smallholders/ Pastoralists, Sample size - 27 NDV isolates from 34 chickens		genetic diversity within and among local NDV populations in Africa.
3<br>3<br>web	(Olobatoke, Mobayo, & Mathuthu, 2015)	Nigeria	Cross-sectional survey of 61 farmers Smallholders	ND, Infectious bursal disease (IBD)	**Perceptions:** 59% of farmers said ND most devastating chicken disease, 30% said IBD (Gumboro).<br>**Incidence/ Prevalence:** Newcastle disease reported during harmattan by 62% of farmers, during dry season by 18%, and during rainy season by 20%.
4<br>2<br>web	(Kouakou et al., 2015)	Ivory Coast	Molecular screening and seroprevalence of 22,000 avian swabs from longitudinal AI surveillance	ND<br>IBD	**Incidence/ Prevalence:** The NDV and IBV seroprevalences over the study period reached 22% and 72%, respectively. We found 14.7% pooled swabs (pool of 5 birds) positive by PCR for NDV and 14.6% for IBV. Both pathogens are therefore endemic in Ivory-Coast.
5<br>3<br>web	(Fenteng et al., 2011)	Ghana	Cross-sectional survey and sampling of wild and domestic birds, 1282 samples	Avian influenza, ND	**Incidence/ Prevalence:** All avian samples were negative for Influenza A viruses. Newcastle disease virus was detected in 8% (5/63) of the farms where chickens sampled showed respiratory and nervous signs.
6<br>2<br>web	(Saka et al., 2017)	Nigeria	Cross-sectional Survey<br>240 farms<br>Small commercial	IBD<br>Coccidiosis<br>CRD<br>ND<br>Fowl Pox	**Incidence/ Prevalence:** Infectious bursal disease (IBD), coccidiosis, and chronic respiratory disease (CRD) were the major diseases reported by 17.1%, 12.9%, and 7.1% of the farms, respectively.

					**Impact:** Mortality estimated at 54% for CRD, 51% for Fowl Pox, 39% for IBD, 28% for ND, 9% for coccidiosis. This is despite 97% of farms reporting vaccinated (not specified against which disease(s)).
7<br>3<br>web	(Fuller et al., 2015)	Cameroon, Benin, Côte d'Ivoire, Togo	Surveillance, sampling wild and domestic birds, 40,000 birds from 2010-2014	Avian influenza	**Incidence/ Prevalence:** Viral detection was 0% in West Africa for all birds and 3.65% in chickens in Central Africa (n = 1042).
8<br>2<br>web	(Fasina, Sirdar, & Bisschop, 2008)	Nigeria	Economic model Commercial	Avian influenza	**Impact:** An infection involving 10 % of the commercial bird population will cost Nigeria about $245 million and a worse scenario involving 70% of the commercial bird population may lead to a loss of around $700 million.
9<br>3<br>web	(Kouam, Tchouankui, & Ngapagna, 2019)	Cameroon	Outbreak investigation reports	Avian influenza	**Impact:** 24% poultry mortality in the H5N1 outbreaks (101,557 birds from varied production systems including pigeons, ducks, chickens, and guinea fowl).
10<br>3<br>web	(Oluwayelu et al., 2005)	Nigeria	Cross-sectional Outbreak investigation Commercial	Chicken anemia virus (CAV)	**Incidence/ Prevalence:** CAV isolated for the first time in Nigeria in commercial chickens through investigation of outbreaks tentatively identified as IBD.

GALVmed

Protecting Livestock – Improving Human Lives

Article summaries

Geidam, Y. A., Ayi, V. K., Umar, I. I., Sunday, J., Musa, D., Goni, B., & Mwapu, D. N. (2013). Participatory disease surveillance in the detection of trans-boundary animal diseases (TADS) in Borno State of arid north-eastern Nigeria. *Bulletin of Animal Health and Production in Africa*, *61*(2), 231–239.

Participatory epidemiology methods including simple ranking, transect walk, and interview with key informants were employed in 35 communities in north-eastern Nigeria to detect animal diseases, particularly trans-boundary animal diseases.

- The most prevalent disease affecting chickens in the study area was Newcastle disease. Morbidity and mortality rates of 79 and 72% respectively and a case fatality rate of 91% were estimated.
- Other diseases ranked by occurrence are ectoparasitism, fowl pox, coccidiosis, fowl cholera, Gumboro disease, and helminthosis.

Samuel, A., Nayak, B., Paldurai, A., Xiao, S., Aplogan, G. L., Awoume, K. A., Samal, S. K. (2013). Phylogenetic and Pathotypic Characterization of Newcastle Disease Viruses Circulating in West Africa and Efficacy of a Current Vaccine. *Journal of Clinical Microbiology*, *51*(3), 771–781. https://doi.org/10.1128/JCM.02750-12

The study characterized genetic and biological properties of NDV (Newcastle disease virus) strains isolated from two countries, Benin and Togo, of West Africa. Methods included analysis of serum samples from 34 apparently healthy chickens in a live-market, viral isolation, phylogenetic analyses of new strains, and an immunization challenge.

- Of 68 specimens from 34 apparently health chickens in a live-bird market, 37 were positive for NDV, providing a total of 27 new strains of virulent Newcastle disease virus. It is surprising that highly virulent NDV strains were isolated from apparently healthy chickens in live-bird markets in a setting where vaccination is not routinely used due to financial constraints.
- Although the commercial vaccine strain La Sota protected against disease, it did not prevent infection and shedding of the West African strains.
- Phylogenetic analysis showed that the NDV strains from West Africa form separate clusters of NDV that are related most loosely to genotype VII of the known NDV strains. This suggests there is genetic diversity within and among local NDV populations in Africa.

Olobatoke, R., Mobayo, E., & Mathuthu, M. (2015). Evaluation of local chicken production in Kogi State of Nigeria. International Journal of Agricultural Policy and Research, 3(10), 377–381.

Sixty-one questionnaires administered to chicken farmers in a local government area in Nigeria.

- Fifty-nine percent of farmers felt Newcastle disease is the most devastating chicken disease, 30% said Gumboro.
- Newcastle disease reported during harmattan by 62% of farmers, during dry season by 18%, and during rainy season by 20%.

Kouakou, A. V., Kouakou, V., Kouakou, C., Godji, P., Kouassi, A. L., Krou, H. A., Couacy-Hymann, E. (2015). Prevalence of Newcastle disease virus and infectious bronchitis virus in avian influenza negative birds from live bird markets and backyard and commercial farms in Ivory-Coast. *Research in Veterinary Science*, *102*, 83–88. https://doi.org/10.1016/j.rvsc.2015.07.015

Molecular screening and seroprevalence of 22,000 avian swabs from longitudinal avian influenza surveillance from 2010-2012 in Ivory Coast.

- The NDV (Newcastle disease virus) and IBV (Infectious bursal disease virus) seroprevalences over the study period reached 22% and 72%, respectively.
- We found 14.7% pooled swabs (pool of 5 birds) positive by PCR for NDV and 14.6% for IBV.
- Both pathogens are therefore endemic in Ivory-Coast.

Fenteng, D. E., Ampofo, W., Afari, E., Wurapa, F., Aryee, M., Koney, E., Awumbila, B. (2011). Avian influenza surveillance in domestic poultry and wild bird-Tema Metropolis, Ghana, 2010. *Journal of Commonwealth Veterinary Association*, *27*(2), 158–167.

Study designed to determine the profile of avian influenza A virus in wild and domestic birds in an area of Ghana that had experienced an AI outbreak two years previously.

- All avian samples were negative for Influenza A viruses. Newcastle disease virus was detected in 8% (5/63) of the farms where birds sampled showed respiratory and nervous signs.

Saka, J. O., Adesehinwa, A. O. K., Oyegbami, A., Omole, A. J., GyoungRae, C., YoungJoo, S., IkSoo, J. (2017). The effects of health management system on the growth of chicken small farm in southwest states of Nigeria. *Korean Journal of Poultry Science*, *44*(4), 225–233.

Cross-sectional questionnaire administered to 240 small and medium poultry farms in Nigeria. A small farm is defined as 20-1,000 chickens and 88% of the enrolled farms use intensive management systems. Growth in the poultry sector in Nigeria is being driven by small and medium farms, some of which might be considered smallholder enterprises.

- Infectious bursal disease (IBD), coccidiosis, and chronic respiratory disease (CRD) were the major diseases reported by 17.1%, 12.9%, and 7.1% of the farms, respectively.

- Mortality estimated at 54% for CRD, 51% for Fowl Pox, 39% for IBD, 28% for ND, 9% for coccidiosis. This is despite 97% of farms reporting vaccinated (not specified against which disease(s).

Fuller, T. L., Ducatez, M. F., Njabo, K. Y., Couacy-Hymann, E., Chasar, A., Aplogan, G. L., Smith, T. B. (2015). Avian influenza surveillance in Central and West Africa, 2010-2014. *Epidemiology and Infection*, *143*(10), 2205–2212.

Surveillance for Avian influenza in wild and domestic birds in West and Central Africa over a four year period.

- Viral detection was 0% in West Africa for all birds and 3.65% in chickens in Central Africa (n = 1042).

Fasina, F. O., Sirdar, M. M., & Bisschop, S. P. R. (2008). The financial cost implications of the highly pathogenic notifiable avian influenza H5N1 in Nigeria. *The Onderstepoort Journal of Veterinary Research*, *75*(1), 39–46.

The economic evaluation of avian influenza on the commercial poultry sector in Nigeria used epidemiological data, production figures and other input parameters to determine the final costs.

- An infection involving 10 % of the commercial bird population will cost Nigeria about $245 million and a worse scenario involving 70% of the commercial bird population may lead to a loss of around $700 million.

Kouam, M. K., Tchouankui, H. N., & Ngapagna, A. N. (2019). Epidemiological Features of Highly Pathogenic Avian Influenza in Cameroon. *Veterinary Medicine International*, *2019*, 1–5. https://doi.org/10.1155/2019/3796369

Data were collected from follow-up reports of the second Highly Pathogenic Avian Influenza outbreaks prepared by the veterinary health officials of Cameroon and sent to the World Organisation for Animal Health (OIE).

- Two HPAI virus strains (H5N1 and H5N8) were implicated, with H5N1 virus involved in the Center, South, West, and Adamawa regions outbreaks, and H5N8 involved in the Far North outbreak only.
- 24% poultry mortality in the H5N1 outbreaks (101,557 birds from varied production systems including pigeons, ducks, chickens, and guinea fowl). Mortality rate was not given for the H5N8 outbreak.

Oluwayelu, D. O., Todd, D., Ball, N. W., Scott, A. N. J., Oladele, O. A., Emikpe, B. O., Olaleye, O. D. (2005). Isolation and preliminary characterization of chicken anemia virus from

chickens in Nigeria. *Avian Diseases*, *49*(3), 446–450. https://doi.org/10.1637/7339-020705R.1

Outbreak investigation in commercial chickens in Nigeria.

- Chicken anaemia virus isolated for the first time in Nigeria in commercial chickens through investigation of outbreaks tentatively identified as IBD.
