# Supplementary material for: Prioritizing smallholder animal health needs in East Africa, West Africa, and South Asia using three approaches: Literature review, expert workshops, and practitioner surveys
Source: Prev Vet Med. 2021 Apr;189:105279. doi: 10.1016/j.prevetmed.2021.105279 (PMC8024747; doi:10.1016/j.prevetmed.2021.105279)
Supplement: Supplementary file 15 [file mmc15.pdf]

West Africa References

The references below were used for meta-analyses for all livestock types (cattle, small ruminants, and poultry). The sub-set of summarized articles are also included.

Samuel, A., Nayak, B., Paldurai, A., Xiao, S., Aplogan, G. L., Awoume, K. A., Samal, S. K. (2013). Phylogenetic and Pathotypic Characterization of Newcastle Disease Viruses Circulating in West Africa and Efficacy of a Current Vaccine. *Journal of Clinical Microbiology*, *51*(3), 771–781. https://doi.org/10.1128/JCM.02750-12

Ularamu, H. G., Ibu, J. O., Wood, B. A., Abenga, J. N., Lazarus, D. D., Wungak, Y. S., … Adah, M. I. (2017). Characterization of Foot-and-Mouth Disease Viruses Collected in Nigeria Between 2007 and 2014: Evidence for Epidemiological Links Between West and East Africa. *Transboundary and Emerging Diseases*, *64*(6), 1867–1876. https://doi.org/10.1111/tbed.12584

Dahmani, M., Sambou, M., Scandola, P., Raoult, D., Fenollar, F., & Mediannikov, O. (2017). Bartonella bovis and Candidatus Bartonella davousti in cattle from Senegal. *Comparative Immunology, Microbiology and Infectious Diseases*, *50*, 63–69. https://doi.org/10.1016/j.cimid.2016.11.010

Dean, A. S., Fournié, G., Kulo, A. E., Boukaya, G. A., Schelling, E., & Bonfoh, B. (2013). Potential Risk of Regional Disease Spread in West Africa through Cross-Border Cattle Trade. *PLoS ONE*, *8*(10), e75570. https://doi.org/10.1371/journal.pone.0075570

Alhaji, N. B., & Isola, T. O. (2018). Pastoralists' knowledge and practices towards clinical bovine dermatophilosis in cattle herds of North-Central Nigeria: the associated factors, burden and economic impact. *Tropical Animal Health and Production*, *50*(2), 381–391. https://doi.org/10.1007/s11250-017-1445-y

Gorna, K., Houndjè, E., Romey, A., Relmy, A., Blaise-Boisseau, S., Kpodékon, M., Bakkali Kassimi, L. (2014). First isolation and molecular characterization of foot-and-mouth disease virus in Benin. *Veterinary Microbiology*, *171*(1–2), 175–181. https://doi.org/10.1016/j.vetmic.2014.03.003

Isaac, C., Ohiolei, J. A. A., Ebhodaghe, F., Igbinosa, I. B. B., & Eze, A. A. A. (2017). Animal African Trypanosomiasis in Nigeria: A long way from elimination/eradication. *Acta Tropica*, *176*, 323–331. https://doi.org/10.1016/j.actatropica.2017.08.032

Kouakou, A. V., Kouakou, V., Kouakou, C., Godji, P., Kouassi, A. L., Krou, H. A., Couacy-Hymann, E. (2015). Prevalence of Newcastle disease virus and infectious bronchitis virus in avian influenza negative birds from live bird markets and backyard and commercial farms in Ivory-Coast. *Research in Veterinary Science*, *102*, 83–88. https://doi.org/10.1016/j.rvsc.2015.07.015

Mbuh, J. V. V, Ndamukong, K. J. N. J. N., Ntonifor, N., & Nforlem, G. F. F. (2008). Parasites of sheep and goats and their prevalence in Bokova, a rural area of Buea Sub Division, Cameroon. *Veterinary Parasitology*, *156*(3–4), 350–352. https://doi.org/10.1016/j.vetpar.2008.06.021

Motta, P., Handel, I. G., Rydevik, G., Hamman, S. M., Ngwa, V. N., Tanya, V. N., Porphyre, T. (2018). Drivers of Live Cattle Price in the Livestock Trading System of Central Cameroon. *Frontiers in Veterinary Science*, *4*. https://doi.org/10.3389/fvets.2017.00244

Ocholi, R. A., Kwaga, J. K. P., Ajogi, I., & Bale, J. O. O. (2005). Abortion due to Brucella abortus in sheep in Nigeria. *Revue Scientifique et Technique (International Office of Epizootics)*, *24*(3), 973–979.

Boukary, A. R., Saegerman, C., Abatih, E., Fretin, D., Alambédji Bada, R., De Deken, R., Thys, E. (2013). Seroprevalence and Potential Risk Factors for Brucella Spp. Infection in Traditional Cattle, Sheep and Goats Reared in Urban, Periurban and Rural Areas of Niger. *PLoS ONE*, *8*(12), e83175. https://doi.org/10.1371/journal.pone.0083175

Jagun, A., & Onoja, A. B. (2011). The current status of Peste des Petits Ruminant (PPR) in sheep in Ibadan southwestern Nigeria. *Journal of Commonwealth Veterinary Association*, *27*(2), 133–138.

Bok, J., Hogerwerf, L., Germeraad, E. A., Roest, H. I. J., Faye-Joof, T., Jeng, M., Secka, O. (2017). Coxiella burnetii (Q fever) prevalence in associated populations of humans and small ruminants in The Gambia. *Tropical Medicine & International Health*, *22*(3), 323–331. https://doi.org/10.1111/tmi.12827

Squire, S. A., Yang, R., Robertson, I., Ayi, I., Squire, D. S., & Ryan, U. (2018). Gastrointestinal helminths in farmers and their ruminant livestock from the Coastal Savannah zone of Ghana. *Parasitology Research*, *117*(10), 3183–3194. https://doi.org/10.1007/s00436-018-6017-1

Ngomtcho, S. C. H., Weber, J. S., Ngo Bum, E., Gbem, T. T., Kelm, S., & Achukwi, M. D. (2017). Molecular screening of tsetse flies and cattle reveal different Trypanosoma species

including T. grayi and T. theileri in northern Cameroon. *Parasites & Vectors*, *10*(1), 631. https://doi.org/10.1186/s13071-017-2540-7

Boussini, H., Lamien, C. E., Nacoulma, O. G., Kaboré, A., Poda, G., & Viljoen, G. (2014). Prevalence of Rift Valley fever in domestic ruminants in the central and northern regions of Burkina Faso. *Revue Scientifique et Technique (International Office of Epizootics)*, *33*(3), 893–901.

Seck, M. T., Bouyer, J., Sall, B., Bengaly, Z., & Vreysen, M. J. B. (2010). The prevalence of African animal trypanosomoses and tsetse presence in Western Senegal. *Parasite*, *17*(3), 257–265. https://doi.org/10.1051/parasite/2010173257

Mbengue, M., Diallo, A. A., Lo, F. T., Lo, M. M., Diop, M., Seck, P. S., Thiongane, Y. (2013). Réémergence de la péripneumonie contagieuse bovine au Sénégal. *Bulletin de La Société de Pathologie Exotique*, *106*(3), 212–215. https://doi.org/10.1007/s13149-013-0298-5

Sylla, S., Sidimé, Y., Sun, Y., Doumbouya, S., & Cong, Y. (2014). Seroprevalence investigation of bovine brucellosis in Macenta and Yomou, Guinea. *Tropical Animal Health and Production*, *46*(7), 1185–1191. https://doi.org/10.1007/s11250-014-0625-2

Kracalik, I. T., Kenu, E., Ayamdooh, E. N., Allegye-Cudjoe, E., Polkuu, P. N., Frimpong, J. A., … Blackburn, J. K. (2017). Modeling the environmental suitability of anthrax in Ghana and estimating populations at risk: Implications for vaccination and control. *PLOS Neglected Tropical Diseases*, *11*(10), e0005885. https://doi.org/10.1371/journal.pntd.0005885

Adjou Moumouni, P. F., Aplogan, G. L., Katahira, H., Gao, Y., Guo, H., Efstratiou, A., Xuan, X. (2018). Prevalence, risk factors, and genetic diversity of veterinary important tick-borne pathogens in cattle from Rhipicephalus microplus-invaded and non-invaded areas of Benin. *Ticks and Tick-Borne Diseases*, *9*(3), 450–464. https://doi.org/10.1016/j.ttbdis.2017.12.015

Owolodun, O. A., Yakubu, B., Jambol, A. R., Audu, B. J., Dogonyaro, B. B., & Luka, P. D. (2015). Further evidence for very virulent infectious bursal disease virus in vaccinated chickens in Nigeria. *Tropical Animal Health and Production*, *47*(7), 1437–1441. https://doi.org/10.1007/s11250-015-0880-x

Ntirandekura, J.-B., Matemba, L. E., Kimera, S. I., Muma, J. B., & Karimuribo, E. D. (2018). Association of Brucellosis with Abortion Prevalence in Humans and Animals in Africa: A Review. *African Journal of Reproductive Health*, *22*(3), 120–136. https://doi.org/10.29063/ajrh2018/v22i3.13

Akwuobu, C. A., Ayling, R. D., Chah, K. F., & Oboegbulem, S. I. (2014). Studies into the prevalence of Mycoplasma species in small ruminants in Benue State, North-central Nigeria. *Tropical Animal Health and Production*, *46*(6), 1087–1092. https://doi.org/10.1007/s11250-014-0613-6

Opara, M. N., Ukpong, U. M., Okoli, I. C., & Anosike, J. C. (2006). Cysticercosis of slaughter cattle in southeastern Nigeria. *Annals of the New York Academy of Sciences*, *1081*, 339–346. https://doi.org/10.1196/annals.1373.048

Fuller, T. L., Ducatez, M. F., Njabo, K. Y., Couacy-Hymann, E., Chasar, A., Aplogan, G. L., Smith, T. B. (2015). Avian influenza surveillance in Central and West Africa, 2010-2014. *Epidemiology and Infection*, *143*(10), 2205–2212.

Lopes, P. H., Akweongo, P., Wurapa, F., Afari, E., Sackey, S., Ocansey, D., & Nyarko, K. M. (2016). Bovine tuberculosis surveillance system 2valuation, Greater-Accra region, Ghana, 2006-2011. *Pan African Medical Journal*, *25*. https://doi.org/10.11604/pamj.supp.2016.25.1.6181

Kanouté, Y. B., Gragnon, B. G., Schindler, C., Bonfoh, B., & Schelling, E. (2017). Epidemiology of brucellosis, Q Fever and Rift Valley Fever at the human and livestock interface in northern Côte d'Ivoire. *Acta Tropica*, *165*, 66–75.

Suleiman, A., Bello, M., Dzikwi, A. A., Talba, A. M., Grema, H. A., & Geidam, Y. A. (2015). Serological prevalence of contagious bovine pleuropneumonia in agro-pastoral areas of Nigeria. *Tropical Animal Health and Production*, *47*(6), 1033–1042. https://doi.org/10.1007/s11250-015-0824-5

Dossa, L. H., Wollny, C., & Gauly, M. (2007). Smallholders' perceptions of goat farming in southern Benin and opportunities for improvement. *Tropical Animal Health and Production*, *39*(1), 49–57.

Gutiérrez-Expósito, D., Ferre, I., Ortega-Mora, L. M., & Álvarez-García, G. (2017). Advances in the diagnosis of bovine besnoitiosis: current options and applications for control. *International Journal for Parasitology*, *47*(12), 737–751. https://doi.org/10.1016/j.ijpara.2017.08.003

Ehizibolo, D. O., De Vleeschauwer, A. R., Haegeman, A., Lefebvre, D., Nwosuh, C. I., Umoh, J. U., … De Clercq, K. (2019). Serological and molecular epidemiology of foot-and-mouth disease viruses in agro-pastoralist livestock herds in the kachia grazing reserve, Nigeria. *Transboundary and Emerging Diseases*, tbed.13182. https://doi.org/10.1111/tbed.13182

Enwelu, I. A., Ezeuko, E. L., & Machebe, N. S. (2015). Challenges of smallholder sheep and goat keeping in rural communities of Aguata Agricultural Zone of Anambra State, Nigeria. *Indian Journal of Animal Research*, *49*(3), 373. https://doi.org/10.5958/0976-0555.2015.00131.4

Akinseye, V. O., Adesokan, H. K., Ogugua, A. J., Adedoyin, F. J., Otu, P. I., Kwaghe, A. V., Cadmus, S. I. (2016). Sero-epidemiological survey and risk factors associated with bovine brucellosis among slaughtered cattle in Nigeria. *Onderstepoort J Vet Res*, *83*(1). https://doi.org/10.4102/ojvr.v83i1.1002

Ogugua, A. J., Akinseye, V. O., Cadmus, E. O., Jolaoluwa Awosanya, E. A., Alabi, P. I., Idowu, O. S., … Cadmus, S. I. B. (2018). Prevalence and risk factors associated with bovine brucellosis in herds under extensive production system in southwestern Nigeria. *Tropical Animal Health and Production*, *50*(7), 1573–1582. https://doi.org/10.1007/s11250-018-1597-4

Majekodunmi, A. O., Fajinmi, A., Dongkum, C., Picozzi, K., MacLeod, E., Thrusfield, M. V, Welburn, S. C. (2013). Social factors affecting seasonal variation in bovine trypanosomiasis on the Jos Plateau, Nigeria. *Parasites & Vectors*, *6*(1), 293. https://doi.org/10.1186/1756-3305-6-293

Carlson, C. J., Kracalik, I. T., Ross, N., Alexander, K. A., Hugh-Jones, M. E., Fegan, M., Blackburn, J. K. (2019). The global distribution of Bacillus anthracis and associated anthrax risk to humans, livestock and wildlife. *Nature Microbiology*. https://doi.org/10.1038/s41564-019-0435-4

Pomeroy, L. W., Bjørnstad, O. N., Kim, H., Jumbo, S. D., Abdoulkadiri, S., & Garabed, R. (2015). Serotype-Specific Transmission and Waning Immunity of Endemic Foot-and-Mouth Disease Virus in Cameroon. *PloS One*, *10*(9), e0136642. https://doi.org/10.1371/journal.pone.0136642

Odugbo, M. O., Ogunjumo, S. O., Chukwukere, S. C., Kumbish, P. R., Musa, A., Ekundayo, S. O., … Haruna, G. (2009). The first report of Histophilus somni pneumonia in Nigerian dairy cattle. *Veterinary Journal (London, England : 1997)*, *181*(3), 340–342. https://doi.org/10.1016/j.tvjl.2008.03.007

Alhaji, N. B., Wungak, Y. S., & Bertu, W. J. (2016). Serological survey of bovine brucellosis in Fulani nomadic cattle breeds (Bos indicus) of North-central Nigeria: Potential risk factors and zoonotic implications. *Acta Tropica*, *153*, 28–35. https://doi.org/10.1016/j.actatropica.2015.10.003

Usman, I. S., Bzugu, P. M., Pur, J. T., & Abdullahi, A. (2017). Indigenous Control Methods for Parasites among Pastoralists Communities in Adamawa State, Nigeria. *Journal of Agricultural Extension*, *21*(1), 109. https://doi.org/10.4314/jae.v21i1.9

Morgan, K. L., Handel, I. G., Tanya, V. N., Hamman, S. M., Nfon, C., Bergman, I. E., … de C Bronsvoort, B. M. (2014). Accuracy of herdsmen reporting versus serologic testing for estimating foot-and-mouth disease prevalence. *Emerging Infectious Diseases*, *20*(12), 2048–2054. https://doi.org/10.3201/eid2012.140931

Musa, I., Ndahi, M., Pam, E., Okike, A., Cyrile, P., Saskia, H., & Jost, C. (2013). Using participatory epidemiological techniques to establish rural based poultry disease profiles: practical field experience in the Jos Plateau, Nigeria. *World's Poultry Science Journal*, *69*(2), 387–400. https://doi.org/10.1017/S0043933913000378

Kouam, M. K., Tchouankui, H. N., & Ngapagna, A. N. (2019). Epidemiological Features of Highly Pathogenic Avian Influenza in Cameroon. *Veterinary Medicine International*, *2019*, 1–5. https://doi.org/10.1155/2019/3796369

Abraham-Oyiguh, J., Adewumi, M. O., Onoja, A. B., Suleiman, I., Sulaiman, L. K., Ahmed, S. J., & Jagboro, S. T. (2015). Seroprevalence of Infectious Bursal Disease Virus in Local Chickens in Udu Local Government Area of Delta State, South East Nigeria. *Journal of Immunoassay and Immunochemistry*, *36*(4), 398–404. https://doi.org/10.1080/15321819.2014.973116

Hammami, P., Lancelot, R., & Lesnoff, M. (2016). Modelling the dynamics of post-vaccination immunity rate in a population of Sahelian sheep after a vaccination campaign against peste des petits ruminants virus. *PLoS ONE*, *11*(9), e0161769.

Tarnagda, Z., Yougbare, I., Kam, A., Tahita, M. C., & Ouedraogo, J. B. (2011). Prevalence of infectious bronchitis and Newcastle disease virus among domestic and wild birds in H5N1 outbreaks areas. *Journal of Infection in Developing Countries*, *5*(8), 565–570.

Suleiman, A., Jackson, E. L., & Rushton, J. (2015). Challenges of pastoral cattle production in a sub-humid zone of Nigeria. *Tropical Animal Health and Production*, *47*(6), 1177–1185. https://doi.org/10.1007/s11250-015-0845-0

Shittu, A., Abdullahi, J., Jibril, A., Mohammed, A. A., & Fasina, F. O. (2012). Sub-clinical mastitis and associated risk factors on lactating cows in the Savannah Region of Nigeria. *BMC Veterinary Research*, *8*(1), 134. https://doi.org/10.1186/1746-6148-8-134

Suleiman, A., Jackson, E., & Rushton, J. (2018). Perceptions, circumstances and motivators affecting the implementation of contagious bovine pleuropneumonia control programmes in Nigerian Fulani pastoral herds. *Preventive Veterinary Medicine*, *149*, 67–74. https://doi.org/10.1016/j.prevetmed.2017.10.011

Wu, T., & Perrings, C. (2018). The live poultry trade and the spread of highly pathogenic avian influenza: Regional differences between Europe, West Africa, and Southeast Asia. *PLOS ONE*, *13*(12), e0208197. https://doi.org/10.1371/journal.pone.0208197

Kelly, R. F., Mazeri, S., Hartley, C., Hamman, S. M., Ngu Ngwa, V., Nkongho, E. F., Williams, D. J. L. (2019). Assessing the performance of a Fasciola gigantica serum antibody ELISA to estimate prevalence in cattle in Cameroon. *BMC Veterinary Research*, *15*(1), 8. https://doi.org/10.1186/s12917-018-1762-z

Motta, P., Porphyre, T., Handel, I. G., Hamman, S. M., Ngu Ngwa, V., Tanya, V. N.,Bronsvoort, B. M. de C. (2019). Characterizing Livestock Markets, Primary Diseases, and Key Management Practices Along the Livestock Supply Chain in Cameroon. *Frontiers in Veterinary Science*, *6*, 101. https://doi.org/10.3389/fvets.2019.00101

Fenteng, D. E., Ampofo, W., Afari, E., Wurapa, F., Aryee, M., Koney, E., Awumbila, B. (2011). Avian influenza surveillance in domestic poultry and wild bird-Tema Metropolis, Ghana, 2010. *Journal of Commonwealth Veterinary Association*, *27*(2), 158–167.

Chah, J. M.Obi, U. P. and Ndofor-Foleng, H. M. (2013). Management practices and perceived training needs of small ruminant farmers in Anambra State, Nigeria. *African Journal of Agricultural Research*, *8*(22), 2713–2721. https://doi.org/10.5897/AJAR2013.7209

Alhaji, N. B., & Babalobi, O. O. (2016). Sero-positivity and associated risk factors for contagious bovine pleuropneumonia under two cattle production systems in North Central Nigeria. *Tropical Animal Health and Production*, *48*(2), 311–320. https://doi.org/10.1007/s11250-015-0952-y

Sodjinou, E., Henningsen, A., & Koudande, O. D. (2012). Improving village poultry's survival rate through community-based poultry health management: evidence from Benin. *Tropical Animal Health and Production*, *45*(1), 59–66. https://doi.org/10.1007/s11250-012-0174-5

Snoeck, C. J., Adeyanju, A. T., Owoade, A. A., Couacy-Hymann, E., Alkali, B. R., Ottosson, U., & Muller, C. P. (2013). Genetic Diversity of Newcastle Disease Virus in Wild Birds and Pigeons in West Africa. *Applied and Environmental Microbiology*, *79*(24), 7867–7874. https://doi.org/10.1128/AEM.02716-13

Bertram, M. R., Bravo de Rueda, C., Garabed, R., Dickmu Jumbo, S., Moritz, M., Pauszek, S., Arzt, J. (2018). Molecular Epidemiology of Foot-and-Mouth Disease Virus in the Context of Transboundary Animal Movement in the Far North Region of Cameroon. *Frontiers in Veterinary Science*, *5*. https://doi.org/10.3389/fvets.2018.00320

Awah-Ndukum, J., Mouiche, M. M. M., Bayang, H. N., Ngwa, V. N., Assana, E., Feussom, K. J. M., … Zoli, P. A. (2018). Seroprevalence and Associated Risk Factors of Brucellosis among Indigenous Cattle in the Adamawa and North Regions of Cameroon. *Veterinary Medicine International*, *2018*, 1–10. https://doi.org/10.1155/2018/3468596

Alhaji, N. B., Babalobi, O. O., Wungak, Y., & Ularamu, H. G. (2018). Participatory survey of Rift Valley fever in nomadic pastoral communities of North-central Nigeria: The associated risk pathways and factors. *PLOS Neglected Tropical Diseases*, *12*(10), e0006858. https://doi.org/10.1371/journal.pntd.0006858

Craighead, L., Meyer, A., Chengat, B., Musallam, I., Akakpo, J., Kone, P., Häsler, B. (2018). Brucellosis in West and Central Africa: A review of the current situation in a changing landscape of dairy cattle systems. *Acta Tropica*, *179*, 96–108. https://doi.org/10.1016/j.actatropica.2017.12.026

Germeraad, E. A., Hogerwerf, L., Faye-Joof, T., Goossens, B., van der Hoek, W., Jeng, M., Secka, O. (2016). Low Seroprevalence of Brucellosis in Humans and Small Ruminants in the Gambia. *PLOS ONE*, *11*(11), e0166035. https://doi.org/10.1371/journal.pone.0166035

Saka, J. O., Adesehinwa, A. O. K., Oyegbami, A., Omole, A. J., GyoungRae, C., YoungJoo, S., … IkSoo, J. (2017). The effects of health management system on the growth of chicken small farm in southwest states of Nigeria. *Korean Journal of Poultry Science*, *44*(4), 225–233.

Olobatoke, R., Mobayo, E., & Mathuthu, M. (2015). Evaluation of local chicken production in Kogi State of Nigeria. *International Journal of Agricultural Policy and Research*, *3*(10), 377–381.

Kamani, J., Apanaskevich, D. A., Gutiérrez, R., Nachum-Biala, Y., Baneth, G., & Harrus, S. (2017). Morphological and molecular identification of Rhipicephalus (Boophilus) microplus in Nigeria, West Africa: a threat to livestock health. *Experimental and Applied Acarology*, *73*(2), 283–296. https://doi.org/10.1007/s10493-017-0177-z

Ludi, A., Ahmed, Z., Pomeroy, L. W., Pauszek, S. J., Smoliga, G. R., Moritz, M., … Rodriguez, L. L. (2016). Serotype Diversity of Foot-and-Mouth-Disease Virus in Livestock without History of

Vaccination in the Far North Region of Cameroon. *Transboundary and Emerging Diseases*, *63*(1), e27–e38. https://doi.org/10.1111/tbed.12227

Adombi, C. M., Waqas, A., Dundon, W. G., Li, S., Daojin, Y., Kakpo, L., Diallo, A. (2017). Peste Des Petits Ruminants in Benin: Persistence of a Single Virus Genotype in the Country for Over 42 Years. *Transboundary and Emerging Diseases*, *64*(4), 1037–1044. https://doi.org/10.1111/tbed.12471

Ehizibolo, D. O., Haegeman, A., De Vleeschauwer, A. R., Umoh, J. U., Kazeem, H. M., Okolocha, E. C., … De Clercq, K. (2017). Detection and Molecular Characterization of Foot and Mouth Disease Viruses from Outbreaks in Some States of Northern Nigeria 2013-2015. *Transboundary and Emerging Diseases*, *64*(6), 1979–1990. https://doi.org/10.1111/tbed.12602

Bell-Sakyi, L., Koney, E. B. M., Dogbey, O., & Walker, A. R. (2004). Ehrlichia ruminantium seroprevalence in domestic ruminants in Ghana; I. Longitudinal survey in the Greater Accra Region. *Veterinary Microbiology*, *100*(3–4), 175–188. https://doi.org/10.1016/j.vetmic.2004.02.010

Farougou, S., Gagara, M., & Mensah, G. A. (2013). Prevalence of peste des petits ruminants in the arid zone in the Republic of Niger. *Onderstepoort J Vet Res*, *80*(1). https://doi.org/10.4102/ojvr.v80i1.544

Sundufu, A. J., Ansumana, R., Bockarie, A. S., Bangura, U., Lamin, J. M., Jacobsen, K. H., & Stenger, D. A. (2015). Syndromic surveillance of peste des petits ruminants and other animal diseases in Koinadugu district, Sierra Leone, 2011-2012. *Tropical Animal Health and Production*, *47*(2), 473–477. https://doi.org/10.1007/s11250-014-0736-9

Fasina, F. O., Sirdar, M. M., & Bisschop, S. P. R. (2008). The financial cost implications of the highly pathogenic notifiable avian influenza H5N1 in Nigeria. *The Onderstepoort Journal of Veterinary Research*, *75*(1), 39–46.

Bertu, W. J., Ducrotoy, M. J., Muñoz, P. M., Mick, V., Zúñiga-Ripa, A., Bryssinckx, W., Ocholi, R. A. (2015). Phenotypic and genotypic characterization of Brucella strains isolated from autochthonous livestock reveals the dominance of B. abortus biovar 3a in Nigeria. *Veterinary Microbiology*, *180*(1–2), 103–108. https://doi.org/10.1016/j.vetmic.2015.08.014

Zúquete, S. T., Coelho, J., Rosa, F., Vaz, Y., Cassamá, B., Padre, L., Leitão, A. (2017). Tick (Acari: Ixodidae) infestations in cattle along Geba River basin in Guinea-Bissau. *Ticks and Tick-Borne Diseases*, *8*(1), 161–169. https://doi.org/10.1016/j.ttbdis.2016.10.013

Kelly, R. F., Hamman, S. M., Morgan, K. L., Nkongho, E. F., Ngwa, V. N., Tanya, V., Bronsvoort, B. M. de. C. (2016). Knowledge of Bovine Tuberculosis, Cattle Husbandry and Dairy Practices amongst Pastoralists and Small-Scale Dairy Farmers in Cameroon. *PLoS ONE*, *11*(1), e0146538. https://doi.org/10.1371/journal.pone.0146538

Oluwayelu, D. O., Todd, D., Ball, N. W., Scott, A. N. J., Oladele, O. A., Emikpe, B. O., … Olaleye, O. D. (2005). Isolation and preliminary characterization of chicken anemia virus from chickens in Nigeria. *Avian Diseases*, *49*(3), 446–450. https://doi.org/10.1637/7339-020705R.1

Mamoudou, A., Njanloga, A., Hayatou, A., Suh, P. F., & Achukwi, M. D. (2016). Animal trypanosomosis in clinically healthy cattle of north Cameroon: epidemiological implications. *Parasites & Vectors*, *9*(1), 206. https://doi.org/10.1186/s13071-016-1498-1

Morakinyo, O. A., & Babalobi, O. O. (2013). Participatory appraisal of Peste des Petite Ruminants (PPR) outbreaks in Iseyin local government area of Oyo State, Nigeria. In *Southern African Society for Veterinary Epidemiology and Preventive Medicine* (pp. 28–33). KZN Dolphin Coast, South Africa.

Mafimisebi, T. E., Oguntade, A. E., Fajemisin, A. N., & Aiyelari, O. P. (2012). Local knowledge and socio-economic determinants of traditional medicines' utilization in livestock health management in Southwest Nigeria. *Journal of Ethnobiology and Ethnomedicine*, *8*(1), 2. https://doi.org/10.1186/1746-4269-8-2

Sanogo, M., Abatih, E., Thys, E., Fretin, D., Berkvens, D., & Saegerman, C. (2013). Importance of identification and typing of Brucellae from West African cattle: A review. *Veterinary Microbiology*, *164*(3–4), 202–211. https://doi.org/10.1016/j.vetmic.2013.02.009

Webster, B. L., Diaw, O. T., Seye, M. M., Webster, J. P., & Rollinson, D. (2013). Introgressive Hybridization of Schistosoma haematobium Group Species in Senegal: Species Barrier Break Down between Ruminant and Human Schistosomes. *PLoS Neglected Tropical Diseases*, *7*(4), e2110. https://doi.org/10.1371/journal.pntd.0002110

Nkegbe, E., Munkaila, L., & Odoom-Sam, K. (2013). Regulatory compliance of small holder livestock farmers and herdsmen in the use of acaricides and gastrointestinal anthelmintics in sub-urban Accra, Ghana. *International Journal of Diseases and Disorders*, *1*, 39–44.

Nwankiti, O. O., Ikeh, E. I., Asala, O., & Seuberlich, T. (2013). A Pilot Study for Targeted Surveillance of Bovine Spongiform Encephalopathy in Nigeria. *Transboundary and Emerging Diseases*, *60*(3), 279–283. https://doi.org/10.1111/j.1865-1682.2012.01340.x

Awa, D. N., Adakal, H., Luogbou, N. D. D., Wachong, K. H., Leinyuy, I., & Achukwi, M. D. (2015). Cattle ticks in Cameroon: Is Rhipicephalus (Boophilus) microplus absent in Cameroon and the Central African region? *Ticks and Tick-Borne Diseases*, *6*(2), 117–122. https://doi.org/10.1016/j.ttbdis.2014.10.005

Dalis, J. S., Kazeem, H. M., Kwaga, J. K. P., Kwanashie, C. N., Yakubu, B., Owolodun, O. A., & Jambol, A. R. (2018). Molecular characterization of dermatophytes isolated from cattle in Plateau State, Nigeria. *Veterinary Microbiology*, *219*, 212–218. https://doi.org/10.1016/j.vetmic.2018.04.022

Alhaji, N. B., & Babalobi, O. O. (2016). Qualitative and quantitative impacts assessment of contagious bovine pleuropneumonia in Fulani pastoral herds of North-central Nigeria: The associated socio-cultural factors. *Preventive Veterinary Medicine*, *128*, 124–134. https://doi.org/10.1016/j.prevetmed.2016.04.004

Adedeji, A. J., Dashe, Y., Akanbi, O. B., Woma, T. Y., Jambol, A. R., Adole, J. A., Okewole, P. (2019). Co-infection of peste des petits ruminants and goatpox in a mixed flock of sheep and goats in Kanam, North Central Nigeria. *Veterinary Medicine and Science*, vms3.170. https://doi.org/10.1002/vms3.170

Hector, E., Elelu, N., Ferrolho, J., Couto, J., Sanches, G., Antunes, S. Eisler, M. (2019). PCR detection of Ehrlichia ruminantium and Babesia bigemina in cattle from Kwara State, Nigeria: unexpected absence of infection. *Parasitology Research*, *118*(3), 1025–1029. https://doi.org/10.1007/s00436-019-06204-1

Alhaji, N., & Babalobi, O. (2015). Socio-cultural factors influencing transmission of Mycoplasma mycoides mycoides small colony in pastoral cattle herds of north-central Nigeria. *Vom Journal of Veterinary Science*, *10*, 1–13.

Arowolo, O. O., Banmeke, T. O. A., & Ajayi, M. T. (2012). Assessment of the training needs of poultry farmers in Ovia and Oredo Local Government Areas of Edo State, Nigeria. *African Journal of Livestock Extension*, *10*, 23–30.

Ogundiyi, A. I., Bemji, M. N., Adebambo, O. A., Dipeolu, M. A., Onagbesan, O. M., James, I. J., & Osinowo, O. A. (2012). Prevalence of mange among West African Dwarf sheep and goats

and associated haematological and biochemical parameters. *Tropical Animal Health and Production*, *44*(6), 1263–1269. https://doi.org/10.1007/s11250-011-0067-z

Healy Profitós, J. M., Moritz, M., & Garabed, R. B. (2013). What to do with chronically sick animals? Pastoralists' management strategies in the far north region of Cameroon. *Pastoralism*, *3*(1), 1–11. https://doi.org/10.1186/2041-7136-3-8

Majekodunmi, A., Fajinmi, A., Dongkum, C., Picozzi, K., Thrusfield, M., & Welburn, S. (2013). A longitudinal survey of African animal trypanosomiasis in domestic cattle on the Jos Plateau, Nigeria: prevalence, distribution and risk factors. *Parasites & Vectors*, *6*(1), 239. https://doi.org/10.1186/1756-3305-6-239

Mazeri, S., Scolamacchia, F., Handel, I. G., Morgan, K. L., Tanya, V. N., & Bronsvoort, B. M. deC. (2013). Risk factor analysis for antibodies to Brucella, Leptospira and C. burnetii among cattle in the Adamawa Region of Cameroon: a cross-sectional study. *Tropical Animal Health and Production*, *45*(2), 617–623. https://doi.org/10.1007/s11250-012-0268-0

Oluwayelu, D., Adebiyi, A., & Tomori, O. (2018). Endemic and emerging arboviral diseases of livestock in Nigeria: a review. *Parasites & Vectors*, *11*(1), 337. https://doi.org/10.1186/s13071-018-2911-8

Esemu, S. N., Besong, W. O., Ndip, R. N., & Ndip, L. M. (2013). Prevalence of Ehrlichia ruminantium in adult Amblyomma variegatum collected from cattle in Cameroon. *Experimental and Applied Acarology*, *59*(3), 377–387. https://doi.org/10.1007/s10493-012-9599-9

Souley Kouato, B., Thys, E., Renault, V., Abatih, E., Marichatou, H., Issa, S., & Saegerman, C. (2018). Spatio-temporal patterns of foot-and-mouth disease transmission in cattle between 2007 and 2015 and quantitative assessment of the economic impact of the disease in Niger. *Transboundary and Emerging Diseases*, *65*(4), 1049–1066. https://doi.org/10.1111/tbed.12845

Geidam, Y. A., Ayi, V. K., Umar, I. I., Sunday, J., Musa, D., Goni, B., & Mwapu, D. N. (2013). Participatory disease surveillance in the detection of trans-boundary animal diseases (TADS) in Borno State of arid north-eastern Nigeria. *Bulletin of Animal Health and Production in Africa*, *61*(2), 231–239.

Akidarju, M. S., Onyemaechi, E. G., & Dauda, M. G. (2010). An assessment of some poultry management practices and disease recognition by poultry farmers in Maiduguri arid zone,

Nigeria. *World's Poultry Science Journal*, *66*(2), 285–296. https://doi.org/10.1017/S0043933910000334

Jibril, A. H., Umoh, J. U., Kabir, J., Gashua, M. M., & Bello, M. B. (2015). Application of participatory epidemiology techniques to investigate Newcastle disease among rural farmers in Zamfara state, Nigeria. *The Journal of Applied Poultry Research*, *24*(2), 233–239. https://doi.org/10.3382/japr/pfv012

Henning, J., Bett, B., Okike, I., Abdu, P., & Perry, B. (2013). Incidence of Highly Pathogenic Avian Influenza H5N1 in Nigeria, 2005-2008. *Transboundary and Emerging Diseases*, *60*(3), 222–230. https://doi.org/10.1111/j.1865-1682.2012.01331.x

Elelu, N. (2017). Epidemiological risk factors of knowledge and preventive practice regarding avian influenza among poultry farmers and live bird traders in Ikorodu, Lagos State, Nigeria. *International Journal of Veterinary Science and Medicine*, *5*(1), 47–52. https://doi.org/10.1016/j.ijvsm.2017.03.002

Elelu, N., Ambali, A., Coles, G. C., & Eisler, M. C. (2016). Cross-sectional study of Fasciola gigantica and other trematode infections of cattle in Edu Local Government Area, Kwara State, north-central Nigeria. *Parasites & Vectors*, *9*(1), 470. https://doi.org/10.1186/s13071-016-1737-5
