# Supplementary material for: Prioritizing smallholder animal health needs in East Africa, West Africa, and South Asia using three approaches: Literature review, expert workshops, and practitioner surveys
Source: Prev Vet Med. 2021 Apr;189:105279. doi: 10.1016/j.prevetmed.2021.105279 (PMC8024747; doi:10.1016/j.prevetmed.2021.105279)
Supplement: Supplementary file 16 [file mmc16.pdf]

Smallholder Animal Health Needs Assessment
West Africa Small Ruminants

GALVmed

August 5, 2019

Assembled by Zoë Campbell, Thembinkosi Ramuthivheli, Tom Osebe, and Paul Coleman

Meta-analysis summary

The literature review yielded 34 articles, 14 of which focused on impact, incidence, and prevalence, and are summarized below. The table below shows the number of articles in which each animal health concern appears in the literature about small ruminants in West Africa (many articles mention multiple concerns). There were no articles given a green ranking for focus on both incidence/ prevalence and impact. The summarized articles are in the bibliography within this document. The full list of articles considered in the West Africa meta-analysis is available as a separate document.

The most mentioned concerns are Peste des Petits Ruminants (PPR), ectoparasites, helminths, endoparasites, brucellosis, and mange. Although mentioned in East Africa literature, toxoplasmosis, sheep pox, and orf are not mentioned in the West Africa literature for small ruminants.

**Animal health concern**	**Frequency of mention (meta-analysis)**	**Frequency of mention (impact articles)**
Peste des Petits Ruminants	9	3
Ectoparasites	8	3
Helminths	7	4
Endoparasites	6	3
Brucellosis	6	2
Mange	5	2
Rift Valley fever	3	2
Abortion	3	1
Tick-borne disease	2	2
Foot and mouth disease	2	0
African Animal Trypanosomiasis	2	0
Pasteurellosis	2	2

Dermatophilosis	2	1
Q fever	2	1
Diarrhea	2	1
Babesiosis	2	1
Heartwater	2	2
Schistosomiasis	2	1
Contagious Caprine Pleuropneumonia	1	0
Goat pox	1	1
Theileriosis	1	1
Anaplasmosis	1	0
Anthrax	1	1
Hydatidosis	1	0
Haemonchosis	1	3

Table of articles

Abbreviations:<br>RVF – Rift Valley Fever<br>PPR – Peste des Petits Ruminants<br>AAT – Animal African Trypanosomiasis<br>GTP – Goat pox<br>CCPP - Contagious Caprine Pleuropneumonia

Note: Clicking on the article number links to an expanded summary of the article below. The “web” link connects to the abstract of the article online or to full text for open access articles.

#	Citation	Country	Study design	Pathogen/ disease	Findings
1<br>2<br>web	(Geidam et al., 2013)	Nigeria	Cross-sectional, Participatory epidemiology, Qualitative methods, 35 villages, Agro-pastoralists	PPR	**Incidence/ prevalence:** The most prevalent disease affecting sheep and goats in the study area was Peste des Petits Ruminants (PPR).<br>**Impact:** PPR was estimated to have a morbidity rate of 73%, mortality rate of 62% and a case fatality rate of 84%.

2<br>3<br>web	(Jagun & Onoja, 2011)	Nigeria	Cross-sectional Seroprevalence 200 sheep	PPR	**Incidence/ Prevalence:** 73% of the sheep had detectable antibody levels. Unspecified strategy for sampling animals. Unspecified production system.
3<br>3<br>web	(Ishag, Saeed, & Ali, 2015)	Sudan	Longitudinal, Outbreak investigation, Serosurvey, Smallholders, 209 serum samples	PPR	**Incidence/ Prevalence:** Out of 209 serum samples tested, 113 (54%) were found positive for Peste des Petits Ruminants virus.<br>**Impact:** A mortality rate of 4.2% was reported across eight different outbreaks.
4<br>3<br>web	(Morakinyo & Babalobi, 2013)	Nigeria	Qualitative Focus groups Interviews Participatory epidemiology Smallholders	PPR Diarrhea Mange Tetanus	**Perceptions:** Most farmers and goat sellers were unaware of PPR until they experienced outbreaks in their flocks. There was little awareness of preventive measures (including vaccination).<br>**Incidence/ Prevalence:** Goat-owning respondents symptomatically identified PPR, diarrhea, mange, and tetanus as the major diseases and health problems in the community, with PPR identified as the major disease by almost all the respondents.
5<br>3<br>web	(Adedeji et al., 2019)	Nigeria	Outbreak investigation, Virus detection, Single outbreak of sheep and goats, 9 sera and 7 tissue samples	Peste des Petits Ruminants (PPR), Goat pox (GTP)	**Incidence/ Prevalence:** This is the first report of concurrent infection of PPR and GTP in mixed flock of sheep and goats in Nigeria.
6	(Squire et al., 2018)	Ghana	Cross-sectional Fecal samples	Helminths	**Incidence/ Prevalence:** Gastrointestinal parasites were identified in a total of 64.3% (n = 534, CI = 61–67.6) of the

3<br>web			95 farmers<br>Smallholders<br>1442 cattle, sheep, goats		830 livestock, with strongylid nematodes the most prevalent (56.6%).
7<br>2<br>web	(Kanouté et al., 2017)	Côte d'Ivoire	Cross-sectional<br>Seroprevalence<br>633 cattle, 622 small ruminants<br>Agro-pastoralists	Brucellosis<br>Q fever<br>RVF	**Incidence/ prevalence:** The seroprevalence of Q fever was 9.4% in sheep and 12.4% in goats. The seroprevalence of RVF was 2.4% in sheep and 0% in goats. Seroprevalences were within or below expected ranges for W. Africa. Small ruminants within the study were seronegative for *B. melitensis*.<br>**Impact:** Abortions reported in most herds but not significantly associated with Q Fever or brucellosis seropositivity.
8<br>2<br>web	(Bell-Sakyi et al., 2004)	Ghana	Longitudinal; Seroprevalence; 3,234 serum samples from goats, sheep, cattle; Smallholders and commercial	Heartwater	**Incidence/ Prevalence:** Virtually all cattle on the survey farms were exposed to *E. ruminantium* without suffering disease but a substantial proportion of sheep and goats escaped exposure and thus formed a susceptible population. *E. ruminantium* was detected in brains of 14, 36 and 4% of cattle, sheep and goats submitted for post mortem.
9<br>3<br>web	(Chah, J. M.Obi, U. P. and Ndofor-Foleng, 2013)	Nigeria	Cross-sectional, Questionnaire, 60 goat farmers, smallholder	Gastro-intestinal symptoms	**Incidence/ Prevalence:** Majority (62%) of the farmers indicated that diarrhoea was the most frequently observed health problem in their flocks. Fifty-five percent of the respondents reported "bloat" as a frequent health problem.

10<br>2<br>web	(Craighead et al., 2018)	14 West and Central African countries	Systematic literature review and expert workshops, Dairy	Brucellosis	**Incidence/ Prevalence:** There is insufficient data to conclude any meaningful estimates of the burden in small ruminant populations.<br>**Perceptions:** Workshop attendees agreed that cattle vaccines were needed, but opinions were mixed on whether vaccination in small ruminants was necessary.
11<br>2<br>web	(Germeraad et al., 2016)	Gambia	Cross-sectional, Questionnaire, Serosurvey of 599 humans and 623 small ruminants from 12 rural villages and 500 small ruminants from abattoirs and livestock markets	Brucellosis	**Incidence/ Prevalence:** One human and 14 sheep sera were positive by the Rose Bengal Test. Other tests were negative. The hypothesis that brucellosis is endemic in the investigated rural and urban areas of The Gambia could not be confirmed in the present study. Seropositivity for *Brucella* spp. was found in only a very small percentage of humans and small ruminants in rural and urban Gambian sites, although risk factors for obtaining *Brucella* infection such as raw milk consumption were present.
12<br>2<br>web	(Boussini et al., 2014)	Burkina Faso	Cross-sectional, Seroprevalence, Pastoralists, 520 serum samples of cattle, sheep, goats	Rift Valley fever	**Incidence/ Prevalence:** In the northern region, the overall seroprevalences were 15%, 8.33% and 6.66% for cattle, sheep and goats respectively, and in the central region overall seroprevalences were 15%, 5% and 0% respectively.
13<br>2<br>web	(Isaac et al., 2017)	Nigeria	Review, Economic estimates for 10 West and Central African countries,	AAT	**Incidence/ Prevalence:** Reviews of serosurveys include ranges of seroprevalence in sheep from 1.2%-40.9% and in goats from 0.7%-75%.<br>**Impact:** Estimates of impacts of AAT on agricultural GDP for 10 West and Central African countries ranges from 800

			Multiple species		million USD to 1.6 billion USD (assuming 40% and 80% AAT impacts, respectively).
14<br>2<br>web	(Carlson et al., 2019)	Sub-Saharan Africa	Global incidence database of human, wildlife, and livestock anthrax cases from multiple sources spanning 71 countries from 1914-2018	Anthrax	**Incidence/ Prevalence:** Using past occurrence as a proxy for risk, in Sub-Saharan Africa 30.5 million cattle, 22.4 million goats, and 14.5 million sheep are at risk.<br>**Impact:** Vaccination rates in Sub-Saharan Africa are 0-6%. Vaccination may be less effective for the 31 million livestock and 4.6 million poor livestock keepers in West Africa, where an endemic lineage of *B. anthracis* shares an anthrose-deficiency mutation that has been hypothesized to lead to a vaccine escape.

Summary of articles

Geidam, Y. A., Ayi, V. K., Umar, I. I., Sunday, J., Musa, D., Goni, B., & Mwapu, D. N. (2013). Participatory disease surveillance in the detection of trans-boundary animal diseases (TADS) in Borno State of arid north-eastern Nigeria. *Bulletin of Animal Health and Production in Africa*, *61*(2), 231–239.

Participatory epidemiology methods including simple ranking, transect walk, and interview with key informants were employed in 35 communities in north-eastern Nigeria to detect animal diseases, particularly trans-boundary animal diseases.

- The most frequently occurring disease affecting sheep and goats in the study area was Peste des Petits Ruminants (PPR). This was followed by foot rot, mange, babesiosis, orf, helminthosis, CCPP, cowdrioisis, sheep pox, cysticercosis, cutaneous larva migration, and bloat.
- PPR was estimated to have a morbidity rate of 73%, mortality rate of 62% and a case fatality rate of 84%.

Jagun, A., & Onoja, A. B. (2011). The current status of Peste des Petits Ruminant (PPR) in sheep in Ibadan southwestern Nigeria. *Journal of Commonwealth Veterinary Association*, *27*(2), 133–138.

Cross-sectional seroprevalence study of 200 sheep not previously vaccinated for PPR. Two breeds represented. Unspecified strategy for sampling animals.

- 73% of sheep had detectable antibody levels for PPR.

Ishag, O. M., Saeed, I. K., & Ali, Y. H. (2015). Peste des petits ruminants outbreaks in White Nile State, Sudan. *The Onderstepoort Journal of Veterinary Research*, *82*(1), E1-4. https://doi.org/10.4102/ojvr.v82i1.897

A longitudinal study of eight outbreaks of peste des petits ruminants (PPR) in sheep and goats reported in White Nile State, Sudan, between 2008 and 2009. Serum samples (209) were collected and tested by competitive enzyme-linked immunosorbent assay.

- A mortality rate of 4.2% was reported across eight different outbreaks.
- Out of 209 serum samples tested, 113 (54%) were found positive for PPR virus.

Morakinyo, O. A., & Babalobi, O. O. (2013). Participatory appraisal of Peste des Petite Ruminants (PPR) outbreaks in Iseyin local government area of Oyo State, Nigeria. In *Southern African Society for Veterinary Epidemiology and Preventive Medicine* (pp. 28–33). KZN Dolphin Coast, South Africa.

Participatory Appraisal approach (that enables local people to identify their own animal health problems and make decisions on possible solutions), was applied to ascertain the livestock farmer's awareness of PPR after informal reports of an outbreak in a sub-urban town in Nigeria. Qualitative research included focus groups with goat farmers and sellers and key informant interviews.

- Goat-owning respondents symptomatically identified PPR, diarrhoea, mange, and tetanus as the major diseases and health problems in the community, with PPR identified as the major disease by almost all the respondents.
- There was a considerable awareness of the disease amongst the farmers and goat sellers who had experienced outbreaks of the disease. Most farmers and goat sellers were unaware of PPR until they experienced outbreaks in their flocks. There was little awareness of preventive measures (including vaccination).
- Participatory methods can be applied to research and disease surveillance.

Adedeji, A. J., Dashe, Y., Akanbi, O. B., Woma, T. Y., Jambol, A. R., Adole, J. A., Okewole, P. (2019). Co-infection of peste des petits ruminants and goatpox in a mixed flock of sheep and goats in Kanam, North Central Nigeria. *Veterinary Medicine and*

Science, vms3.170. https://doi.org/10.1002/vms3.170

Outbreak investigation of a single outbreak of sheep and goats of a smallholder farmer in North Central Nigeria. Nine sera and seven tissue samples were collected for virus isolation.

- This is the first report of concurrent infection of PPR and GTP in mixed flock of sheep and goats in Nigeria.

Squire, S. A., Yang, R., Robertson, I., Ayi, I., Squire, D. S., & Ryan, U. (2018). Gastrointestinal helminths in farmers and their ruminant livestock from the Coastal Savannah zone of Ghana. _Parasitology Research_, _117_(10), 3183–3194. https://doi.org/10.1007/s00436-018-6017-1

To identify the gastrointestinal helminths of veterinary, zoonotic and public health importance in farmers and their ruminant livestock in Ghana, faecal samples were collected from 95 farmers and their livestock (cattle = 328, sheep = 285 and goats = 217) and examined by microscopy and/or molecular techniques.

- Gastrointestinal parasites were identified in a total of 64.3% (n = 534, CI = 61–67.6) of the 830 livestock, with strongylid nematodes the most prevalent (56.6%), followed by *Paramphistomum* spp. (16.9%). Strongylid nematodes were the most prevalent helminths detected in cattle (65.9%), sheep (52.1%) and goats (49.5%).

Kanouté, Y. B., Gragnon, B. G., Schindler, C., Bonfoh, B., & Schelling, E. (2017). Epidemiology of brucellosis, Q Fever and Rift Valley Fever at the human and livestock interface in northern Côte d'Ivoire. Acta Tropica, 165, 66–75.

A cross-sectional cluster survey conducted in three stages from 2012-2014 designed to measure the seroprevalence of brucellosis, Q Fever, and Rift Valley fever in cattle, small ruminants, and humans in northern Côte d'Ivoire. Sample of 633 cattle, 622 small ruminants, and 88 people across 63 villages.

- The seroprevalence of Q fever was 9.4% in sheep and 12.4% in goats. The seroprevalence of RVF was 2.4% in sheep and 0% in goats.
- Seroprevalences were within or below expected ranges found in the literature for West Africa.
- Small ruminants within the study were seronegative for *B. melitensis*. Livestock brucellosis in West Africa has focused on cattle as a reservoir and *B. melitensis* (affecting small ruminants) has been reported in North, East, and Central Africa only.

- Abortions were reported in most of the herds but not significantly associated with Q fever or brucellosis seropositivity.
- Identifies a need to measure the economic impact of these three zoonoses.

Bell-Sakyi, L., Koney, E. B. M., Dogbey, O., & Walker, A. R. (2004). Ehrlichia ruminantium seroprevalence in domestic ruminants in Ghana; I. Longitudinal survey in the Greater Accra Region. *Veterinary Microbiology*, *100*(3–4), 175–188. https://doi.org/10.1016/j.vetmic.2004.02.010

Longitudinal survey (34 months) with collection of sera from 3,243 cattle, goats, and sheep from smallholder and commercial owners in Accra region of Ghana.

- Virtually all cattle on the survey farms were exposed to *E. ruminantium* without suffering disease but a substantial proportion of sheep and goats escaped exposure and thus formed a susceptible population.
- *Amblyomma variegatum* tick vectors were present on livestock in rural areas throughout the year, and first seroconversion occurred at any age, although the majority of calves seroconverted between 1 and 10 months old, sheep by 11 months, and goats by 7 months.
- *E. ruminantium* was detected in brains of 14, 36 and 4% of cattle, sheep and goats submitted for post mortem.

Chah, J. M.Obi, U. P. and Ndofor-Foleng, H. M. (2013). Management practices and perceived training needs of small ruminant farmers in Anambra State, Nigeria. *African Journal of Agricultural Research*, *8*(22), 2713–2721. https://doi.org/10.5897/AJAR2013.7209

Cross-sectional questionnaire administered to 60 goat farmers (randomly selected from 6 purposively selected villages).

- Majority (62%) of the farmers indicated that diarrhoea was the most frequently observed health problem in their flocks. Fifty-five percent of the respondents reported "bloat" as a frequent health problem.

Craighead, L., Meyer, A., Chengat, B., Musallam, I., Akakpo, J., Kone, P., Häsler, B. (2018). Brucellosis in West and Central Africa: A review of the current situation in a changing landscape of dairy cattle systems. *Acta Tropica*, *179*, 96–108. https://doi.org/10.1016/j.actatropica.2017.12.026

Systematic literature review of brucellosis in dairy cattle with secondary focus on small ruminants in fourteen West and Central African countries (Benin, Burkina Faso, Cameroon, Central African Republic, Côte d'Ivoire, Gabon, Ghana, Mali, Mauritania, Niger, Rwanda, Senegal, Chad & Togo). Review supplemented with opinions from expert workshops.

- Minimal surveillance and control activities for brucellosis taking place across the region.
- Low individual seroprevalence and moderate to high herd seroprevalence is reported in cattle suggesting a state of endemicity with relatively low transmission rates.
- Although variable and scarce, available literature suggests a reasonably high burden of brucellosis in the cattle populations of the 14 study countries. There is insufficient data to conclude any meaningful estimates of the burden in small ruminant populations.
- High seroprevalence estimates seen in developing systems such as those reported in peri-urban dairies in Dakar, Senegal (25% seroprevalence ± 4.9%) are concerning.
- Workshop attendees agreed that cattle vaccines were needed, but opinions were mixed on whether vaccination in small ruminants was necessary.

Germeraad, E. A., Hogerwerf, L., Faye-Joof, T., Goossens, B., van der Hoek, W., Jeng, M., Secka, O. (2016). Low Seroprevalence of Brucellosis in Humans and Small Ruminants in the Gambia. *PLOS ONE*, *11*(11), e0166035. https://doi.org/10.1371/journal.pone.0166035

Cross-sectional serosurvey of Brucellosis in humans and small ruminants in the Gambia. Serosurvey of 599 humans and 623 small ruminants from 12 rural villages in addition to questionnaire about risk factors. Additional serosurvey of 500 small ruminants from urban abattoirs and livestock markets.

- One human and 14 sheep sera were positive by the Rose Bengal Test. Other tests were negative.
- The hypothesis that brucellosis is endemic in the investigated rural and urban areas of The Gambia could not be confirmed in the present study.
- Seropositivity for *Brucella* spp. was found in only a very small percentage of humans and small ruminants in rural and urban Gambian sites, although risk factors for obtaining *Brucella* infection such as raw milk consumption were present.

Boussini, H., Lamien, C. E., Nacoulma, O. G., Kaboré, A., Poda, G., & Viljoen, G. (2014). Prevalence of Rift Valley fever in domestic ruminants in the central and northern regions of Burkina Faso. *Revue Scientifique et Technique (International Office of Epizootics)*, *33*(3), 893–901.

Cross sectional study to determine the seroprevalence of Rift Valley fever in cattle, sheep and goats in selected areas of northern and central Burkina Faso. Sample size was 520 serum samples.

- The overall seroprevalence was 7.69% (40/520): the prevalence in the northern region was 9.37% and, in the central region, 5%.
- In the northern region, the overall seroprevalences were 15%, 8.33% and 6.66% for cattle, sheep and goats respectively, and in the central region overall seroprevalences were 15%, 5% and 0% respectively.
- Highest seroprevalence was found in the northern region around temporary ponds and dams.

Isaac, C., Ohiolei, J. A. A., Ebhodaghe, F., Igbinosa, I. B. B., & Eze, A. A. A. (2017). Animal African Trypanosomiasis in Nigeria: A long way from elimination/eradication. *Acta Tropica*, *176*, 323–331. https://doi.org/10.1016/j.actatropica.2017.08.032

Review of AAT studies in Nigeria and other West and Central African countries. Many sources are pre-2002 and results with regards to seroprevalence vary widely with no attempt to address differing methods (e.g.: whether survey was in response to outbreak). The economic model incorporates two published assumptions about effect on GDP and output elasticity of livestock stock but could be more clearly explained (e.g.: Inconsistencies in figure column totals).

- Reviews of serosurveys include ranges of seroprevalence in sheep from 1.2%-40.9% and in goats from 0.7%-75%.
- Estimates of impacts of AAT on agricultural GDP for 10 West and Central African countries ranges from 800 million USD to 1.6 billion USD (assuming 40% and 80% AAT impacts, respectively).

Carlson, C. J., Kracalik, I. T., Ross, N., Alexander, K. A., Hugh-Jones, M. E., Fegan, M., Blackburn, J. K. (2019). The global distribution of Bacillus anthracis and associated anthrax risk to humans, livestock and wildlife. *Nature Microbiology*. https://doi.org/10.1038/s41564-019-0435-4

Global incidence database of human, wildlife, and livestock anthrax cases from multiple sources spanning 71 countries from 1914-2018.

- Using past occurrence as a proxy for risk, in Sub-Saharan Africa, an estimated 30.5 million cattle, 22.4 million goats, and 14.5 million sheep are at risk for anthrax.
- Vaccination rates in Sub-Saharan Africa are 0-6%.
- Vaccination may be less effective for the 31 million livestock and 4.6 million poor livestock keepers in West Africa, where an endemic lineage of *B. anthracis* shares an anthrose-deficiency mutation that has been hypothesized to lead to a vaccine escape.
